# B-spline curve fitting based on dynamic adjustment of knot vector using feature points

**DOI:** 10.1371/journal.pone.0325458

**Published:** 2025-06-27

**Authors:** Xiaobing Chen, Shuxin Guo, Rongrong Wang, Chuangchuang Zhang, Jianchu Lin, Shang Chen

**Affiliations:** Huaiyin Institute of Technology, Huai’an, Jiangsu, China; Dartmouth College Geisel School of Medicine, UNITED STATES OF AMERICA

## Abstract

An essential challenge in B-spline curve fitting is how to produce a B-spline curve that satisfies the accuracy requirement with a minimal number of knots and control points. This paper suggests a better algorithm based on feature points method. During the curve approximation process, the projection points of data points and their parameters are calculated, and the data point parameters are corrected to achieve dynamic adjustment of the knot vector. At the same time, traditional methods are improved in terms of initial feature point selection and new feature points determination. The experimental results indicate that the B-spline curve produced using the method in this work has higher fitting accuracy, fewer control points, and shorter fitting time.

## Introduction

Methods for modifying the shape of a B-spline curve include changing control points and knot vector. Generating a B-spline curve that meets the accuracy requirements with a small number of knots and control points is an important problem in B-spline curve fitting [1–5]. Park *et al*. [6] proposed a dominant points method, denoted as DOM, for B-spline approximation. Zhang *et al*. [7] proposed a new geometric iterative fitting method, which possesses the advantages of least squares iterative approximation method, can flexibly adjust the number of control points and knot vector. B-spline curve fitting technology has been widely used in tool path generation in CNC machining [8–10]. Hu *et al*. [11] proposed a cubic B-spline curve fitting method to deal with linear tool paths. The fitting error estimation scheme based on the Newton method and the dominant points insertion scheme based on fitting error constrain were proposed to modify the B-spline, which achieve data compression. The number of control points of curve fitting by these methods is smaller than the traditional least square fitting method. However, the knot vector is calculated according to the parameter values of the feature points that remain unchanged throughout the curve fitting process, which may still result in more fitting times and control points [12–16]. This paper proposes an improved B-spline curve fitting method based on dynamic adjustment of knot vector, which is denoted as DAKM algorithm. The DAKM algorithm enhances the dominant points fitting algorithm by optimizing the initial feature point selection, feature points parameters adjustment, and new feature points determination. Experimental results demonstrate that the DAKM algorithm achieves higher fitting accuracy, fewer control points and shorter fitting time for B-spline curves.

## Preliminaries

### Definition of B-spline curve

A parametric B-spline curve p(u) of degree *k* is defined by [11]:

$$p(u)=\sum_{i=0}^{n}\;d_{i}N_{i,k}(u),u\in [u_{k},u_{n+1}]$$ (1)

where $d_{i}\;(i=0,1,...,n)$ are control points of B-spline curve of degree *k*, and *N*_*i*,*k*_(*u*) are the basic function of B-spline curve, and knot vector $U=\left[u_{0},u_{1},...,u_{n+k+1}\right]$ is defined as follows:

$$\left\{ \begin{array}{c} N_{i,0}(u)=\left\{ \begin{array}{cc} 1, & u_{i}\leq u\leq u_{i+1}\\ 0, & otherwise \end{array}\right.\\ N_{i,k}(u)=\frac{u-u_{i}}{u_{i+k}-u_{i}}N_{i,k-1}(u)+\frac{u_{i+k+1}-u}{u_{i+k+1}-u_{i+1}}N_{i+1,k-1}(u) \end{array}\right.$$ (2)

By adjusting knot vector, control points, the local shape of B-spline curve can be changed.

### Determination of knot vector

When defining a B-spline curve of degree *k* , knot vector need to be determined. The knots repeatability of knot vector at both sides is *k*+1, namely $u_{0}=u_{1}=...=u_{k}=0$,$u_{n+1}=u_{n+2}=...=u_{n+k+1}=1$.

The commonly methods for parameterization are uniform parameterization, cumulative chord length parameterization, centripetal parameterization, corrected string length parameterization [17]. When control points are unknown, the knot values are calculated from the data points to be fitted. It is necessary to parameterize the given data points, and the parameterization of the data points has an important impact on fitting accuracy and efficiency of the curve [11,12]. Set $p_{i}\;(i=0,1,...,m)$ be data points to be fitted, the data points are parameterized using cumulative chord length method, then we have:

$$\left\{ \begin{array}{c} {\tilde{u}}_{0}=0\\ {\tilde{u}}_{i}=\sum_{j=0}^{i-1}\left|p_{j+1}-p_{j}\right|/\sum_{j=0}^{m-1}\left|p_{j+1}-p_{j}\right|,i=1,2,\ldots ,m \end{array}\right.$$ (3)

So the inner values of the knot vector is [17,18]:

$$u_{k+i}=\{\begin{array}{c} \frac{1}{k}\overset{i-k-1}{\underset{j=i}{\sum }}{\tilde{u}}_{j},m=n\\ (1-\alpha ){\tilde{u}}_{i-1}+\alpha {\tilde{u}}_{i},m>n \end{array}i=1,2,...,n-k$$ (4)

where d=(m+1)/(n−k+1), i=int(jd), α=jd−i, the inner values determined by the above equation guarantees that each knot interval possess at least one data point parameter ${\tilde{u}}_{i}$. When *m*=*n*, the inner values computation method is called AVG (Averaging Technique) method, when *m*>*n*, the inner values computation method is called KTP (Knot Placement Technique) method.

### De-Boor algorithm of B-spline curve

In this paper, the points corresponding to the minimum distances from data points to B-spline curve are called projection points of data points. When calculating these projection points and their parameters, it is necessary to calculate the points on B-spline curve and their derivatives. Here De-Boor algorithm is used, the recursive formula is [17]:

$$p(u)=\sum_{j=0}^{n}\;d_{i}N_{j,k}(u)=\sum_{j=i-k}^{i-l}\;d_{j}^{l}N_{j,k-1}(u)=...=d_{i-k}^{k},u\in \left[u_{i},u_{i+1}\right]$$ (5)

Where

$$d_{j}^{l}=\{\begin{array}{l} d_{j},l=0\\ (1-a_{j}^{l})d_{j}^{l-1}+a_{j}^{l}d_{j+1}^{l-1},j=i-k,i-k+1,...,i-l;l=1,2,...,k \end{array}$$ (6)

$$a_{j}^{l}=\frac{u-u_{j+l}}{u_{j+k+1}-u_{j+l}}$$ (7)

The recursive formula for calculating *r*-order derivatives of B-spline curve is [17,18]:

$$p^{r}\;(u)=\frac{\partial^{r}}{\partial u^{r}}\sum_{j=0}^{n}\;d_{j}N_{j,k}(u)=\sum_{j=i-k}^{i-r}\;d_{j}^{r}N_{j,k-r}(u),u\in \left[u_{i},u_{i+1}\right]$$ (8)

$$d_{j}^{l}=\{\begin{array}{l} d_{j},l=0\\ (k-l+1)\frac{d_{j+1}^{l-1}-d_{j}^{l-1}}{u_{j+k+1}-u_{j+l}},j=i-k,i-k+1,\ldots ,i-r;l=1,2,\ldots ,r \end{array}$$ (9)

Formula (8) shows that the r-order derivatives of B-spline curve of degree *k* can be expressed as B-spline curve of degree (*k*–*r*), which recursive calculation of controls points is according to Formula (9), and its knot vector is:

$$U^{r}=\left[u_{0}^{r},u_{1}^{r},\ldots ,u_{n+k-2r+1}^{r}\right]=[\underset{k+1-r}{\underbrace{0,...,0,}}u_{k+1},\ldots ,u_{n},\underset{k+1-r}{\underbrace{1,..\mathrm{.1}}}]$$ (10)

The 1st and 2nd order derivatives of endpoints of the *k*-degrees B-spline curve can be obtained:

$$\left\{ \begin{array}{c} p^{\prime }\left(u_{k}\right)=\frac{k\left(d_{1}-d_{0}\right)}{u_{k+1}-u_{1}}\\ p^{\prime }\left(u_{n+1}\right)=\frac{k\left(d_{n}-d_{n-1}\right)}{u_{n+k}-u_{n}} \end{array}\right.$$ (11)

$$\left\{ \begin{array}{c} p^{\prime \prime }\left(u_{k}\right)=k(k-1)\frac{\frac{d_{2}-d_{1}}{u_{k+2}-u_{2}}-\frac{d_{1}-d_{0}}{u_{k+1}-u_{1}}}{u_{k+1}-u_{2}}\\ p^{\prime \prime }\left(u_{n+1}\right)=k(k-1)\frac{\frac{d_{n}-d_{n-1}}{u_{n+k}-u_{n}}-\frac{d_{n-1}-d_{n-2}}{u_{n+k-1}-u_{n-1}}}{u_{n+k-1}-u_{n}} \end{array}\right.$$ (12)

### B-spline curve fitting using feature points

When fitting B-spline curve using feature points, a B-spline curve with less error can be obtained with the same number of control points. Conversely, fewer control points are required for the same fitting accuracy, compared to other methods. The feature point algorithms usually take four main steps: parameterization, feature point selection, knot placement, and least squares fitting. These approaches are different from the conventional approaches in knot placement and feature point selection [17].

When selecting initial feature points, the local curvature method is used to determine the initial feature points. With a given initial fitting accuracy, a B-spline basis curve is fitted firstly, then the curvature at each data point is calculated based on the basis curve. The initial feature point linked list is formed by the local curvature maximum points, the first and last points of the data points. If the curvature of the basis curve changes frequently, the initial feature points determined by the local curvature maximum may result in more control points. This is because the number of feature points is equal to the number of control points, and more feature points correspond to more control points in these algorithms.

According to the parameters of feature points, the knot vector is calculated using the average parameter method (AVG method). The least square method is used to fit the data points and fitting error is computed. However, a single fitting might not always meet the desired accuracy. In such cases, a new feature point is added, the knot vector is recalculated, and the least squares fitting is performed again. This fitting process is repeated until the desired fitting accuracy is achieved. Set $e_{\max}$ to the maximum deviation between the data points and the fitted curve, then we have:

$$e_{\max}=\max(|p({\tilde{u}}_{i})-p_{i}|),i=1,2,...,m$$ (13)

Since $p({\tilde{u}}_{i})$ is usually not the closest point on the curve with *p*_*i*_, therefore, $e_{\max}$ may not be the actual maximum deviation of the data point and the fitted curve. On the other hand, in the entire curve fitting process, the feature points parameters remain unchanged. These may produce more feature points during the fitting process, resulting in more fit times and control points [18–21].

## The DAKM algorithm

### Steps of DAKM algorithm

**Fig 1:**
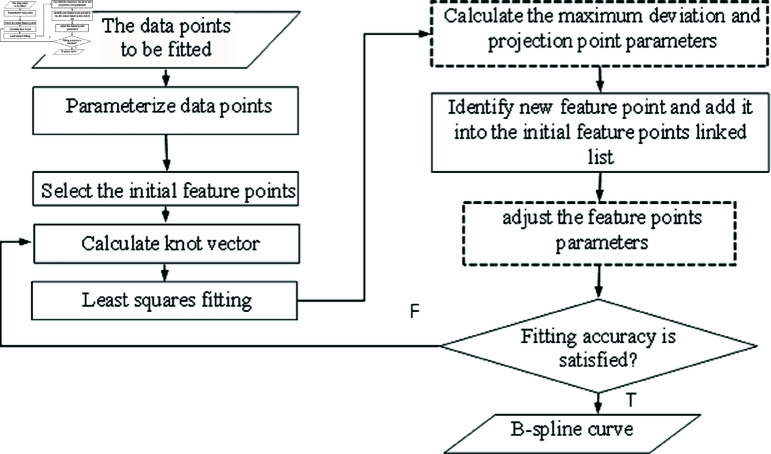
Fitting flow of DAKM algorithm.

Fig 1 illustrates the fitting flow of DAKM algorithm. The biggest difference between DAKM algorithm and other feature point algorithms lies in the calculation of maximum deviation, projection point parameters, and feature point parameter correction. In addition, this algorithm has improved existing algorithms in terms of initial feature point selection and new feature point determination. In the fitting process, in order to calculate the maximum deviation between the fitted data points and the fitted curve, the Newton iteration method is used to calculate the points on the fitted curve that are closest to the fitted data points, and these points on the fitted curve are called the projection points of the fitted data points. When the projection points and its parameters are determined, the projection point parameters are used to correct the parameters of the feature points. In the subsequent fitting process, the parameter values of the fitted data points for calculating knot vectors are dynamically changing, which is fundamentally different from the traditional feature points methods. The steps of the algorithm are as follows:

(1) Data points parameterization. The parameters of the data points are calculated by accumulated chord length parameterization.
(2) Select the initial feature points. The curvature threshold segmentation is used to select the initial feature points. The local curvature maximum points, the first and last points of the data points are used as initial feature points (details in subsection Selection of initial feature points).
(3) Based on the parameters of the feature points, the knot vector is calculated using the average parameter method (details in subsection Knot vector calculation and localization analysis).
(4) The least square method is used to fit the data points and fitting error is computed.
(5) Calculate the maximum deviation and projection point parameters (details in subsection Calculation of projection points for B-spline fitting curve).
(6) Identify new feature point and add it into the initial feature points linked list, and adjust the parameters of feature points (details in subsection New feature point determination and its parameter adjustment).
(7) If the fitting error is less than the desired accuracy, the fitting curve is obtained. Otherwise, a new feature point is determined, and added to feature point list. Then, Step (3) is repeated until the fitting error is within the desired value.

### Selection of initial feature points

The data points are preprocessed before selection of initial feature points. Firstly, the distance is calculated between the data point and the previous data point in sequence,then the data point is deleted if the distance of adjacent data points is less than the fitting error. The DOM algorithm uses the local curvature extremum method to determine the initial feature points. Firstly, a B-spline basis curve is fitted based on the given initial fitting accuracy. Then, by calculating the curvature of the basis curve at each data point, an initial feature point linked list is formed, including the local curvature maximum point and the first and last points of data points.

The curvature threshold segmentation method is used to select the initial feature points in DAKM algorithm. Firstly, calculate the curvature radius of the data points sequentially based on the fitted B-spline basis curve. Then, given a curvature radius threshold, data points are segmented into several regions. Finally, in each data region, calculate the average value of curvature radius, and the point corresponding to the curvature radius closest to the average value is the feature point in that data region. Let the curvature radius of the data point along the tangent direction be $\rho_{F_{i}}\;(i=0,1,...,m)$, the curvature radius $\rho_{F_{i}}$ is divided into n regions called $\Delta \rho_{F_{j}}\;(j=0,1,...,n)$, the curvature radius in th region is $\rho_{Fi}\;(j_{0}\leq i\leq j_{0}\;+\;s\;-\;1)$, *j*_0_ is the serial number of the starting curvature radius in the region, *s* is the number of curvature radius. The basic steps for initial feature points selection are as follows:

(1) Iterate over the curvature radius $\rho_{F_{i}}$, dividing it into *n* regions. Set δ be the fitting accuracy, $\rho_{th}$ is the threshold of radius curvature, the specific formula is $\rho_{th}=2\delta \rho_{Fj_{0}}$, if $\left|\rho_{Fi+1}-\rho_{Fi}\right|<\rho_{th}$, $\rho_{Fi}$ belongs to the same region.
(2) Calculate the average curvature radius ${\bar{\rho }}_{j}$ of the region $\;\;\;\Delta \;\;\;\rho_{Fj}$.
(3) Calculate the minimum between $\rho_{Fi}$ and ${\bar{\rho }}_{j}$ in region, then we have:
  $$\;\;\;\Delta \;\;\;\rho_{\min j}=\min\left(\left|\rho_{F_{j}}-{\bar{\rho }}_{j}\right|\right)$$ (14)
(4) Assuming that the curvature radius number corresponding to $\;\;\;\Delta \;\;\;\rho_{\min j}$ is *f*(*j*), and the feature points are $q_{j}\;(j=0,1,...,n_{0})$, then the feature point corresponding to region $\;\;\;\Delta \;\;\;\rho_{Fj}$ is selected as:
  $$q_{j}=p_{f(j)}$$ (15)

### Knot vector calculation and localization analysis

If KTP method is used to determine the knot vector, the planned knot vector may contain more node values [22–26]. Both DOM algorithm and DAKM algorithm employ the AVG method to calculate the knot vector, based on the feature point parameters. Supposing the degree of fitting curve is *K*, the parameter of point *p*_*i*_ is ${\tilde{u}}_{i}$, $U=\left[u_{0},u_{1},...,u_{n+k+1}\right]$ is a knot vector and the parameter value for feature point *q*_*j*_ is ${\tilde{u}}_{f(j)}$, then the inner knot values of the DAKM algorithm is:

$$u_{k+i}=\frac{1}{k}\sum_{j=i}^{i+k-1}{\tilde{u}}_{f(j)},i=1,2,\ldots ,n-k$$ (16)

Data points often need to be fitted multiple times to meet accuracy requirements. After each fitting, DOM and DAKM algorithms can lead to a change in the number and values of nodes in knot vector due to the addition of new feature points. In each fitting process of DAKM algorithm, the feature points are obtained from the fitted data points, the projection points and their parameters for the fitted data points are calculated, and the data points parameters are replaced with the projection points parameters. That is to say, the feature point parameters are corrected during the fitting process (refer to subsection New feature point determination and its parameter adjustment for more details). However, the DOM algorithm keeps the parameters of the feature points constant.

The knot vector computed by DAKM algorithm contains fewer knot values and possesses locality. Suppose the newly added feature point is *q*_*w*_, the corresponding parameter is ${\tilde{u}}_{w}$, ${\tilde{u}}_{j_{0}}<{\tilde{u}}_{w}<{\tilde{u}}_{j_{0}+1}$. The feature points after the new feature point is added are denoted as $q_{j}^{new}\;(j=0,1,...,n_{0}+1)$, then we have:

$$q_{j}^{new}=\{\begin{array}{l} q_{j},0<j<j_{0}\\ q_{w},j=j_{0}+1\\ q_{j-1,}j_{0}+2\leq j\leq n_{0}+1 \end{array}$$ (17)

The parameters for the new feature point are:

$${\tilde{u}}_{j}^{new}=\{\begin{array}{l} {\tilde{u}}_{j},0<j<j_{0}\\ {\tilde{u}}_{w},j=j_{0}+1\\ {\tilde{u}}_{j-1},j_{0}+2\leq j\leq n_{0}+1 \end{array}$$ (18)

The inner knot values of the new knot vector are:

$$u_{i+k}^{new}\}=\frac{1}{k}\sum_{j=i}^{i+k-1}{\tilde{u}}_{j}^{new}\},i=1,2,...,n_{0}-k+1$$ (19)

By the Formulas (18) and (19), the relationship between the new knot vector and the original knot vector can be obtained:

$$u_{i+k}^{new}=\{\begin{array}{l} u_{i+k},\;i=1,2,...,j_{0}-k+1\\ \frac{1}{2}\sum_{j=i}^{i+k-1}{\tilde{u}}_{j}^{new},i=j_{0}-k+2,j_{0}-k+3,...,j_{0}+1\\ u_{i+k-1},\;\;i=j_{0}+2,...,n_{0}-k+1 \end{array}$$ (20)

From the above formula, it can be seen that the number of nodes in the knot vector may increase by 1 and at most *k* node values may change after inserting a new feature point. The knot vector has locality, which means that impact of a new feature point on the fitting curve is local.

## Calculation of projection points for B-spline fitting curve

The projection points and their parameters of data points are calculated in DAKM algorithm during fitting process. Set ${\tilde{u}}_{i}$ as the parameter value corresponding to the data points $p_{i}\;(i=0,1,...,m)$ to be fitted, and accumulation chord length method is used to parameterize the data points. Set *n* be the number of control points and the knot vector be $U=\left[u_{0},u_{1},...,u_{n+k+1}\right]$. Set $d_{j}\;(j=0,1,...,n)$ be the control points to be solved, *N*_*j*,*k*_(*u*) be a B-spline basis function. Set boundary conditions as: *p*_0_ = *p*(0), *p*_*m*_ = *p*(1), $d_{0}=p_{0}$, $d_{n}=p_{m}$. By least squares method, the objective function is obtained:

$$E(d_{1},...,d_{n-1})=\;\sum_{i=1}^{m-1}{\left[p_{i}-p({\tilde{u}}_{i})\right]}^{2}$$ (21)

The partial derivative of the above equation is calculated and its value is set to zero, the following equation with control points $d_{j}\;(j=1,2,...,n-1)$ as variables can be obtained:

$$(N^{T}N)\left[\begin{array}{c} d_{1}\\ d_{2}\\ \vdots \\ d_{n-1} \end{array}\right]=R$$ (22)

where,

$$N=\left[\begin{array}{ccc} N_{1,k}({\tilde{u}}_{1}) & \ldots & N_{n-1,k}({\tilde{u}}_{1})\\ \vdots & \ddots & \vdots \\ N_{1,k}({\tilde{u}}_{m-1}) & \cdots & N_{n-1,k}({\tilde{u}}_{m-1}) \end{array}\right]$$ (23)

$$R=\left[\begin{array}{ccc} {N_{1}}_{,k}({\tilde{u}}_{1})r_{1} & \ldots & {N_{1}}_{,k}({\tilde{u}}_{m-1})r_{m-1}\\ \vdots & & \vdots \\ {N_{n-1}}_{,k}({\tilde{u}}_{1})r_{m-1} & \cdots & {N_{n-1}}_{,k}({\tilde{u}}_{m-1})r_{m-1} \end{array}\right]$$ (24)

In the above equation,

$$r_{i}=p_{i}-p_{0}N_{0,k}({\tilde{u}}_{i})-p_{m}N_{n,k}({\tilde{u}}_{i}),i=1,2,...,m-1.$$ (25)

The fitting error is the maximum distance from all data points to the fitted curve, where the distance from the data point to the fitted curve needs to be calculated iteratively [27,28]. Let the fitting error be ε, the fitting accuracy is δ, then there is accuracy control condition:

$$\varepsilon =\underset{0\leq i\leq m}{\max}\;(\underset{0\leq u\leq 1}{\min}\;|p_{i}-p(u)|)\leq \delta$$ (26)

**Fig 2:**
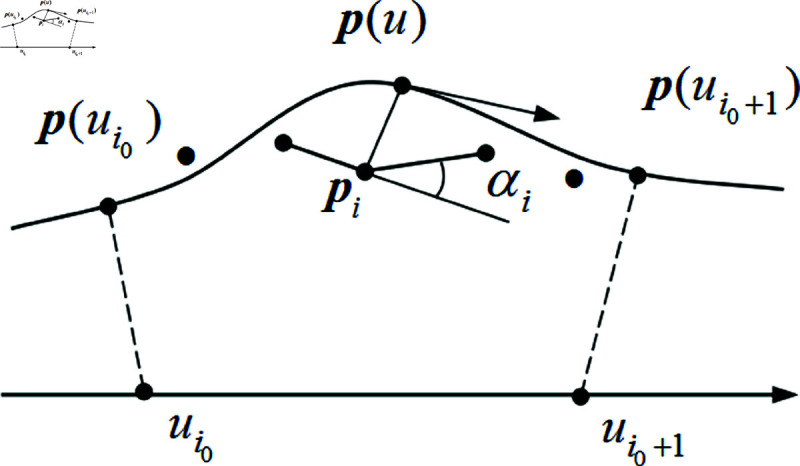
Fitting deviation calculation of data points.

To calculate the fitting error, it is necessary to determine the projection points of each data point and its parameters [29]. In Fig 2, there is the objective function:

$$E(u)=p^{\prime }\left(u\right)(p(u)-p_{i}),u\in \left[0,1\right].$$ (27)

When E(u)=0, the distance from *p*_*i*_ to *p*(*u*) is the shortest. Let *d*(*u*) be the distance between *p*(*u*) and *p*_*i*_, the cosine of the angle between $p^{\prime }(u)$ and *[p*_*i*_,*p*(*u*)*]*, and $\varepsilon_{c}$ be a tiny quantity, then the termination condition of iteration is $c(u)<\varepsilon_{c}$ [30,31]. Here, *c*(*u*) is the cosine of the angle between $p^{\prime }(u)$ and *[p*_*i*_,*p*(*u*)*]*. Set the iterative initial value be ${\tilde{u}}_{i}$, after *r* iterations, parameter is ${\tilde{u}}_{i_{new}}$, then we have:

$$u_{r}=u_{r-1}-\frac{p^{\prime }(u_{r-1})(p(u_{r-1})-p_{i})}{p^{\prime \prime }(u_{r-1})(p(u_{r-1})-p_{i})+|p^{\prime }(u_{r-1}){|}^{2}},{\tilde{u}}_{i_{new}}=|u_{r}|$$ (28)

$p({\tilde{u}}_{i_{new}})$ is the projection point of data point *p*_*i*_, and its parameter is used to replace the data point parameter. Parameter *u*_*r*_ should be between 0 and 1. If *u*_*r*_ is greater than 1, no correction will be made and the initial value will be taken. For cases of non convergence, the iteration loop will be terminated without any parameter adjustments.

## New feature point determination and its parameter adjustment

After each fitting, the data point $p_{e\;\;\;\_\;\;\;\max}$ corresponding to the maximum deviation $e_{\max}$ is determined. However, using point $p_{e\;\;\;\_\;\;\;\max}$ as the new feature point directly may result in more control points, and point $p_{e\;\;\;\_\;\;\;\max}$ is often used to determine the curve segment in which the new feature point is located [32,33]. As shown in Fig 2, $p\left(u_{i0}\right)$ and $p\left(u_{i0+1}\right)$ are the segmented connection points on the parameter curve calculated based on the knot vector *U*, and *p*_*i*_ are the data points inside the curve segment $\left\{p\left(u_{i0}\right)\;p\left(u_{i0+1}\right)\right\}$, that is, the initial data points to be fitted. Then, according to the principle of “maximum angle between adjacent line segments", the data point *p*_*i*_ with the largest adjacent angle is used as the new feature point. The fitting curve that meets accuracy requirements usually needs to be fitted multiple times. Except the first fitting, a new feature point is added before each fitting. The steps for determining a new feature point are as follows [34,35]:

(1) Determine the data point $p_{e\;\;\;\_\;\;\;\max}$ corresponding to the maximum deviation $e_{\max}$.
(2) Compute the segmented connection point $p\left(u_{i}\right)$ of the curve based on the knot vector *U*.
(3) Determine the curve segment $\left\{p\left(u_{i0}\right)\;p\left(u_{i0+1}\right)\right\}$ where point $p_{e\;\;\;\_\;\;\;\max}$ is located, that is, the *x* coordinate of point $p_{e\;\;\;\_\;\;\;\max}$ is within the x coordinate range of point $p\left(u_{i0}\right)$, $p\left(u_{i0+1}\right)$.
(4) Determine data point *p*_*i*_ within the curve segment $\left\{p\left(u_{i0}\right)\;p\left(u_{i0+1}\right)\right\}$, that is, the *x* coordinate of point *p*_*i*_ is within the x coordinate range of point $p\left(u_{i0}\right)$, $p\left(u_{i0+1}\right)$.
(5) Calculate the angle $\alpha_{i}$ of adjacent line segments.
(6) Select the data point corresponding to the maximum value of $\alpha_{i}$ as the new feature point. Set i _ max to be the serial number of the selected data point, the new feature point is:
  $$q_{new}=p_{i\;\;\;\_\;\;\;max}$$ (29)

In DAKM algorithm, new feature points that have been added to the feature point linked list are marked, thus, the same new feature point will be avoided and duplicate knots are not included. After a new feature point is added, the feature points are $q_{j}\;(j=0,1,\ldots ,n+1)$. Set *g*(*j*) be the serial number of the data point corresponding to the feature point *q*_*j*_, namely the serial number of projection point, so the feature point parameter is corrected to:

$${\tilde{u}}_{i}={\tilde{u}}_{g(j)}^{\prime }$$ (30)

## Experimental verification

### Experimental data

**Fig 3:**
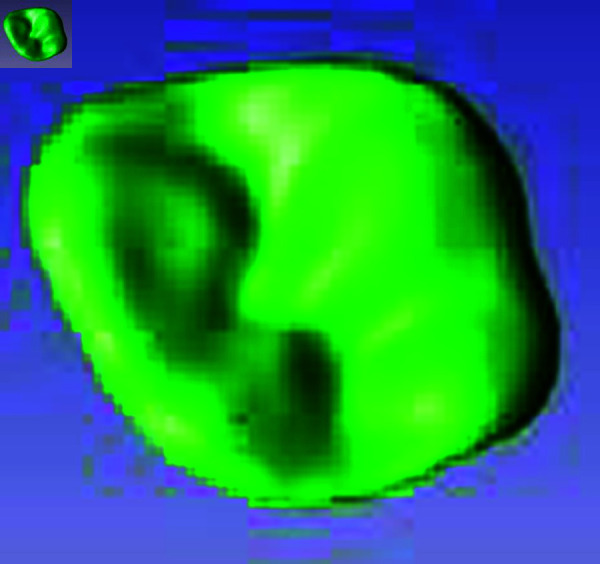
The outer surface of human molar. (Reprinted from [36] under a CC BY license, with permission from [The Science Press], original copyright [2010]).

**Fig 4:**
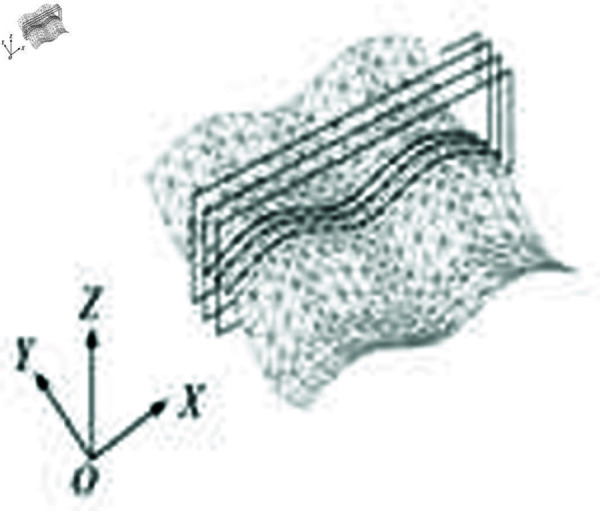
Intersection between cutting planes and a triangular mesh surface.

**Fig 5:**
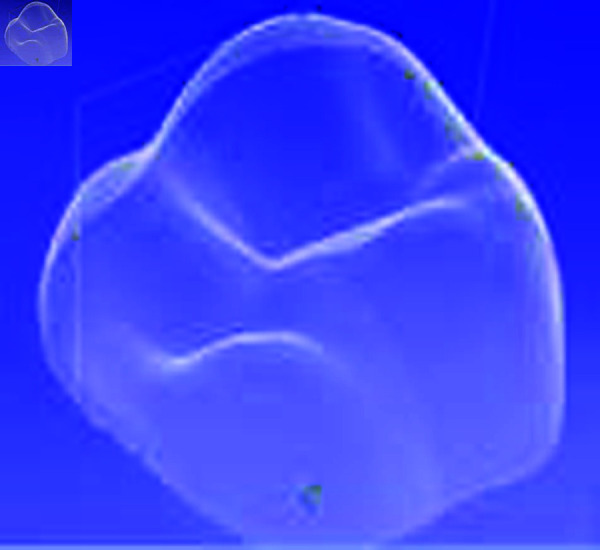
Tool path generated by the section plane method. (Reprinted from [36]under a CC BY license, with permission from [The Science Press], original copyright [2010]).

**Fig 6:**
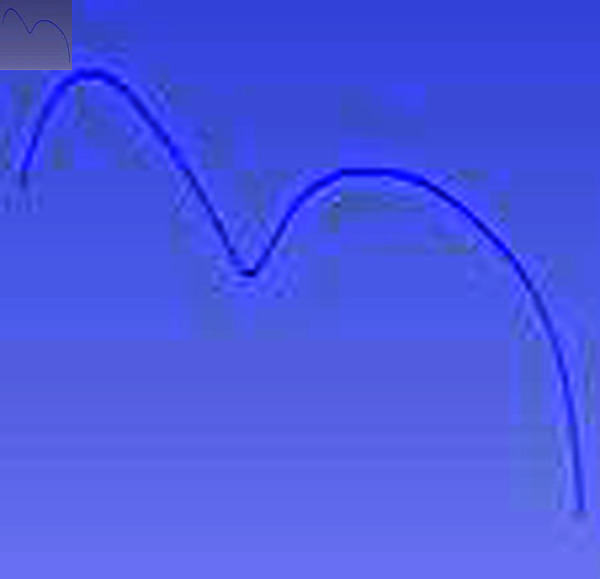
Cutter location points in plane XOZ.

The experiment is conducted under the Dental CAD/CAM prototype system developed by the author’s team in the early stage, using VC++ and HOOPs as development tools. The B-spline tool path generation for CNC machining of the outer surface of human molar is used as an example [31]. The triangular mesh model of teeth is reconstructed from 3D laser point cloud data, and Fig 3 shows the three-dimensional mesh model of molar with lighting effect in Dental CAD/CAM. The molars model is a triangular mesh model with 6064 vertices and 11887 triangular pieces, and its size of the bounding box is 15 mm × 15 mm × 6 mm.The data points to be fitted in the planes are obtained by intersecting a series of section planes and triangular mesh model, as shown in Fig 4, the discrete data points in each plane are a set of test data. In this experiment, the spacing between the section planes is 0.1 mm, with a total of 148 section planes, resulting in 148 sets of test data. Fig 5 shows the linear tool paths using the section plane method in Dental CAD/CAM. Fig 6 shows the linear tool path with section plane of *XOZ*, with a total of 163 data points.

## Experimental analysis

Traditional least squares fitting, DOM, and DAKM algorithms are used to fit B-spline curve. In traditional least squares fitting, knot vectors are calculated using KTP method. In DOM and DAKM algorithms, knot vectors are calculated using AVG method. Therefore, the traditional least squares fitting method is abbreviated as KTP algorithm.

**Fig 7:**
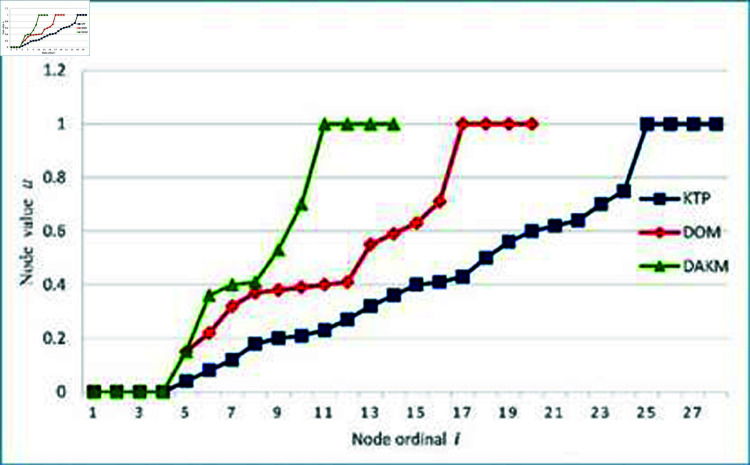
Comparison of knot value distributions for three algorithms.

The data points in the *XOZ* plane are fitted using KTP, DOM, and DAKM algorithm, respectively. Fig 7 shows a comparison of the knot value distributions of three algorithms. From the distribution of inner knot values, the KTP algorithm has a relatively uniform distribution and the largest number of nodes in knot vector, followed by the DOM algorithm, while the DAKM algorithm has a less uniform distribution and the smallest number of nodes. The more evenly and numerous the node values are distributed, the greater the number of control points are required. Table 1 shows the experimental result using different fitting algorithms, indicating that the B-spline curve fitted by the DAKM algorithm requires fewer control points, has a shorter execution time, and a smaller fitting error.

**Table 1:** Comparison of experimental data from three different algorithms.

algorithm	Number of Control points/number	Fitting error/mm	Fitting times/times	Execution time/s
KTP	24	0.0480	19	1.22
DOM	15	0.0495	8	0.43
DAKM	11	0.0475	5	0.28

**Fig 8:**
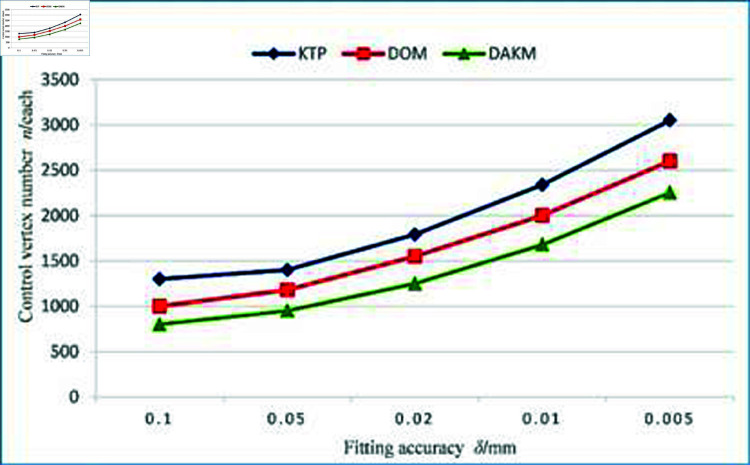
Comparison of the number of control points for the three algorithms.

Then, KTP, DOM, and DAKM algorithm are carried out to fit the data points in each section plane, and the fitting accuracy was 0.005 mm, 0.01 mm, 0.02 mm, 0.05 mm, and 0.1 mm, respectively. Fig 8 shows the number of control points required by the three algorithms at different fitting accuracies. As the fitting accuracy improves, the number of control points required by the three algorithms increases. Among the three algorithms, the DAKM algorithm requires the fewest number of control points under the same fitting accuracy.

**Fig 9:**
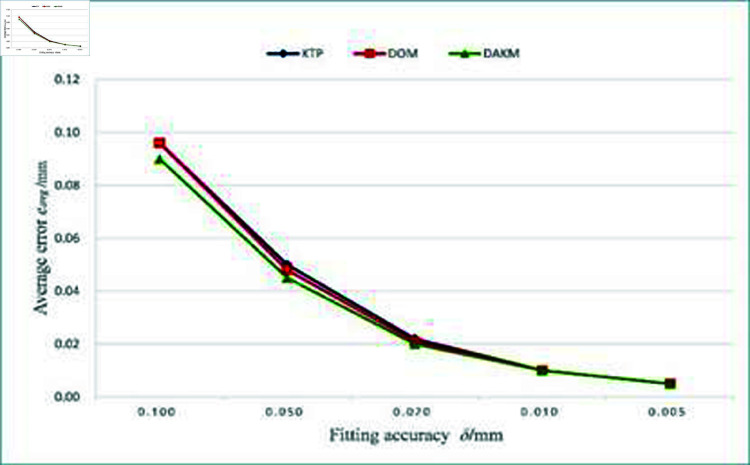
Comparison of average fitting errors for the three algorithms.

**Fig 10:**
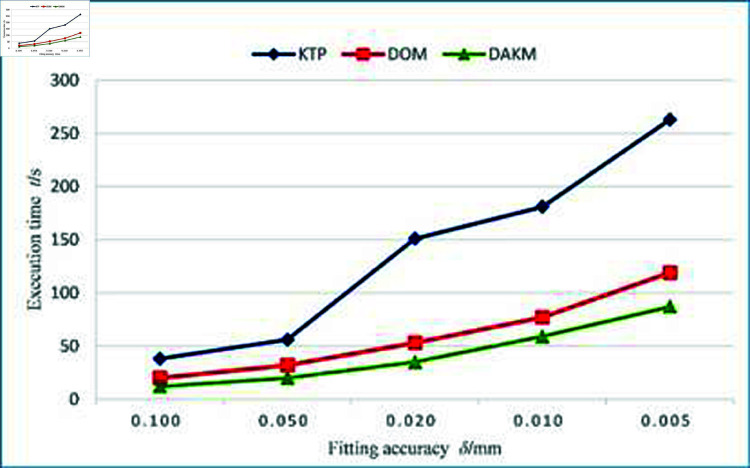
Comparison of execution time for the three algorithms.

Fig 9 shows the average fitting error of the three algorithms under different fitting accuracies. The average fitting error of the KTP and DOM algorithms is very close, while the average fitting error of the DAKM algorithm is smaller than the other two algorithms. Fig 10 shows the execution time of the three algorithms under different fitting accuracies. As the fitting accuracy improves, the execution time of the three algorithms increases. The execution time of the KTP algorithm increases rapidly, while the execution time of the DAKM algorithm is the lowest among the three algorithms under the same fitting accuracy.

## Conclusion

A B-Spline curve fitting algorithm based on dynamic adjustment of knot vector is proposed in this paper. Improvements have been made to the DOM algorithm in terms of the calculation of projection points and their parameters, and correction of feature point parameters. During the fitting process, the projection points and their parameters of the data points are iteratively calculated, and the feature point parameters are corrected to achieve dynamic adjustment of the knot vector. However, the current experimental data is two-dimensional data, and further verification is needed to determine whether it is suitable for three-dimensional spatial data points. On this basis, DAKM algorithm is applied to B-Spline curve fitting of CNC linear tool paths. The results of experiment demonstrate that B-Spline curve fitted by the method has fewer control points, fewer fitting times, and higher fitting accuracy, which has important application value.

## Supporting information

Supporting information The data set for Fig 4 (DOCX)

Supporting information The data set for Figs 5, 6, and 7 (DOCX)

Supporting information File (DOCX)

Supporting information Code (DOCX)
