# Supplementary material for: B-spline curve fitting based on dynamic adjustment of knot vector using feature points
Source: PLoS One. 2025 Jun 27;20(6):e0325458. doi: 10.1371/journal.pone.0325458 (PMC12204621; doi:10.1371/journal.pone.0325458)
Supplement: Supporting information — (DOCX) [file pone.0325458.s001.docx]

N120810 X6.57841 Y0.0 Z3.99559

N120820 X6.57136 Y0.0 Z4.0187

N120830 X6.54511 Y0.0 Z4.10933

N120840 X6.5207 Y0.0 Z4.17635

N120850 X6.50597 Y0.0 Z4.22047

N120860 X6.47893 Y0.0 Z4.29959

N120870 X6.46685 Y0.0 Z4.33587

N120880 X6.45248 Y0.0 Z4.37402

N120890 X6.41652 Y0.0 Z4.47575

N120900 X6.3934 Y0.0 Z4.53423

N120910 X6.36113 Y0.0 Z4.61327

N120920 X6.34181 Y0.0 Z4.65955

N120930 X6.30125 Y0.0 Z4.75049

N120940 X6.29649 Y0.0 Z4.7613

N120950 X6.28844 Y0.0 Z4.77757

N120960 X6.24525 Y0.0 Z4.86749

N120970 X6.13186 Y0.0 Z5.06693

N120980 X6.12228 Y0.0 Z5.08319

N120990 X6.07635 Y0.0 Z5.15581

N121000 X6.0689 Y0.0 Z5.16772

N121010 X6.01966 Y0.0 Z5.2399

N121020 X6.00445 Y0.0 Z5.26218

N121030 X5.93891 Y0.0 Z5.35155

N121040 X5.90918 Y0.0 Z5.38886

N121050 X5.86887 Y0.0 Z5.43906

N121060 X5.81418 Y0.0 Z5.50286

N121070 X5.79121 Y0.0 Z5.52894

N121080 X5.72545 Y0.0 Z5.59963

N121090 X5.68869 Y0.0 Z5.63728

N121100 X5.61929 Y0.0 Z5.70294

N121110 X5.59339 Y0.0 Z5.72696

N121120 X5.52317 Y0.0 Z5.7868

N121130 X5.50344 Y0.0 Z5.80393

N121140 X5.49235 Y0.0 Z5.81261

N121150 X5.41147 Y0.0 Z5.87647

N121160 X5.34088 Y0.0 Z5.92531

N121170 X5.31971 Y0.0 Z5.94024

N121180 X5.28278 Y0.0 Z5.96292

N121190 X5.22697 Y0.0 Z5.99767

N121200 X5.20255 Y0.0 Z6.01127

N121210 X5.11731 Y0.0 Z6.05833

N121220 X5.06591 Y0.0 Z6.08398

N121230 X4.96975 Y0.0 Z6.13038

N121240 X4.92986 Y0.0 Z6.14801

N121250 X4.8246 Y0.0 Z6.19355

N121260 X4.78199 Y0.0 Z6.20951

N121270 X4.70267 Y0.0 Z6.23993

N121280 X4.63416 Y0.0 Z6.26269

N121290 X4.58936 Y0.0 Z6.2775

N121300 X4.47922 Y0.0 Z6.30955

N121310 X4.45696 Y0.0 Z6.31577

N121320 X4.42239 Y0.0 Z6.32441

N121330 X4.34251 Y0.0 Z6.3448

N121340 X4.25441 Y0.0 Z6.36484

N121350 X4.2356 Y0.0 Z6.36918

N121360 X4.21382 Y0.0 Z6.37379

N121370 X4.09622 Y0.0 Z6.39883

N121380 X4.02189 Y0.0 Z6.4127

N121390 X3.92391 Y0.0 Z6.43179

N121400 X3.80233 Y0.0 Z6.45367

N121410 X3.73555 Y0.0 Z6.46592

N121420 X3.61359 Y0.0 Z6.48725

N121430 X3.55856 Y0.0 Z6.49676

N121440 X3.48118 Y0.0 Z6.5091

N121450 X3.41837 Y0.0 Z6.51736

N121460 X3.38624 Y0.0 Z6.52066

N121470 X3.34339 Y0.0 Z6.5249

N121480 X3.27599 Y0.0 Z6.53013

N121490 X3.22436 Y0.0 Z6.53608

N121500 X3.08765 Y0.0 Z6.54955

N121510 X2.99622 Y0.0 Z6.55547

N121520 X2.95538 Y0.0 Z6.55747

N121530 X2.83445 Y0.0 Z6.55895

N121540 X2.80654 Y0.0 Z6.55879

N121550 X2.69859 Y0.0 Z6.55616

N121560 X2.67328 Y0.0 Z6.55531

N121570 X2.5888 Y0.0 Z6.54968

N121580 X2.55339 Y0.0 Z6.54719

N121590 X2.54092 Y0.0 Z6.54603

N121600 X2.44053 Y0.0 Z6.53401

N121610 X2.37246 Y0.0 Z6.5256

N121620 X2.28883 Y0.0 Z6.51464

N121630 X2.21833 Y0.0 Z6.50383

N121640 X2.13151 Y0.0 Z6.48976

N121650 X2.07588 Y0.0 Z6.47901

N121660 X1.97305 Y0.0 Z6.45821

N121670 X1.92982 Y0.0 Z6.44814

N121680 X1.82061 Y0.0 Z6.42167

N121690 X1.74841 Y0.0 Z6.40197

N121700 X1.6606 Y0.0 Z6.37794

N121710 X1.57105 Y0.0 Z6.35177

N121720 X1.5048 Y0.0 Z6.33088

N121730 X1.3936 Y0.0 Z6.29343

N121740 X1.3283 Y0.0 Z6.27

N121750 X1.24527 Y0.0 Z6.23925

N121760 X1.11206 Y0.0 Z6.18751

N121770 X1.10093 Y0.0 Z6.18292

N121780 X0.957413 Y0.0 Z6.12219

N121790 X0.877031 Y0.0 Z6.08569

N121800 X0.790492 Y0.0 Z6.04679

N121810 X0.63905 Y0.0 Z5.97486

N121820 X0.625015 Y0.0 Z5.96779

N121830 X0.515292 Y0.0 Z5.91161

N121840 X0.459259 Y0.0 Z5.88128

N121850 X0.409989 Y0.0 Z5.85372

N121860 X0.341811 Y0.0 Z5.8141

N121870 X0.311823 Y0.0 Z5.79586

N121880 X0.292158 Y0.0 Z5.78302

N121890 X0.222205 Y0.0 Z5.73671

N121900 X0.142893 Y0.0 Z5.68137

N121910 X0.130742 Y0.0 Z5.67214

N121920 X-0.129171 Y0.0 Z5.46779

N121930 X-0.180955 Y0.0 Z5.42682

N121940 X-0.230791 Y0.0 Z5.38401

N121950 X-0.316632 Y0.0 Z5.30648

N121960 X-0.392227 Y0.0 Z5.23337

N121970 X-0.434809 Y0.0 Z5.1897

N121980 X-0.490602 Y0.0 Z5.12887

N121990 X-0.535479 Y0.0 Z5.08125

N122000 X-0.627476 Y0.0 Z4.97576

N122010 X-0.683729 Y0.0 Z4.90877

N122020 X-0.712362 Y0.0 Z4.87407

N122030 X-0.773882 Y0.0 Z4.79898

N122040 X-0.793772 Y0.0 Z4.77493

N122050 X-0.811743 Y0.0 Z4.75317

N122060 X-0.876877 Y0.0 Z4.67495

N122070 X-0.932209 Y0.0 Z4.60976

N122080 X-0.955605 Y0.0 Z4.58258

N122090 X-0.987157 Y0.0 Z4.54734

N122100 X-1.02128 Y0.0 Z4.50989

N122110 X-1.04856 Y0.0 Z4.48154

N122120 X-1.07023 Y0.0 Z4.45982

N122130 X-1.09731 Y0.0 Z4.43508

N122140 X-1.11079 Y0.0 Z4.4235

N122150 X-1.13419 Y0.0 Z4.40529

N122160 X-1.15169 Y0.0 Z4.39328

N122170 X-1.1669 Y0.0 Z4.38471

N122180 X-1.17963 Y0.0 Z4.38616

N122190 X-1.19278 Y0.0 Z4.3881

N122200 X-1.2155 Y0.0 Z4.39154

N122210 X-1.24812 Y0.0 Z4.3988

N122220 X-1.26514 Y0.0 Z4.40442

N122230 X-1.29119 Y0.0 Z4.41319

N122240 X-1.30326 Y0.0 Z4.41802

N122250 X-1.3524 Y0.0 Z4.43929

N122260 X-1.39815 Y0.0 Z4.46112

N122270 X-1.43412 Y0.0 Z4.47826

N122280 X-1.45755 Y0.0 Z4.4898

N122290 X-1.50914 Y0.0 Z4.51645

N122300 X-1.54864 Y0.0 Z4.53795

N122310 X-1.58194 Y0.0 Z4.55633

N122320 X-1.65929 Y0.0 Z4.60007

N122330 X-1.71563 Y0.0 Z4.63083

N122340 X-1.777 Y0.0 Z4.6649

N122350 X-1.8834 Y0.0 Z4.71942

N122360 X-1.89673 Y0.0 Z4.7261

N122370 X-2.0006 Y0.0 Z4.77535

N122380 X-2.06706 Y0.0 Z4.80357

N122390 X-2.10803 Y0.0 Z4.82143

N122400 X-2.22004 Y0.0 Z4.86594

N122410 X-2.23533 Y0.0 Z4.8723

N122420 X-2.27577 Y0.0 Z4.88751

N122430 X-2.36129 Y0.0 Z4.91966

N122440 X-2.38399 Y0.0 Z4.92722

N122450 X-2.48383 Y0.0 Z4.96314

N122460 X-2.6919 Y0.0 Z5.03319

N122470 X-2.8799 Y0.0 Z5.09583

N122480 X-2.91413 Y0.0 Z5.10716

N122490 X-3.13442 Y0.0 Z5.18011

N122500 X-3.16667 Y0.0 Z5.19088

N122510 X-3.34014 Y0.0 Z5.24871

N122520 X-3.43212 Y0.0 Z5.28095

N122530 X-3.51414 Y0.0 Z5.30891

N122540 X-3.63193 Y0.0 Z5.35229

N122550 X-3.68252 Y0.0 Z5.3698

N122560 X-3.78379 Y0.0 Z5.40738

N122570 X-3.87096 Y0.0 Z5.43566

N122580 X-3.91955 Y0.0 Z5.45207

N122590 X-4.01541 Y0.0 Z5.47935

N122600 X-4.03478 Y0.0 Z5.48488

N122610 X-4.11915 Y0.0 Z5.50598

N122620 X-4.13439 Y0.0 Z5.50976

N122630 X-4.20844 Y0.0 Z5.52631

N122640 X-4.22721 Y0.0 Z5.5304

N122650 X-4.32683 Y0.0 Z5.54855

N122660 X-4.34466 Y0.0 Z5.55173

N122670 X-4.36181 Y0.0 Z5.55432

N122680 X-4.45981 Y0.0 Z5.56685

N122690 X-4.49222 Y0.0 Z5.57025

N122700 X-4.55329 Y0.0 Z5.57425

N122710 X-4.60737 Y0.0 Z5.57661

N122720 X-4.63736 Y0.0 Z5.57663

N122730 X-4.72355 Y0.0 Z5.57429

N122740 X-4.79914 Y0.0 Z5.56729

N122750 X-4.81261 Y0.0 Z5.56599

N122760 X-4.86032 Y0.0 Z5.55852

N122770 X-4.90723 Y0.0 Z5.55108

N122780 X-4.99564 Y0.0 Z5.53086

N122790 X-5.03578 Y0.0 Z5.51941

N122800 X-5.07302 Y0.0 Z5.50776

N122810 X-5.13015 Y0.0 Z5.48646

N122820 X-5.15524 Y0.0 Z5.47687

N122830 X-5.22159 Y0.0 Z5.44693

N122840 X-5.27055 Y0.0 Z5.42428

N122850 X-5.30994 Y0.0 Z5.40401

N122860 X-5.40028 Y0.0 Z5.35425

N122870 X-5.41688 Y0.0 Z5.34391

N122880 X-5.4841 Y0.0 Z5.30164

N122890 X-5.55731 Y0.0 Z5.25039

N122900 X-5.59641 Y0.0 Z5.22036

N122910 X-5.61851 Y0.0 Z5.20295

N122920 X-5.676 Y0.0 Z5.15187

N122930 X-5.68703 Y0.0 Z5.14186

N122940 X-5.75053 Y0.0 Z5.07761

N122950 X-5.76514 Y0.0 Z5.06293

N122960 X-5.89516 Y0.0 Z4.90977

N122970 X-5.90471 Y0.0 Z4.89765

N122980 X-5.92518 Y0.0 Z4.87002

N122990 X-5.96485 Y0.0 Z4.81528

N123000 X-5.98108 Y0.0 Z4.79103

N123010 X-6.01411 Y0.0 Z4.74012

N123020 X-6.03415 Y0.0 Z4.70585

N123030 X-6.06401 Y0.0 Z4.65375

N123040 X-6.08677 Y0.0 Z4.61083

N123050 X-6.11238 Y0.0 Z4.56099

N123060 X-6.14753 Y0.0 Z4.48644

N123070 X-6.1611 Y0.0 Z4.4567

N123080 X-6.17604 Y0.0 Z4.42163

N123090 X-6.20369 Y0.0 Z4.3519

N123100 X-6.22495 Y0.0 Z4.28942

N123110 X-6.24716 Y0.0 Z4.21964

N123120 X-6.26768 Y0.0 Z4.14952

N123130 X-6.29792 Y0.0 Z4.03271

N123140 X-6.30399 Y0.0 Z4.00998

N123150 X-6.31105 Y0.0 Z3.98368

N123160 X-6.34391 Y0.0 Z3.86052

N123170 X-6.29828 Y0.0 Z3.82844

N123180 X-6.2825 Y0.0 Z3.88727
