# Supplementary material for: B-spline curve fitting based on dynamic adjustment of knot vector using feature points
Source: PLoS One. 2025 Jun 27;20(6):e0325458. doi: 10.1371/journal.pone.0325458 (PMC12204621; doi:10.1371/journal.pone.0325458)
Supplement: Supporting information — (DOCX) [file pone.0325458.s002.docx]

%

O0001

N10 G00 X0.0 Y0.0 Z11

N20 X0.0Y6.04737

N40 G01N100 X-1.52861 Y6.03737 Z4.54921

N110 X-1.50356 Y6.03737 Z4.5513

N120 X-1.3402 Y6.03737 Z4.56213

N130 X-1.29969 Y6.03737 Z4.56364

N140 X-1.17516 Y6.03737 Z4.56997

N150 X-1.05975 Y6.03737 Z4.57469

N160 X-0.985059 Y6.03737 Z4.57661

N170 X-0.881134 Y6.03737 Z4.5799

N180 X-0.682557 Y6.03737 Z4.58383

N190 X-0.62111 Y6.03737 Z4.58491

N200 X-0.494285 Y6.03737 Z4.58575

N210 X-0.464537 Y6.03737 Z4.58602

N220 X-0.300744 Y6.03737 Z4.58487

N230 X-0.286896 Y6.03737 Z4.58506

N240 X-0.272192 Y6.03737 Z4.58498

N250 X-0.190764 Y6.03737 Z4.58206

N260 X0.481063 Y5.99631 Z4.65228

N270 X0.380131 Y5.99631 Z4.66647

N280 X0.247347 Y5.99631 Z4.68176

N290 X0.110534 Y5.99631 Z4.69444

N300 X-0.133382 Y5.99631 Z4.70897

N310 X-0.149114 Y5.99631 Z4.70944

N320 X-0.190453 Y5.99631 Z4.70886

N330 X-0.349813 Y5.99631 Z4.7098

N340 X-0.462885 Y5.99631 Z4.7082

N350 X-0.528944 Y5.99631 Z4.70676

N360 X-0.554281 Y5.99631 Z4.7065

N370 X-0.71571 Y5.99631 Z4.70498

N380 X-0.77086 Y5.99631 Z4.70492

N390 X-0.935292 Y5.99631 Z4.70231

N400 X-0.999403 Y5.99631 Z4.70073

N410 X-1.1969 Y5.99631 Z4.69563

N420 X-1.24835 Y5.99631 Z4.69602

N430 X-1.44673 Y5.99631 Z4.68811

N440 X-1.48711 Y5.99631 Z4.68639

N450 X-1.72407 Y5.99631 Z4.66825

N460 X-1.75059 Y5.99631 Z4.66603

N470 X-1.93789 Y5.99631 Z4.64458

N480 X-2.07759 Y5.99631 Z4.6269

N490 X-2.16422 Y5.99631 Z4.6147

N500 X-2.50439 Y5.95812 Z4.67758

N510 X-2.4357 Y5.95812 Z4.691

N520 X-2.36923 Y5.95812 Z4.70434

N530 X-2.32666 Y5.95812 Z4.71096

N540 X-2.27421 Y5.95812 Z4.71965

N550 X-2.2052 Y5.95812 Z4.73427

N560 X-2.07296 Y5.95812 Z4.75911

N570 X-2.06146 Y5.95812 Z4.76122

N580 X-2.03964 Y5.95812 Z4.76447

N590 X-1.88533 Y5.95812 Z4.78307

N600 X-1.70741 Y5.95812 Z4.80327

N610 X-1.53063 Y5.95812 Z4.81079

N620 X-1.37664 Y5.95812 Z4.81368

N630 X-1.16835 Y5.95812 Z4.81246

N640 X-1.14889 Y5.95812 Z4.81295

N650 X-0.979598 Y5.95812 Z4.81326

N660 X-0.94651 Y5.95812 Z4.81253

N670 X-0.783849 Y5.95812 Z4.81411

N680 X-0.759879 Y5.95812 Z4.81397

N690 X-0.605052 Y5.95812 Z4.81876

N700 X-0.577561 Y5.95812 Z4.81904

N710 X-0.404322 Y5.95812 Z4.82552

N720 X-0.252402 Y5.95812 Z4.83349

N730 X-0.188425 Y5.95812 Z4.83426

N740 X-0.054798 Y5.95812 Z4.83098

N750 X-0.00588477 Y5.95812 Z4.82826

N760 X0.25918 Y5.95812 Z4.80204

N770 X0.343762 Y5.95812 Z4.79021

N780 X0.48368 Y5.95812 Z4.7714

N790 X0.49366 Y5.95812 Z4.76993

N800 X0.566578 Y5.95812 Z4.75704

N810 X0.617557 Y5.95812 Z4.74831

N820 X0.635951 Y5.95812 Z4.74473

N830 X0.736983 Y5.95812 Z4.72415

N840 X0.807707 Y5.95812 Z4.70633

N850 X1.06602 Y5.91942 Z4.75635

N860 X0.962748 Y5.91942 Z4.78903

N870 X0.891612 Y5.91942 Z4.80969

N880 X0.797138 Y5.91942 Z4.83331

N890 X0.67486 Y5.91942 Z4.86589

N900 X0.660904 Y5.91942 Z4.86897

N910 X0.485762 Y5.91942 Z4.89469

N920 X0.456153 Y5.91942 Z4.89867

N930 X0.400969 Y5.91942 Z4.90639

N940 X0.0774992 Y5.91942 Z4.93569

N950 X0.0594297 Y5.91942 Z4.93619

N960 X0.0317716 Y5.91942 Z4.93758

N970 X-0.100755 Y5.91942 Z4.94116

N980 X-0.205593 Y5.91942 Z4.94041

N990 X-0.249718 Y5.91942 Z4.93995

N1000 X-0.363502 Y5.91942 Z4.93378

N1010 X-0.402794 Y5.91942 Z4.93076

N1020 X-0.521214 Y5.91942 Z4.91903

N1030 X-0.589759 Y5.91942 Z4.91447

N1040 X-0.740938 Y5.91942 Z4.91046

N1050 X-0.775679 Y5.91942 Z4.90939

N1060 X-0.943873 Y5.91942 Z4.91032

N1070 X-0.973929 Y5.91942 Z4.91098

N1080 X-1.08518 Y5.91942 Z4.91147

N1090 X-1.09774 Y5.91942 Z4.91197

N1100 X-1.28292 Y5.91942 Z4.9087

N1110 X-1.36322 Y5.91942 Z4.90934

N1120 X-1.4744 Y5.91942 Z4.91138

N1130 X-1.5433 Y5.91942 Z4.91314

N1140 X-1.65623 Y5.91942 Z4.91386

N1150 X-1.71953 Y5.91942 Z4.9096

N1160 X-1.86407 Y5.91942 Z4.90687

N1170 X-1.88993 Y5.91942 Z4.90458

N1180 X-2.16289 Y5.91942 Z4.87296

N1190 X-2.19165 Y5.91942 Z4.8688

N1200 X-2.3186 Y5.91942 Z4.84356

N1210 X-2.3744 Y5.91942 Z4.83216

N1220 X-2.45215 Y5.91942 Z4.81297

N1230 X-2.55662 Y5.91942 Z4.78728

N1240 X-2.57095 Y5.91942 Z4.78441

N1250 X-2.60446 Y5.91942 Z4.77786

N1260 X-2.71859 Y5.91942 Z4.75245

N1270 X-2.78743 Y5.91942 Z4.73411

N1280 X-3.03951 Y5.87888 Z4.78105

N1290 X-2.95732 Y5.87888 Z4.81098

N1300 X-2.81673 Y5.87888 Z4.85098

N1310 X-2.68666 Y5.87888 Z4.88754

N1320 X-2.62391 Y5.87888 Z4.90507

N1330 X-2.55044 Y5.87888 Z4.92113

N1340 X-2.40958 Y5.87888 Z4.95116

N1350 X-2.36062 Y5.87888 Z4.95796

N1360 X-2.19276 Y5.87888 Z4.9806

N1370 X-2.06586 Y5.87888 Z4.9909

N1380 X-1.89302 Y5.87888 Z5.00192

N1390 X-1.60982 Y5.87888 Z5.00406

N1400 X-1.54809 Y5.87888 Z5.00262

N1410 X-1.21219 Y5.87888 Z4.99742

N1420 X-1.18736 Y5.87888 Z4.99801

N1430 X-1.08465 Y5.87888 Z4.99386

N1440 X-1.04261 Y5.87888 Z4.99368

N1450 X-0.870289 Y5.87888 Z4.99219

N1460 X-0.790838 Y5.87888 Z4.99293

N1470 X-0.611398 Y5.87888 Z5.00246

N1480 X-0.438954 Y5.87888 Z5.01787

N1490 X-0.31467 Y5.87888 Z5.0296

N1500 X-0.172604 Y5.87888 Z5.03295

N1510 X-0.138882 Y5.87888 Z5.03352

N1520 X0.0495011 Y5.87888 Z5.03432

N1530 X0.302745 Y5.87888 Z5.02715

N1540 X0.54144 Y5.87888 Z5.00601

N1550 X0.726023 Y5.87888 Z4.98462

N1560 X1.03715 Y5.87888 Z4.89668

N1570 X1.18587 Y5.87888 Z4.84552

N1580 X1.28804 Y5.87888 Z4.80501

N1590 X1.49798 Y5.83702 Z4.84883

N1600 X1.48544 Y5.83702 Z4.85554

N1610 X1.47465 Y5.83702 Z4.86093

N1620 X1.40514 Y5.83702 Z4.8938

N1630 X1.30714 Y5.83702 Z4.93512

N1640 X1.27812 Y5.83702 Z4.94663

N1650 X0.985641 Y5.83702 Z5.05425

N1660 X0.921376 Y5.83702 Z5.07374

N1670 X0.79926 Y5.83702 Z5.09796

N1680 X0.617973 Y5.83702 Z5.11462

N1690 X0.582842 Y5.83702 Z5.11784

N1700 X0.388896 Y5.83702 Z5.12225

N1710 X0.204881 Y5.83702 Z5.12268

N1720 X0.187835 Y5.83702 Z5.12321

N1730 X-0.262467 Y5.83702 Z5.10913

N1740 X-0.338767 Y5.83702 Z5.10544

N1750 X-0.471134 Y5.83702 Z5.09469

N1760 X-0.56945 Y5.83702 Z5.089

N1770 X-0.6453 Y5.83702 Z5.0802

N1780 X-0.813222 Y5.83702 Z5.07206

N1790 X-0.950105 Y5.83702 Z5.06616

N1800 X-0.973436 Y5.83702 Z5.06637

N1810 X-1.08319 Y5.83702 Z5.06572

N1820 X-1.12883 Y5.83702 Z5.06802

N1830 X-1.20902 Y5.83702 Z5.06614

N1840 X-1.30355 Y5.83702 Z5.07137

N1850 X-1.35871 Y5.83702 Z5.07114

N1860 X-1.51186 Y5.83702 Z5.07779

N1870 X-1.56057 Y5.83702 Z5.07892

N1880 X-1.79585 Y5.83702 Z5.08443

N1890 X-1.83634 Y5.83702 Z5.08391

N1900 X-1.9176 Y5.83702 Z5.0838

N1910 X-2.11281 Y5.83702 Z5.08069

N1920 X-2.32635 Y5.83702 Z5.06634

N1930 X-2.37683 Y5.83702 Z5.06316

N1940 X-2.59434 Y5.83702 Z5.03308

N1950 X-2.90532 Y5.83702 Z4.96043

N1960 X-3.00901 Y5.83702 Z4.92388

N1970 X-3.03228 Y5.83702 Z4.91547

N1980 X-3.04409 Y5.83702 Z4.91003

N1990 X-3.17774 Y5.83702 Z4.86013

N2000 X-3.26552 Y5.83702 Z4.81658

N2010 X-3.47006 Y5.79459 Z4.84035

N2020 X-3.39027 Y5.79459 Z4.89083

N2030 X-3.2341 Y5.79459 Z4.96949

N2040 X-3.07197 Y5.79459 Z5.04211

N2050 X-2.96547 Y5.79459 Z5.07347

N2060 X-2.85539 Y5.79459 Z5.10069

N2070 X-2.81516 Y5.79459 Z5.10706

N2080 X-2.65443 Y5.79459 Z5.12953

N2090 X-2.59644 Y5.79459 Z5.13759

N2100 X-2.58176 Y5.79459 Z5.13962

N2110 X-2.39573 Y5.79459 Z5.15133

N2120 X-2.2791 Y5.79459 Z5.15844

N2130 X-2.26878 Y5.79459 Z5.15836

N2140 X-1.96821 Y5.79459 Z5.15848

N2150 X-1.86325 Y5.79459 Z5.15378

N2160 X-1.6407 Y5.79459 Z5.15005

N2170 X-1.56779 Y5.79459 Z5.14462

N2180 X-1.38761 Y5.79459 Z5.13997

N2190 X-1.3565 Y5.79459 Z5.13818

N2200 X-1.21723 Y5.79459 Z5.13641

N2210 X-1.20581 Y5.79459 Z5.13577

N2220 X-1.08111 Y5.79459 Z5.12964

N2230 X-1.04621 Y5.79459 Z5.12985

N2240 X-0.956258 Y5.79459 Z5.12904

N2250 X-0.898379 Y5.79459 Z5.13154

N2260 X-0.822034 Y5.79459 Z5.13433

N2270 X-0.725733 Y5.79459 Z5.14349

N2280 X-0.672197 Y5.79459 Z5.14668

N2290 X-0.534022 Y5.79459 Z5.16084

N2300 X-0.476463 Y5.79459 Z5.16417

N2310 X-0.43074 Y5.79459 Z5.16789

N2320 X-0.25072 Y5.79459 Z5.17754

N2330 X-0.132552 Y5.79459 Z5.18253

N2340 X0.0618033 Y5.79459 Z5.19294

N2350 X0.27921 Y5.79459 Z5.20522

N2360 X0.315889 Y5.79459 Z5.20637

N2370 X0.428764 Y5.79459 Z5.20946

N2380 X0.513156 Y5.79459 Z5.21444

N2390 X0.643532 Y5.79459 Z5.21036

N2400 X0.742719 Y5.79459 Z5.21139

N2410 X0.851822 Y5.79459 Z5.20544

N2420 X0.911041 Y5.79459 Z5.19978

N2430 X0.989497 Y5.79459 Z5.18241

N2440 X1.09929 Y5.79459 Z5.15624

N2450 X1.13215 Y5.79459 Z5.14456

N2460 X1.24889 Y5.79459 Z5.10225

N2470 X1.29542 Y5.79459 Z5.08422

N2480 X1.37888 Y5.79459 Z5.04733

N2490 X1.5693 Y5.79459 Z4.95527

N2500 X1.57867 Y5.79459 Z4.95026

N2510 X1.63825 Y5.79459 Z4.9171

N2520 X1.68603 Y5.79459 Z4.88696

N2530 X1.85796 Y5.75251 Z4.92265

N2540 X1.71654 Y5.75251 Z5.01603

N2550 X1.61028 Y5.75251 Z5.08152

N2560 X1.59444 Y5.75251 Z5.08978

N2570 X1.50938 Y5.75251 Z5.13208

N2580 X1.47334 Y5.75251 Z5.14924

N2590 X1.36257 Y5.75251 Z5.19509

N2600 X1.30785 Y5.75251 Z5.21522

N2610 X1.23347 Y5.75251 Z5.24138

N2620 X1.16286 Y5.75251 Z5.2592

N2630 X1.07008 Y5.75251 Z5.28264

N2640 X1.02256 Y5.75251 Z5.28815

N2650 X0.876291 Y5.75251 Z5.30306

N2660 X0.858192 Y5.75251 Z5.30321

N2670 X0.677412 Y5.75251 Z5.30133

N2680 X0.661384 Y5.75251 Z5.30028

N2690 X0.638565 Y5.75251 Z5.29827

N2700 X0.443241 Y5.75251 Z5.28668

N2710 X0.414414 Y5.75251 Z5.28344

N2720 X0.34643 Y5.75251 Z5.2777

N2730 X0.222649 Y5.75251 Z5.26764

N2740 X0.00591406 Y5.75251 Z5.25167

N2750 X-0.0257997 Y5.75251 Z5.24931

N2760 X-0.0783394 Y5.75251 Z5.24765

N2770 X-0.211388 Y5.75251 Z5.23931

N2780 X-0.274641 Y5.75251 Z5.23689

N2790 X-0.428232 Y5.75251 Z5.22701

N2800 X-0.514243 Y5.75251 Z5.21893

N2810 X-0.612506 Y5.75251 Z5.21279

N2820 X-0.693925 Y5.75251 Z5.20445

N2830 X-0.836039 Y5.75251 Z5.19599

N2840 X-0.846271 Y5.75251 Z5.19552

N2850 X-0.962183 Y5.75251 Z5.18684

N2860 X-0.973518 Y5.75251 Z5.18656

N2870 X-1.0809 Y5.75251 Z5.18341

N2880 X-1.10583 Y5.75251 Z5.18433

N2890 X-1.20189 Y5.75251 Z5.18368

N2900 X-1.26272 Y5.75251 Z5.18815

N2910 X-1.34726 Y5.75251 Z5.18923

N2920 X-1.47094 Y5.75251 Z5.19633

N2930 X-1.78335 Y5.75251 Z5.2148

N2940 X-1.86627 Y5.75251 Z5.21619

N2950 X-2.13063 Y5.75251 Z5.22803

N2960 X-2.23811 Y5.75251 Z5.22799

N2970 X-2.47464 Y5.75251 Z5.22982

N2980 X-2.60166 Y5.75251 Z5.22208

N2990 X-2.81349 Y5.75251 Z5.21069

N3000 X-2.83712 Y5.75251 Z5.20739

N3010 X-2.87242 Y5.75251 Z5.2018

N3020 X-3.00043 Y5.75251 Z5.18175

N3030 X-3.07415 Y5.75251 Z5.16763

N3040 X-3.12196 Y5.75251 Z5.15362

N3050 X-3.18648 Y5.75251 Z5.13243

N3060 X-3.22006 Y5.75251 Z5.12078

N3070 X-3.24861 Y5.75251 Z5.1085

N3080 X-3.55939 Y5.75251 Z4.92374

N3090 X-3.6011 Y5.75251 Z4.89226

N3100 X-3.63008 Y5.75251 Z4.86974

N3110 X-3.65386 Y5.75251 Z4.84938

N3120 X-3.81516 Y5.70944 Z4.85512

N3130 X-3.80647 Y5.70944 Z4.8641

N3140 X-3.7928 Y5.70944 Z4.87803

N3150 X-3.75032 Y5.70944 Z4.91805

N3160 X-3.71797 Y5.70944 Z4.94657

N3170 X-3.5734 Y5.70944 Z5.06774

N3180 X-3.545 Y5.70944 Z5.087

N3190 X-3.45524 Y5.70944 Z5.14035

N3200 X-3.42765 Y5.70944 Z5.1571

N3210 X-3.27125 Y5.70944 Z5.22659

N3220 X-3.24651 Y5.70944 Z5.23384

N3230 X-3.21808 Y5.70944 Z5.24137

N3240 X-3.14076 Y5.70944 Z5.26038

N3250 X-3.06186 Y5.70944 Z5.27657

N3260 X-3.02415 Y5.70944 Z5.28079

N3270 X-2.98443 Y5.70944 Z5.28443

N3280 X-2.87225 Y5.70944 Z5.2934

N3290 X-2.7222 Y5.70944 Z5.30123

N3300 X-2.63867 Y5.70944 Z5.30168

N3310 X-2.58952 Y5.70944 Z5.29975

N3320 X-2.29752 Y5.70944 Z5.2969

N3330 X-2.22083 Y5.70944 Z5.29043

N3340 X-1.92659 Y5.70944 Z5.27761

N3350 X-1.87041 Y5.70944 Z5.27219

N3360 X-1.66424 Y5.70944 Z5.26131

N3370 X-1.57198 Y5.70944 Z5.25444

N3380 X-1.55624 Y5.70944 Z5.25403

N3390 X-1.3378 Y5.70944 Z5.24148

N3400 X-1.3227 Y5.70944 Z5.24129

N3410 X-1.19786 Y5.70944 Z5.23211

N3420 X-1.15545 Y5.70944 Z5.2324

N3430 X-1.08236 Y5.70944 Z5.22972

N3440 X-1.02348 Y5.70944 Z5.23144

N3450 X-0.967735 Y5.70944 Z5.23279

N3460 X-0.894536 Y5.70944 Z5.23827

N3470 X-0.846687 Y5.70944 Z5.24106

N3480 X-0.741657 Y5.70944 Z5.25271

N3490 X-0.712833 Y5.70944 Z5.25457

N3500 X-0.626127 Y5.70944 Z5.2658

N3510 X-0.565299 Y5.70944 Z5.2696

N3520 X-0.460962 Y5.70944 Z5.2794

N3530 X-0.388175 Y5.70944 Z5.28432

N3540 X-0.367299 Y5.70944 Z5.28593

N3550 X-0.308051 Y5.70944 Z5.28985

N3560 X-0.177815 Y5.70944 Z5.29485

N3570 X-0.111676 Y5.70944 Z5.29899

N3580 X0.112593 Y5.70944 Z5.31531

N3590 X0.148308 Y5.70944 Z5.31974

N3600 X0.221744 Y5.70944 Z5.32614

N3610 X0.317189 Y5.70944 Z5.33782

N3620 X0.346856 Y5.70944 Z5.34135

N3630 X0.368721 Y5.70944 Z5.3446

N3640 X0.517162 Y5.70944 Z5.36114

N3650 X0.555149 Y5.70944 Z5.36622

N3660 X0.630756 Y5.70944 Z5.37408

N3670 X0.702512 Y5.70944 Z5.3804

N3680 X0.833291 Y5.70944 Z5.38895

N3690 X0.863203 Y5.70944 Z5.38926

N3700 X1.01104 Y5.70944 Z5.38804

N3710 X1.04546 Y5.70944 Z5.38453

N3720 X1.12647 Y5.70944 Z5.37515

N3730 X1.19387 Y5.70944 Z5.36736

N3740 X1.21725 Y5.70944 Z5.3633

N3750 X1.3287 Y5.70944 Z5.33483

N3760 X1.35458 Y5.70944 Z5.3281

N3770 X1.44829 Y5.70944 Z5.29649

N3780 X1.47614 Y5.70944 Z5.28624

N3790 X1.59609 Y5.70944 Z5.23275

N3800 X1.73189 Y5.70944 Z5.16342

N3810 X1.76921 Y5.70944 Z5.14055

N3820 X2.02382 Y5.70944 Z4.95483

N3830 X2.18137 Y5.66617 Z4.98203

N3840 X2.06608 Y5.66617 Z5.07886

N3850 X2.04638 Y5.66617 Z5.09358

N3860 X1.93852 Y5.66617 Z5.18518

N3870 X1.90123 Y5.66617 Z5.21159

N3880 X1.82444 Y5.66617 Z5.25922

N3890 X1.73497 Y5.66617 Z5.30648

N3900 X1.70036 Y5.66617 Z5.32237

N3910 X1.66552 Y5.66617 Z5.33705

N3920 X1.57723 Y5.66617 Z5.37371

N3930 X1.48875 Y5.66617 Z5.40341

N3940 X1.46217 Y5.66617 Z5.41238

N3950 X1.3469 Y5.66617 Z5.44234

N3960 X1.22794 Y5.66617 Z5.463

N3970 X1.20018 Y5.66617 Z5.4662

N3980 X1.09759 Y5.66617 Z5.47343

N3990 X1.02327 Y5.66617 Z5.47182

N4000 X0.930506 Y5.66617 Z5.47238

N4010 X0.811702 Y5.66617 Z5.46318

N4020 X0.781967 Y5.66617 Z5.46122

N4030 X0.712582 Y5.66617 Z5.45327

N4040 X0.631919 Y5.66617 Z5.44367

N4050 X0.58865 Y5.66617 Z5.43707

N4060 X0.472746 Y5.66617 Z5.42133

N4070 X0.415723 Y5.66617 Z5.41127

N4080 X0.292288 Y5.66617 Z5.39322

N4090 X0.282191 Y5.66617 Z5.39141

N4100 X0.192907 Y5.66617 Z5.37708

N4110 X0.13621 Y5.66617 Z5.37013

N4120 X0.106067 Y5.66617 Z5.36639

N4130 X-0.137374 Y5.66617 Z5.34584

N4140 X-0.170528 Y5.66617 Z5.34304

N4150 X-0.338071 Y5.66617 Z5.33391

N4160 X-0.455491 Y5.66617 Z5.32483

N4170 X-0.529549 Y5.66617 Z5.31983

N4180 X-0.597939 Y5.66617 Z5.31033

N4190 X-0.643432 Y5.66617 Z5.30732

N4200 X-0.728635 Y5.66617 Z5.29629

N4210 X-0.814007 Y5.66617 Z5.29077

N4220 X-0.855477 Y5.66617 Z5.28617

N4230 X-0.952282 Y5.66617 Z5.28053

N4240 X-0.973311 Y5.66617 Z5.27896

N4250 X-1.07367 Y5.66617 Z5.27653

N4260 X-1.08382 Y5.66617 Z5.27623

N4270 X-1.13876 Y5.66617 Z5.27825

N4280 X-1.2037 Y5.66617 Z5.27799

N4290 X-1.32958 Y5.66617 Z5.2801

N4300 X-1.37487 Y5.66617 Z5.28455

N4310 X-1.54015 Y5.66617 Z5.29375

N4320 X-1.59654 Y5.66617 Z5.29699

N4330 X-1.68623 Y5.66617 Z5.30748

N4340 X-1.87604 Y5.66617 Z5.3175

N4350 X-2.0666 Y5.66617 Z5.33587

N4360 X-2.22038 Y5.66617 Z5.34257

N4370 X-2.46589 Y5.66617 Z5.36329

N4380 X-2.5438 Y5.66617 Z5.36405

N4390 X-2.73519 Y5.66617 Z5.37156

N4400 X-2.83078 Y5.66617 Z5.37106

N4410 X-2.90795 Y5.66617 Z5.36962

N4420 X-3.00707 Y5.66617 Z5.36741

N4430 X-3.04272 Y5.66617 Z5.36414

N4440 X-3.06797 Y5.66617 Z5.36132

N4450 X-3.14962 Y5.66617 Z5.35004

N4460 X-3.25659 Y5.66617 Z5.33391

N4470 X-3.35549 Y5.66617 Z5.30276

N4480 X-3.41443 Y5.66617 Z5.28446

N4490 X-3.46252 Y5.66617 Z5.26158

N4500 X-3.5393 Y5.66617 Z5.2261

N4510 X-3.57412 Y5.66617 Z5.20554

N4520 X-3.64567 Y5.66617 Z5.16091

N4530 X-3.68927 Y5.66617 Z5.12935

N4540 X-3.74301 Y5.66617 Z5.08589

N4550 X-3.88708 Y5.66617 Z4.93475

N4560 X-3.93879 Y5.66617 Z4.87971

N4570 X-3.9568 Y5.66617 Z4.85891

N4580 X-4.08632 Y5.62319 Z4.86019

N4590 X-4.00056 Y5.62319 Z4.95897

N4600 X-3.90871 Y5.62319 Z5.07289

N4610 X-3.83974 Y5.62319 Z5.14023

N4620 X-3.80455 Y5.62319 Z5.16922

N4630 X-3.7387 Y5.62319 Z5.22112

N4640 X-3.68969 Y5.62319 Z5.25244

N4650 X-3.63038 Y5.62319 Z5.2883

N4660 X-3.57263 Y5.62319 Z5.31605

N4670 X-3.5137 Y5.62319 Z5.34396

N4680 X-3.46003 Y5.62319 Z5.36264

N4690 X-3.37811 Y5.62319 Z5.39063

N4700 X-3.35252 Y5.62319 Z5.39544

N4710 X-3.32051 Y5.62319 Z5.40193

N4720 X-3.24891 Y5.62319 Z5.41368

N4730 X-3.17875 Y5.62319 Z5.42579

N4740 X-3.15107 Y5.62319 Z5.42704

N4750 X-3.13389 Y5.62319 Z5.42844

N4760 X-3.05006 Y5.62319 Z5.43164

N4770 X-2.95902 Y5.62319 Z5.43696

N4780 X-2.94228 Y5.62319 Z5.43734

N4790 X-2.91628 Y5.62319 Z5.43782

N4800 X-2.7995 Y5.62319 Z5.4338

N4810 X-2.74981 Y5.62319 Z5.43155

N4820 X-2.60066 Y5.62319 Z5.42517

N4830 X-2.52836 Y5.62319 Z5.41682

N4840 X-2.26251 Y5.62319 Z5.39796

N4850 X-2.21993 Y5.62319 Z5.39437

N4860 X-2.20569 Y5.62319 Z5.39375

N4870 X-1.88164 Y5.62319 Z5.3625

N4880 X-1.8256 Y5.62319 Z5.35955

N4890 X-1.62613 Y5.62319 Z5.33622

N4900 X-1.5007 Y5.62319 Z5.329

N4910 X-1.42446 Y5.62319 Z5.32476

N4920 X-1.32177 Y5.62319 Z5.31467

N4930 X-1.24577 Y5.62319 Z5.31339

N4940 X-1.19403 Y5.62319 Z5.30924

N4950 X-1.11164 Y5.62319 Z5.3107

N4960 X-1.08465 Y5.62319 Z5.31066

N4970 X-0.996337 Y5.62319 Z5.31514

N4980 X-0.975059 Y5.62319 Z5.31639

N4990 X-0.874539 Y5.62319 Z5.32527

N5000 X-0.861329 Y5.62319 Z5.32611

N5010 X-0.784046 Y5.62319 Z5.33516

N5020 X-0.744333 Y5.62319 Z5.33773

N5030 X-0.732258 Y5.62319 Z5.33929

N5040 X-0.622581 Y5.62319 Z5.34655

N5050 X-0.542838 Y5.62319 Z5.35763

N5060 X-0.506538 Y5.62319 Z5.36034

N5070 X-0.475621 Y5.62319 Z5.3643

N5080 X-0.36116 Y5.62319 Z5.37298

N5090 X-0.263128 Y5.62319 Z5.38091

N5100 X-0.187848 Y5.62319 Z5.38387

N5110 X-0.0909594 Y5.62319 Z5.39204

N5120 X-0.0531198 Y5.62319 Z5.39493

N5130 X0.0179642 Y5.62319 Z5.40183

N5140 X0.0606115 Y5.62319 Z5.40683

N5150 X0.0986465 Y5.62319 Z5.41189

N5160 X0.156577 Y5.62319 Z5.42154

N5170 X0.170421 Y5.62319 Z5.42386

N5180 X0.203563 Y5.62319 Z5.43087

N5190 X0.252623 Y5.62319 Z5.43857

N5200 X0.306984 Y5.62319 Z5.44926

N5210 X0.356693 Y5.62319 Z5.45877

N5220 X0.392403 Y5.62319 Z5.46702

N5230 X0.492383 Y5.62319 Z5.4844

N5240 X0.553034 Y5.62319 Z5.49687

N5250 X0.657396 Y5.62319 Z5.51331

N5260 X0.690269 Y5.62319 Z5.51872

N5270 X0.729374 Y5.62319 Z5.52448

N5280 X0.835588 Y5.62319 Z5.53806

N5290 X0.948114 Y5.62319 Z5.54787

N5300 X0.995905 Y5.62319 Z5.55253

N5310 X1.02073 Y5.62319 Z5.55466

N5320 X1.14878 Y5.62319 Z5.55766

N5330 X1.16139 Y5.62319 Z5.55763

N5340 X1.18854 Y5.62319 Z5.55629

N5350 X1.26074 Y5.62319 Z5.55103

N5360 X1.33026 Y5.62319 Z5.54065

N5370 X1.36236 Y5.62319 Z5.53485

N5380 X1.43878 Y5.62319 Z5.51968

N5390 X1.48936 Y5.62319 Z5.5076

N5400 X1.55367 Y5.62319 Z5.49313

N5410 X1.64238 Y5.62319 Z5.46711

N5420 X1.67104 Y5.62319 Z5.45904

N5430 X1.72326 Y5.62319 Z5.44023

N5440 X1.78127 Y5.62319 Z5.41579

N5450 X1.83591 Y5.62319 Z5.39071

N5460 X1.88836 Y5.62319 Z5.36434

N5470 X1.93585 Y5.62319 Z5.33823

N5480 X1.98491 Y5.62319 Z5.3076

N5490 X2.07424 Y5.62319 Z5.24038

N5500 X2.09866 Y5.62319 Z5.21962

N5510 X2.19513 Y5.62319 Z5.12583

N5520 X2.23872 Y5.62319 Z5.08914

N5530 X2.32712 Y5.62319 Z5.00888

N5540 X2.47298 Y5.57886 Z5.0361

N5550 X2.4309 Y5.57886 Z5.07361

N5560 X2.34877 Y5.57886 Z5.14871

N5570 X2.32356 Y5.57886 Z5.17844

N5580 X2.26011 Y5.57886 Z5.2395

N5590 X2.21662 Y5.57886 Z5.28132

N5600 X2.10972 Y5.57886 Z5.36381

N5610 X2.08861 Y5.57886 Z5.37986

N5620 X2.00497 Y5.57886 Z5.43179

N5630 X1.97199 Y5.57886 Z5.45171

N5640 X1.96045 Y5.57886 Z5.45769

N5650 X1.8639 Y5.57886 Z5.50153

N5660 X1.77316 Y5.57886 Z5.53734

N5670 X1.75436 Y5.57886 Z5.54411

N5680 X1.70371 Y5.57886 Z5.55838

N5690 X1.64118 Y5.57886 Z5.57401

N5700 X1.62642 Y5.57886 Z5.57681

N5710 X1.5266 Y5.57886 Z5.59661

N5720 X1.48629 Y5.57886 Z5.6037

N5730 X1.41446 Y5.57886 Z5.61651

N5740 X1.35214 Y5.57886 Z5.62748

N5750 X1.32 Y5.57886 Z5.63226

N5760 X1.23202 Y5.57886 Z5.63962

N5770 X1.22184 Y5.57886 Z5.64012

N5780 X1.19206 Y5.57886 Z5.6402

N5790 X1.12265 Y5.57886 Z5.63934

N5800 X1.08142 Y5.57886 Z5.6362

N5810 X1.01218 Y5.57886 Z5.63026

N5820 X0.95323 Y5.57886 Z5.6245

N5830 X0.870104 Y5.57886 Z5.61323

N5840 X0.753566 Y5.57886 Z5.59535

N5850 X0.72109 Y5.57886 Z5.59057

N5860 X0.698261 Y5.57886 Z5.58681

N5870 X0.584922 Y5.57886 Z5.5643

N5880 X0.496077 Y5.57886 Z5.54636

N5890 X0.451172 Y5.57886 Z5.53499

N5900 X0.355057 Y5.57886 Z5.51337

N5910 X0.324664 Y5.57886 Z5.50635

N5920 X0.31036 Y5.57886 Z5.50361

N5930 X0.227742 Y5.57886 Z5.48265

N5940 X0.178669 Y5.57886 Z5.47256

N5950 X0.154045 Y5.57886 Z5.46735

N5960 X0.105365 Y5.57886 Z5.45918

N5970 X0.0905261 Y5.57886 Z5.45595

N5980 X0.0233319 Y5.57886 Z5.44463

N5990 X0.00527971 Y5.57886 Z5.44189

N6000 X-0.0636819 Y5.57886 Z5.43396

N6010 X-0.0950876 Y5.57886 Z5.4302

N6020 X-0.178773 Y5.57886 Z5.424

N6030 X-0.207744 Y5.57886 Z5.42094

N6040 X-0.263101 Y5.57886 Z5.41749

N6050 X-0.323867 Y5.57886 Z5.41209

N6060 X-0.441162 Y5.57886 Z5.4041

N6070 X-0.540496 Y5.57886 Z5.39136

N6080 X-0.636285 Y5.57886 Z5.3842

N6090 X-0.650686 Y5.57886 Z5.38239

N6100 X-0.750555 Y5.57886 Z5.36891

N6110 X-0.78536 Y5.57886 Z5.36662

N6120 X-0.861682 Y5.57886 Z5.35768

N6130 X-0.914592 Y5.57886 Z5.35433

N6140 X-0.974191 Y5.57886 Z5.34906

N6150 X-1.03241 Y5.57886 Z5.34564

N6160 X-1.08534 Y5.57886 Z5.34296

N6170 X-1.14756 Y5.57886 Z5.34304

N6180 X-1.19348 Y5.57886 Z5.34222

N6190 X-1.28917 Y5.57886 Z5.34991

N6200 X-1.3137 Y5.57886 Z5.35032

N6210 X-1.42566 Y5.57886 Z5.36132

N6220 X-1.4729 Y5.57886 Z5.36429

N6230 X-1.51038 Y5.57886 Z5.36826

N6240 X-1.65664 Y5.57886 Z5.37668

N6250 X-1.78488 Y5.57886 Z5.39168

N6260 X-1.88787 Y5.57886 Z5.39964

N6270 X-1.94553 Y5.57886 Z5.40793

N6280 X-2.18732 Y5.57886 Z5.429

N6290 X-2.26288 Y5.57886 Z5.43816

N6300 X-2.39109 Y5.57886 Z5.45476

N6310 X-2.5303 Y5.57886 Z5.46463

N6320 X-2.67805 Y5.57886 Z5.48169

N6330 X-2.70567 Y5.57886 Z5.48419

N6340 X-2.7562 Y5.57886 Z5.48825

N6350 X-2.83961 Y5.57886 Z5.49203

N6360 X-2.8699 Y5.57886 Z5.49307

N6370 X-2.95117 Y5.57886 Z5.49305

N6380 X-3.04079 Y5.57886 Z5.49563

N6390 X-3.05294 Y5.57886 Z5.49523

N6400 X-3.07299 Y5.57886 Z5.49593

N6410 X-3.14529 Y5.57886 Z5.49003

N6420 X-3.19839 Y5.57886 Z5.48763

N6430 X-3.2437 Y5.57886 Z5.48311

N6440 X-3.30213 Y5.57886 Z5.48013

N6450 X-3.34407 Y5.57886 Z5.47162

N6460 X-3.40502 Y5.57886 Z5.46016

N6470 X-3.45106 Y5.57886 Z5.44936

N6480 X-3.47122 Y5.57886 Z5.44436

N6490 X-3.5641 Y5.57886 Z5.41068

N6500 X-3.59328 Y5.57886 Z5.40034

N6510 X-3.68388 Y5.57886 Z5.35728

N6520 X-3.71204 Y5.57886 Z5.34379

N6530 X-3.80665 Y5.57886 Z5.28517

N6540 X-3.82584 Y5.57886 Z5.27304

N6550 X-3.92227 Y5.57886 Z5.19613

N6560 X-3.93068 Y5.57886 Z5.18954

N6570 X-4.03009 Y5.57886 Z5.08844

N6580 X-4.03635 Y5.57886 Z5.0798

N6590 X-4.10327 Y5.57886 Z4.98416

N6600 X-4.20536 Y5.57886 Z4.86046

N6610 X-4.3178 Y5.53244 Z4.8615

N6620 X-4.25013 Y5.53244 Z4.94684

N6630 X-4.11724 Y5.53244 Z5.13079

N6640 X-4.03575 Y5.53244 Z5.21003

N6650 X-4.0146 Y5.53244 Z5.23157

N6660 X-3.92479 Y5.53244 Z5.30188

N6670 X-3.90346 Y5.53244 Z5.31889

N6680 X-3.79836 Y5.53244 Z5.38532

N6690 X-3.78537 Y5.53244 Z5.39337

N6700 X-3.67044 Y5.53244 Z5.44837

N6710 X-3.66064 Y5.53244 Z5.45303

N6720 X-3.54847 Y5.53244 Z5.49276

N6730 X-3.53476 Y5.53244 Z5.49773

N6740 X-3.43778 Y5.53244 Z5.52179

N6750 X-3.4058 Y5.53244 Z5.5293

N6760 X-3.34187 Y5.53244 Z5.53342

N6770 X-3.28051 Y5.53244 Z5.54175

N6780 X-3.23823 Y5.53244 Z5.54391

N6790 X-3.18545 Y5.53244 Z5.54918

N6800 X-3.13819 Y5.53244 Z5.54767

N6810 X-3.10702 Y5.53244 Z5.54854

N6820 X-3.04301 Y5.53244 Z5.54629

N6830 X-2.96063 Y5.53244 Z5.549

N6840 X-2.85051 Y5.53244 Z5.54132

N6850 X-2.78327 Y5.53244 Z5.53518

N6860 X-2.71593 Y5.53244 Z5.52976

N6870 X-2.58799 Y5.53244 Z5.51819

N6880 X-2.53593 Y5.53244 Z5.51097

N6890 X-2.48966 Y5.53244 Z5.50579

N6900 X-2.31283 Y5.53244 Z5.4829

N6910 X-2.14949 Y5.53244 Z5.4631

N6920 X-2.05404 Y5.53244 Z5.45478

N6930 X-1.89473 Y5.53244 Z5.43188

N6940 X-1.71921 Y5.53244 Z5.4183

N6950 X-1.68245 Y5.53244 Z5.41281

N6960 X-1.65531 Y5.53244 Z5.41118

N6970 X-1.50965 Y5.53244 Z5.39575

N6980 X-1.45542 Y5.53244 Z5.38895

N6990 X-1.33474 Y5.53244 Z5.3828

N7000 X-1.30979 Y5.53244 Z5.38008

N7010 X-1.22461 Y5.53244 Z5.3793

N7020 X-1.19291 Y5.53244 Z5.37676

N7030 X-1.07017 Y5.53244 Z5.37758

N7040 X-0.973282 Y5.53244 Z5.38327

N7050 X-0.956525 Y5.53244 Z5.38475

N7060 X-0.862052 Y5.53244 Z5.39072

N7070 X-0.833268 Y5.53244 Z5.3941

N7080 X-0.75508 Y5.53244 Z5.39925

N7090 X-0.704689 Y5.53244 Z5.40605

N7100 X-0.65572 Y5.53244 Z5.40971

N7110 X-0.581642 Y5.53244 Z5.42064

N7120 X-0.569913 Y5.53244 Z5.42166

N7130 X-0.553191 Y5.53244 Z5.42429

N7140 X-0.483827 Y5.53244 Z5.43019

N7150 X-0.434839 Y5.53244 Z5.43484

N7160 X-0.367524 Y5.53244 Z5.44138

N7170 X-0.32984 Y5.53244 Z5.44464

N7180 X-0.269798 Y5.53244 Z5.45154

N7190 X-0.230905 Y5.53244 Z5.45397

N7200 X-0.138362 Y5.53244 Z5.46374

N7210 X-0.0418363 Y5.53244 Z5.47498

N7220 X0.0215946 Y5.53244 Z5.48461

N7230 X0.0344603 Y5.53244 Z5.48676

N7240 X0.0812479 Y5.53244 Z5.49537

N7250 X0.108117 Y5.53244 Z5.50122

N7260 X0.139671 Y5.53244 Z5.50803

N7270 X0.198111 Y5.53244 Z5.52342

N7280 X0.225719 Y5.53244 Z5.53145

N7290 X0.299989 Y5.53244 Z5.54976

N7300 X0.408691 Y5.53244 Z5.58093

N7310 X0.437172 Y5.53244 Z5.58734

N7320 X0.585051 Y5.53244 Z5.62499

N7330 X0.635772 Y5.53244 Z5.63761

N7340 X0.738022 Y5.53244 Z5.65822

N7350 X0.884904 Y5.53244 Z5.6867

N7360 X0.899938 Y5.53244 Z5.68948

N7370 X1.03551 Y5.53244 Z5.70739

N7380 X1.14869 Y5.53244 Z5.71888

N7390 X1.18472 Y5.53244 Z5.72018

N7400 X1.27082 Y5.53244 Z5.7208

N7410 X1.29448 Y5.53244 Z5.71901

N7420 X1.3731 Y5.53244 Z5.71189

N7430 X1.46826 Y5.53244 Z5.69781

N7440 X1.48141 Y5.53244 Z5.69539

N7450 X1.54154 Y5.53244 Z5.68589

N7460 X1.61087 Y5.53244 Z5.67371

N7470 X1.62137 Y5.53244 Z5.67162

N7480 X1.72553 Y5.53244 Z5.65184

N7490 X1.75088 Y5.53244 Z5.64551

N7500 X1.83883 Y5.53244 Z5.62379

N7510 X1.87327 Y5.53244 Z5.61122

N7520 X1.93891 Y5.53244 Z5.5848

N7530 X2.02974 Y5.53244 Z5.54049

N7540 X2.0716 Y5.53244 Z5.51881

N7550 X2.09851 Y5.53244 Z5.50255

N7560 X2.22219 Y5.53244 Z5.42599

N7570 X2.25921 Y5.53244 Z5.39761

N7580 X2.34692 Y5.53244 Z5.32864

N7590 X2.40703 Y5.53244 Z5.27462

N7600 X2.43856 Y5.53244 Z5.24365

N7610 X2.50424 Y5.53244 Z5.16989

N7620 X2.57098 Y5.53244 Z5.11006

N7630 X2.62236 Y5.53244 Z5.06425

N7640 X2.77302 Y5.48178 Z5.07116

N7650 X2.64842 Y5.48178 Z5.1976

N7660 X2.57398 Y5.48178 Z5.28811

N7670 X2.47272 Y5.48178 Z5.38363

N7680 X2.41699 Y5.48178 Z5.43094

N7690 X2.37235 Y5.48178 Z5.46561

N7700 X2.28496 Y5.48178 Z5.5212

N7710 X2.21727 Y5.48178 Z5.56298

N7720 X2.12058 Y5.48178 Z5.61579

N7730 X2.04964 Y5.48178 Z5.65065

N7740 X1.9941 Y5.48178 Z5.67388

N7750 X1.91575 Y5.48178 Z5.70031

N7760 X1.87502 Y5.48178 Z5.71434

N7770 X1.85477 Y5.48178 Z5.71945

N7780 X1.76133 Y5.48178 Z5.73805

N7790 X1.71054 Y5.48178 Z5.74709

N7800 X1.62213 Y5.48178 Z5.76136

N7810 X1.58028 Y5.48178 Z5.76803

N7820 X1.4976 Y5.48178 Z5.78016

N7830 X1.45265 Y5.48178 Z5.78843

N7840 X1.40445 Y5.48178 Z5.7947

N7850 X1.34891 Y5.48178 Z5.79971

N7860 X1.30918 Y5.48178 Z5.8025

N7870 X1.20273 Y5.48178 Z5.80208

N7880 X1.17764 Y5.48178 Z5.79972

N7890 X1.07248 Y5.48178 Z5.78872

N7900 X1.05432 Y5.48178 Z5.78607

N7910 X1.00233 Y5.48178 Z5.77785

N7920 X0.911895 Y5.48178 Z5.76288

N7930 X0.832786 Y5.48178 Z5.74704

N7940 X0.761601 Y5.48178 Z5.73045

N7950 X0.737489 Y5.48178 Z5.72402

N7960 X0.607578 Y5.48178 Z5.69219

N7970 X0.582784 Y5.48178 Z5.68598

N7980 X0.457005 Y5.48178 Z5.64889

N7990 X0.391719 Y5.48178 Z5.62801

N8000 X0.31887 Y5.48178 Z5.60759

N8010 X0.274119 Y5.48178 Z5.59234

N8020 X0.222923 Y5.48178 Z5.57696

N8030 X0.18908 Y5.48178 Z5.56659

N8040 X0.118816 Y5.48178 Z5.54721

N8050 X0.0709039 Y5.48178 Z5.53467

N8060 X0.050785 Y5.48178 Z5.53003

N8070 X-0.0460724 Y5.48178 Z5.51088

N8080 X-0.0872154 Y5.48178 Z5.50397

N8090 X-0.121944 Y5.48178 Z5.49977

N8100 X-0.171313 Y5.48178 Z5.49298

N8110 X-0.216506 Y5.48178 Z5.48891

N8120 X-0.256525 Y5.48178 Z5.48394

N8130 X-0.321116 Y5.48178 Z5.47791

N8140 X-0.340805 Y5.48178 Z5.47565

N8150 X-0.380282 Y5.48178 Z5.47223

N8160 X-0.430174 Y5.48178 Z5.46623

N8170 X-0.443385 Y5.48178 Z5.46478

N8180 X-0.514846 Y5.48178 Z5.45726

N8190 X-0.536097 Y5.48178 Z5.45504

N8200 X-0.582335 Y5.48178 Z5.44774

N8210 X-0.644507 Y5.48178 Z5.44233

N8220 X-0.665989 Y5.48178 Z5.43916

N8230 X-0.747082 Y5.48178 Z5.43311

N8240 X-0.762094 Y5.48178 Z5.43143

N8250 X-0.858824 Y5.48178 Z5.42128

N8260 X-0.8769 Y5.48178 Z5.42012

N8270 X-0.966562 Y5.48178 Z5.4125

N8280 X-0.992788 Y5.48178 Z5.4109

N8290 X-1.08066 Y5.48178 Z5.4061

N8300 X-1.10545 Y5.48178 Z5.40462

N8310 X-1.1924 Y5.48178 Z5.40235

N8320 X-1.22916 Y5.48178 Z5.40493

N8330 X-1.30966 Y5.48178 Z5.40567

N8340 X-1.3846 Y5.48178 Z5.41384

N8350 X-1.44461 Y5.48178 Z5.4169

N8360 X-1.54977 Y5.48178 Z5.43009

N8370 X-1.59443 Y5.48178 Z5.43482

N8380 X-1.69966 Y5.48178 Z5.44114

N8390 X-1.8422 Y5.48178 Z5.46242

N8400 X-1.90222 Y5.48178 Z5.46706

N8410 X-2.02936 Y5.48178 Z5.48534

N8420 X-2.12102 Y5.48178 Z5.49582

N8430 X-2.15183 Y5.48178 Z5.50125

N8440 X-2.34767 Y5.48178 Z5.52732

N8450 X-2.36036 Y5.48178 Z5.52899

N8460 X-2.38825 Y5.48178 Z5.53347

N8470 X-2.55151 Y5.48178 Z5.55175

N8480 X-2.68149 Y5.48178 Z5.56978

N8490 X-2.73055 Y5.48178 Z5.57621

N8500 X-2.84446 Y5.48178 Z5.58907

N8510 X-2.85767 Y5.48178 Z5.59051

N8520 X-2.93258 Y5.48178 Z5.59099

N8530 X-3.01623 Y5.48178 Z5.59842

N8540 X-3.05176 Y5.48178 Z5.60061

N8550 X-3.12895 Y5.48178 Z5.59846

N8560 X-3.18638 Y5.48178 Z5.6003

N8570 X-3.23492 Y5.48178 Z5.59824

N8580 X-3.2875 Y5.48178 Z5.59839

N8590 X-3.34319 Y5.48178 Z5.59083

N8600 X-3.38431 Y5.48178 Z5.58817

N8610 X-3.45161 Y5.48178 Z5.57862

N8620 X-3.51491 Y5.48178 Z5.56172

N8630 X-3.58448 Y5.48178 Z5.54741

N8640 X-3.64264 Y5.48178 Z5.52638

N8650 X-3.7205 Y5.48178 Z5.50073

N8660 X-3.77551 Y5.48178 Z5.47468

N8670 X-3.85191 Y5.48178 Z5.43926

N8680 X-3.91157 Y5.48178 Z5.4026

N8690 X-3.97875 Y5.48178 Z5.36175

N8700 X-4.04148 Y5.48178 Z5.31257

N8710 X-4.11622 Y5.48178 Z5.25665

N8720 X-4.14803 Y5.48178 Z5.22479

N8730 X-4.20075 Y5.48178 Z5.16752

N8740 X-4.23326 Y5.48178 Z5.13176

N8750 X-4.29844 Y5.48178 Z5.04027

N8760 X-4.33126 Y5.48178 Z4.98871

N8770 X-4.43137 Y5.48178 Z4.85984

N8780 X-4.54069 Y5.43081 Z4.85215

N8790 X-4.30952 Y5.43081 Z5.16973

N8800 X-4.27216 Y5.43081 Z5.21354

N8810 X-4.23813 Y5.43081 Z5.25098

N8820 X-4.17199 Y5.43081 Z5.30982

N8830 X-4.05581 Y5.43081 Z5.3986

N8840 X-4.04271 Y5.43081 Z5.40628

N8850 X-3.91008 Y5.43081 Z5.48396

N8860 X-3.88731 Y5.43081 Z5.49368

N8870 X-3.7746 Y5.43081 Z5.5453

N8880 X-3.74606 Y5.43081 Z5.55411

N8890 X-3.63034 Y5.43081 Z5.59339

N8900 X-3.61032 Y5.43081 Z5.59716

N8910 X-3.49639 Y5.43081 Z5.62339

N8920 X-3.478 Y5.43081 Z5.62717

N8930 X-3.46543 Y5.43081 Z5.62924

N8940 X-3.30746 Y5.43081 Z5.64322

N8950 X-3.2338 Y5.43081 Z5.64302

N8960 X-3.15078 Y5.43081 Z5.64652

N8970 X-3.11971 Y5.43081 Z5.64469

N8980 X-3.09507 Y5.43081 Z5.64395

N8990 X-3.01148 Y5.43081 Z5.63684

N9000 X-2.91582 Y5.43081 Z5.63426

N9010 X-2.90397 Y5.43081 Z5.63367

N9020 X-2.78011 Y5.43081 Z5.62016

N9030 X-2.75148 Y5.43081 Z5.61693

N9040 X-2.58135 Y5.43081 Z5.5946

N9050 X-2.56804 Y5.43081 Z5.59268

N9060 X-2.37215 Y5.43081 Z5.56123

N9070 X-2.28129 Y5.43081 Z5.54926

N9080 X-2.21938 Y5.43081 Z5.54102

N9090 X-2.11156 Y5.43081 Z5.52203

N9100 X-1.95944 Y5.43081 Z5.50464

N9110 X-1.91041 Y5.43081 Z5.49616

N9120 X-1.85005 Y5.43081 Z5.4895

N9130 X-1.71697 Y5.43081 Z5.46963

N9140 X-1.62746 Y5.43081 Z5.46426

N9150 X-1.57036 Y5.43081 Z5.45641

N9160 X-1.43499 Y5.43081 Z5.44496

N9170 X-1.30953 Y5.43081 Z5.43141

N9180 X-1.27412 Y5.43081 Z5.43109

N9190 X-1.1919 Y5.43081 Z5.4253

N9200 X-1.13707 Y5.43081 Z5.42673

N9210 X-1.07118 Y5.43081 Z5.43066

N9220 X-1.02393 Y5.43081 Z5.43324

N9230 X-0.956583 Y5.43081 Z5.43735

N9240 X-0.909941 Y5.43081 Z5.44131

N9250 X-0.851027 Y5.43081 Z5.44511

N9260 X-0.798234 Y5.43081 Z5.45064

N9270 X-0.754892 Y5.43081 Z5.45382

N9280 X-0.69321 Y5.43081 Z5.46127

N9290 X-0.668957 Y5.43081 Z5.46341

N9300 X-0.596797 Y5.43081 Z5.47319

N9310 X-0.578514 Y5.43081 Z5.47551

N9320 X-0.515873 Y5.43081 Z5.48204

N9330 X-0.431879 Y5.43081 Z5.49118

N9340 X-0.398063 Y5.43081 Z5.49525

N9350 X-0.345589 Y5.43081 Z5.50107

N9360 X-0.289271 Y5.43081 Z5.50911

N9370 X-0.27518 Y5.43081 Z5.51101

N9380 X-0.211863 Y5.43081 Z5.51805

N9390 X-0.19195 Y5.43081 Z5.52124

N9400 X-0.1312 Y5.43081 Z5.52938

N9410 X-0.112569 Y5.43081 Z5.53273

N9420 X-0.060882 Y5.43081 Z5.54174

N9430 X-0.0475258 Y5.43081 Z5.54459

N9440 X0.00833884 Y5.43081 Z5.55716

N9450 X0.0602831 Y5.43081 Z5.57049

N9460 X0.111778 Y5.43081 Z5.5842

N9470 X0.130791 Y5.43081 Z5.59007

N9480 X0.172192 Y5.43081 Z5.60301

N9490 X0.24172 Y5.43081 Z5.62761

N9500 X0.254206 Y5.43081 Z5.63217

N9510 X0.346458 Y5.43081 Z5.66268

N9520 X0.3758 Y5.43081 Z5.6736

N9530 X0.468061 Y5.43081 Z5.70421

N9540 X0.484654 Y5.43081 Z5.7102

N9550 X0.515503 Y5.43081 Z5.72058

N9560 X0.589078 Y5.43081 Z5.74253

N9570 X0.684284 Y5.43081 Z5.77118

N9580 X0.700437 Y5.43081 Z5.77514

N9590 X0.802067 Y5.43081 Z5.80224

N9600 X0.841611 Y5.43081 Z5.81146

N9610 X0.918382 Y5.43081 Z5.82914

N9620 X1.00938 Y5.43081 Z5.84886

N9630 X1.03543 Y5.43081 Z5.85298

N9640 X1.14036 Y5.43081 Z5.86824

N9650 X1.15189 Y5.43081 Z5.86945

N9660 X1.24501 Y5.43081 Z5.87539

N9670 X1.27875 Y5.43081 Z5.87616

N9680 X1.34181 Y5.43081 Z5.87395

N9690 X1.40039 Y5.43081 Z5.87159

N9700 X1.43023 Y5.43081 Z5.86908

N9710 X1.49498 Y5.43081 Z5.85886

N9720 X1.53916 Y5.43081 Z5.85418

N9730 X1.62161 Y5.43081 Z5.84206

N9740 X1.6697 Y5.43081 Z5.83807

N9750 X1.7674 Y5.43081 Z5.82326

N9760 X1.80102 Y5.43081 Z5.81811

N9770 X1.85473 Y5.43081 Z5.80776

N9780 X1.91942 Y5.43081 Z5.79389

N9790 X1.99119 Y5.43081 Z5.7731

N9800 X2.0319 Y5.43081 Z5.7609

N9810 X2.06604 Y5.43081 Z5.74894

N9820 X2.14005 Y5.43081 Z5.71809

N9830 X2.20496 Y5.43081 Z5.68799

N9840 X2.28735 Y5.43081 Z5.64347

N9850 X2.34225 Y5.43081 Z5.61344

N9860 X2.39798 Y5.43081 Z5.57922

N9870 X2.47531 Y5.43081 Z5.52905

N9880 X2.57768 Y5.43081 Z5.44875

N9890 X2.58602 Y5.43081 Z5.44212

N9900 X2.68184 Y5.43081 Z5.34821

N9910 X2.73754 Y5.43081 Z5.28492

N9920 X2.76986 Y5.43081 Z5.2487

N9930 X2.79169 Y5.43081 Z5.21996

N9940 X2.86592 Y5.43081 Z5.13843

N9950 X2.91649 Y5.43081 Z5.07796

N9960 X3.06635 Y5.37623 Z5.08554

N9970 X3.05675 Y5.37623 Z5.09791

N9980 X2.94288 Y5.37623 Z5.23539

N9990 X2.91248 Y5.37623 Z5.27637

N10000 X2.87379 Y5.37623 Z5.3273

N10010 X2.85849 Y5.37623 Z5.34444

N10020 X2.79479 Y5.37623 Z5.41059

N10030 X2.75242 Y5.37623 Z5.44948

N10040 X2.68358 Y5.37623 Z5.51238

N10050 X2.61082 Y5.37623 Z5.57028

N10060 X2.55849 Y5.37623 Z5.60854

N10070 X2.45007 Y5.37623 Z5.67645

N10080 X2.43535 Y5.37623 Z5.6855

N10090 X2.42341 Y5.37623 Z5.69203

N10100 X2.31133 Y5.37623 Z5.74864

N10110 X2.24556 Y5.37623 Z5.77657

N10120 X2.18956 Y5.37623 Z5.80182

N10130 X2.09939 Y5.37623 Z5.8352

N10140 X2.08251 Y5.37623 Z5.84094

N10150 X2.07163 Y5.37623 Z5.84415

N10160 X1.97852 Y5.37623 Z5.86929

N10170 X1.89254 Y5.37623 Z5.88894

N10180 X1.87915 Y5.37623 Z5.89152

N10190 X1.82468 Y5.37623 Z5.89987

N10200 X1.75339 Y5.37623 Z5.90926

N10210 X1.70304 Y5.37623 Z5.91439

N10220 X1.61922 Y5.37623 Z5.92135

N10230 X1.60674 Y5.37623 Z5.92318

N10240 X1.51577 Y5.37623 Z5.93281

N10250 X1.47362 Y5.37623 Z5.93597

N10260 X1.37393 Y5.37623 Z5.94201

N10270 X1.35095 Y5.37623 Z5.94338

N10280 X1.2812 Y5.37623 Z5.9429

N10290 X1.22522 Y5.37623 Z5.94138

N10300 X1.18293 Y5.37623 Z5.93779

N10310 X1.11456 Y5.37623 Z5.93014

N10320 X1.0128 Y5.37623 Z5.91294

N10330 X0.947493 Y5.37623 Z5.9002

N10340 X0.915525 Y5.37623 Z5.89177

N10350 X0.894456 Y5.37623 Z5.88614

N10360 X0.817062 Y5.37623 Z5.86452

N10370 X0.768634 Y5.37623 Z5.85061

N10380 X0.722754 Y5.37623 Z5.83785

N10390 X0.669427 Y5.37623 Z5.8216

N10400 X0.624638 Y5.37623 Z5.80874

N10410 X0.583159 Y5.37623 Z5.79504

N10420 X0.515715 Y5.37623 Z5.77278

N10430 X0.50241 Y5.37623 Z5.7683

N10440 X0.458277 Y5.37623 Z5.75239

N10450 X0.407676 Y5.37623 Z5.73319

N10460 X0.312847 Y5.37623 Z5.69781

N10470 X0.228325 Y5.37623 Z5.66541

N10480 X0.195296 Y5.37623 Z5.65394

N10490 X0.155843 Y5.37623 Z5.63827

N10500 X0.135856 Y5.37623 Z5.63111

N10510 X0.0977737 Y5.37623 Z5.61769

N10520 X0.0681636 Y5.37623 Z5.60825

N10530 X0.0113523 Y5.37623 Z5.59193

N10540 X-0.0526872 Y5.37623 Z5.57607

N10550 X-0.109806 Y5.37623 Z5.56398

N10560 X-0.179245 Y5.37623 Z5.55159

N10570 X-0.264571 Y5.37623 Z5.53831

N10580 X-0.321966 Y5.37623 Z5.53057

N10590 X-0.352849 Y5.37623 Z5.526

N10600 X-0.430616 Y5.37623 Z5.51475

N10610 X-0.459781 Y5.37623 Z5.51093

N10620 X-0.51302 Y5.37623 Z5.50413

N10630 X-0.550686 Y5.37623 Z5.49953

N10640 X-0.590362 Y5.37623 Z5.49434

N10650 X-0.640323 Y5.37623 Z5.48901

N10660 X-0.666748 Y5.37623 Z5.48543

N10670 X-0.734723 Y5.37623 Z5.47944

N10680 X-0.750076 Y5.37623 Z5.47758

N10690 X-0.842676 Y5.37623 Z5.47062

N10700 X-0.945897 Y5.37623 Z5.46397

N10710 X-1.06104 Y5.37623 Z5.45696

N10720 X-1.17094 Y5.37623 Z5.45041

N10730 X-1.19137 Y5.37623 Z5.44988

N10740 X-1.27275 Y5.37623 Z5.45561

N10750 X-1.31056 Y5.37623 Z5.45557

N10760 X-1.32475 Y5.37623 Z5.45711

N10770 X-1.43609 Y5.37623 Z5.46266

N10780 X-1.5038 Y5.37623 Z5.47161

N10790 X-1.57579 Y5.37623 Z5.47769

N10800 X-1.72568 Y5.37623 Z5.49829

N10810 X-1.74327 Y5.37623 Z5.50063

N10820 X-1.91993 Y5.37623 Z5.52013

N10830 X-2.07758 Y5.37623 Z5.54737

N10840 X-2.10142 Y5.37623 Z5.5501

N10850 X-2.13677 Y5.37623 Z5.55632

N10860 X-2.24891 Y5.37623 Z5.57269

N10870 X-2.36597 Y5.37623 Z5.59328

N10880 X-2.38478 Y5.37623 Z5.59576

N10890 X-2.4019 Y5.37623 Z5.59851

N10900 X-2.52943 Y5.37623 Z5.61708

N10910 X-2.61768 Y5.37623 Z5.63288

N10920 X-2.70001 Y5.37623 Z5.6448

N10930 X-2.78276 Y5.37623 Z5.65511

N10940 X-2.86751 Y5.37623 Z5.66321

N10950 X-2.99346 Y5.37623 Z5.67778

N10960 X-3.10998 Y5.37623 Z5.68125

N10970 X-3.20866 Y5.37623 Z5.68708

N10980 X-3.23157 Y5.37623 Z5.68738

N10990 X-3.26099 Y5.37623 Z5.68748

N11000 X-3.36027 Y5.37623 Z5.68069

N11010 X-3.42138 Y5.37623 Z5.67486

N11020 X-3.5153 Y5.37623 Z5.66713

N11030 X-3.5662 Y5.37623 Z5.65541

N11040 X-3.67799 Y5.37623 Z5.63439

N11050 X-3.70618 Y5.37623 Z5.62482

N11060 X-3.82353 Y5.37623 Z5.58857

N11070 X-3.84715 Y5.37623 Z5.57775

N11080 X-3.95513 Y5.37623 Z5.53165

N11090 X-3.99318 Y5.37623 Z5.50936

N11100 X-4.07647 Y5.37623 Z5.46449

N11110 X-4.19053 Y5.37623 Z5.39415

N11120 X-4.20365 Y5.37623 Z5.3852

N11130 X-4.30623 Y5.37623 Z5.29365

N11140 X-4.40061 Y5.37623 Z5.18981

N11150 X-4.41559 Y5.37623 Z5.16982

N11160 X-4.45053 Y5.37623 Z5.12054

N11170 X-4.50997 Y5.37623 Z5.03497

N11180 X-4.59292 Y5.37623 Z4.92144

N11190 X-4.6523 Y5.37623 Z4.83861

N11200 X-4.75718 Y5.32165 Z4.82303

N11210 X-4.69832 Y5.32165 Z4.91218

N11220 X-4.66743 Y5.32165 Z4.95593

N11230 X-4.52791 Y5.32165 Z5.15125

N11240 X-4.49291 Y5.32165 Z5.20164

N11250 X-4.43365 Y5.32165 Z5.26523

N11260 X-4.34613 Y5.32165 Z5.35975

N11270 X-4.33423 Y5.32165 Z5.36939

N11280 X-4.31901 Y5.32165 Z5.38053

N11290 X-4.21765 Y5.32165 Z5.45536

N11300 X-4.11489 Y5.32165 Z5.51931

N11310 X-4.10412 Y5.32165 Z5.52469

N11320 X-3.98651 Y5.32165 Z5.57872

N11330 X-3.94564 Y5.32165 Z5.59402

N11340 X-3.85989 Y5.32165 Z5.62945

N11350 X-3.80386 Y5.32165 Z5.64554

N11360 X-3.72099 Y5.32165 Z5.67149

N11370 X-3.66403 Y5.32165 Z5.68177

N11380 X-3.55873 Y5.32165 Z5.70318

N11390 X-3.5245 Y5.32165 Z5.70562

N11400 X-3.37654 Y5.32165 Z5.71973

N11410 X-3.35511 Y5.32165 Z5.71998

N11420 X-3.22586 Y5.32165 Z5.71951

N11430 X-3.10194 Y5.32165 Z5.71791

N11440 X-2.97567 Y5.32165 Z5.70462

N11450 X-2.83297 Y5.32165 Z5.69245

N11460 X-2.8181 Y5.32165 Z5.69082

N11470 X-2.79281 Y5.32165 Z5.68767

N11480 X-2.6526 Y5.32165 Z5.66756

N11490 X-2.62532 Y5.32165 Z5.66346

N11500 X-2.49454 Y5.32165 Z5.64203

N11510 X-2.3742 Y5.32165 Z5.61737

N11520 X-2.29206 Y5.32165 Z5.60417

N11530 X-2.24593 Y5.32165 Z5.59606

N11540 X-2.19255 Y5.32165 Z5.58827

N11550 X-2.10176 Y5.32165 Z5.57066

N11560 X-1.99091 Y5.32165 Z5.55471

N11570 X-1.92946 Y5.32165 Z5.54409

N11580 X-1.87634 Y5.32165 Z5.53823

N11590 X-1.74564 Y5.32165 Z5.51581

N11600 X-1.59918 Y5.32165 Z5.50145

N11610 X-1.58123 Y5.32165 Z5.49898

N11620 X-1.43822 Y5.32165 Z5.48049

N11630 X-1.38223 Y5.32165 Z5.4777

N11640 X-1.31482 Y5.32165 Z5.47038

N11650 X-1.2135 Y5.32165 Z5.47047

N11660 X-1.1956 Y5.32165 Z5.46922

N11670 X-1.17871 Y5.32165 Z5.47042

N11680 X-1.0816 Y5.32165 Z5.47188

N11690 X-1.04166 Y5.32165 Z5.47393

N11700 X-0.971453 Y5.32165 Z5.47771

N11710 X-0.929426 Y5.32165 Z5.48015

N11720 X-0.86592 Y5.32165 Z5.48492

N11730 X-0.830073 Y5.32165 Z5.48741

N11740 X-0.767976 Y5.32165 Z5.49322

N11750 X-0.741434 Y5.32165 Z5.4955

N11760 X-0.67829 Y5.32165 Z5.50257

N11770 X-0.661471 Y5.32165 Z5.50436

N11780 X-0.594435 Y5.32165 Z5.51278

N11790 X-0.508523 Y5.32165 Z5.52435

N11800 X-0.491662 Y5.32165 Z5.52688

N11810 X-0.426503 Y5.32165 Z5.53543

N11820 X-0.413455 Y5.32165 Z5.53731

N11830 X-0.3386 Y5.32165 Z5.54789

N11840 X-0.300329 Y5.32165 Z5.5536

N11850 X-0.246206 Y5.32165 Z5.56378

N11860 X-0.217479 Y5.32165 Z5.56818

N11870 X-0.170583 Y5.32165 Z5.57749

N11880 X-0.148765 Y5.32165 Z5.58147

N11890 X-0.111351 Y5.32165 Z5.58963

N11900 X-0.0885228 Y5.32165 Z5.59478

N11910 X-0.0595285 Y5.32165 Z5.60229

N11920 X-0.0344842 Y5.32165 Z5.60896

N11930 X-0.0109755 Y5.32165 Z5.61595

N11940 X0.0172946 Y5.32165 Z5.62447

N11950 X0.0361836 Y5.32165 Z5.63077

N11960 X0.0709468 Y5.32165 Z5.64272

N11970 X0.0849042 Y5.32165 Z5.64789

N11980 X0.141119 Y5.32165 Z5.66997

N11990 X0.210579 Y5.32165 Z5.69672

N12000 X0.262315 Y5.32165 Z5.71869

N12010 X0.290801 Y5.32165 Z5.72931

N12020 X0.352162 Y5.32165 Z5.7546

N12030 X0.376336 Y5.32165 Z5.76357

N12040 X0.433555 Y5.32165 Z5.78763

N12050 X0.456954 Y5.32165 Z5.7964

N12060 X0.508924 Y5.32165 Z5.81822

N12070 X0.534057 Y5.32165 Z5.82739

N12080 X0.568882 Y5.32165 Z5.83909

N12090 X0.586387 Y5.32165 Z5.84535

N12100 X0.63921 Y5.32165 Z5.86187

N12110 X0.663062 Y5.32165 Z5.86937

N12120 X0.71793 Y5.32165 Z5.88635

N12130 X0.739495 Y5.32165 Z5.89317

N12140 X0.8085 Y5.32165 Z5.9152

N12150 X0.82028 Y5.32165 Z5.91894

N12160 X0.902337 Y5.32165 Z5.94308

N12170 X0.930403 Y5.32165 Z5.95047

N12180 X0.987741 Y5.32165 Z5.96412

N12190 X1.02182 Y5.32165 Z5.97141

N12200 X1.07918 Y5.32165 Z5.98122

N12210 X1.1199 Y5.32165 Z5.9875

N12220 X1.1797 Y5.32165 Z5.99495

N12230 X1.21351 Y5.32165 Z5.99809

N12240 X1.2953 Y5.32165 Z6.00276

N12250 X1.30972 Y5.32165 Z6.00305

N12260 X1.33925 Y5.32165 Z6.00289

N12270 X1.40224 Y5.32165 Z5.99915

N12280 X1.43483 Y5.32165 Z5.99718

N12290 X1.51738 Y5.32165 Z5.99374

N12300 X1.57583 Y5.32165 Z5.98908

N12310 X1.63927 Y5.32165 Z5.98488

N12320 X1.70634 Y5.32165 Z5.98423

N12330 X1.76187 Y5.32165 Z5.97858

N12340 X1.85202 Y5.32165 Z5.9738

N12350 X1.94027 Y5.32165 Z5.95871

N12360 X1.99452 Y5.32165 Z5.9479

N12370 X2.03435 Y5.32165 Z5.93852

N12380 X2.10926 Y5.32165 Z5.91921

N12390 X2.1509 Y5.32165 Z5.90598

N12400 X2.22667 Y5.32165 Z5.87972

N12410 X2.27436 Y5.32165 Z5.86401

N12420 X2.35977 Y5.32165 Z5.82899

N12430 X2.39072 Y5.32165 Z5.81639

N12440 X2.43155 Y5.32165 Z5.79809

N12450 X2.50667 Y5.32165 Z5.76031

N12460 X2.6273 Y5.32165 Z5.69251

N12470 X2.66195 Y5.32165 Z5.66801

N12480 X2.76912 Y5.32165 Z5.58975

N12490 X2.79007 Y5.32165 Z5.57316

N12500 X2.86565 Y5.32165 Z5.50496

N12510 X2.90499 Y5.32165 Z5.46885

N12520 X2.99865 Y5.32165 Z5.36581

N12530 X3.00637 Y5.32165 Z5.35541

N12540 X3.065 Y5.32165 Z5.26954

N12550 X3.10589 Y5.32165 Z5.22057

N12560 X3.20669 Y5.32165 Z5.09065

N12570 X3.35012 Y5.2642 Z5.09101

N12580 X3.2794 Y5.2642 Z5.19047

N12590 X3.19973 Y5.2642 Z5.29368

N12600 X3.16456 Y5.2642 Z5.34111

N12610 X3.11157 Y5.2642 Z5.41073

N12620 X3.07228 Y5.2642 Z5.46415

N12630 X3.04635 Y5.2642 Z5.49288

N12640 X2.95895 Y5.2642 Z5.58187

N12650 X2.92701 Y5.2642 Z5.61069

N12660 X2.82565 Y5.2642 Z5.69095

N12670 X2.79142 Y5.2642 Z5.71594

N12680 X2.69538 Y5.2642 Z5.77461

N12690 X2.63404 Y5.2642 Z5.80907

N12700 X2.57018 Y5.2642 Z5.83927

N12710 X2.45734 Y5.2642 Z5.88944

N12720 X2.35352 Y5.2642 Z5.92736

N12730 X2.31296 Y5.2642 Z5.94082

N12740 X2.26272 Y5.2642 Z5.95783

N12750 X2.23474 Y5.2642 Z5.96464

N12760 X2.20602 Y5.2642 Z5.97295

N12770 X2.15791 Y5.2642 Z5.98586

N12780 X2.0915 Y5.2642 Z6.00474

N12790 X2.07282 Y5.2642 Z6.00878

N12800 X1.98784 Y5.2642 Z6.02474

N12810 X1.92791 Y5.2642 Z6.03314

N12820 X1.88504 Y5.2642 Z6.03869

N12830 X1.83519 Y5.2642 Z6.04298

N12840 X1.77766 Y5.2642 Z6.04437

N12850 X1.70781 Y5.2642 Z6.04774

N12860 X1.66549 Y5.2642 Z6.04815

N12870 X1.61852 Y5.2642 Z6.05125

N12880 X1.55155 Y5.2642 Z6.05087

N12890 X1.52844 Y5.2642 Z6.05089

N12900 X1.43631 Y5.2642 Z6.05335

N12910 X1.41124 Y5.2642 Z6.053

N12920 X1.36462 Y5.2642 Z6.0557

N12930 X1.32506 Y5.2642 Z6.05592

N12940 X1.23695 Y5.2642 Z6.05414

N12950 X1.14751 Y5.2642 Z6.04411

N12960 X1.13473 Y5.2642 Z6.04226

N12970 X1.05768 Y5.2642 Z6.02965

N12980 X1.03742 Y5.2642 Z6.02543

N12990 X0.968418 Y5.2642 Z6.01112

N13000 X0.948478 Y5.2642 Z6.00581

N13010 X0.893963 Y5.2642 Z5.9892

N13020 X0.838835 Y5.2642 Z5.97271

N13030 X0.813912 Y5.2642 Z5.9647

N13040 X0.741843 Y5.2642 Z5.9418

N13050 X0.713745 Y5.2642 Z5.93291

N13060 X0.673241 Y5.2642 Z5.9191

N13070 X0.605457 Y5.2642 Z5.89682

N13080 X0.541137 Y5.2642 Z5.87511

N13090 X0.479573 Y5.2642 Z5.84976

N13100 X0.46009 Y5.2642 Z5.84186

N13110 X0.42837 Y5.2642 Z5.82567

N13120 X0.381432 Y5.2642 Z5.80615

N13130 X0.350348 Y5.2642 Z5.79228

N13140 X0.301952 Y5.2642 Z5.77236

N13150 X0.268415 Y5.2642 Z5.75764

N13160 X0.214851 Y5.2642 Z5.73569

N13170 X0.191582 Y5.2642 Z5.7251

N13180 X0.157489 Y5.2642 Z5.71097

N13190 X0.126894 Y5.2642 Z5.69773

N13200 X0.0846265 Y5.2642 Z5.68116

N13210 X0.0733426 Y5.2642 Z5.67637

N13220 X0.0249423 Y5.2642 Z5.65772

N13230 X-0.0213967 Y5.2642 Z5.64145

N13240 X-0.0674675 Y5.2642 Z5.62678

N13250 X-0.0792166 Y5.2642 Z5.62317

N13260 X-0.114905 Y5.2642 Z5.61341

N13270 X-0.132226 Y5.2642 Z5.60893

N13280 X-0.166735 Y5.2642 Z5.6009

N13290 X-0.190851 Y5.2642 Z5.59563

N13300 X-0.224363 Y5.2642 Z5.58909

N13310 X-0.250208 Y5.2642 Z5.58298

N13320 X-0.317384 Y5.2642 Z5.5712

N13330 X-0.3342 Y5.2642 Z5.56738

N13340 X-0.35486 Y5.2642 Z5.56395

N13350 X-0.420191 Y5.2642 Z5.5527

N13360 X-0.464419 Y5.2642 Z5.54557

N13370 X-0.499385 Y5.2642 Z5.54032

N13380 X-0.548941 Y5.2642 Z5.53306

N13390 X-0.575891 Y5.2642 Z5.52942

N13400 X-0.628204 Y5.2642 Z5.52274

N13410 X-0.6515 Y5.2642 Z5.51982

N13420 X-0.710245 Y5.2642 Z5.51356

N13430 X-0.730343 Y5.2642 Z5.51131

N13440 X-0.798664 Y5.2642 Z5.50543

N13450 X-0.895003 Y5.2642 Z5.49832

N13460 X-0.912014 Y5.2642 Z5.49705

N13470 X-0.99883 Y5.2642 Z5.49201

N13480 X-1.02035 Y5.2642 Z5.49085

N13490 X-1.1063 Y5.2642 Z5.48644

N13500 X-1.14667 Y5.2642 Z5.48584

N13510 X-1.20737 Y5.2642 Z5.48152

N13520 X-1.27171 Y5.2642 Z5.48601

N13530 X-1.31931 Y5.2642 Z5.48597

N13540 X-1.44318 Y5.2642 Z5.4992

N13550 X-1.58228 Y5.2642 Z5.51149

N13560 X-1.66604 Y5.2642 Z5.52438

N13570 X-1.87468 Y5.2642 Z5.5535

N13580 X-1.94167 Y5.2642 Z5.5633

N13590 X-2.01709 Y5.2642 Z5.5779

N13600 X-2.10463 Y5.2642 Z5.5905

N13610 X-2.17929 Y5.2642 Z5.60498

N13620 X-2.24221 Y5.2642 Z5.6155

N13630 X-2.32117 Y5.2642 Z5.63232

N13640 X-2.36039 Y5.2642 Z5.63863

N13650 X-2.38495 Y5.2642 Z5.64366

N13660 X-2.47063 Y5.2642 Z5.65881

N13670 X-2.54774 Y5.2642 Z5.67709

N13680 X-2.56408 Y5.2642 Z5.67976

N13690 X-2.66505 Y5.2642 Z5.69493

N13700 X-2.72903 Y5.2642 Z5.70411

N13710 X-2.81516 Y5.2642 Z5.71397

N13720 X-2.9257 Y5.2642 Z5.72973

N13730 X-2.95984 Y5.2642 Z5.73264

N13740 X-2.9863 Y5.2642 Z5.73548

N13750 X-3.09229 Y5.2642 Z5.74237

N13760 X-3.22408 Y5.2642 Z5.75295

N13770 X-3.34609 Y5.2642 Z5.74931

N13780 X-3.4297 Y5.2642 Z5.75073

N13790 X-3.4845 Y5.2642 Z5.74551

N13800 X-3.61854 Y5.2642 Z5.73482

N13810 X-3.69386 Y5.2642 Z5.72123

N13820 X-3.76279 Y5.2642 Z5.70828

N13830 X-3.84554 Y5.2642 Z5.68531

N13840 X-3.89701 Y5.2642 Z5.67007

N13850 X-4.00986 Y5.2642 Z5.62783

N13860 X-4.04139 Y5.2642 Z5.61331

N13870 X-4.12157 Y5.2642 Z5.57799

N13880 X-4.21313 Y5.2642 Z5.53235

N13890 X-4.2366 Y5.2642 Z5.52063

N13900 X-4.26938 Y5.2642 Z5.50232

N13910 X-4.34745 Y5.2642 Z5.4452

N13920 X-4.4389 Y5.2642 Z5.37109

N13930 X-4.45785 Y5.2642 Z5.35548

N13940 X-4.57298 Y5.2642 Z5.22967

N13950 X-4.63423 Y5.2642 Z5.1505

N13960 X-4.66676 Y5.2642 Z5.11095

N13970 X-4.72581 Y5.2642 Z5.02007

N13980 X-4.75305 Y5.2642 Z4.98081

N13990 X-4.78418 Y5.2642 Z4.92872

N14000 X-4.81633 Y5.2642 Z4.87914

N14010 X-4.86078 Y5.2642 Z4.80562

N14020 X-4.96238 Y5.20334 Z4.78969

N14030 X-4.92164 Y5.20334 Z4.86377

N14040 X-4.89428 Y5.20334 Z4.909

N14050 X-4.83878 Y5.20334 Z5.01019

N14060 X-4.82948 Y5.20334 Z5.02737

N14070 X-4.82335 Y5.20334 Z5.03567

N14080 X-4.73362 Y5.20334 Z5.15914

N14090 X-4.64924 Y5.20334 Z5.26455

N14100 X-4.64165 Y5.20334 Z5.27256

N14110 X-4.55818 Y5.20334 Z5.35801

N14120 X-4.54226 Y5.20334 Z5.37577

N14130 X-4.46063 Y5.20334 Z5.44205

N14140 X-4.39477 Y5.20334 Z5.49673

N14150 X-4.35807 Y5.20334 Z5.51869

N14160 X-4.31969 Y5.20334 Z5.54051

N14170 X-4.24817 Y5.20334 Z5.58047

N14180 X-4.15051 Y5.20334 Z5.62924

N14190 X-4.13941 Y5.20334 Z5.63356

N14200 X-4.02797 Y5.20334 Z5.67368

N14210 X-3.98496 Y5.20334 Z5.6872

N14220 X-3.91406 Y5.20334 Z5.71082

N14230 X-3.845 Y5.20334 Z5.72876

N14240 X-3.79256 Y5.20334 Z5.74332

N14250 X-3.70632 Y5.20334 Z5.75893

N14260 X-3.65763 Y5.20334 Z5.76811

N14270 X-3.57592 Y5.20334 Z5.77329

N14280 X-3.48413 Y5.20334 Z5.78157

N14290 X-3.44746 Y5.20334 Z5.77997

N14300 X-3.36895 Y5.20334 Z5.78158

N14310 X-3.32257 Y5.20334 Z5.78079

N14320 X-3.29793 Y5.20334 Z5.78153

N14330 X-3.20522 Y5.20334 Z5.77547

N14340 X-3.11106 Y5.20334 Z5.77036

N14350 X-3.08389 Y5.20334 Z5.76813

N14360 X-3.04681 Y5.20334 Z5.76572

N14370 X-2.95256 Y5.20334 Z5.75352

N14380 X-2.85968 Y5.20334 Z5.74457

N14390 X-2.80877 Y5.20334 Z5.73731

N14400 X-2.74208 Y5.20334 Z5.72968

N14410 X-2.67027 Y5.20334 Z5.71769

N14420 X-2.63095 Y5.20334 Z5.71144

N14430 X-2.54903 Y5.20334 Z5.69658

N14440 X-2.45646 Y5.20334 Z5.67922

N14450 X-2.44661 Y5.20334 Z5.67689

N14460 X-2.43292 Y5.20334 Z5.67447

N14470 X-2.3492 Y5.20334 Z5.65416

N14480 X-2.28737 Y5.20334 Z5.6425

N14490 X-2.23779 Y5.20334 Z5.63193

N14500 X-2.16013 Y5.20334 Z5.61895

N14510 X-2.10834 Y5.20334 Z5.60764

N14520 X-2.06711 Y5.20334 Z5.60073

N14530 X-1.95622 Y5.20334 Z5.57927

N14540 X-1.76925 Y5.20334 Z5.5519

N14550 X-1.58266 Y5.20334 Z5.52319

N14560 X-1.52001 Y5.20334 Z5.51766

N14570 X-1.44254 Y5.20334 Z5.50656

N14580 X-1.33335 Y5.20334 Z5.5015

N14590 X-1.30729 Y5.20334 Z5.50066

N14600 X-1.21985 Y5.20334 Z5.49455

N14610 X-1.15487 Y5.20334 Z5.49917

N14620 X-1.12888 Y5.20334 Z5.49884

N14630 X-1.11373 Y5.20334 Z5.49934

N14640 X-1.02503 Y5.20334 Z5.5019

N14650 X-0.996166 Y5.20334 Z5.50321

N14660 X-0.922781 Y5.20334 Z5.50697

N14670 X-0.891931 Y5.20334 Z5.50883

N14680 X-0.827614 Y5.20334 Z5.51345

N14690 X-0.799833 Y5.20334 Z5.5157

N14700 X-0.740621 Y5.20334 Z5.52121

N14710 X-0.716577 Y5.20334 Z5.52375

N14720 X-0.660367 Y5.20334 Z5.53008

N14730 X-0.638816 Y5.20334 Z5.5329

N14740 X-0.584546 Y5.20334 Z5.53998

N14750 X-0.563332 Y5.20334 Z5.54329

N14760 X-0.508503 Y5.20334 Z5.55155

N14770 X-0.487793 Y5.20334 Z5.55526

N14780 X-0.425521 Y5.20334 Z5.5662

N14790 X-0.413676 Y5.20334 Z5.56856

N14800 X-0.390281 Y5.20334 Z5.57352

N14810 X-0.343185 Y5.20334 Z5.58133

N14820 X-0.286454 Y5.20334 Z5.59423

N14830 X-0.274766 Y5.20334 Z5.59663

N14840 X-0.216959 Y5.20334 Z5.6093

N14850 X-0.181371 Y5.20334 Z5.61807

N14860 X-0.167917 Y5.20334 Z5.62145

N14870 X-0.123792 Y5.20334 Z5.63383

N14880 X-0.0830579 Y5.20334 Z5.64685

N14890 X-0.0293439 Y5.20334 Z5.66546

N14900 X0.0174618 Y5.20334 Z5.6837

N14910 X0.0358006 Y5.20334 Z5.69134

N14920 X0.0629904 Y5.20334 Z5.7025

N14930 X0.106768 Y5.20334 Z5.72035

N14940 X0.163648 Y5.20334 Z5.74669

N14950 X0.242125 Y5.20334 Z5.78169

N14960 X0.257272 Y5.20334 Z5.78855

N14970 X0.326348 Y5.20334 Z5.81965

N14980 X0.389492 Y5.20334 Z5.84884

N14990 X0.408325 Y5.20334 Z5.85724

N15000 X0.478051 Y5.20334 Z5.88857

N15010 X0.502725 Y5.20334 Z5.89805

N15020 X0.562558 Y5.20334 Z5.92363

N15030 X0.616355 Y5.20334 Z5.94101

N15040 X0.628399 Y5.20334 Z5.94498

N15050 X0.673905 Y5.20334 Z5.95979

N15060 X0.695537 Y5.20334 Z5.96721

N15070 X0.737122 Y5.20334 Z5.98159

N15080 X0.772462 Y5.20334 Z5.99413

N15090 X0.810377 Y5.20334 Z6.00723

N15100 X0.870282 Y5.20334 Z6.02777

N15110 X0.890795 Y5.20334 Z6.03437

N15120 X0.980965 Y5.20334 Z6.06005

N15130 X0.995962 Y5.20334 Z6.06336

N15140 X1.07788 Y5.20334 Z6.08042

N15150 X1.16716 Y5.20334 Z6.09347

N15160 X1.18648 Y5.20334 Z6.09564

N15170 X1.24992 Y5.20334 Z6.10103

N15180 X1.28996 Y5.20334 Z6.1032

N15190 X1.32979 Y5.20334 Z6.10459

N15200 X1.37516 Y5.20334 Z6.10589

N15210 X1.40191 Y5.20334 Z6.10435

N15220 X1.47629 Y5.20334 Z6.10538

N15230 X1.491 Y5.20334 Z6.10499

N15240 X1.5772 Y5.20334 Z6.10491

N15250 X1.62223 Y5.20334 Z6.10517

N15260 X1.68297 Y5.20334 Z6.10488

N15270 X1.76774 Y5.20334 Z6.10695

N15280 X1.80287 Y5.20334 Z6.10526

N15290 X1.84242 Y5.20334 Z6.1043

N15300 X1.91885 Y5.20334 Z6.10066

N15310 X2.0085 Y5.20334 Z6.09156

N15320 X2.02326 Y5.20334 Z6.08968

N15330 X2.04835 Y5.20334 Z6.08583

N15340 X2.10729 Y5.20334 Z6.07308

N15350 X2.13764 Y5.20334 Z6.06652

N15360 X2.17987 Y5.20334 Z6.05645

N15370 X2.24245 Y5.20334 Z6.04349

N15380 X2.25621 Y5.20334 Z6.04054

N15390 X2.31745 Y5.20334 Z6.0243

N15400 X2.35723 Y5.20334 Z6.01252

N15410 X2.40015 Y5.20334 Z6.00068

N15420 X2.46733 Y5.20334 Z5.98078

N15430 X2.50349 Y5.20334 Z5.9679

N15440 X2.57268 Y5.20334 Z5.94204

N15450 X2.62579 Y5.20334 Z5.92231

N15460 X2.64721 Y5.20334 Z5.91398

N15470 X2.754 Y5.20334 Z5.8635

N15480 X2.80398 Y5.20334 Z5.83806

N15490 X2.88082 Y5.20334 Z5.79097

N15500 X2.94704 Y5.20334 Z5.74733

N15510 X3.00759 Y5.20334 Z5.69942

N15520 X3.07219 Y5.20334 Z5.64236

N15530 X3.19881 Y5.20334 Z5.50629

N15540 X3.23632 Y5.20334 Z5.457

N15550 X3.29375 Y5.20334 Z5.37956

N15560 X3.32906 Y5.20334 Z5.33266

N15570 X3.34826 Y5.20334 Z5.30253

N15580 X3.41196 Y5.20334 Z5.2128

N15590 X3.44715 Y5.20334 Z5.15611

N15600 X3.49054 Y5.20334 Z5.091

N15610 X3.6207 Y5.14302 Z5.08911

N15620 X3.58337 Y5.14302 Z5.14913

N15630 X3.53305 Y5.14302 Z5.23385

N15640 X3.46933 Y5.14302 Z5.33767

N15650 X3.45698 Y5.14302 Z5.35789

N15660 X3.39257 Y5.14302 Z5.45978

N15670 X3.32602 Y5.14302 Z5.5427

N15680 X3.29312 Y5.14302 Z5.57877

N15690 X3.21063 Y5.14302 Z5.67148

N15700 X3.176 Y5.14302 Z5.70458

N15710 X3.0922 Y5.14302 Z5.77859

N15720 X3.05542 Y5.14302 Z5.80546

N15730 X2.95663 Y5.14302 Z5.87087

N15740 X2.93367 Y5.14302 Z5.88358

N15750 X2.80815 Y5.14302 Z5.94768

N15760 X2.78689 Y5.14302 Z5.95585

N15770 X2.6697 Y5.14302 Z6.00145

N15780 X2.63566 Y5.14302 Z6.0141

N15790 X2.52907 Y5.14302 Z6.04529

N15800 X2.46136 Y5.14302 Z6.06487

N15810 X2.42705 Y5.14302 Z6.07274

N15820 X2.4034 Y5.14302 Z6.07794

N15830 X2.34233 Y5.14302 Z6.09222

N15840 X2.28933 Y5.14302 Z6.10268

N15850 X2.26673 Y5.14302 Z6.10756

N15860 X2.2173 Y5.14302 Z6.11796

N15870 X2.19208 Y5.14302 Z6.12185

N15880 X2.12791 Y5.14302 Z6.13215

N15890 X2.08286 Y5.14302 Z6.14001

N15900 X2.04659 Y5.14302 Z6.14557

N15910 X1.97671 Y5.14302 Z6.15447

N15920 X1.95195 Y5.14302 Z6.15626

N15930 X1.93929 Y5.14302 Z6.15705

N15940 X1.84322 Y5.14302 Z6.15903

N15950 X1.80172 Y5.14302 Z6.15792

N15960 X1.71814 Y5.14302 Z6.15804

N15970 X1.69671 Y5.14302 Z6.15752

N15980 X1.67565 Y5.14302 Z6.15762

N15990 X1.59625 Y5.14302 Z6.15296

N16000 X1.568 Y5.14302 Z6.15177

N16010 X1.50129 Y5.14302 Z6.14967

N16020 X1.46366 Y5.14302 Z6.14786

N16030 X1.41396 Y5.14302 Z6.14818

N16040 X1.38526 Y5.14302 Z6.14667

N16050 X1.3245 Y5.14302 Z6.14779

N16060 X1.26313 Y5.14302 Z6.14315

N16070 X1.2368 Y5.14302 Z6.14117

N16080 X1.18441 Y5.14302 Z6.13541

N16090 X1.13388 Y5.14302 Z6.1289

N16100 X1.09625 Y5.14302 Z6.12287

N16110 X1.0231 Y5.14302 Z6.10799

N16120 X0.997724 Y5.14302 Z6.10252

N16130 X0.912491 Y5.14302 Z6.07922

N16140 X0.898537 Y5.14302 Z6.07524

N16150 X0.833885 Y5.14302 Z6.05447

N16160 X0.804939 Y5.14302 Z6.04447

N16170 X0.733285 Y5.14302 Z6.01754

N16180 X0.717825 Y5.14302 Z6.01173

N16190 X0.669058 Y5.14302 Z5.99342

N16200 X0.64601 Y5.14302 Z5.98513

N16210 X0.609536 Y5.14302 Z5.97203

N16220 X0.567222 Y5.14302 Z5.95708

N16230 X0.544902 Y5.14302 Z5.94897

N16240 X0.512436 Y5.14302 Z5.93641

N16250 X0.47128 Y5.14302 Z5.91814

N16260 X0.430452 Y5.14302 Z5.89836

N16270 X0.388436 Y5.14302 Z5.87913

N16280 X0.358102 Y5.14302 Z5.8651

N16290 X0.299646 Y5.14302 Z5.83817

N16300 X0.280674 Y5.14302 Z5.82963

N16310 X0.225282 Y5.14302 Z5.80455

N16320 X0.206657 Y5.14302 Z5.79554

N16330 X0.18959 Y5.14302 Z5.78748

N16340 X0.140861 Y5.14302 Z5.76373

N16350 X0.121155 Y5.14302 Z5.75506

N16360 X0.0833714 Y5.14302 Z5.73668

N16370 X0.0377602 Y5.14302 Z5.71723

N16380 X0.0268674 Y5.14302 Z5.71244

N16390 X-0.0209403 Y5.14302 Z5.6914

N16400 X-0.032572 Y5.14302 Z5.6869

N16410 X-0.0707963 Y5.14302 Z5.67122

N16420 X-0.0832277 Y5.14302 Z5.66677

N16430 X-0.117995 Y5.14302 Z5.65413

N16440 X-0.12816 Y5.14302 Z5.65087

N16450 X-0.173584 Y5.14302 Z5.63715

N16460 X-0.187608 Y5.14302 Z5.63362

N16470 X-0.225648 Y5.14302 Z5.62263

N16480 X-0.250441 Y5.14302 Z5.61647

N16490 X-0.28545 Y5.14302 Z5.60726

N16500 X-0.313744 Y5.14302 Z5.60143

N16510 X-0.352832 Y5.14302 Z5.59125

N16520 X-0.398671 Y5.14302 Z5.58238

N16530 X-0.409322 Y5.14302 Z5.58012

N16540 X-0.473708 Y5.14302 Z5.56739

N16550 X-0.548489 Y5.14302 Z5.55412

N16560 X-0.624121 Y5.14302 Z5.54256

N16570 X-0.688623 Y5.14302 Z5.53413

N16580 X-0.701361 Y5.14302 Z5.5327

N16590 X-0.768012 Y5.14302 Z5.52568

N16600 X-0.782664 Y5.14302 Z5.52431

N16610 X-0.854225 Y5.14302 Z5.51852

N16620 X-0.871172 Y5.14302 Z5.51731

N16630 X-0.948726 Y5.14302 Z5.51265

N16640 X-0.971049 Y5.14302 Z5.5115

N16650 X-1.049 Y5.14302 Z5.50798

N16660 X-1.08308 Y5.14302 Z5.50699

N16670 X-1.14398 Y5.14302 Z5.50499

N16680 X-1.21668 Y5.14302 Z5.50592

N16690 X-1.22992 Y5.14302 Z5.5051

N16700 X-1.24038 Y5.14302 Z5.50567

N16710 X-1.32812 Y5.14302 Z5.50484

N16720 X-1.39437 Y5.14302 Z5.51191

N16730 X-1.44461 Y5.14302 Z5.51423

N16740 X-1.55988 Y5.14302 Z5.53075

N16750 X-1.58215 Y5.14302 Z5.53281

N16760 X-1.59528 Y5.14302 Z5.53513

N16770 X-1.74669 Y5.14302 Z5.55389

N16780 X-1.80344 Y5.14302 Z5.5635

N16790 X-1.97063 Y5.14302 Z5.59506

N16800 X-2.11297 Y5.14302 Z5.61894

N16810 X-2.19983 Y5.14302 Z5.63789

N16820 X-2.23493 Y5.14302 Z5.64467

N16830 X-2.28945 Y5.14302 Z5.65787

N16840 X-2.33923 Y5.14302 Z5.66726

N16850 X-2.36723 Y5.14302 Z5.67406

N16860 X-2.43826 Y5.14302 Z5.6885

N16870 X-2.54248 Y5.14302 Z5.71326

N16880 X-2.66606 Y5.14302 Z5.73292

N16890 X-2.7445 Y5.14302 Z5.74601

N16900 X-2.80506 Y5.14302 Z5.75377

N16910 X-2.871 Y5.14302 Z5.76384

N16920 X-2.94849 Y5.14302 Z5.77131

N16930 X-3.01134 Y5.14302 Z5.77944

N16940 X-3.08444 Y5.14302 Z5.78595

N16950 X-3.18455 Y5.14302 Z5.79774

N16960 X-3.19717 Y5.14302 Z5.79849

N16970 X-3.2942 Y5.14302 Z5.8002

N16980 X-3.34893 Y5.14302 Z5.80589

N16990 X-3.41735 Y5.14302 Z5.80449

N17000 X-3.5357 Y5.14302 Z5.80958

N17010 X-3.65837 Y5.14302 Z5.79962

N17020 X-3.69098 Y5.14302 Z5.79706

N17030 X-3.79313 Y5.14302 Z5.77967

N17040 X-3.81687 Y5.14302 Z5.77596

N17050 X-3.93143 Y5.14302 Z5.7497

N17060 X-3.94146 Y5.14302 Z5.74717

N17070 X-4.04208 Y5.14302 Z5.71555

N17080 X-4.07429 Y5.14302 Z5.70394

N17090 X-4.14908 Y5.14302 Z5.67746

N17100 X-4.23301 Y5.14302 Z5.64486

N17110 X-4.25728 Y5.14302 Z5.63528

N17120 X-4.29514 Y5.14302 Z5.61924

N17130 X-4.36314 Y5.14302 Z5.58057

N17140 X-4.43486 Y5.14302 Z5.53766

N17150 X-4.4672 Y5.14302 Z5.51735

N17160 X-4.48796 Y5.14302 Z5.50428

N17170 X-4.56524 Y5.14302 Z5.43775

N17180 X-4.61304 Y5.14302 Z5.39883

N17190 X-4.65919 Y5.14302 Z5.34941

N17200 X-4.71807 Y5.14302 Z5.29343

N17210 X-4.74489 Y5.14302 Z5.25993

N17220 X-4.81884 Y5.14302 Z5.18044

N17230 X-4.90929 Y5.14302 Z5.05636

N17240 X-4.97009 Y5.14302 Z4.9426

N17250 X-4.98721 Y5.14302 Z4.90345

N17260 X-5.03876 Y5.14302 Z4.81011

N17270 X-5.0563 Y5.14302 Z4.77649

N17280 X-5.14918 Y5.07976 Z4.76471

N17290 X-5.08853 Y5.07976 Z4.88038

N17300 X-4.97483 Y5.07976 Z5.10796

N17310 X-4.91103 Y5.07976 Z5.18723

N17320 X-4.89994 Y5.07976 Z5.2023

N17330 X-4.83725 Y5.07976 Z5.26583

N17340 X-4.79519 Y5.07976 Z5.31391

N17350 X-4.75693 Y5.07976 Z5.34674

N17360 X-4.68138 Y5.07976 Z5.42324

N17370 X-4.66797 Y5.07976 Z5.43386

N17380 X-4.59334 Y5.07976 Z5.49714

N17390 X-4.5705 Y5.07976 Z5.51573

N17400 X-4.46689 Y5.07976 Z5.58204

N17410 X-4.40777 Y5.07976 Z5.61915

N17420 X-4.3651 Y5.07976 Z5.63867

N17430 X-4.32818 Y5.07976 Z5.65488

N17440 X-4.26175 Y5.07976 Z5.68303

N17450 X-4.15946 Y5.07976 Z5.72341

N17460 X-4.05325 Y5.07976 Z5.75331

N17470 X-4.01438 Y5.07976 Z5.76314

N17480 X-3.94544 Y5.07976 Z5.78119

N17490 X-3.87502 Y5.07976 Z5.79642

N17500 X-3.83242 Y5.07976 Z5.80618

N17510 X-3.73817 Y5.07976 Z5.8209

N17520 X-3.71177 Y5.07976 Z5.82539

N17530 X-3.61051 Y5.07976 Z5.83332

N17540 X-3.58823 Y5.07976 Z5.83513

N17550 X-3.50885 Y5.07976 Z5.8298

N17560 X-3.39526 Y5.07976 Z5.82961

N17570 X-3.36913 Y5.07976 Z5.82739

N17580 X-3.26264 Y5.07976 Z5.8163

N17590 X-3.21883 Y5.07976 Z5.81553

N17600 X-3.13097 Y5.07976 Z5.80633

N17610 X-3.08879 Y5.07976 Z5.80136

N17620 X-2.99898 Y5.07976 Z5.79337

N17630 X-2.94801 Y5.07976 Z5.78625

N17640 X-2.90027 Y5.07976 Z5.78073

N17650 X-2.80316 Y5.07976 Z5.7659

N17660 X-2.69815 Y5.07976 Z5.75245

N17670 X-2.66329 Y5.07976 Z5.74625

N17680 X-2.63709 Y5.07976 Z5.74171

N17690 X-2.54024 Y5.07976 Z5.72178

N17700 X-2.44562 Y5.07976 Z5.7032

N17710 X-2.43152 Y5.07976 Z5.69985

N17720 X-2.41188 Y5.07976 Z5.69585

N17730 X-2.3354 Y5.07976 Z5.67635

N17740 X-2.28758 Y5.07976 Z5.66607

N17750 X-2.23404 Y5.07976 Z5.6531

N17760 X-2.12457 Y5.07976 Z5.63197

N17770 X-2.11412 Y5.07976 Z5.62961

N17780 X-1.98479 Y5.07976 Z5.60128

N17790 X-1.90191 Y5.07976 Z5.58743

N17800 X-1.83994 Y5.07976 Z5.57574

N17810 X-1.72341 Y5.07976 Z5.556

N17820 X-1.66981 Y5.07976 Z5.54936

N17830 X-1.57724 Y5.07976 Z5.53301

N17840 X-1.45992 Y5.07976 Z5.52215

N17850 X-1.44635 Y5.07976 Z5.51999

N17860 X-1.42302 Y5.07976 Z5.51905

N17870 X-1.33247 Y5.07976 Z5.50939

N17880 X-1.2963 Y5.07976 Z5.50973

N17890 X-1.23079 Y5.07976 Z5.5062

N17900 X-1.16433 Y5.07976 Z5.51028

N17910 X-1.0761 Y5.07976 Z5.51092

N17920 X-1.05164 Y5.07976 Z5.51173

N17930 X-0.976452 Y5.07976 Z5.51403

N17940 X-0.943831 Y5.07976 Z5.51556

N17950 X-0.881257 Y5.07976 Z5.51891

N17960 X-0.848364 Y5.07976 Z5.52122

N17970 X-0.795049 Y5.07976 Z5.5254

N17980 X-0.762871 Y5.07976 Z5.52865

N17990 X-0.71628 Y5.07976 Z5.53341

N18000 X-0.6827 Y5.07976 Z5.53782

N18010 X-0.64293 Y5.07976 Z5.54273

N18020 X-0.605093 Y5.07976 Z5.54884

N18030 X-0.573032 Y5.07976 Z5.55356

N18040 X-0.527397 Y5.07976 Z5.56234

N18050 X-0.502763 Y5.07976 Z5.56667

N18060 X-0.448528 Y5.07976 Z5.57861

N18070 X-0.431552 Y5.07976 Z5.58205

N18080 X-0.363791 Y5.07976 Z5.59852

N18090 X-0.295425 Y5.07976 Z5.61517

N18100 X-0.239329 Y5.07976 Z5.63183

N18110 X-0.229643 Y5.07976 Z5.63438

N18120 X-0.180839 Y5.07976 Z5.64879

N18130 X-0.163458 Y5.07976 Z5.6545

N18140 X-0.134056 Y5.07976 Z5.6651

N18150 X-0.11223 Y5.07976 Z5.67322

N18160 X-0.083223 Y5.07976 Z5.68554

N18170 X-0.0590212 Y5.07976 Z5.69531

N18180 X-0.0191666 Y5.07976 Z5.71366

N18190 X0.0572705 Y5.07976 Z5.74846

N18200 X0.0721058 Y5.07976 Z5.75596

N18210 X0.116405 Y5.07976 Z5.77642

N18220 X0.155642 Y5.07976 Z5.79674

N18230 X0.164894 Y5.07976 Z5.80134

N18240 X0.188242 Y5.07976 Z5.81323

N18250 X0.218999 Y5.07976 Z5.82823

N18260 X0.2326 Y5.07976 Z5.83479

N18270 X0.280521 Y5.07976 Z5.85728

N18280 X0.310663 Y5.07976 Z5.87202

N18290 X0.36451 Y5.07976 Z5.89781

N18300 X0.384528 Y5.07976 Z5.90654

N18310 X0.447432 Y5.07976 Z5.93688

N18320 X0.458709 Y5.07976 Z5.94208

N18330 X0.529098 Y5.07976 Z5.97177

N18340 X0.591592 Y5.07976 Z5.99699

N18350 X0.628744 Y5.07976 Z6.01158

N18360 X0.662518 Y5.07976 Z6.02373

N18370 X0.732208 Y5.07976 Z6.04993

N18380 X0.750036 Y5.07976 Z6.05663

N18390 X0.815101 Y5.07976 Z6.08038

N18400 X0.851751 Y5.07976 Z6.09292

N18410 X0.909795 Y5.07976 Z6.11267

N18420 X0.958994 Y5.07976 Z6.12657

N18430 X1.01598 Y5.07976 Z6.14264

N18440 X1.07247 Y5.07976 Z6.15472

N18450 X1.11475 Y5.07976 Z6.16256

N18460 X1.18885 Y5.07976 Z6.17366

N18470 X1.20106 Y5.07976 Z6.17497

N18480 X1.24048 Y5.07976 Z6.17909

N18490 X1.27753 Y5.07976 Z6.18188

N18500 X1.35592 Y5.07976 Z6.18638

N18510 X1.42773 Y5.07976 Z6.18955

N18520 X1.43851 Y5.07976 Z6.18994

N18530 X1.50651 Y5.07976 Z6.19347

N18540 X1.52386 Y5.07976 Z6.1943

N18550 X1.60995 Y5.07976 Z6.19794

N18560 X1.63905 Y5.07976 Z6.19964

N18570 X1.69826 Y5.07976 Z6.2019

N18580 X1.7818 Y5.07976 Z6.20895

N18590 X1.81045 Y5.07976 Z6.20959

N18600 X1.88314 Y5.07976 Z6.21062

N18610 X1.90695 Y5.07976 Z6.21044

N18620 X1.97518 Y5.07976 Z6.2062

N18630 X2.02048 Y5.07976 Z6.20292

N18640 X2.06407 Y5.07976 Z6.19865

N18650 X2.13483 Y5.07976 Z6.19208

N18660 X2.1453 Y5.07976 Z6.19025

N18670 X2.21761 Y5.07976 Z6.1793

N18680 X2.24659 Y5.07976 Z6.1748

N18690 X2.29288 Y5.07976 Z6.1695

N18700 X2.33621 Y5.07976 Z6.16217

N18710 X2.36888 Y5.07976 Z6.15802

N18720 X2.43867 Y5.07976 Z6.14535

N18730 X2.45522 Y5.07976 Z6.14248

N18740 X2.53804 Y5.07976 Z6.12339

N18750 X2.6072 Y5.07976 Z6.10911

N18760 X2.66278 Y5.07976 Z6.09281

N18770 X2.7371 Y5.07976 Z6.07312

N18780 X2.82798 Y5.07976 Z6.04275

N18790 X2.86228 Y5.07976 Z6.02957

N18800 X2.95976 Y5.07976 Z5.98534

N18810 X2.98991 Y5.07976 Z5.96988

N18820 X3.08525 Y5.07976 Z5.9171

N18830 X3.10476 Y5.07976 Z5.90608

N18840 X3.21688 Y5.07976 Z5.82431

N18850 X3.23687 Y5.07976 Z5.80839

N18860 X3.33643 Y5.07976 Z5.71389

N18870 X3.34898 Y5.07976 Z5.7012

N18880 X3.42907 Y5.07976 Z5.61218

N18890 X3.44308 Y5.07976 Z5.59548

N18900 X3.50312 Y5.07976 Z5.51444

N18910 X3.51253 Y5.07976 Z5.49993

N18920 X3.57171 Y5.07976 Z5.4052

N18930 X3.63573 Y5.07976 Z5.28951

N18940 X3.65438 Y5.07976 Z5.25667

N18950 X3.68383 Y5.07976 Z5.19605

N18960 X3.74869 Y5.07976 Z5.084

N18970 X3.87595 Y5.01514 Z5.07747

N18980 X3.82791 Y5.01514 Z5.16343

N18990 X3.79536 Y5.01514 Z5.21929

N19000 X3.78131 Y5.01514 Z5.24912

N19010 X3.7363 Y5.01514 Z5.33823

N19020 X3.69176 Y5.01514 Z5.42176

N19030 X3.67606 Y5.01514 Z5.44901

N19040 X3.64237 Y5.01514 Z5.50775

N19050 X3.6198 Y5.01514 Z5.54523

N19060 X3.6064 Y5.01514 Z5.56578

N19070 X3.564 Y5.01514 Z5.62807

N19080 X3.52239 Y5.01514 Z5.68012

N19090 X3.50287 Y5.01514 Z5.70367

N19100 X3.46878 Y5.01514 Z5.74179

N19110 X3.44015 Y5.01514 Z5.77125

N19120 X3.40537 Y5.01514 Z5.8059

N19130 X3.36787 Y5.01514 Z5.84152

N19140 X3.35279 Y5.01514 Z5.85425

N19150 X3.25906 Y5.01514 Z5.92907

N19160 X3.23754 Y5.01514 Z5.9432

N19170 X3.16305 Y5.01514 Z5.99056

N19180 X3.12861 Y5.01514 Z6.01

N19190 X3.06002 Y5.01514 Z6.04502

N19200 X3.0115 Y5.01514 Z6.0684

N19210 X2.96423 Y5.01514 Z6.08973

N19220 X2.90276 Y5.01514 Z6.1134

N19230 X2.85733 Y5.01514 Z6.12828

N19240 X2.80979 Y5.01514 Z6.1415

N19250 X2.77466 Y5.01514 Z6.14946

N19260 X2.70172 Y5.01514 Z6.16746

N19270 X2.6408 Y5.01514 Z6.17855

N19280 X2.58077 Y5.01514 Z6.1912

N19290 X2.53739 Y5.01514 Z6.19678

N19300 X2.48064 Y5.01514 Z6.20632

N19310 X2.45295 Y5.01514 Z6.21075

N19320 X2.43339 Y5.01514 Z6.21449

N19330 X2.38124 Y5.01514 Z6.22054

N19340 X2.33169 Y5.01514 Z6.22707

N19350 X2.30432 Y5.01514 Z6.22874

N19360 X2.24632 Y5.01514 Z6.23474

N19370 X2.21536 Y5.01514 Z6.2357

N19380 X2.16332 Y5.01514 Z6.24023

N19390 X2.09461 Y5.01514 Z6.24675

N19400 X2.07633 Y5.01514 Z6.24845

N19410 X2.04765 Y5.01514 Z6.25126

N19420 X1.98941 Y5.01514 Z6.2522

N19430 X1.95369 Y5.01514 Z6.25349

N19440 X1.90634 Y5.01514 Z6.25373

N19450 X1.83477 Y5.01514 Z6.25448

N19460 X1.77557 Y5.01514 Z6.24855

N19470 X1.69671 Y5.01514 Z6.24527

N19480 X1.61843 Y5.01514 Z6.23711

N19490 X1.58493 Y5.01514 Z6.23461

N19500 X1.53676 Y5.01514 Z6.23078

N19510 X1.48987 Y5.01514 Z6.2269

N19520 X1.45489 Y5.01514 Z6.22509

N19530 X1.40709 Y5.01514 Z6.22097

N19540 X1.37307 Y5.01514 Z6.21995

N19550 X1.33015 Y5.01514 Z6.21652

N19560 X1.29254 Y5.01514 Z6.21445

N19570 X1.24254 Y5.01514 Z6.21056

N19580 X1.21542 Y5.01514 Z6.20772

N19590 X1.13161 Y5.01514 Z6.19857

N19600 X1.03598 Y5.01514 Z6.17936

N19610 X1.00573 Y5.01514 Z6.17133

N19620 X0.925765 Y5.01514 Z6.14875

N19630 X0.899888 Y5.01514 Z6.13912

N19640 X0.823392 Y5.01514 Z6.11259

N19650 X0.794546 Y5.01514 Z6.10165

N19660 X0.735528 Y5.01514 Z6.07883

N19670 X0.699244 Y5.01514 Z6.06426

N19680 X0.66018 Y5.01514 Z6.04863

N19690 X0.617307 Y5.01514 Z6.03176

N19700 X0.591787 Y5.01514 Z6.02169

N19710 X0.515125 Y5.01514 Z5.99214

N19720 X0.433071 Y5.01514 Z5.95429

N19730 X0.411392 Y5.01514 Z5.94384

N19740 X0.354459 Y5.01514 Z5.91903

N19750 X0.337096 Y5.01514 Z5.91085

N19760 X0.264654 Y5.01514 Z5.87506

N19770 X0.250627 Y5.01514 Z5.86805

N19780 X0.209394 Y5.01514 Z5.84651

N19790 X0.153816 Y5.01514 Z5.81781

N19800 X0.0988423 Y5.01514 Z5.78872

N19810 X0.0484404 Y5.01514 Z5.76412

N19820 X0.0338493 Y5.01514 Z5.75675

N19830 X0.0212815 Y5.01514 Z5.75109

N19840 X-0.0315664 Y5.01514 Z5.72481

N19850 X-0.0701741 Y5.01514 Z5.70774

N19860 X-0.0959026 Y5.01514 Z5.6953

N19870 X-0.135243 Y5.01514 Z5.67868

N19880 X-0.154715 Y5.01514 Z5.67033

N19890 X-0.187378 Y5.01514 Z5.65788

N19900 X-0.217424 Y5.01514 Z5.64741

N19910 X-0.241185 Y5.01514 Z5.63947

N19920 X-0.289287 Y5.01514 Z5.62554

N19930 X-0.304438 Y5.01514 Z5.62043

N19940 X-0.319856 Y5.01514 Z5.61605

N19950 X-0.379155 Y5.01514 Z5.59849

N19960 X-0.413228 Y5.01514 Z5.59072

N19970 X-0.460144 Y5.01514 Z5.57831

N19980 X-0.501575 Y5.01514 Z5.56954

N19990 X-0.533157 Y5.01514 Z5.56212

N20000 X-0.582754 Y5.01514 Z5.55328

N20010 X-0.601781 Y5.01514 Z5.54948

N20020 X-0.670766 Y5.01514 Z5.5392

N20030 X-0.743668 Y5.01514 Z5.53044

N20040 X-0.777475 Y5.01514 Z5.52729

N20050 X-0.824521 Y5.01514 Z5.52259

N20060 X-0.915391 Y5.01514 Z5.51631

N20070 X-1.00515 Y5.01514 Z5.5121

N20080 X-1.02056 Y5.01514 Z5.51163

N20090 X-1.1067 Y5.01514 Z5.50877

N20100 X-1.13432 Y5.01514 Z5.50855

N20110 X-1.2133 Y5.01514 Z5.50845

N20120 X-1.23169 Y5.01514 Z5.50732

N20130 X-1.28893 Y5.01514 Z5.51041

N20140 X-1.33485 Y5.01514 Z5.51028

N20150 X-1.35285 Y5.01514 Z5.51194

N20160 X-1.44623 Y5.01514 Z5.51571

N20170 X-1.53386 Y5.01514 Z5.52966

N20180 X-1.57223 Y5.01514 Z5.53321

N20190 X-1.6419 Y5.01514 Z5.54552

N20200 X-1.70593 Y5.01514 Z5.55395

N20210 X-1.73315 Y5.01514 Z5.55964

N20220 X-1.84413 Y5.01514 Z5.57872

N20230 X-1.86903 Y5.01514 Z5.58264

N20240 X-1.93257 Y5.01514 Z5.5965

N20250 X-1.99924 Y5.01514 Z5.60763

N20260 X-2.03832 Y5.01514 Z5.61619

N20270 X-2.12557 Y5.01514 Z5.6333

N20280 X-2.21475 Y5.01514 Z5.65505

N20290 X-2.23624 Y5.01514 Z5.65979

N20300 X-2.27948 Y5.01514 Z5.67111

N20310 X-2.33515 Y5.01514 Z5.68308

N20320 X-2.36239 Y5.01514 Z5.69002

N20330 X-2.43445 Y5.01514 Z5.70507

N20340 X-2.53965 Y5.01514 Z5.73083

N20350 X-2.66498 Y5.01514 Z5.75258

N20360 X-2.77264 Y5.01514 Z5.77171

N20370 X-2.80301 Y5.01514 Z5.77616

N20380 X-2.83241 Y5.01514 Z5.78086

N20390 X-2.95421 Y5.01514 Z5.79494

N20400 X-3.05369 Y5.01514 Z5.80883

N20410 X-3.10085 Y5.01514 Z5.81444

N20420 X-3.22784 Y5.01514 Z5.82831

N20430 X-3.25613 Y5.01514 Z5.83259

N20440 X-3.36118 Y5.01514 Z5.83931

N20450 X-3.44931 Y5.01514 Z5.85049

N20460 X-3.48269 Y5.01514 Z5.85054

N20470 X-3.51628 Y5.01514 Z5.8528

N20480 X-3.59573 Y5.01514 Z5.85471

N20490 X-3.67447 Y5.01514 Z5.84963

N20500 X-3.72342 Y5.01514 Z5.84874

N20510 X-3.81604 Y5.01514 Z5.83644

N20520 X-3.84555 Y5.01514 Z5.83334

N20530 X-3.96419 Y5.01514 Z5.81216

N20540 X-4.06204 Y5.01514 Z5.78742

N20550 X-4.0955 Y5.01514 Z5.77803

N20560 X-4.16376 Y5.01514 Z5.75881

N20570 X-4.24449 Y5.01514 Z5.73449

N20580 X-4.29844 Y5.01514 Z5.71729

N20590 X-4.36267 Y5.01514 Z5.6891

N20600 X-4.42231 Y5.01514 Z5.66181

N20610 X-4.4624 Y5.01514 Z5.64271

N20620 X-4.49568 Y5.01514 Z5.62647

N20630 X-4.56349 Y5.01514 Z5.58163

N20640 X-4.62882 Y5.01514 Z5.53899

N20650 X-4.66659 Y5.01514 Z5.50697

N20660 X-4.74409 Y5.01514 Z5.44562

N20670 X-4.76941 Y5.01514 Z5.41998

N20680 X-4.81609 Y5.01514 Z5.37993

N20690 X-4.85655 Y5.01514 Z5.34378

N20700 X-4.88708 Y5.01514 Z5.31572

N20710 X-4.93386 Y5.01514 Z5.26565

N20720 X-4.99372 Y5.01514 Z5.20103

N20730 X-5.00498 Y5.01514 Z5.18663

N20740 X-5.03618 Y5.01514 Z5.14226

N20750 X-5.06909 Y5.01514 Z5.0949

N20760 X-5.17979 Y5.01514 Z4.86508

N20770 X-5.19522 Y5.01514 Z4.83565

N20780 X-5.23699 Y5.01514 Z4.75296

N20790 X-5.32817 Y4.94569 Z4.73983

N20800 X-5.30435 Y4.94569 Z4.78862

N20810 X-5.27925 Y4.94569 Z4.83763

N20820 X-5.24779 Y4.94569 Z4.90553

N20830 X-5.11626 Y4.94569 Z5.15418

N20840 X-5.03806 Y4.94569 Z5.25471

N20850 X-5.00448 Y4.94569 Z5.29177

N20860 X-4.96372 Y4.94569 Z5.33242

N20870 X-4.8849 Y4.94569 Z5.40562

N20880 X-4.85911 Y4.94569 Z5.42867

N20890 X-4.79596 Y4.94569 Z5.47842

N20900 X-4.76598 Y4.94569 Z5.49947

N20910 X-4.68622 Y4.94569 Z5.56334

N20920 X-4.65586 Y4.94569 Z5.58222

N20930 X-4.56871 Y4.94569 Z5.64053

N20940 X-4.55179 Y4.94569 Z5.64903

N20950 X-4.51455 Y4.94569 Z5.66784

N20960 X-4.45067 Y4.94569 Z5.69903

N20970 X-4.41062 Y4.94569 Z5.7181

N20980 X-4.35597 Y4.94569 Z5.73684

N20990 X-4.31804 Y4.94569 Z5.74957

N21000 X-4.26241 Y4.94569 Z5.76756

N21010 X-4.16999 Y4.94569 Z5.79628

N21020 X-4.0695 Y4.94569 Z5.81817

N21030 X-4.02928 Y4.94569 Z5.82595

N21040 X-3.96646 Y4.94569 Z5.83863

N21050 X-3.8929 Y4.94569 Z5.8504

N21060 X-3.85562 Y4.94569 Z5.85701

N21070 X-3.7506 Y4.94569 Z5.86807

N21080 X-3.73061 Y4.94569 Z5.87072

N21090 X-3.60177 Y4.94569 Z5.87306

N21100 X-3.59001 Y4.94569 Z5.87382

N21110 X-3.56292 Y4.94569 Z5.87317

N21120 X-3.46752 Y4.94569 Z5.86409

N21130 X-3.36656 Y4.94569 Z5.85507

N21140 X-3.33391 Y4.94569 Z5.85093

N21150 X-3.31456 Y4.94569 Z5.84969

N21160 X-3.18392 Y4.94569 Z5.82995

N21170 X-3.12735 Y4.94569 Z5.82377

N21180 X-2.98648 Y4.94569 Z5.80701

N21190 X-2.96167 Y4.94569 Z5.80317

N21200 X-2.94015 Y4.94569 Z5.80015

N21210 X-2.80944 Y4.94569 Z5.77926

N21220 X-2.69901 Y4.94569 Z5.76308

N21230 X-2.66952 Y4.94569 Z5.75761

N21240 X-2.64678 Y4.94569 Z5.75321

N21250 X-2.54929 Y4.94569 Z5.73183

N21260 X-2.45367 Y4.94569 Z5.7129

N21270 X-2.43954 Y4.94569 Z5.70943

N21280 X-2.42084 Y4.94569 Z5.70553

N21290 X-2.34444 Y4.94569 Z5.68592

N21300 X-2.29602 Y4.94569 Z5.6745

N21310 X-2.24715 Y4.94569 Z5.66171

N21320 X-2.14359 Y4.94569 Z5.63886

N21330 X-2.13375 Y4.94569 Z5.63646

N21340 X-2.11254 Y4.94569 Z5.63231

N21350 X-2.01456 Y4.94569 Z5.60836

N21360 X-1.96457 Y4.94569 Z5.59847

N21370 X-1.88823 Y4.94569 Z5.58182

N21380 X-1.79992 Y4.94569 Z5.56791

N21390 X-1.78533 Y4.94569 Z5.5654

N21400 X-1.69497 Y4.94569 Z5.54653

N21410 X-1.61527 Y4.94569 Z5.53603

N21420 X-1.56883 Y4.94569 Z5.52709

N21430 X-1.50897 Y4.94569 Z5.52112

N21440 X-1.4461 Y4.94569 Z5.51111

N21450 X-1.41232 Y4.94569 Z5.50974

N21460 X-1.33253 Y4.94569 Z5.50239

N21470 X-1.2676 Y4.94569 Z5.50257

N21480 X-1.22051 Y4.94569 Z5.50121

N21490 X-1.14333 Y4.94569 Z5.50219

N21500 X-1.10237 Y4.94569 Z5.50331

N21510 X-1.03869 Y4.94569 Z5.50422

N21520 X-0.984118 Y4.94569 Z5.5061

N21530 X-0.938177 Y4.94569 Z5.5078

N21540 X-0.885264 Y4.94569 Z5.51076

N21550 X-0.849038 Y4.94569 Z5.51309

N21560 X-0.798144 Y4.94569 Z5.51798

N21570 X-0.771085 Y4.94569 Z5.5205

N21580 X-0.715178 Y4.94569 Z5.52798

N21590 X-0.699002 Y4.94569 Z5.52992

N21600 X-0.634888 Y4.94569 Z5.54048

N21610 X-0.608401 Y4.94569 Z5.54555

N21620 X-0.563547 Y4.94569 Z5.55374

N21630 X-0.49244 Y4.94569 Z5.56972

N21640 X-0.471122 Y4.94569 Z5.57563

N21650 X-0.409295 Y4.94569 Z5.59169

N21660 X-0.386486 Y4.94569 Z5.59846

N21670 X-0.342659 Y4.94569 Z5.61297

N21680 X-0.311837 Y4.94569 Z5.62173

N21690 X-0.291854 Y4.94569 Z5.62846

N21700 X-0.250481 Y4.94569 Z5.64238

N21710 X-0.205806 Y4.94569 Z5.65972

N21720 X-0.191901 Y4.94569 Z5.66543

N21730 X-0.134589 Y4.94569 Z5.69062

N21740 X-0.0293502 Y4.94569 Z5.74212

N21750 X0.0899574 Y4.94569 Z5.80275

N21760 X0.131333 Y4.94569 Z5.82559

N21770 X0.15741 Y4.94569 Z5.83921

N21780 X0.22401 Y4.94569 Z5.87537

N21790 X0.234986 Y4.94569 Z5.88086

N21800 X0.280944 Y4.94569 Z5.90564

N21810 X0.295125 Y4.94569 Z5.913

N21820 X0.35366 Y4.94569 Z5.9403

N21830 X0.38991 Y4.94569 Z5.95756

N21840 X0.424766 Y4.94569 Z5.97208

N21850 X0.459635 Y4.94569 Z5.9883

N21860 X0.509178 Y4.94569 Z6.00957

N21870 X0.554409 Y4.94569 Z6.02922

N21880 X0.586146 Y4.94569 Z6.04216

N21890 X0.647206 Y4.94569 Z6.06785

N21900 X0.659903 Y4.94569 Z6.07324

N21910 X0.736795 Y4.94569 Z6.1052

N21920 X0.768219 Y4.94569 Z6.11789

N21930 X0.835098 Y4.94569 Z6.14327

N21940 X0.8478 Y4.94569 Z6.14767

N21950 X0.900509 Y4.94569 Z6.16727

N21960 X0.941551 Y4.94569 Z6.18169

N21970 X0.951053 Y4.94569 Z6.18508

N21980 X1.05693 Y4.94569 Z6.21376

N21990 X1.14429 Y4.94569 Z6.22738

N22000 X1.18647 Y4.94569 Z6.23328

N22010 X1.22666 Y4.94569 Z6.23731

N22020 X1.2891 Y4.94569 Z6.24371

N22030 X1.30688 Y4.94569 Z6.24555

N22040 X1.37258 Y4.94569 Z6.25078

N22050 X1.38918 Y4.94569 Z6.25278

N22060 X1.45338 Y4.94569 Z6.25795

N22070 X1.47086 Y4.94569 Z6.25999

N22080 X1.53823 Y4.94569 Z6.26655

N22090 X1.54932 Y4.94569 Z6.26777

N22100 X1.59646 Y4.94569 Z6.27328

N22110 X1.62442 Y4.94569 Z6.27536

N22120 X1.69732 Y4.94569 Z6.28072

N22130 X1.74992 Y4.94569 Z6.287

N22140 X1.76675 Y4.94569 Z6.28822

N22150 X1.79059 Y4.94569 Z6.29059

N22160 X1.83918 Y4.94569 Z6.29288

N22170 X1.87746 Y4.94569 Z6.29438

N22180 X1.91505 Y4.94569 Z6.29532

N22190 X1.96624 Y4.94569 Z6.29733

N22200 X1.99454 Y4.94569 Z6.29631

N22210 X2.01841 Y4.94569 Z6.29592

N22220 X2.0838 Y4.94569 Z6.29432

N22230 X2.17191 Y4.94569 Z6.29404

N22240 X2.18272 Y4.94569 Z6.29301

N22250 X2.27853 Y4.94569 Z6.28975

N22260 X2.37403 Y4.94569 Z6.28268

N22270 X2.40248 Y4.94569 Z6.28124

N22280 X2.45507 Y4.94569 Z6.27443

N22290 X2.5169 Y4.94569 Z6.26895

N22300 X2.53601 Y4.94569 Z6.26703

N22310 X2.55393 Y4.94569 Z6.26612

N22320 X2.62174 Y4.94569 Z6.25694

N22330 X2.66991 Y4.94569 Z6.25165

N22340 X2.73261 Y4.94569 Z6.24058

N22350 X2.77738 Y4.94569 Z6.23413

N22360 X2.86036 Y4.94569 Z6.21699

N22370 X2.87671 Y4.94569 Z6.21393

N22380 X2.91549 Y4.94569 Z6.20348

N22390 X2.96756 Y4.94569 Z6.18868

N22400 X2.99102 Y4.94569 Z6.17991

N22410 X3.04956 Y4.94569 Z6.15771

N22420 X3.12634 Y4.94569 Z6.12476

N22430 X3.13822 Y4.94569 Z6.11941

N22440 X3.15415 Y4.94569 Z6.11167

N22450 X3.22432 Y4.94569 Z6.07321

N22460 X3.30891 Y4.94569 Z6.02247

N22470 X3.32722 Y4.94569 Z6.00959

N22480 X3.39676 Y4.94569 Z5.95746

N22490 X3.42447 Y4.94569 Z5.93455

N22500 X3.47009 Y4.94569 Z5.89524

N22510 X3.51953 Y4.94569 Z5.84771

N22520 X3.53327 Y4.94569 Z5.83347

N22530 X3.5564 Y4.94569 Z5.80887

N22540 X3.59451 Y4.94569 Z5.76619

N22550 X3.6197 Y4.94569 Z5.73549

N22560 X3.65622 Y4.94569 Z5.68893

N22570 X3.6996 Y4.94569 Z5.62639

N22580 X3.71624 Y4.94569 Z5.60168

N22590 X3.73356 Y4.94569 Z5.5737

N22600 X3.77323 Y4.94569 Z5.5049

N22610 X3.8054 Y4.94569 Z5.44719

N22620 X3.83542 Y4.94569 Z5.39158

N22630 X3.87291 Y4.94569 Z5.31639

N22640 X3.90418 Y4.94569 Z5.25

N22650 X3.93701 Y4.94569 Z5.19364

N22660 X4.00517 Y4.94569 Z5.06992

N22670 X4.13738 Y4.87116 Z5.0572

N22680 X4.02893 Y4.87116 Z5.2534

N22690 X3.9827 Y4.87116 Z5.34623

N22700 X3.93726 Y4.87116 Z5.44009

N22710 X3.90917 Y4.87116 Z5.4906

N22720 X3.86807 Y4.87116 Z5.56221

N22730 X3.81611 Y4.87116 Z5.64906

N22740 X3.80374 Y4.87116 Z5.66832

N22750 X3.78546 Y4.87116 Z5.69417

N22760 X3.73914 Y4.87116 Z5.75867

N22770 X3.7104 Y4.87116 Z5.79467

N22780 X3.67444 Y4.87116 Z5.83729

N22790 X3.60713 Y4.87116 Z5.91046

N22800 X3.53352 Y4.87116 Z5.97917

N22810 X3.49117 Y4.87116 Z6.01511

N22820 X3.45404 Y4.87116 Z6.04467

N22830 X3.39775 Y4.87116 Z6.08542

N22840 X3.36993 Y4.87116 Z6.10486

N22850 X3.3511 Y4.87116 Z6.11676

N22860 X3.27919 Y4.87116 Z6.15763

N22870 X3.21247 Y4.87116 Z6.19202

N22880 X3.19123 Y4.87116 Z6.20233

N22890 X3.16832 Y4.87116 Z6.21265

N22900 X3.10166 Y4.87116 Z6.23835

N22910 X3.05204 Y4.87116 Z6.25557

N22920 X3.01349 Y4.87116 Z6.2684

N22930 X2.97457 Y4.87116 Z6.27949

N22940 X2.9276 Y4.87116 Z6.29017

N22950 X2.85522 Y4.87116 Z6.30362

N22960 X2.8205 Y4.87116 Z6.3076

N22970 X2.73714 Y4.87116 Z6.31875

N22980 X2.68626 Y4.87116 Z6.32163

N22990 X2.62695 Y4.87116 Z6.3276

N23000 X2.56 Y4.87116 Z6.33087

N23010 X2.53858 Y4.87116 Z6.33197

N23020 X2.51478 Y4.87116 Z6.33435

N23030 X2.45475 Y4.87116 Z6.33543

N23040 X2.38072 Y4.87116 Z6.34045

N23050 X2.36624 Y4.87116 Z6.34133

N23060 X2.28233 Y4.87116 Z6.3419

N23070 X2.24876 Y4.87116 Z6.34322

N23080 X2.18782 Y4.87116 Z6.34024

N23090 X2.09924 Y4.87116 Z6.34085

N23100 X2.07984 Y4.87116 Z6.34113

N23110 X1.99494 Y4.87116 Z6.33582

N23120 X1.90806 Y4.87116 Z6.33395

N23130 X1.8366 Y4.87116 Z6.32834

N23140 X1.78939 Y4.87116 Z6.32485

N23150 X1.76554 Y4.87116 Z6.32248

N23160 X1.73394 Y4.87116 Z6.32019

N23170 X1.69757 Y4.87116 Z6.31547

N23180 X1.67802 Y4.87116 Z6.31365

N23190 X1.6294 Y4.87116 Z6.30733

N23200 X1.58168 Y4.87116 Z6.30258

N23210 X1.55846 Y4.87116 Z6.29986

N23220 X1.49662 Y4.87116 Z6.29308

N23230 X1.48484 Y4.87116 Z6.29193

N23240 X1.41507 Y4.87116 Z6.28379

N23250 X1.40482 Y4.87116 Z6.28296

N23260 X1.33079 Y4.87116 Z6.27406

N23270 X1.31783 Y4.87116 Z6.27303

N23280 X1.2384 Y4.87116 Z6.26484

N23290 X1.22738 Y4.87116 Z6.26373

N23300 X1.15074 Y4.87116 Z6.25258

N23310 X1.11038 Y4.87116 Z6.24633

N23320 X1.06285 Y4.87116 Z6.23715

N23330 X0.982106 Y4.87116 Z6.21898

N23340 X0.969799 Y4.87116 Z6.21492

N23350 X0.904826 Y4.87116 Z6.19171

N23360 X0.879519 Y4.87116 Z6.18283

N23370 X0.828153 Y4.87116 Z6.16129

N23380 X0.774697 Y4.87116 Z6.14074

N23390 X0.747959 Y4.87116 Z6.13002

N23400 X0.680478 Y4.87116 Z6.1015

N23410 X0.665425 Y4.87116 Z6.09524

N23420 X0.583435 Y4.87116 Z6.06046

N23430 X0.561985 Y4.87116 Z6.05171

N23440 X0.501954 Y4.87116 Z6.02469

N23450 X0.461539 Y4.87116 Z6.00805

N23460 X0.423186 Y4.87116 Z5.9902

N23470 X0.399741 Y4.87116 Z5.98044

N23480 X0.350003 Y4.87116 Z5.95693

N23490 X0.288232 Y4.87116 Z5.9269

N23500 X0.269127 Y4.87116 Z5.91714

N23510 X0.199647 Y4.87116 Z5.87867

N23520 X0.179984 Y4.87116 Z5.86792

N23530 X0.0929762 Y4.87116 Z5.8199

N23540 X-0.0859639 Y4.87116 Z5.72451

N23550 X-0.177259 Y4.87116 Z5.67798

N23560 X-0.190067 Y4.87116 Z5.67235

N23570 X-0.226935 Y4.87116 Z5.6572

N23580 X-0.261631 Y4.87116 Z5.64118

N23590 X-0.27973 Y4.87116 Z5.63416

N23600 X-0.321156 Y4.87116 Z5.61814

N23610 X-0.382575 Y4.87116 Z5.59783

N23620 X-0.442814 Y4.87116 Z5.57879

N23630 X-0.457365 Y4.87116 Z5.57414

N23640 X-0.526926 Y4.87116 Z5.55531

N23650 X-0.561607 Y4.87116 Z5.5474

N23660 X-0.600437 Y4.87116 Z5.53839

N23670 X-0.659551 Y4.87116 Z5.52625

N23680 X-0.682484 Y4.87116 Z5.52286

N23690 X-0.727953 Y4.87116 Z5.51479

N23700 X-0.768617 Y4.87116 Z5.51006

N23710 X-0.799482 Y4.87116 Z5.50545

N23720 X-0.85546 Y4.87116 Z5.50081

N23730 X-0.878026 Y4.87116 Z5.49886

N23740 X-0.946683 Y4.87116 Z5.49532

N23750 X-0.970749 Y4.87116 Z5.49445

N23760 X-1.05636 Y4.87116 Z5.49244

N23770 X-1.07369 Y4.87116 Z5.49194

N23780 X-1.13072 Y4.87116 Z5.49203

N23790 X-1.18671 Y4.87116 Z5.4905

N23800 X-1.20131 Y4.87116 Z5.49031

N23810 X-1.33005 Y4.87116 Z5.49392

N23820 X-1.34131 Y4.87116 Z5.49496

N23830 X-1.44411 Y4.87116 Z5.49926

N23840 X-1.49151 Y4.87116 Z5.50737

N23850 X-1.56695 Y4.87116 Z5.5149

N23860 X-1.65988 Y4.87116 Z5.53279

N23870 X-1.68569 Y4.87116 Z5.53653

N23880 X-1.7067 Y4.87116 Z5.541

N23890 X-1.79607 Y4.87116 Z5.55571

N23900 X-1.88692 Y4.87116 Z5.57486

N23910 X-1.90772 Y4.87116 Z5.57874

N23920 X-1.94433 Y4.87116 Z5.58761

N23930 X-2.03084 Y4.87116 Z5.60472

N23940 X-2.07079 Y4.87116 Z5.61449

N23950 X-2.15904 Y4.87116 Z5.63467

N23960 X-2.23753 Y4.87116 Z5.6553

N23970 X-2.27176 Y4.87116 Z5.66354

N23980 X-2.35992 Y4.87116 Z5.68691

N23990 X-2.45429 Y4.87116 Z5.70625

N24000 X-2.55708 Y4.87116 Z5.73181

N24010 X-2.57105 Y4.87116 Z5.7349

N24020 X-2.68402 Y4.87116 Z5.75676

N24030 X-2.7732 Y4.87116 Z5.77331

N24040 X-2.81631 Y4.87116 Z5.78041

N24050 X-2.85059 Y4.87116 Z5.78662

N24060 X-2.97531 Y4.87116 Z5.80407

N24070 X-3.11246 Y4.87116 Z5.82535

N24080 X-3.15011 Y4.87116 Z5.83086

N24090 X-3.17426 Y4.87116 Z5.8353

N24100 X-3.31601 Y4.87116 Z5.85403

N24110 X-3.40096 Y4.87116 Z5.86742

N24120 X-3.46002 Y4.87116 Z5.8727

N24130 X-3.51074 Y4.87116 Z5.87753

N24140 X-3.60206 Y4.87116 Z5.88605

N24150 X-3.67869 Y4.87116 Z5.8853

N24160 X-3.74364 Y4.87116 Z5.88633

N24170 X-3.82737 Y4.87116 Z5.87933

N24180 X-3.8675 Y4.87116 Z5.87713

N24190 X-3.96158 Y4.87116 Z5.86513

N24200 X-3.97629 Y4.87116 Z5.86366

N24210 X-4.0249 Y4.87116 Z5.85627

N24220 X-4.07591 Y4.87116 Z5.8464

N24230 X-4.09294 Y4.87116 Z5.8427

N24240 X-4.16813 Y4.87116 Z5.82599

N24250 X-4.2294 Y4.87116 Z5.81194

N24260 X-4.25742 Y4.87116 Z5.80522

N24270 X-4.30807 Y4.87116 Z5.79322

N24280 X-4.34431 Y4.87116 Z5.78106

N24290 X-4.37197 Y4.87116 Z5.77158

N24300 X-4.43141 Y4.87116 Z5.75066

N24310 X-4.50016 Y4.87116 Z5.72599

N24320 X-4.52712 Y4.87116 Z5.71237

N24330 X-4.63194 Y4.87116 Z5.65852

N24340 X-4.7356 Y4.87116 Z5.59406

N24350 X-4.74631 Y4.87116 Z5.58549

N24360 X-4.8369 Y4.87116 Z5.52188

N24370 X-4.86396 Y4.87116 Z5.50057

N24380 X-4.92516 Y4.87116 Z5.4546

N24390 X-4.98023 Y4.87116 Z5.40725

N24400 X-4.99784 Y4.87116 Z5.39185

N24410 X-5.01459 Y4.87116 Z5.37684

N24420 X-5.06614 Y4.87116 Z5.32533

N24430 X-5.11584 Y4.87116 Z5.2718

N24440 X-5.13921 Y4.87116 Z5.24223

N24450 X-5.22113 Y4.87116 Z5.12814

N24460 X-5.30071 Y4.87116 Z4.9832

N24470 X-5.31298 Y4.87116 Z4.95825

N24480 X-5.33082 Y4.87116 Z4.91825

N24490 X-5.37187 Y4.87116 Z4.82654

N24500 X-5.40821 Y4.87116 Z4.75355

N24510 X-5.42165 Y4.87116 Z4.72479

N24520 X-5.51621 Y4.79203 Z4.70675

N24530 X-5.50465 Y4.79203 Z4.73147

N24540 X-5.45941 Y4.79203 Z4.82695

N24550 X-5.42284 Y4.79203 Z4.91463

N24560 X-5.40486 Y4.79203 Z4.95494

N24570 X-5.34237 Y4.79203 Z5.08194

N24580 X-5.27573 Y4.79203 Z5.18717

N24590 X-5.22297 Y4.79203 Z5.25082

N24600 X-5.19762 Y4.79203 Z5.28579

N24610 X-5.10965 Y4.79203 Z5.37747

N24620 X-5.04548 Y4.79203 Z5.43532

N24630 X-5.03233 Y4.79203 Z5.44711

N24640 X-5.01836 Y4.79203 Z5.45932

N24650 X-4.94863 Y4.79203 Z5.51329

N24660 X-4.90171 Y4.79203 Z5.54806

N24670 X-4.84024 Y4.79203 Z5.58647

N24680 X-4.78103 Y4.79203 Z5.62647

N24690 X-4.70449 Y4.79203 Z5.66852

N24700 X-4.66208 Y4.79203 Z5.69463

N24710 X-4.58616 Y4.79203 Z5.72945

N24720 X-4.54949 Y4.79203 Z5.74833

N24730 X-4.49111 Y4.79203 Z5.76989

N24740 X-4.4205 Y4.79203 Z5.7958

N24750 X-4.40781 Y4.79203 Z5.79901

N24760 X-4.32786 Y4.79203 Z5.81836

N24770 X-4.27227 Y4.79203 Z5.83194

N24780 X-4.24752 Y4.79203 Z5.8378

N24790 X-4.20517 Y4.79203 Z5.84795

N24800 X-4.16524 Y4.79203 Z5.85452

N24810 X-4.14395 Y4.79203 Z5.8582

N24820 X-4.07986 Y4.79203 Z5.86871

N24830 X-4.01713 Y4.79203 Z5.87775

N24840 X-3.98718 Y4.79203 Z5.8823

N24850 X-3.89546 Y4.79203 Z5.89148

N24860 X-3.88117 Y4.79203 Z5.8933

N24870 X-3.7612 Y4.79203 Z5.89989

N24880 X-3.62251 Y4.79203 Z5.89615

N24890 X-3.60641 Y4.79203 Z5.89453

N24900 X-3.47839 Y4.79203 Z5.88079

N24910 X-3.44622 Y4.79203 Z5.87575

N24920 X-3.33626 Y4.79203 Z5.86035

N24930 X-3.30021 Y4.79203 Z5.85467

N24940 X-3.28249 Y4.79203 Z5.85232

N24950 X-3.15638 Y4.79203 Z5.82915

N24960 X-3.01631 Y4.79203 Z5.80865

N24970 X-2.99041 Y4.79203 Z5.80377

N24980 X-2.95007 Y4.79203 Z5.79671

N24990 X-2.82352 Y4.79203 Z5.77379

N25000 X-2.75803 Y4.79203 Z5.76301

N25010 X-2.66948 Y4.79203 Z5.74456

N25020 X-2.61137 Y4.79203 Z5.73155

N25030 X-2.47717 Y4.79203 Z5.70257

N25040 X-2.46135 Y4.79203 Z5.69882

N25050 X-2.34555 Y4.79203 Z5.67022

N25060 X-2.32575 Y4.79203 Z5.66497

N25070 X-2.21936 Y4.79203 Z5.63939

N25080 X-2.19168 Y4.79203 Z5.63223

N25090 X-2.18073 Y4.79203 Z5.62951

N25100 X-2.06334 Y4.79203 Z5.5993

N25110 X-1.99596 Y4.79203 Z5.58301

N25120 X-1.92328 Y4.79203 Z5.56539

N25130 X-1.81812 Y4.79203 Z5.54577

N25140 X-1.79892 Y4.79203 Z5.54122

N25150 X-1.78113 Y4.79203 Z5.53807

N25160 X-1.68581 Y4.79203 Z5.51779

N25170 X-1.59972 Y4.79203 Z5.50531

N25180 X-1.56769 Y4.79203 Z5.49907

N25190 X-1.53863 Y4.79203 Z5.49538

N25200 X-1.4398 Y4.79203 Z5.47846

N25210 X-1.37959 Y4.79203 Z5.47594

N25220 X-1.31001 Y4.79203 Z5.47254

N25230 X-1.24737 Y4.79203 Z5.4716

N25240 X-1.19652 Y4.79203 Z5.46979

N25250 X-1.12367 Y4.79203 Z5.47213

N25260 X-1.10383 Y4.79203 Z5.4721

N25270 X-1.01902 Y4.79203 Z5.47453

N25280 X-1.00636 Y4.79203 Z5.47469

N25290 X-0.974433 Y4.79203 Z5.47606

N25300 X-0.91197 Y4.79203 Z5.47832

N25310 X-0.850462 Y4.79203 Z5.48351

N25320 X-0.828735 Y4.79203 Z5.48519

N25330 X-0.757488 Y4.79203 Z5.4935

N25340 X-0.732599 Y4.79203 Z5.49869

N25350 X-0.689402 Y4.79203 Z5.50531

N25360 X-0.641021 Y4.79203 Z5.51608

N25370 X-0.622711 Y4.79203 Z5.51952

N25380 X-0.560137 Y4.79203 Z5.53527

N25390 X-0.494742 Y4.79203 Z5.55326

N25400 X-0.477452 Y4.79203 Z5.55911

N25410 X-0.439298 Y4.79203 Z5.57092

N25420 X-0.397926 Y4.79203 Z5.58574

N25430 X-0.379915 Y4.79203 Z5.592

N25440 X-0.334974 Y4.79203 Z5.60853

N25450 X-0.280357 Y4.79203 Z5.63249

N25460 X-0.270679 Y4.79203 Z5.63659

N25470 X-0.202493 Y4.79203 Z5.66794

N25480 X-0.143584 Y4.79203 Z5.70014

N25490 X-0.10826 Y4.79203 Z5.71769

N25500 X-0.0045772 Y4.79203 Z5.77593

N25510 X0.0202808 Y4.79203 Z5.7893

N25520 X0.161347 Y4.79203 Z5.86878

N25530 X0.233349 Y4.79203 Z5.90927

N25540 X0.261229 Y4.79203 Z5.92433

N25550 X0.301588 Y4.79203 Z5.94599

N25560 X0.345758 Y4.79203 Z5.96843

N25570 X0.394108 Y4.79203 Z5.98964

N25580 X0.466752 Y4.79203 Z6.02345

N25590 X0.49294 Y4.79203 Z6.03423

N25600 X0.516979 Y4.79203 Z6.04506

N25610 X0.592224 Y4.79203 Z6.07817

N25620 X0.624546 Y4.79203 Z6.09221

N25630 X0.677908 Y4.79203 Z6.11576

N25640 X0.712065 Y4.79203 Z6.13043

N25650 X0.752717 Y4.79203 Z6.14827

N25660 X0.799553 Y4.79203 Z6.16834

N25670 X0.815755 Y4.79203 Z6.17461

N25680 X0.8317 Y4.79203 Z6.18136

N25690 X0.884037 Y4.79203 Z6.20088

N25700 X0.947281 Y4.79203 Z6.22439

N25710 X0.965675 Y4.79203 Z6.23046

N25720 X0.991966 Y4.79203 Z6.23754

N25730 X1.05787 Y4.79203 Z6.25414

N25740 X1.14237 Y4.79203 Z6.27135

N25750 X1.15515 Y4.79203 Z6.27328

N25760 X1.24096 Y4.79203 Z6.28372

N25770 X1.28155 Y4.79203 Z6.28878

N25780 X1.3375 Y4.79203 Z6.29773

N25790 X1.3742 Y4.79203 Z6.30194

N25800 X1.42455 Y4.79203 Z6.30952

N25810 X1.45544 Y4.79203 Z6.3132

N25820 X1.49875 Y4.79203 Z6.31908

N25830 X1.53445 Y4.79203 Z6.32376

N25840 X1.56806 Y4.79203 Z6.32828

N25850 X1.61832 Y4.79203 Z6.33535

N25860 X1.63451 Y4.79203 Z6.33723

N25870 X1.67049 Y4.79203 Z6.34251

N25880 X1.69966 Y4.79203 Z6.34522

N25890 X1.71493 Y4.79203 Z6.3472

N25900 X1.76498 Y4.79203 Z6.35219

N25910 X1.8143 Y4.79203 Z6.35813

N25920 X1.83183 Y4.79203 Z6.36008

N25930 X1.85828 Y4.79203 Z6.36329

N25940 X1.90009 Y4.79203 Z6.3659

N25950 X1.9927 Y4.79203 Z6.37486

N25960 X2.09698 Y4.79203 Z6.38049

N25970 X2.18984 Y4.79203 Z6.38898

N25980 X2.27924 Y4.79203 Z6.39167

N25990 X2.33082 Y4.79203 Z6.39366

N26000 X2.36633 Y4.79203 Z6.39278

N26010 X2.43145 Y4.79203 Z6.39337

N26020 X2.45103 Y4.79203 Z6.39313

N26030 X2.46652 Y4.79203 Z6.39395

N26040 X2.54068 Y4.79203 Z6.3917

N26050 X2.60945 Y4.79203 Z6.39157

N26060 X2.65588 Y4.79203 Z6.39361

N26070 X2.77957 Y4.79203 Z6.38882

N26080 X2.79354 Y4.79203 Z6.38879

N26090 X2.80982 Y4.79203 Z6.38766

N26100 X2.9232 Y4.79203 Z6.37417

N26110 X2.98083 Y4.79203 Z6.36367

N26120 X3.03253 Y4.79203 Z6.3517

N26130 X3.09194 Y4.79203 Z6.33461

N26140 X3.13042 Y4.79203 Z6.32274

N26150 X3.17094 Y4.79203 Z6.30903

N26160 X3.22347 Y4.79203 Z6.28766

N26170 X3.26898 Y4.79203 Z6.26739

N26180 X3.31891 Y4.79203 Z6.24433

N26190 X3.38057 Y4.79203 Z6.21227

N26200 X3.41369 Y4.79203 Z6.1927

N26210 X3.45069 Y4.79203 Z6.1684

N26220 X3.50356 Y4.79203 Z6.13271

N26230 X3.54592 Y4.79203 Z6.10126

N26240 X3.58792 Y4.79203 Z6.06697

N26250 X3.65163 Y4.79203 Z6.01058

N26260 X3.66743 Y4.79203 Z5.99628

N26270 X3.67709 Y4.79203 Z5.98698

N26280 X3.73929 Y4.79203 Z5.92184

N26290 X3.78586 Y4.79203 Z5.86974

N26300 X3.80862 Y4.79203 Z5.84233

N26310 X3.85707 Y4.79203 Z5.77892

N26320 X3.87726 Y4.79203 Z5.75219

N26330 X3.88504 Y4.79203 Z5.74142

N26340 X3.94859 Y4.79203 Z5.64172

N26350 X3.97841 Y4.79203 Z5.59344

N26360 X4.03865 Y4.79203 Z5.48543

N26370 X4.06585 Y4.79203 Z5.43504

N26380 X4.1372 Y4.79203 Z5.29229

N26390 X4.16289 Y4.79203 Z5.24062

N26400 X4.27461 Y4.79203 Z5.03598

N26410 X4.41556 Y4.70926 Z5.00565

N26420 X4.32719 Y4.70926 Z5.17176

N26430 X4.30394 Y4.70926 Z5.21432

N26440 X4.29601 Y4.70926 Z5.22885

N26450 X4.24703 Y4.70926 Z5.32735

N26460 X4.19324 Y4.70926 Z5.43414

N26470 X4.18484 Y4.70926 Z5.45001

N26480 X4.10886 Y4.70926 Z5.58972

N26490 X4.08945 Y4.70926 Z5.62228

N26500 X4.02807 Y4.70926 Z5.72179

N26510 X4.00955 Y4.70926 Z5.74803

N26520 X3.93806 Y4.70926 Z5.84768

N26530 X3.92524 Y4.70926 Z5.86316

N26540 X3.86582 Y4.70926 Z5.93415

N26550 X3.84535 Y4.70926 Z5.95737

N26560 X3.79078 Y4.70926 Z6.01341

N26570 X3.72898 Y4.70926 Z6.07393

N26580 X3.71397 Y4.70926 Z6.08708

N26590 X3.6923 Y4.70926 Z6.1052

N26600 X3.63022 Y4.70926 Z6.15602

N26610 X3.58262 Y4.70926 Z6.19225

N26620 X3.54115 Y4.70926 Z6.22042

N26630 X3.49314 Y4.70926 Z6.24993

N26640 X3.44504 Y4.70926 Z6.27884

N26650 X3.39298 Y4.70926 Z6.30648

N26660 X3.34415 Y4.70926 Z6.32928

N26670 X3.30829 Y4.70926 Z6.3435

N26680 X3.24035 Y4.70926 Z6.37032

N26690 X3.15089 Y4.70926 Z6.4021

N26700 X3.13428 Y4.70926 Z6.40681

N26710 X3.03838 Y4.70926 Z6.43082

N26720 X2.97174 Y4.70926 Z6.44388

N26730 X2.92298 Y4.70926 Z6.45125

N26740 X2.8489 Y4.70926 Z6.45717

N26750 X2.80515 Y4.70926 Z6.46022

N26760 X2.74284 Y4.70926 Z6.46033

N26770 X2.68082 Y4.70926 Z6.45831

N26780 X2.66343 Y4.70926 Z6.45627

N26790 X2.55004 Y4.70926 Z6.44975

N26800 X2.52511 Y4.70926 Z6.44648

N26810 X2.47054 Y4.70926 Z6.44507

N26820 X2.43272 Y4.70926 Z6.44307

N26830 X2.41455 Y4.70926 Z6.44329

N26840 X2.34647 Y4.70926 Z6.4387

N26850 X2.29499 Y4.70926 Z6.43782

N26860 X2.25709 Y4.70926 Z6.4342

N26870 X2.2052 Y4.70926 Z6.42876

N26880 X2.11992 Y4.70926 Z6.42201

N26890 X2.10552 Y4.70926 Z6.41985

N26900 X2.09173 Y4.70926 Z6.41823

N26910 X2.00429 Y4.70926 Z6.4066

N26920 X1.94485 Y4.70926 Z6.40058

N26930 X1.92478 Y4.70926 Z6.399

N26940 X1.87613 Y4.70926 Z6.39093

N26950 X1.82036 Y4.70926 Z6.38591

N26960 X1.76686 Y4.70926 Z6.37795

N26970 X1.74289 Y4.70926 Z6.37479

N26980 X1.70112 Y4.70926 Z6.36848

N26990 X1.65085 Y4.70926 Z6.36294

N27000 X1.63997 Y4.70926 Z6.36135

N27010 X1.61457 Y4.70926 Z6.3584

N27020 X1.57792 Y4.70926 Z6.35266

N27030 X1.56455 Y4.70926 Z6.3506

N27040 X1.51376 Y4.70926 Z6.34276

N27050 X1.48917 Y4.70926 Z6.33891

N27060 X1.44503 Y4.70926 Z6.33238

N27070 X1.41369 Y4.70926 Z6.32748

N27080 X1.36814 Y4.70926 Z6.32091

N27090 X1.33916 Y4.70926 Z6.31503

N27100 X1.26113 Y4.70926 Z6.3034

N27110 X1.2384 Y4.70926 Z6.2988

N27120 X1.19406 Y4.70926 Z6.29198

N27130 X1.13784 Y4.70926 Z6.28227

N27140 X1.07558 Y4.70926 Z6.27034

N27150 X1.04217 Y4.70926 Z6.26244

N27160 X1.01317 Y4.70926 Z6.25434

N27170 X0.949561 Y4.70926 Z6.23556

N27180 X0.905518 Y4.70926 Z6.22047

N27190 X0.869345 Y4.70926 Z6.20674

N27200 X0.823711 Y4.70926 Z6.18915

N27210 X0.801564 Y4.70926 Z6.17978

N27220 X0.788339 Y4.70926 Z6.17466

N27230 X0.737152 Y4.70926 Z6.15236

N27240 X0.702511 Y4.70926 Z6.13717

N27250 X0.661084 Y4.70926 Z6.11872

N27260 X0.612878 Y4.70926 Z6.09744

N27270 X0.566795 Y4.70926 Z6.07675

N27280 X0.508884 Y4.70926 Z6.05081

N27290 X0.448953 Y4.70926 Z6.02278

N27300 X0.377024 Y4.70926 Z5.9903

N27310 X0.325085 Y4.70926 Z5.96357

N27320 X0.254878 Y4.70926 Z5.927

N27330 X0.199031 Y4.70926 Z5.89591

N27340 X0.121944 Y4.70926 Z5.85202

N27350 X0.0617303 Y4.70926 Z5.81717

N27360 X-0.0170425 Y4.70926 Z5.77088

N27370 X-0.070854 Y4.70926 Z5.74117

N27380 X-0.1293 Y4.70926 Z5.70697

N27390 X-0.219798 Y4.70926 Z5.65758

N27400 X-0.261436 Y4.70926 Z5.63649

N27410 X-0.294412 Y4.70926 Z5.61958

N27420 X-0.317588 Y4.70926 Z5.6095

N27430 X-0.354604 Y4.70926 Z5.59146

N27440 X-0.390762 Y4.70926 Z5.57698

N27450 X-0.408582 Y4.70926 Z5.56959

N27460 X-0.422423 Y4.70926 Z5.56481

N27470 X-0.463259 Y4.70926 Z5.54888

N27480 X-0.507717 Y4.70926 Z5.53455

N27490 X-0.522772 Y4.70926 Z5.52913

N27500 X-0.571965 Y4.70926 Z5.5149

N27510 X-0.590577 Y4.70926 Z5.50955

N27520 X-0.651607 Y4.70926 Z5.49301

N27530 X-0.682027 Y4.70926 Z5.48685

N27540 X-0.719311 Y4.70926 Z5.47742

N27550 X-0.77507 Y4.70926 Z5.46766

N27560 X-0.793079 Y4.70926 Z5.46446

N27570 X-0.863024 Y4.70926 Z5.45302

N27580 X-0.893029 Y4.70926 Z5.4507

N27590 X-0.954225 Y4.70926 Z5.44782

N27600 X-0.971317 Y4.70926 Z5.44708

N27610 X-1.04519 Y4.70926 Z5.4448

N27620 X-1.05937 Y4.70926 Z5.44463

N27630 X-1.12223 Y4.70926 Z5.44339

N27640 X-1.1708 Y4.70926 Z5.44422

N27650 X-1.19413 Y4.70926 Z5.44389

N27660 X-1.20597 Y4.70926 Z5.44442

N27670 X-1.28337 Y4.70926 Z5.4462

N27680 X-1.30194 Y4.70926 Z5.44674

N27690 X-1.43706 Y4.70926 Z5.45701

N27700 X-1.5759 Y4.70926 Z5.47461

N27710 X-1.6479 Y4.70926 Z5.48864

N27720 X-1.68502 Y4.70926 Z5.49457

N27730 X-1.72412 Y4.70926 Z5.50324

N27740 X-1.7974 Y4.70926 Z5.5162

N27750 X-1.9 Y4.70926 Z5.54055

N27760 X-1.93256 Y4.70926 Z5.54771

N27770 X-1.95278 Y4.70926 Z5.55314

N27780 X-2.07929 Y4.70926 Z5.58521

N27790 X-2.10911 Y4.70926 Z5.59242

N27800 X-2.26759 Y4.70926 Z5.63182

N27810 X-2.31723 Y4.70926 Z5.64466

N27820 X-2.4408 Y4.70926 Z5.67532

N27830 X-2.51003 Y4.70926 Z5.69161

N27840 X-2.60793 Y4.70926 Z5.71514

N27850 X-2.68357 Y4.70926 Z5.73296

N27860 X-2.82962 Y4.70926 Z5.76321

N27870 X-2.90204 Y4.70926 Z5.77867

N27880 X-3.0092 Y4.70926 Z5.79742

N27890 X-3.13882 Y4.70926 Z5.82185

N27900 X-3.16516 Y4.70926 Z5.82674

N27910 X-3.18075 Y4.70926 Z5.82985

N27920 X-3.30157 Y4.70926 Z5.84996

N27930 X-3.38301 Y4.70926 Z5.86525

N27940 X-3.42416 Y4.70926 Z5.87101

N27950 X-3.53198 Y4.70926 Z5.88788

N27960 X-3.55293 Y4.70926 Z5.89013

N27970 X-3.66239 Y4.70926 Z5.90114

N27980 X-3.69949 Y4.70926 Z5.90218

N27990 X-3.78908 Y4.70926 Z5.90573

N28000 X-3.83594 Y4.70926 Z5.90387

N28010 X-3.90234 Y4.70926 Z5.90317

N28020 X-3.9516 Y4.70926 Z5.89901

N28030 X-3.99905 Y4.70926 Z5.89678

N28040 X-4.05507 Y4.70926 Z5.89063

N28050 X-4.07834 Y4.70926 Z5.88792

N28060 X-4.12111 Y4.70926 Z5.88329

N28070 X-4.15578 Y4.70926 Z5.8773

N28080 X-4.177 Y4.70926 Z5.87381

N28090 X-4.23163 Y4.70926 Z5.86463

N28100 X-4.29505 Y4.70926 Z5.8542

N28110 X-4.30883 Y4.70926 Z5.852

N28120 X-4.33535 Y4.70926 Z5.84738

N28130 X-4.38677 Y4.70926 Z5.8348

N28140 X-4.41593 Y4.70926 Z5.82742

N28150 X-4.46981 Y4.70926 Z5.81389

N28160 X-4.51655 Y4.70926 Z5.79614

N28170 X-4.57165 Y4.70926 Z5.77954

N28180 X-4.63605 Y4.70926 Z5.75061

N28190 X-4.6902 Y4.70926 Z5.73016

N28200 X-4.81332 Y4.70926 Z5.66665

N28210 X-4.82412 Y4.70926 Z5.66164

N28220 X-4.83414 Y4.70926 Z5.65626

N28230 X-4.95603 Y4.70926 Z5.57989

N28240 X-5.0083 Y4.70926 Z5.54342

N28250 X-5.0696 Y4.70926 Z5.49639

N28260 X-5.11221 Y4.70926 Z5.45919

N28270 X-5.17722 Y4.70926 Z5.40481

N28280 X-5.21692 Y4.70926 Z5.36417

N28290 X-5.27532 Y4.70926 Z5.30582

N28300 X-5.30199 Y4.70926 Z5.27369

N28310 X-5.36185 Y4.70926 Z5.19805

N28320 X-5.43595 Y4.70926 Z5.07588

N28330 X-5.44994 Y4.70926 Z5.04777

N28340 X-5.49357 Y4.70926 Z4.95502

N28350 X-5.53798 Y4.70926 Z4.85067

N28360 X-5.54918 Y4.70926 Z4.82291

N28370 X-5.61052 Y4.70926 Z4.6854

N28380 X-5.70164 Y4.62638 Z4.66062

N28390 X-5.69413 Y4.62638 Z4.67885

N28400 X-5.64775 Y4.62638 Z4.78949

N28410 X-5.63222 Y4.62638 Z4.82426

N28420 X-5.61052 Y4.62638 Z4.87806

N28430 X-5.47352 Y4.62638 Z5.15963

N28440 X-5.45327 Y4.62638 Z5.19324

N28450 X-5.39234 Y4.62638 Z5.27354

N28460 X-5.36578 Y4.62638 Z5.30705

N28470 X-5.30481 Y4.62638 Z5.37171

N28480 X-5.26232 Y4.62638 Z5.41423

N28490 X-5.20643 Y4.62638 Z5.46289

N28500 X-5.13645 Y4.62638 Z5.52141

N28510 X-5.08763 Y4.62638 Z5.55622

N28520 X-4.98004 Y4.62638 Z5.62911

N28530 X-4.965 Y4.62638 Z5.63961

N28540 X-4.83463 Y4.62638 Z5.71024

N28550 X-4.71914 Y4.62638 Z5.76377

N28560 X-4.70866 Y4.62638 Z5.76798

N28570 X-4.57933 Y4.62638 Z5.8133

N28580 X-4.53665 Y4.62638 Z5.82301

N28590 X-4.46561 Y4.62638 Z5.84391

N28600 X-4.42077 Y4.62638 Z5.85195

N28610 X-4.36986 Y4.62638 Z5.8635

N28620 X-4.30713 Y4.62638 Z5.87347

N28630 X-4.28814 Y4.62638 Z5.87677

N28640 X-4.25235 Y4.62638 Z5.88249

N28650 X-4.21132 Y4.62638 Z5.8869

N28660 X-4.19088 Y4.62638 Z5.8888

N28670 X-4.13635 Y4.62638 Z5.89449

N28680 X-4.07402 Y4.62638 Z5.90118

N28690 X-4.05404 Y4.62638 Z5.90344

N28700 X-3.98974 Y4.62638 Z5.90672

N28710 X-3.93529 Y4.62638 Z5.90952

N28720 X-3.89908 Y4.62638 Z5.90912

N28730 X-3.82654 Y4.62638 Z5.9096

N28740 X-3.78457 Y4.62638 Z5.90723

N28750 X-3.71016 Y4.62638 Z5.9037

N28760 X-3.65371 Y4.62638 Z5.8979

N28770 X-3.59236 Y4.62638 Z5.89131

N28780 X-3.52779 Y4.62638 Z5.88196

N28790 X-3.46896 Y4.62638 Z5.8731

N28800 X-3.41741 Y4.62638 Z5.86387

N28810 X-3.31294 Y4.62638 Z5.84551

N28820 X-3.29664 Y4.62638 Z5.84229

N28830 X-3.18755 Y4.62638 Z5.82048

N28840 X-3.07598 Y4.62638 Z5.79979

N28850 X-3.03297 Y4.62638 Z5.79064

N28860 X-2.93979 Y4.62638 Z5.77047

N28870 X-2.83383 Y4.62638 Z5.74785

N28880 X-2.79396 Y4.62638 Z5.73959

N28890 X-2.67119 Y4.62638 Z5.71067

N28900 X-2.56478 Y4.62638 Z5.68494

N28910 X-2.51816 Y4.62638 Z5.67284

N28920 X-2.35459 Y4.62638 Z5.63163

N28930 X-2.26606 Y4.62638 Z5.60843

N28940 X-2.18543 Y4.62638 Z5.58739

N28950 X-2.06037 Y4.62638 Z5.5548

N28960 X-2.02954 Y4.62638 Z5.54699

N28970 X-1.91691 Y4.62638 Z5.5167

N28980 X-1.84287 Y4.62638 Z5.50042

N28990 X-1.79325 Y4.62638 Z5.48751

N29000 X-1.74115 Y4.62638 Z5.47709

N29010 X-1.68239 Y4.62638 Z5.46407

N29020 X-1.63172 Y4.62638 Z5.45597

N29030 X-1.58395 Y4.62638 Z5.4465

N29040 X-1.47223 Y4.62638 Z5.42812

N29050 X-1.42422 Y4.62638 Z5.42081

N29060 X-1.35506 Y4.62638 Z5.41668

N29070 X-1.32595 Y4.62638 Z5.41446

N29080 X-1.28703 Y4.62638 Z5.41334

N29090 X-1.25272 Y4.62638 Z5.41114

N29100 X-1.20052 Y4.62638 Z5.40968

N29110 X-1.18175 Y4.62638 Z5.40884

N29120 X-1.10571 Y4.62638 Z5.40826

N29130 X-1.09099 Y4.62638 Z5.40835

N29140 X-1.02613 Y4.62638 Z5.40983

N29150 X-1.00915 Y4.62638 Z5.41046

N29160 X-0.959917 Y4.62638 Z5.41231

N29170 X-0.916177 Y4.62638 Z5.41593

N29180 X-0.892025 Y4.62638 Z5.41748

N29190 X-0.864689 Y4.62638 Z5.42208

N29200 X-0.814 Y4.62638 Z5.42831

N29210 X-0.760074 Y4.62638 Z5.44106

N29220 X-0.746079 Y4.62638 Z5.44408

N29230 X-0.728166 Y4.62638 Z5.44908

N29240 X-0.677809 Y4.62638 Z5.46088

N29250 X-0.622391 Y4.62638 Z5.47742

N29260 X-0.610452 Y4.62638 Z5.48047

N29270 X-0.573026 Y4.62638 Z5.49266

N29280 X-0.54613 Y4.62638 Z5.50102

N29290 X-0.486668 Y4.62638 Z5.52153

N29300 X-0.459125 Y4.62638 Z5.53324

N29310 X-0.432871 Y4.62638 Z5.54291

N29320 X-0.394348 Y4.62638 Z5.56062

N29330 X-0.37645 Y4.62638 Z5.56846

N29340 X-0.357788 Y4.62638 Z5.57862

N29350 X-0.310477 Y4.62638 Z5.6015

N29360 X-0.246348 Y4.62638 Z5.63683

N29370 X-0.232533 Y4.62638 Z5.64441

N29380 X-0.223652 Y4.62638 Z5.64969

N29390 X-0.143924 Y4.62638 Z5.69456

N29400 X-0.107537 Y4.62638 Z5.71657

N29410 X-0.0497227 Y4.62638 Z5.75019

N29420 X0.132484 Y4.62638 Z5.85889

N29430 X0.233512 Y4.62638 Z5.91633

N29440 X0.283287 Y4.62638 Z5.94398

N29450 X0.386082 Y4.62638 Z5.99704

N29460 X0.422051 Y4.62638 Z6.01501

N29470 X0.509602 Y4.62638 Z6.05587

N29480 X0.542979 Y4.62638 Z6.07131

N29490 X0.614755 Y4.62638 Z6.10347

N29500 X0.648132 Y4.62638 Z6.11837

N29510 X0.702816 Y4.62638 Z6.14234

N29520 X0.747067 Y4.62638 Z6.16163

N29530 X0.780023 Y4.62638 Z6.17535

N29540 X0.849073 Y4.62638 Z6.20432

N29550 X0.938197 Y4.62638 Z6.23557

N29560 X1.01601 Y4.62638 Z6.26054

N29570 X1.03306 Y4.62638 Z6.26494

N29580 X1.11919 Y4.62638 Z6.28469

N29590 X1.18566 Y4.62638 Z6.29898

N29600 X1.21737 Y4.62638 Z6.30386

N29610 X1.39346 Y4.62638 Z6.33719

N29620 X1.44034 Y4.62638 Z6.34521

N29630 X1.46523 Y4.62638 Z6.34997

N29640 X1.51658 Y4.62638 Z6.35864

N29650 X1.53004 Y4.62638 Z6.36075

N29660 X1.58414 Y4.62638 Z6.36997

N29670 X1.63396 Y4.62638 Z6.37569

N29680 X1.6768 Y4.62638 Z6.38299

N29690 X1.69992 Y4.62638 Z6.38618

N29700 X1.7723 Y4.62638 Z6.39845

N29710 X1.84349 Y4.62638 Z6.4083

N29720 X1.91829 Y4.62638 Z6.41995

N29730 X1.97527 Y4.62638 Z6.42931

N29740 X2.00949 Y4.62638 Z6.43473

N29750 X2.04518 Y4.62638 Z6.4413

N29760 X2.12215 Y4.62638 Z6.45035

N29770 X2.18779 Y4.62638 Z6.46022

N29780 X2.23399 Y4.62638 Z6.46735

N29790 X2.31332 Y4.62638 Z6.47487

N29800 X2.35923 Y4.62638 Z6.48077

N29810 X2.39425 Y4.62638 Z6.48249

N29820 X2.46363 Y4.62638 Z6.48985

N29830 X2.53753 Y4.62638 Z6.49862

N29840 X2.57029 Y4.62638 Z6.50238

N29850 X2.68645 Y4.62638 Z6.51698

N29860 X2.79963 Y4.62638 Z6.52253

N29870 X2.8787 Y4.62638 Z6.52052

N29880 X2.91467 Y4.62638 Z6.51908

N29890 X2.94359 Y4.62638 Z6.5163

N29900 X3.02715 Y4.62638 Z6.50228

N29910 X3.1042 Y4.62638 Z6.48587

N29920 X3.13922 Y4.62638 Z6.47614

N29930 X3.17622 Y4.62638 Z6.46546

N29940 X3.25228 Y4.62638 Z6.44371

N29950 X3.34588 Y4.62638 Z6.41112

N29960 X3.36149 Y4.62638 Z6.40568

N29970 X3.38346 Y4.62638 Z6.39647

N29980 X3.46733 Y4.62638 Z6.35677

N29990 X3.53885 Y4.62638 Z6.3178

N30000 X3.56802 Y4.62638 Z6.30152

N30010 X3.59418 Y4.62638 Z6.28546

N30020 X3.66049 Y4.62638 Z6.23934

N30030 X3.73728 Y4.62638 Z6.18235

N30040 X3.74809 Y4.62638 Z6.1742

N30050 X3.75673 Y4.62638 Z6.16749

N30060 X3.82864 Y4.62638 Z6.10379

N30070 X3.88548 Y4.62638 Z6.05189

N30080 X3.90908 Y4.62638 Z6.02747

N30090 X3.98335 Y4.62638 Z5.94732

N30100 X3.99114 Y4.62638 Z5.93791

N30110 X4.04563 Y4.62638 Z5.87023

N30120 X4.06799 Y4.62638 Z5.84229

N30130 X4.14674 Y4.62638 Z5.73088

N30140 X4.15834 Y4.62638 Z5.71365

N30150 X4.23304 Y4.62638 Z5.58895

N30160 X4.31151 Y4.62638 Z5.44088

N30170 X4.34038 Y4.62638 Z5.38356

N30180 X4.40195 Y4.62638 Z5.26034

N30190 X4.46056 Y4.62638 Z5.14745

N30200 X4.4722 Y4.62638 Z5.1238

N30210 X4.55437 Y4.62638 Z4.96732

N30220 X4.69644 Y4.54083 Z4.91832

N30230 X4.59949 Y4.54083 Z5.10988

N30240 X4.51037 Y4.54083 Z5.29011

N30250 X4.49276 Y4.54083 Z5.32573

N30260 X4.42907 Y4.54083 Z5.4503

N30270 X4.38453 Y4.54083 Z5.53498

N30280 X4.35471 Y4.54083 Z5.59051

N30290 X4.28936 Y4.54083 Z5.70411

N30300 X4.23622 Y4.54083 Z5.78302

N30310 X4.20774 Y4.54083 Z5.82477

N30320 X4.12581 Y4.54083 Z5.92695

N30330 X4.09867 Y4.54083 Z5.96087

N30340 X4.02635 Y4.54083 Z6.04115

N30350 X4.01623 Y4.54083 Z6.0521

N30360 X3.93908 Y4.54083 Z6.12445

N30370 X3.91238 Y4.54083 Z6.1488

N30380 X3.85708 Y4.54083 Z6.19237

N30390 X3.78278 Y4.54083 Z6.25

N30400 X3.77264 Y4.54083 Z6.25787

N30410 X3.76424 Y4.54083 Z6.26421

N30420 X3.68167 Y4.54083 Z6.31848

N30430 X3.58727 Y4.54083 Z6.37774

N30440 X3.48424 Y4.54083 Z6.42834

N30450 X3.37746 Y4.54083 Z6.47414

N30460 X3.36296 Y4.54083 Z6.47919

N30470 X3.26672 Y4.54083 Z6.50906

N30480 X3.22212 Y4.54083 Z6.52001

N30490 X3.15472 Y4.54083 Z6.53704

N30500 X3.09266 Y4.54083 Z6.54873

N30510 X3.03624 Y4.54083 Z6.56169

N30520 X2.99063 Y4.54083 Z6.56765

N30530 X2.88371 Y4.54083 Z6.57888

N30540 X2.77974 Y4.54083 Z6.57471

N30550 X2.69591 Y4.54083 Z6.56802

N30560 X2.67962 Y4.54083 Z6.56655

N30570 X2.58162 Y4.54083 Z6.55035

N30580 X2.49636 Y4.54083 Z6.53348

N30590 X2.47937 Y4.54083 Z6.53152

N30600 X2.39749 Y4.54083 Z6.518

N30610 X2.37813 Y4.54083 Z6.51638

N30620 X2.32039 Y4.54083 Z6.50929

N30630 X2.27536 Y4.54083 Z6.50186

N30640 X2.15746 Y4.54083 Z6.48335

N30650 X2.12792 Y4.54083 Z6.47727

N30660 X2.05286 Y4.54083 Z6.46402

N30670 X1.99939 Y4.54083 Z6.45417

N30680 X1.97385 Y4.54083 Z6.45013

N30690 X1.89417 Y4.54083 Z6.43299

N30700 X1.82231 Y4.54083 Z6.41962

N30710 X1.79894 Y4.54083 Z6.4149

N30720 X1.78768 Y4.54083 Z6.41334

N30730 X1.69352 Y4.54083 Z6.39709

N30740 X1.68357 Y4.54083 Z6.39572

N30750 X1.61196 Y4.54083 Z6.38205

N30760 X1.54388 Y4.54083 Z6.37225

N30770 X1.47194 Y4.54083 Z6.35853

N30780 X1.41551 Y4.54083 Z6.34792

N30790 X1.36664 Y4.54083 Z6.33757

N30800 X1.31252 Y4.54083 Z6.32532

N30810 X1.26916 Y4.54083 Z6.31747

N30820 X1.18287 Y4.54083 Z6.2978

N30830 X1.12996 Y4.54083 Z6.28727

N30840 X1.06431 Y4.54083 Z6.27059

N30850 X1.02967 Y4.54083 Z6.26108

N30860 X0.976317 Y4.54083 Z6.24554

N30870 X0.935149 Y4.54083 Z6.23245

N30880 X0.891042 Y4.54083 Z6.21752

N30890 X0.845304 Y4.54083 Z6.20029

N30900 X0.817591 Y4.54083 Z6.18951

N30910 X0.757422 Y4.54083 Z6.16531

N30920 X0.701863 Y4.54083 Z6.1419

N30930 X0.664744 Y4.54083 Z6.12612

N30940 X0.591096 Y4.54083 Z6.09362

N30950 X0.566572 Y4.54083 Z6.08243

N30960 X0.475271 Y4.54083 Z6.04034

N30970 X0.45514 Y4.54083 Z6.03046

N30980 X0.350027 Y4.54083 Z5.9778

N30990 X0.32686 Y4.54083 Z5.96535

N31000 X0.215426 Y4.54083 Z5.90343

N31010 X0.192112 Y4.54083 Z5.88973

N31020 X0.0642042 Y4.54083 Z5.81288

N31030 X0.0530122 Y4.54083 Z5.80579

N31040 X-0.0582412 Y4.54083 Z5.73717

N31050 X-0.111356 Y4.54083 Z5.70545

N31060 X-0.145448 Y4.54083 Z5.68437

N31070 X-0.219378 Y4.54083 Z5.64084

N31080 X-0.238848 Y4.54083 Z5.6296

N31090 X-0.311709 Y4.54083 Z5.58651

N31100 X-0.374373 Y4.54083 Z5.55163

N31110 X-0.413322 Y4.54083 Z5.53229

N31120 X-0.460445 Y4.54083 Z5.50795

N31130 X-0.497483 Y4.54083 Z5.49336

N31140 X-0.511948 Y4.54083 Z5.48691

N31150 X-0.576433 Y4.54083 Z5.46356

N31160 X-0.634837 Y4.54083 Z5.44325

N31170 X-0.648904 Y4.54083 Z5.43924

N31180 X-0.700295 Y4.54083 Z5.42262

N31190 X-0.76535 Y4.54083 Z5.40534

N31200 X-0.832814 Y4.54083 Z5.38941

N31210 X-0.845773 Y4.54083 Z5.38691

N31220 X-0.880657 Y4.54083 Z5.38189

N31230 X-0.914595 Y4.54083 Z5.37617

N31240 X-0.932046 Y4.54083 Z5.37506

N31250 X-0.966367 Y4.54083 Z5.37161

N31260 X-0.997422 Y4.54083 Z5.37003

N31270 X-1.01283 Y4.54083 Z5.36915

N31280 X-1.06185 Y4.54083 Z5.36764

N31290 X-1.08263 Y4.54083 Z5.36716

N31300 X-1.14852 Y4.54083 Z5.36739

N31310 X-1.16788 Y4.54083 Z5.36747

N31320 X-1.23089 Y4.54083 Z5.37004

N31330 X-1.25682 Y4.54083 Z5.37119

N31340 X-1.32929 Y4.54083 Z5.3751

N31350 X-1.41268 Y4.54083 Z5.38349

N31360 X-1.43072 Y4.54083 Z5.38624

N31370 X-1.49615 Y4.54083 Z5.39518

N31380 X-1.50858 Y4.54083 Z5.39725

N31390 X-1.59276 Y4.54083 Z5.41223

N31400 X-1.66598 Y4.54083 Z5.42553

N31410 X-1.73586 Y4.54083 Z5.44183

N31420 X-1.78434 Y4.54083 Z5.45153

N31430 X-1.84455 Y4.54083 Z5.46719

N31440 X-1.91258 Y4.54083 Z5.48463

N31450 X-1.97346 Y4.54083 Z5.50178

N31460 X-2.02117 Y4.54083 Z5.51423

N31470 X-2.10755 Y4.54083 Z5.5378

N31480 X-2.19262 Y4.54083 Z5.56069

N31490 X-2.2644 Y4.54083 Z5.58054

N31500 X-2.44516 Y4.54083 Z5.62938

N31510 X-2.45736 Y4.54083 Z5.63254

N31520 X-2.66003 Y4.54083 Z5.68514

N31530 X-2.77328 Y4.54083 Z5.71253

N31540 X-2.8767 Y4.54083 Z5.73869

N31550 X-2.98009 Y4.54083 Z5.76324

N31560 X-3.06956 Y4.54083 Z5.78259

N31570 X-3.15652 Y4.54083 Z5.80109

N31580 X-3.21181 Y4.54083 Z5.81316

N31590 X-3.25068 Y4.54083 Z5.82164

N31600 X-3.32408 Y4.54083 Z5.83601

N31610 X-3.40772 Y4.54083 Z5.8528

N31620 X-3.42672 Y4.54083 Z5.85659

N31630 X-3.51637 Y4.54083 Z5.87241

N31640 X-3.55021 Y4.54083 Z5.878

N31650 X-3.62259 Y4.54083 Z5.8885

N31660 X-3.65825 Y4.54083 Z5.89341

N31670 X-3.74168 Y4.54083 Z5.90213

N31680 X-3.76719 Y4.54083 Z5.90477

N31690 X-3.85596 Y4.54083 Z5.90983

N31700 X-3.87913 Y4.54083 Z5.91124

N31710 X-3.95223 Y4.54083 Z5.91183

N31720 X-4.00227 Y4.54083 Z5.91225

N31730 X-4.03351 Y4.54083 Z5.91075

N31740 X-4.06113 Y4.54083 Z5.90939

N31750 X-4.10639 Y4.54083 Z5.90785

N31760 X-4.18508 Y4.54083 Z5.90404

N31770 X-4.26253 Y4.54083 Z5.89737

N31780 X-4.30026 Y4.54083 Z5.89248

N31790 X-4.35222 Y4.54083 Z5.88786

N31800 X-4.41252 Y4.54083 Z5.87769

N31810 X-4.45887 Y4.54083 Z5.87261

N31820 X-4.55283 Y4.54083 Z5.85248

N31830 X-4.58225 Y4.54083 Z5.84753

N31840 X-4.62932 Y4.54083 Z5.83495

N31850 X-4.71292 Y4.54083 Z5.80963

N31860 X-4.77578 Y4.54083 Z5.7844

N31870 X-4.84155 Y4.54083 Z5.75972

N31880 X-4.89095 Y4.54083 Z5.73802

N31890 X-4.96543 Y4.54083 Z5.69925

N31900 X-5.08752 Y4.54083 Z5.62912

N31910 X-5.1937 Y4.54083 Z5.5543

N31920 X-5.24127 Y4.54083 Z5.51896

N31930 X-5.29679 Y4.54083 Z5.47096

N31940 X-5.3632 Y4.54083 Z5.4104

N31950 X-5.39184 Y4.54083 Z5.38019

N31960 X-5.45936 Y4.54083 Z5.30583

N31970 X-5.48167 Y4.54083 Z5.27642

N31980 X-5.53561 Y4.54083 Z5.20362

N31990 X-5.5697 Y4.54083 Z5.14591

N32000 X-5.64996 Y4.54083 Z4.98794

N32010 X-5.6594 Y4.54083 Z4.96497

N32020 X-5.69939 Y4.54083 Z4.86672

N32030 X-5.73295 Y4.54083 Z4.77945

N32040 X-5.74558 Y4.54083 Z4.74909

N32050 X-5.76304 Y4.54083 Z4.70479

N32060 X-5.7831 Y4.54083 Z4.65203

N32070 X-5.78985 Y4.54083 Z4.63351

N32080 X-5.87376 Y4.45318 Z4.6106

N32090 X-5.83601 Y4.45318 Z4.7141

N32100 X-5.82643 Y4.45318 Z4.7393

N32110 X-5.80075 Y4.45318 Z4.81884

N32120 X-5.7768 Y4.45318 Z4.88297

N32130 X-5.76264 Y4.45318 Z4.91912

N32140 X-5.75106 Y4.45318 Z4.94683

N32150 X-5.71508 Y4.45318 Z5.0301

N32160 X-5.66742 Y4.45318 Z5.13085

N32170 X-5.60767 Y4.45318 Z5.23101

N32180 X-5.56448 Y4.45318 Z5.28989

N32190 X-5.54041 Y4.45318 Z5.32245

N32200 X-5.47092 Y4.45318 Z5.40057

N32210 X-5.45767 Y4.45318 Z5.41515

N32220 X-5.37742 Y4.45318 Z5.48996

N32230 X-5.34899 Y4.45318 Z5.51566

N32240 X-5.20612 Y4.45318 Z5.62249

N32250 X-5.18054 Y4.45318 Z5.63818

N32260 X-5.14199 Y4.45318 Z5.66067

N32270 X-5.07423 Y4.45318 Z5.7009

N32280 X-5.02744 Y4.45318 Z5.72706

N32290 X-4.96139 Y4.45318 Z5.75795

N32300 X-4.86528 Y4.45318 Z5.79867

N32310 X-4.84138 Y4.45318 Z5.80917

N32320 X-4.82309 Y4.45318 Z5.81603

N32330 X-4.71318 Y4.45318 Z5.85073

N32340 X-4.59128 Y4.45318 Z5.8802

N32350 X-4.57476 Y4.45318 Z5.88287

N32360 X-4.45672 Y4.45318 Z5.90059

N32370 X-4.39895 Y4.45318 Z5.90413

N32380 X-4.33904 Y4.45318 Z5.91048

N32390 X-4.27898 Y4.45318 Z5.91146

N32400 X-4.22983 Y4.45318 Z5.91516

N32410 X-4.15087 Y4.45318 Z5.91671

N32420 X-4.14083 Y4.45318 Z5.91723

N32430 X-4.12497 Y4.45318 Z5.91757

N32440 X-4.06035 Y4.45318 Z5.91645

N32450 X-4.01622 Y4.45318 Z5.9149

N32460 X-3.98702 Y4.45318 Z5.9145

N32470 X-3.95869 Y4.45318 Z5.91399

N32480 X-3.90698 Y4.45318 Z5.91063

N32490 X-3.83643 Y4.45318 Z5.90617

N32500 X-3.81295 Y4.45318 Z5.90371

N32510 X-3.72916 Y4.45318 Z5.89511

N32520 X-3.70493 Y4.45318 Z5.8918

N32530 X-3.62985 Y4.45318 Z5.88154

N32540 X-3.60306 Y4.45318 Z5.87722

N32550 X-3.53144 Y4.45318 Z5.86526

N32560 X-3.51603 Y4.45318 Z5.86255

N32570 X-3.48877 Y4.45318 Z5.85702

N32580 X-3.42838 Y4.45318 Z5.84538

N32590 X-3.39165 Y4.45318 Z5.8379

N32600 X-3.33891 Y4.45318 Z5.82614

N32610 X-3.2777 Y4.45318 Z5.81215

N32620 X-3.23924 Y4.45318 Z5.80375

N32630 X-3.19487 Y4.45318 Z5.79407

N32640 X-3.1052 Y4.45318 Z5.7727

N32650 X-2.99903 Y4.45318 Z5.74649

N32660 X-2.94318 Y4.45318 Z5.73323

N32670 X-2.87071 Y4.45318 Z5.7149

N32680 X-2.74913 Y4.45318 Z5.68307

N32690 X-2.66574 Y4.45318 Z5.65969

N32700 X-2.52894 Y4.45318 Z5.62319

N32710 X-2.34406 Y4.45318 Z5.57151

N32720 X-2.17557 Y4.45318 Z5.5236

N32730 X-2.09828 Y4.45318 Z5.50129

N32740 X-2.04234 Y4.45318 Z5.48568

N32750 X-1.93941 Y4.45318 Z5.45691

N32760 X-1.92176 Y4.45318 Z5.45185

N32770 X-1.79501 Y4.45318 Z5.41759

N32780 X-1.75335 Y4.45318 Z5.40672

N32790 X-1.6452 Y4.45318 Z5.38242

N32800 X-1.59032 Y4.45318 Z5.37142

N32810 X-1.54025 Y4.45318 Z5.36144

N32820 X-1.50285 Y4.45318 Z5.35517

N32830 X-1.46163 Y4.45318 Z5.34818

N32840 X-1.39967 Y4.45318 Z5.33924

N32850 X-1.35048 Y4.45318 Z5.33462

N32860 X-1.32461 Y4.45318 Z5.33187

N32870 X-1.28683 Y4.45318 Z5.3294

N32880 X-1.2452 Y4.45318 Z5.32647

N32890 X-1.22065 Y4.45318 Z5.32564

N32900 X-1.1577 Y4.45318 Z5.32353

N32910 X-1.11115 Y4.45318 Z5.3234

N32920 X-1.0587 Y4.45318 Z5.32366

N32930 X-1.03795 Y4.45318 Z5.32423

N32940 X-1.00425 Y4.45318 Z5.32598

N32950 X-0.986342 Y4.45318 Z5.32685

N32960 X-0.976029 Y4.45318 Z5.32766

N32970 X-0.943438 Y4.45318 Z5.3306

N32980 X-0.905967 Y4.45318 Z5.33574

N32990 X-0.889431 Y4.45318 Z5.33812

N33000 X-0.836448 Y4.45318 Z5.34832

N33010 X-0.771775 Y4.45318 Z5.36368

N33020 X-0.761263 Y4.45318 Z5.36672

N33030 X-0.71808 Y4.45318 Z5.37922

N33040 X-0.663097 Y4.45318 Z5.39778

N33050 X-0.602064 Y4.45318 Z5.41978

N33060 X-0.54491 Y4.45318 Z5.44214

N33070 X-0.491822 Y4.45318 Z5.46512

N33080 X-0.468517 Y4.45318 Z5.47759

N33090 X-0.425655 Y4.45318 Z5.49879

N33100 X-0.381453 Y4.45318 Z5.52531

N33110 X-0.319995 Y4.45318 Z5.56187

N33120 X-0.292677 Y4.45318 Z5.579

N33130 X-0.252302 Y4.45318 Z5.60448

N33140 X-0.198736 Y4.45318 Z5.63639

N33150 X-0.162286 Y4.45318 Z5.65904

N33160 X-0.110884 Y4.45318 Z5.69097

N33170 X0.0199197 Y4.45318 Z5.77338

N33180 X0.0604508 Y4.45318 Z5.79815

N33190 X0.170156 Y4.45318 Z5.86755

N33200 X0.290722 Y4.45318 Z5.93844

N33210 X0.410759 Y4.45318 Z6.00293

N33220 X0.522393 Y4.45318 Z6.05734

N33230 X0.536597 Y4.45318 Z6.06428

N33240 X0.624733 Y4.45318 Z6.10402

N33250 X0.656383 Y4.45318 Z6.11767

N33260 X0.730703 Y4.45318 Z6.1489

N33270 X0.780894 Y4.45318 Z6.16745

N33280 X0.843907 Y4.45318 Z6.19133

N33290 X0.946538 Y4.45318 Z6.22751

N33300 X1.06614 Y4.45318 Z6.26238

N33310 X1.12096 Y4.45318 Z6.27673

N33320 X1.19914 Y4.45318 Z6.29692

N33330 X1.28539 Y4.45318 Z6.31643

N33340 X1.36048 Y4.45318 Z6.33431

N33350 X1.4426 Y4.45318 Z6.35226

N33360 X1.55778 Y4.45318 Z6.37555

N33370 X1.56902 Y4.45318 Z6.37791

N33380 X1.58643 Y4.45318 Z6.38156

N33390 X1.67696 Y4.45318 Z6.39813

N33400 X1.70296 Y4.45318 Z6.40291

N33410 X1.77172 Y4.45318 Z6.4169

N33420 X1.83748 Y4.45318 Z6.43225

N33430 X1.86708 Y4.45318 Z6.43912

N33440 X1.906 Y4.45318 Z6.44916

N33450 X1.97508 Y4.45318 Z6.4637

N33460 X2.08155 Y4.45318 Z6.48856

N33470 X2.10225 Y4.45318 Z6.49222

N33480 X2.24385 Y4.45318 Z6.52136

N33490 X2.25462 Y4.45318 Z6.52305

N33500 X2.26745 Y4.45318 Z6.52517

N33510 X2.38657 Y4.45318 Z6.5475

N33520 X2.42043 Y4.45318 Z6.55282

N33530 X2.49272 Y4.45318 Z6.56893

N33540 X2.51281 Y4.45318 Z6.57246

N33550 X2.55763 Y4.45318 Z6.58421

N33560 X2.57777 Y4.45318 Z6.58872

N33570 X2.59552 Y4.45318 Z6.59285

N33580 X2.65393 Y4.45318 Z6.60288

N33590 X2.70282 Y4.45318 Z6.61022

N33600 X2.75379 Y4.45318 Z6.61702

N33610 X2.80472 Y4.45318 Z6.6217

N33620 X2.85775 Y4.45318 Z6.62256

N33630 X2.90051 Y4.45318 Z6.62223

N33640 X2.95729 Y4.45318 Z6.62089

N33650 X3.01635 Y4.45318 Z6.61228

N33660 X3.0673 Y4.45318 Z6.60835

N33670 X3.12818 Y4.45318 Z6.59713

N33680 X3.17589 Y4.45318 Z6.59052

N33690 X3.25893 Y4.45318 Z6.57215

N33700 X3.28435 Y4.45318 Z6.56659

N33710 X3.33085 Y4.45318 Z6.55345

N33720 X3.39115 Y4.45318 Z6.53421

N33730 X3.43372 Y4.45318 Z6.5181

N33740 X3.49547 Y4.45318 Z6.4936

N33750 X3.55871 Y4.45318 Z6.46599

N33760 X3.59734 Y4.45318 Z6.44639

N33770 X3.63759 Y4.45318 Z6.42451

N33780 X3.69358 Y4.45318 Z6.39331

N33790 X3.74726 Y4.45318 Z6.36327

N33800 X3.78786 Y4.45318 Z6.33636

N33810 X3.8301 Y4.45318 Z6.30817

N33820 X3.87946 Y4.45318 Z6.27614

N33830 X3.92753 Y4.45318 Z6.24378

N33840 X3.9654 Y4.45318 Z6.21435

N33850 X4.01373 Y4.45318 Z6.17595

N33860 X4.03591 Y4.45318 Z6.15847

N33870 X4.05991 Y4.45318 Z6.13683

N33880 X4.10091 Y4.45318 Z6.09792

N33890 X4.13035 Y4.45318 Z6.06589

N33900 X4.17778 Y4.45318 Z6.01585

N33910 X4.20842 Y4.45318 Z5.98388

N33920 X4.26745 Y4.45318 Z5.91256

N33930 X4.29351 Y4.45318 Z5.88031

N33940 X4.32899 Y4.45318 Z5.8316

N33950 X4.35633 Y4.45318 Z5.79348

N33960 X4.37275 Y4.45318 Z5.76771

N33970 X4.40943 Y4.45318 Z5.70864

N33980 X4.43466 Y4.45318 Z5.66549

N33990 X4.47371 Y4.45318 Z5.59674

N34000 X4.53557 Y4.45318 Z5.48177

N34010 X4.5462 Y4.45318 Z5.4618

N34020 X4.57361 Y4.45318 Z5.40707

N34030 X4.63049 Y4.45318 Z5.29204

N34040 X4.7407 Y4.45318 Z5.06351

N34050 X4.7521 Y4.45318 Z5.04109

N34060 X4.84068 Y4.45318 Z4.85883

N34070 X4.98459 Y4.36455 Z4.79311

N34080 X4.97761 Y4.36455 Z4.80831

N34090 X4.90575 Y4.36455 Z4.96174

N34100 X4.81976 Y4.36455 Z5.146

N34110 X4.7798 Y4.36455 Z5.22972

N34120 X4.72637 Y4.36455 Z5.33787

N34130 X4.67171 Y4.36455 Z5.45143

N34140 X4.65909 Y4.36455 Z5.47662

N34150 X4.62712 Y4.36455 Z5.5367

N34160 X4.5911 Y4.36455 Z5.60305

N34170 X4.5809 Y4.36455 Z5.62101

N34180 X4.52032 Y4.36455 Z5.72391

N34190 X4.46059 Y4.36455 Z5.82046

N34200 X4.45377 Y4.36455 Z5.83054

N34210 X4.3883 Y4.36455 Z5.92214

N34220 X4.33017 Y4.36455 Z5.99334

N34230 X4.31296 Y4.36455 Z6.01424

N34240 X4.30444 Y4.36455 Z6.0231

N34250 X4.21893 Y4.36455 Z6.11158

N34260 X4.15235 Y4.36455 Z6.17237

N34270 X4.09799 Y4.36455 Z6.21916

N34280 X4.08033 Y4.36455 Z6.23268

N34290 X4.05706 Y4.36455 Z6.24886

N34300 X3.99677 Y4.36455 Z6.29219

N34310 X3.92763 Y4.36455 Z6.3368

N34320 X3.89964 Y4.36455 Z6.35304

N34330 X3.87924 Y4.36455 Z6.36309

N34340 X3.79408 Y4.36455 Z6.40989

N34350 X3.70301 Y4.36455 Z6.46089

N34360 X3.68894 Y4.36455 Z6.46735

N34370 X3.59679 Y4.36455 Z6.5099

N34380 X3.51597 Y4.36455 Z6.54683

N34390 X3.50011 Y4.36455 Z6.55278

N34400 X3.48333 Y4.36455 Z6.55889

N34410 X3.39834 Y4.36455 Z6.58775

N34420 X3.30789 Y4.36455 Z6.61519

N34430 X3.29457 Y4.36455 Z6.61836

N34440 X3.19639 Y4.36455 Z6.63773

N34450 X3.16364 Y4.36455 Z6.64086

N34460 X3.08401 Y4.36455 Z6.65176

N34470 X3.04862 Y4.36455 Z6.65298

N34480 X2.96345 Y4.36455 Z6.65988

N34490 X2.92088 Y4.36455 Z6.65878

N34500 X2.84453 Y4.36455 Z6.65825

N34510 X2.74132 Y4.36455 Z6.65067

N34520 X2.7287 Y4.36455 Z6.64923

N34530 X2.71351 Y4.36455 Z6.64646

N34540 X2.62302 Y4.36455 Z6.62957

N34550 X2.53137 Y4.36455 Z6.60869

N34560 X2.4562 Y4.36455 Z6.58751

N34570 X2.40882 Y4.36455 Z6.57756

N34580 X2.3401 Y4.36455 Z6.56121

N34590 X2.28913 Y4.36455 Z6.55085

N34600 X2.20245 Y4.36455 Z6.53015

N34610 X2.16055 Y4.36455 Z6.52159

N34620 X2.07155 Y4.36455 Z6.49874

N34630 X2.01842 Y4.36455 Z6.48651

N34640 X1.94913 Y4.36455 Z6.46707

N34650 X1.84968 Y4.36455 Z6.44147

N34660 X1.83455 Y4.36455 Z6.43712

N34670 X1.75011 Y4.36455 Z6.41473

N34680 X1.70357 Y4.36455 Z6.40353

N34690 X1.6589 Y4.36455 Z6.39341

N34700 X1.56199 Y4.36455 Z6.37207

N34710 X1.54549 Y4.36455 Z6.36861

N34720 X1.51332 Y4.36455 Z6.36187

N34730 X1.40652 Y4.36455 Z6.33644

N34740 X1.29698 Y4.36455 Z6.3104

N34750 X1.21966 Y4.36455 Z6.29008

N34760 X1.13953 Y4.36455 Z6.26905

N34770 X1.01782 Y4.36455 Z6.23437

N34780 X0.999806 Y4.36455 Z6.229

N34790 X0.967475 Y4.36455 Z6.21857

N34800 X0.870059 Y4.36455 Z6.18687

N34810 X0.825662 Y4.36455 Z6.17214

N34820 X0.733943 Y4.36455 Z6.13824

N34830 X0.694686 Y4.36455 Z6.12338

N34840 X0.612419 Y4.36455 Z6.0884

N34850 X0.593239 Y4.36455 Z6.07936

N34860 X0.524657 Y4.36455 Z6.04713

N34870 X0.493377 Y4.36455 Z6.03183

N34880 X0.484067 Y4.36455 Z6.02731

N34890 X0.387248 Y4.36455 Z5.97574

N34900 X0.361614 Y4.36455 Z5.96198

N34910 X0.271699 Y4.36455 Z5.9112

N34920 X0.251173 Y4.36455 Z5.89914

N34930 X0.157966 Y4.36455 Z5.84359

N34940 X-0.0320891 Y4.36455 Z5.72104

N34950 X-0.145023 Y4.36455 Z5.64864

N34960 X-0.225545 Y4.36455 Z5.59741

N34970 X-0.24818 Y4.36455 Z5.58313

N34980 X-0.296583 Y4.36455 Z5.55278

N34990 X-0.348233 Y4.36455 Z5.51939

N35000 X-0.365719 Y4.36455 Z5.50799

N35010 X-0.437797 Y4.36455 Z5.4645

N35020 X-0.454994 Y4.36455 Z5.45398

N35030 X-0.528821 Y4.36455 Z5.41497

N35040 X-0.585469 Y4.36455 Z5.38883

N35050 X-0.600314 Y4.36455 Z5.38239

N35060 X-0.637075 Y4.36455 Z5.36758

N35070 X-0.663148 Y4.36455 Z5.35758

N35080 X-0.684961 Y4.36455 Z5.34955

N35090 X-0.722466 Y4.36455 Z5.33678

N35100 X-0.73829 Y4.36455 Z5.33166

N35110 X-0.775593 Y4.36455 Z5.32049

N35120 X-0.804177 Y4.36455 Z5.31312

N35130 X-0.817659 Y4.36455 Z5.30984

N35140 X-0.83852 Y4.36455 Z5.30556

N35150 X-0.868251 Y4.36455 Z5.29941

N35160 X-0.884592 Y4.36455 Z5.29642

N35170 X-0.929789 Y4.36455 Z5.28971

N35180 X-0.959566 Y4.36455 Z5.28561

N35190 X-0.997601 Y4.36455 Z5.28204

N35200 X-1.01009 Y4.36455 Z5.28105

N35210 X-1.02481 Y4.36455 Z5.28034

N35220 X-1.06725 Y4.36455 Z5.27899

N35230 X-1.14069 Y4.36455 Z5.27875

N35240 X-1.15445 Y4.36455 Z5.27896

N35250 X-1.17164 Y4.36455 Z5.27939

N35260 X-1.24858 Y4.36455 Z5.28184

N35270 X-1.30844 Y4.36455 Z5.28569

N35280 X-1.31961 Y4.36455 Z5.28642

N35290 X-1.33248 Y4.36455 Z5.28779

N35300 X-1.38701 Y4.36455 Z5.29372

N35310 X-1.45165 Y4.36455 Z5.3032

N35320 X-1.46608 Y4.36455 Z5.3054

N35330 X-1.55584 Y4.36455 Z5.32203

N35340 X-1.58866 Y4.36455 Z5.3285

N35350 X-1.70136 Y4.36455 Z5.35391

N35360 X-1.73285 Y4.36455 Z5.36212

N35370 X-1.82348 Y4.36455 Z5.38577

N35380 X-1.86583 Y4.36455 Z5.39787

N35390 X-1.9478 Y4.36455 Z5.42106

N35400 X-1.97407 Y4.36455 Z5.42859

N35410 X-2.07912 Y4.36455 Z5.45982

N35420 X-2.18157 Y4.36455 Z5.48987

N35430 X-2.22495 Y4.36455 Z5.5031

N35440 X-2.29729 Y4.36455 Z5.52467

N35450 X-2.38089 Y4.36455 Z5.54925

N35460 X-2.48097 Y4.36455 Z5.57814

N35470 X-2.56582 Y4.36455 Z5.6034

N35480 X-2.61685 Y4.36455 Z5.61869

N35490 X-2.80332 Y4.36455 Z5.67104

N35500 X-2.83651 Y4.36455 Z5.68019

N35510 X-2.98621 Y4.36455 Z5.72116

N35520 X-3.11909 Y4.36455 Z5.75572

N35530 X-3.13571 Y4.36455 Z5.76027

N35540 X-3.15453 Y4.36455 Z5.76525

N35550 X-3.25097 Y4.36455 Z5.78959

N35560 X-3.32709 Y4.36455 Z5.80839

N35570 X-3.34578 Y4.36455 Z5.81333

N35580 X-3.36459 Y4.36455 Z5.81823

N35590 X-3.42245 Y4.36455 Z5.8321

N35600 X-3.51451 Y4.36455 Z5.85232

N35610 X-3.52635 Y4.36455 Z5.85482

N35620 X-3.608 Y4.36455 Z5.87021

N35630 X-3.64798 Y4.36455 Z5.87653

N35640 X-3.70265 Y4.36455 Z5.88568

N35650 X-3.75364 Y4.36455 Z5.89259

N35660 X-3.80596 Y4.36455 Z5.89995

N35670 X-3.84199 Y4.36455 Z5.90393

N35680 X-3.90973 Y4.36455 Z5.91167

N35690 X-3.92401 Y4.36455 Z5.91349

N35700 X-3.99197 Y4.36455 Z5.91989

N35710 X-4.08299 Y4.36455 Z5.92656

N35720 X-4.16791 Y4.36455 Z5.92975

N35730 X-4.22381 Y4.36455 Z5.93301

N35740 X-4.27561 Y4.36455 Z5.93209

N35750 X-4.34341 Y4.36455 Z5.93352

N35760 X-4.40718 Y4.36455 Z5.92997

N35770 X-4.46023 Y4.36455 Z5.92841

N35780 X-4.53297 Y4.36455 Z5.92111

N35790 X-4.58327 Y4.36455 Z5.91481

N35800 X-4.64146 Y4.36455 Z5.90317

N35810 X-4.71061 Y4.36455 Z5.89019

N35820 X-4.75494 Y4.36455 Z5.87954

N35830 X-4.83466 Y4.36455 Z5.85562

N35840 X-4.93877 Y4.36455 Z5.81897

N35850 X-4.95268 Y4.36455 Z5.81429

N35860 X-4.9636 Y4.36455 Z5.8101

N35870 X-5.06134 Y4.36455 Z5.76561

N35880 X-5.15515 Y4.36455 Z5.71984

N35890 X-5.16439 Y4.36455 Z5.71445

N35900 X-5.17724 Y4.36455 Z5.70657

N35910 X-5.26358 Y4.36455 Z5.65413

N35920 X-5.31789 Y4.36455 Z5.62007

N35930 X-5.35811 Y4.36455 Z5.58952

N35940 X-5.44804 Y4.36455 Z5.51944

N35950 X-5.53935 Y4.36455 Z5.433

N35960 X-5.60685 Y4.36455 Z5.35602

N35970 X-5.63541 Y4.36455 Z5.31777

N35980 X-5.66705 Y4.36455 Z5.27516

N35990 X-5.70644 Y4.36455 Z5.2136

N36000 X-5.7227 Y4.36455 Z5.18647

N36010 X-5.74349 Y4.36455 Z5.14488

N36020 X-5.77193 Y4.36455 Z5.08678

N36030 X-5.82911 Y4.36455 Z4.95479

N36040 X-5.8698 Y4.36455 Z4.84012

N36050 X-5.88207 Y4.36455 Z4.80168

N36060 X-5.90608 Y4.36455 Z4.71934

N36070 X-5.9274 Y4.36455 Z4.66102

N36080 X-5.95201 Y4.36455 Z4.59052

N36090 X-6.02794 Y4.27488 Z4.57167

N36100 X-5.97913 Y4.27488 Z4.70631

N36110 X-5.96532 Y4.27488 Z4.75497

N36120 X-5.93994 Y4.27488 Z4.83905

N36130 X-5.89663 Y4.27488 Z4.9674

N36140 X-5.85325 Y4.27488 Z5.07991

N36150 X-5.80959 Y4.27488 Z5.17513

N36160 X-5.76279 Y4.27488 Z5.25904

N36170 X-5.74514 Y4.27488 Z5.28655

N36180 X-5.71491 Y4.27488 Z5.32777

N36190 X-5.69354 Y4.27488 Z5.3559

N36200 X-5.65987 Y4.27488 Z5.39803

N36210 X-5.60109 Y4.27488 Z5.46687

N36220 X-5.52207 Y4.27488 Z5.54199

N36230 X-5.51232 Y4.27488 Z5.54978

N36240 X-5.46994 Y4.27488 Z5.58332

N36250 X-5.42985 Y4.27488 Z5.61434

N36260 X-5.4164 Y4.27488 Z5.62478

N36270 X-5.34209 Y4.27488 Z5.67251

N36280 X-5.274 Y4.27488 Z5.71545

N36290 X-5.24665 Y4.27488 Z5.72992

N36300 X-5.21015 Y4.27488 Z5.74838

N36310 X-5.1481 Y4.27488 Z5.77978

N36320 X-5.09512 Y4.27488 Z5.8053

N36330 X-5.04705 Y4.27488 Z5.82488

N36340 X-4.9872 Y4.27488 Z5.84736

N36350 X-4.94078 Y4.27488 Z5.86514

N36360 X-4.89836 Y4.27488 Z5.87943

N36370 X-4.82794 Y4.27488 Z5.89904

N36380 X-4.7499 Y4.27488 Z5.91669

N36390 X-4.70619 Y4.27488 Z5.92719

N36400 X-4.6742 Y4.27488 Z5.93319

N36410 X-4.58323 Y4.27488 Z5.94609

N36420 X-4.47199 Y4.27488 Z5.95594

N36430 X-4.45788 Y4.27488 Z5.95641

N36440 X-4.35318 Y4.27488 Z5.95839

N36450 X-4.29373 Y4.27488 Z5.9555

N36460 X-4.24359 Y4.27488 Z5.95442

N36470 X-4.17631 Y4.27488 Z5.9484

N36480 X-4.12766 Y4.27488 Z5.94549

N36490 X-4.07652 Y4.27488 Z5.93949

N36500 X-4.00636 Y4.27488 Z5.93258

N36510 X-3.98626 Y4.27488 Z5.92971

N36520 X-3.92078 Y4.27488 Z5.92077

N36530 X-3.90212 Y4.27488 Z5.91831

N36540 X-3.82589 Y4.27488 Z5.90623

N36550 X-3.78682 Y4.27488 Z5.89989

N36560 X-3.74829 Y4.27488 Z5.893

N36570 X-3.66596 Y4.27488 Z5.87809

N36580 X-3.65089 Y4.27488 Z5.87497

N36590 X-3.53181 Y4.27488 Z5.84865

N36600 X-3.4996 Y4.27488 Z5.84169

N36610 X-3.34448 Y4.27488 Z5.79975

N36620 X-3.33071 Y4.27488 Z5.79601

N36630 X-3.23828 Y4.27488 Z5.76946

N36640 X-3.22133 Y4.27488 Z5.76488

N36650 X-3.18314 Y4.27488 Z5.75413

N36660 X-3.09795 Y4.27488 Z5.72982

N36670 X-3.03778 Y4.27488 Z5.71227

N36680 X-2.96218 Y4.27488 Z5.69103

N36690 X-2.91262 Y4.27488 Z5.67676

N36700 X-2.83658 Y4.27488 Z5.65453

N36710 X-2.73568 Y4.27488 Z5.62538

N36720 X-2.66567 Y4.27488 Z5.60418

N36730 X-2.54067 Y4.27488 Z5.56655

N36740 X-2.49524 Y4.27488 Z5.55225

N36750 X-2.40589 Y4.27488 Z5.52424

N36760 X-2.3736 Y4.27488 Z5.51433

N36770 X-2.3457 Y4.27488 Z5.5057

N36780 X-2.25049 Y4.27488 Z5.47638

N36790 X-2.20715 Y4.27488 Z5.46247

N36800 X-2.10395 Y4.27488 Z5.43093

N36810 X-2.00251 Y4.27488 Z5.39921

N36820 X-1.96514 Y4.27488 Z5.38803

N36830 X-1.86983 Y4.27488 Z5.35949

N36840 X-1.84037 Y4.27488 Z5.35069

N36850 X-1.74108 Y4.27488 Z5.32281

N36860 X-1.70065 Y4.27488 Z5.31243

N36870 X-1.65754 Y4.27488 Z5.30168

N36880 X-1.62194 Y4.27488 Z5.29375

N36890 X-1.55554 Y4.27488 Z5.27918

N36900 X-1.50431 Y4.27488 Z5.26938

N36910 X-1.45252 Y4.27488 Z5.25972

N36920 X-1.40066 Y4.27488 Z5.25158

N36930 X-1.37183 Y4.27488 Z5.24737

N36940 X-1.34069 Y4.27488 Z5.24353

N36950 X-1.31763 Y4.27488 Z5.24101

N36960 X-1.29458 Y4.27488 Z5.23946

N36970 X-1.25969 Y4.27488 Z5.23762

N36980 X-1.22992 Y4.27488 Z5.23632

N36990 X-1.17535 Y4.27488 Z5.23497

N37000 X-1.10607 Y4.27488 Z5.2339

N37010 X-1.09418 Y4.27488 Z5.23385

N37020 X-1.02901 Y4.27488 Z5.23633

N37030 X-0.979362 Y4.27488 Z5.2407

N37040 X-0.963843 Y4.27488 Z5.24216

N37050 X-0.948937 Y4.27488 Z5.24406

N37060 X-0.896123 Y4.27488 Z5.25187

N37070 X-0.847696 Y4.27488 Z5.26083

N37080 X-0.795348 Y4.27488 Z5.27376

N37090 X-0.760307 Y4.27488 Z5.28402

N37100 X-0.744349 Y4.27488 Z5.28884

N37110 X-0.7106 Y4.27488 Z5.3003

N37120 X-0.686155 Y4.27488 Z5.30905

N37130 X-0.649873 Y4.27488 Z5.32358

N37140 X-0.619103 Y4.27488 Z5.33687

N37150 X-0.599052 Y4.27488 Z5.34596

N37160 X-0.57956 Y4.27488 Z5.35547

N37170 X-0.54436 Y4.27488 Z5.37286

N37180 X-0.489222 Y4.27488 Z5.40342

N37190 X-0.474251 Y4.27488 Z5.41134

N37200 X-0.399908 Y4.27488 Z5.45683

N37210 X-0.384845 Y4.27488 Z5.46592

N37220 X-0.29436 Y4.27488 Z5.52493

N37230 X-0.260207 Y4.27488 Z5.54701

N37240 X-0.184987 Y4.27488 Z5.59661

N37250 X-0.0941156 Y4.27488 Z5.65633

N37260 X-0.0794761 Y4.27488 Z5.66583

N37270 X0.0281374 Y4.27488 Z5.73514

N37280 X0.08176 Y4.27488 Z5.77075

N37290 X0.124117 Y4.27488 Z5.7979

N37300 X0.188508 Y4.27488 Z5.83848

N37310 X0.218673 Y4.27488 Z5.85722

N37320 X0.236095 Y4.27488 Z5.86776

N37330 X0.320798 Y4.27488 Z5.9161

N37340 X0.374501 Y4.27488 Z5.94668

N37350 X0.446894 Y4.27488 Z5.98673

N37360 X0.488011 Y4.27488 Z6.00815

N37370 X0.574994 Y4.27488 Z6.05249

N37380 X0.587291 Y4.27488 Z6.05827

N37390 X0.606931 Y4.27488 Z6.06753

N37400 X0.686805 Y4.27488 Z6.10212

N37410 X0.76336 Y4.27488 Z6.13147

N37420 X0.807426 Y4.27488 Z6.14758

N37430 X0.923458 Y4.27488 Z6.18671

N37440 X0.943004 Y4.27488 Z6.19305

N37450 X0.991086 Y4.27488 Z6.20787

N37460 X1.08354 Y4.27488 Z6.23593

N37470 X1.12751 Y4.27488 Z6.2486

N37480 X1.22845 Y4.27488 Z6.27744

N37490 X1.35425 Y4.27488 Z6.31082

N37500 X1.39631 Y4.27488 Z6.32243

N37510 X1.47079 Y4.27488 Z6.34184

N37520 X1.54226 Y4.27488 Z6.35938

N37530 X1.57262 Y4.27488 Z6.36663

N37540 X1.646 Y4.27488 Z6.38505

N37550 X1.70243 Y4.27488 Z6.39974

N37560 X1.73168 Y4.27488 Z6.40779

N37570 X1.79979 Y4.27488 Z6.42824

N37580 X1.82108 Y4.27488 Z6.43405

N37590 X1.84085 Y4.27488 Z6.43996

N37600 X1.92606 Y4.27488 Z6.46557

N37610 X1.96983 Y4.27488 Z6.47985

N37620 X2.04877 Y4.27488 Z6.50189

N37630 X2.10416 Y4.27488 Z6.51879

N37640 X2.16878 Y4.27488 Z6.53526

N37650 X2.22549 Y4.27488 Z6.55181

N37660 X2.26597 Y4.27488 Z6.56161

N37670 X2.32009 Y4.27488 Z6.57586

N37680 X2.3885 Y4.27488 Z6.59285

N37690 X2.42447 Y4.27488 Z6.60245

N37700 X2.47884 Y4.27488 Z6.61861

N37710 X2.51039 Y4.27488 Z6.62671

N37720 X2.5343 Y4.27488 Z6.63315

N37730 X2.59813 Y4.27488 Z6.64971

N37740 X2.69821 Y4.27488 Z6.67153

N37750 X2.71604 Y4.27488 Z6.67396

N37760 X2.82816 Y4.27488 Z6.68599

N37770 X2.85178 Y4.27488 Z6.68826

N37780 X2.97476 Y4.27488 Z6.69281

N37790 X2.9856 Y4.27488 Z6.69329

N37800 X3.097 Y4.27488 Z6.68898

N37810 X3.11563 Y4.27488 Z6.68849

N37820 X3.20153 Y4.27488 Z6.67904

N37830 X3.26555 Y4.27488 Z6.66911

N37840 X3.29977 Y4.27488 Z6.66164

N37850 X3.3323 Y4.27488 Z6.65346

N37860 X3.39727 Y4.27488 Z6.6356

N37870 X3.45994 Y4.27488 Z6.61713

N37880 X3.49403 Y4.27488 Z6.6047

N37890 X3.53171 Y4.27488 Z6.59056

N37900 X3.58671 Y4.27488 Z6.56853

N37910 X3.63652 Y4.27488 Z6.55008

N37920 X3.67915 Y4.27488 Z6.52965

N37930 X3.74293 Y4.27488 Z6.50163

N37940 X3.78381 Y4.27488 Z6.48293

N37950 X3.80603 Y4.27488 Z6.47546

N37960 X3.92405 Y4.27488 Z6.42109

N37970 X4.02738 Y4.27488 Z6.36471

N37980 X4.11231 Y4.27488 Z6.31187

N37990 X4.18841 Y4.27488 Z6.25547

N38000 X4.23831 Y4.27488 Z6.2167

N38010 X4.25663 Y4.27488 Z6.20013

N38020 X4.30368 Y4.27488 Z6.15806

N38030 X4.32554 Y4.27488 Z6.13826

N38040 X4.33314 Y4.27488 Z6.13145

N38050 X4.40572 Y4.27488 Z6.05618

N38060 X4.4203 Y4.27488 Z6.03958

N38070 X4.47692 Y4.27488 Z5.96968

N38080 X4.53067 Y4.27488 Z5.89479

N38090 X4.54555 Y4.27488 Z5.87359

N38100 X4.60285 Y4.27488 Z5.78287

N38110 X4.62338 Y4.27488 Z5.75001

N38120 X4.64809 Y4.27488 Z5.70729

N38130 X4.71296 Y4.27488 Z5.59303

N38140 X4.72685 Y4.27488 Z5.56745

N38150 X4.7976 Y4.27488 Z5.43066

N38160 X4.80904 Y4.27488 Z5.40819

N38170 X4.89264 Y4.27488 Z5.2352

N38180 X4.9521 Y4.27488 Z5.11052

N38190 X5.05251 Y4.27488 Z4.88552

N38200 X5.0974 Y4.27488 Z4.78625

N38210 X5.12397 Y4.27488 Z4.72863

N38220 X5.27203 Y4.18191 Z4.64192

N38230 X5.26658 Y4.18191 Z4.65391

N38240 X5.25205 Y4.18191 Z4.6863

N38250 X5.22497 Y4.18191 Z4.7449

N38260 X5.21395 Y4.18191 Z4.76996

N38270 X5.13274 Y4.18191 Z4.96146

N38280 X5.05514 Y4.18191 Z5.13391

N38290 X5.00646 Y4.18191 Z5.23812

N38300 X4.97303 Y4.18191 Z5.30693

N38310 X4.94226 Y4.18191 Z5.36796

N38320 X4.87895 Y4.18191 Z5.49113

N38330 X4.84196 Y4.18191 Z5.56381

N38340 X4.77716 Y4.18191 Z5.68555

N38350 X4.75126 Y4.18191 Z5.73057

N38360 X4.68604 Y4.18191 Z5.84053

N38370 X4.67132 Y4.18191 Z5.86407

N38380 X4.61199 Y4.18191 Z5.95229

N38390 X4.59252 Y4.18191 Z5.97977

N38400 X4.54444 Y4.18191 Z6.04179

N38410 X4.5125 Y4.18191 Z6.08122

N38420 X4.48555 Y4.18191 Z6.11091

N38430 X4.43936 Y4.18191 Z6.15858

N38440 X4.41729 Y4.18191 Z6.17916

N38450 X4.34978 Y4.18191 Z6.24011

N38460 X4.34091 Y4.18191 Z6.24716

N38470 X4.28203 Y4.18191 Z6.29309

N38480 X4.25334 Y4.18191 Z6.31548

N38490 X4.2123 Y4.18191 Z6.34274

N38500 X4.14563 Y4.18191 Z6.38606

N38510 X4.13243 Y4.18191 Z6.3944

N38520 X4.04887 Y4.18191 Z6.4382

N38530 X3.95335 Y4.18191 Z6.48282

N38540 X3.92276 Y4.18191 Z6.49492

N38550 X3.83443 Y4.18191 Z6.5274

N38560 X3.79582 Y4.18191 Z6.54137

N38570 X3.73653 Y4.18191 Z6.5613

N38580 X3.71907 Y4.18191 Z6.56929

N38590 X3.64986 Y4.18191 Z6.59102

N38600 X3.56591 Y4.18191 Z6.62405

N38610 X3.47784 Y4.18191 Z6.65108

N38620 X3.40349 Y4.18191 Z6.67502

N38630 X3.38724 Y4.18191 Z6.67898

N38640 X3.36237 Y4.18191 Z6.68473

N38650 X3.28685 Y4.18191 Z6.70082

N38660 X3.21509 Y4.18191 Z6.71225

N38670 X3.1727 Y4.18191 Z6.71697

N38680 X3.11121 Y4.18191 Z6.71914

N38690 X3.0528 Y4.18191 Z6.72068

N38700 X3.0295 Y4.18191 Z6.72158

N38710 X2.91081 Y4.18191 Z6.71642

N38720 X2.89211 Y4.18191 Z6.71572

N38730 X2.77675 Y4.18191 Z6.70455

N38740 X2.674 Y4.18191 Z6.68837

N38750 X2.66128 Y4.18191 Z6.68562

N38760 X2.59016 Y4.18191 Z6.66779

N38770 X2.55033 Y4.18191 Z6.65692

N38780 X2.50578 Y4.18191 Z6.64389

N38790 X2.42046 Y4.18191 Z6.61991

N38800 X2.34124 Y4.18191 Z6.59665

N38810 X2.29791 Y4.18191 Z6.58349

N38820 X2.25186 Y4.18191 Z6.57051

N38830 X2.2 Y4.18191 Z6.55565

N38840 X2.15661 Y4.18191 Z6.54133

N38850 X2.06011 Y4.18191 Z6.51199

N38860 X2.02768 Y4.18191 Z6.50064

N38870 X1.92875 Y4.18191 Z6.46841

N38880 X1.90181 Y4.18191 Z6.45854

N38890 X1.84496 Y4.18191 Z6.43927

N38900 X1.80284 Y4.18191 Z6.42505

N38910 X1.7627 Y4.18191 Z6.41249

N38920 X1.71549 Y4.18191 Z6.3967

N38930 X1.69605 Y4.18191 Z6.39062

N38940 X1.6327 Y4.18191 Z6.37188

N38950 X1.57814 Y4.18191 Z6.35698

N38960 X1.55754 Y4.18191 Z6.3515

N38970 X1.44876 Y4.18191 Z6.32146

N38980 X1.31107 Y4.18191 Z6.2823

N38990 X1.24999 Y4.18191 Z6.26402

N39000 X1.16913 Y4.18191 Z6.24048

N39010 X1.02698 Y4.18191 Z6.19693

N39020 X0.885529 Y4.18191 Z6.15037

N39030 X0.844925 Y4.18191 Z6.13591

N39040 X0.744531 Y4.18191 Z6.09893

N39050 X0.674755 Y4.18191 Z6.07003

N39060 X0.617633 Y4.18191 Z6.04469

N39070 X0.572804 Y4.18191 Z6.02277

N39080 X0.497274 Y4.18191 Z5.98602

N39090 X0.412273 Y4.18191 Z5.93986

N39100 X0.380033 Y4.18191 Z5.92203

N39110 X0.343066 Y4.18191 Z5.90123

N39120 X0.26644 Y4.18191 Z5.85627

N39130 X0.203546 Y4.18191 Z5.8188

N39140 X0.174847 Y4.18191 Z5.80126

N39150 X0.103766 Y4.18191 Z5.7546

N39160 X-0.0937073 Y4.18191 Z5.6265

N39170 X-0.114017 Y4.18191 Z5.61309

N39180 X-0.125609 Y4.18191 Z5.60543

N39190 X-0.228798 Y4.18191 Z5.53651

N39200 X-0.276452 Y4.18191 Z5.50455

N39210 X-0.346672 Y4.18191 Z5.45856

N39220 X-0.394842 Y4.18191 Z5.42719

N39230 X-0.453182 Y4.18191 Z5.39149

N39240 X-0.492988 Y4.18191 Z5.36735

N39250 X-0.534261 Y4.18191 Z5.34438

N39260 X-0.564201 Y4.18191 Z5.32818

N39270 X-0.623738 Y4.18191 Z5.29891

N39280 X-0.639575 Y4.18191 Z5.29194

N39290 X-0.683023 Y4.18191 Z5.27312

N39300 X-0.708239 Y4.18191 Z5.26251

N39310 X-0.727135 Y4.18191 Z5.25527

N39320 X-0.757906 Y4.18191 Z5.24478

N39330 X-0.778573 Y4.18191 Z5.23817

N39340 X-0.811461 Y4.18191 Z5.22856

N39350 X-0.863236 Y4.18191 Z5.21545

N39360 X-0.878097 Y4.18191 Z5.21253

N39370 X-0.916756 Y4.18191 Z5.2055

N39380 X-0.950725 Y4.18191 Z5.20059

N39390 X-0.978409 Y4.18191 Z5.19707

N39400 X-1.01163 Y4.18191 Z5.19394

N39410 X-1.04325 Y4.18191 Z5.19167

N39420 X-1.08201 Y4.18191 Z5.19038

N39430 X-1.11119 Y4.18191 Z5.18983

N39440 X-1.13916 Y4.18191 Z5.18995

N39450 X-1.19664 Y4.18191 Z5.19186

N39460 X-1.26248 Y4.18191 Z5.19409

N39470 X-1.2894 Y4.18191 Z5.1958

N39480 X-1.34065 Y4.18191 Z5.20042

N39490 X-1.37261 Y4.18191 Z5.20407

N39500 X-1.41952 Y4.18191 Z5.21115

N39510 X-1.46207 Y4.18191 Z5.21757

N39520 X-1.50984 Y4.18191 Z5.22654

N39530 X-1.53601 Y4.18191 Z5.23215

N39540 X-1.59663 Y4.18191 Z5.24583

N39550 X-1.62345 Y4.18191 Z5.25208

N39560 X-1.6387 Y4.18191 Z5.25596

N39570 X-1.72266 Y4.18191 Z5.27835

N39580 X-1.78665 Y4.18191 Z5.29666

N39590 X-1.83922 Y4.18191 Z5.31223

N39600 X-1.96271 Y4.18191 Z5.35039

N39610 X-1.97294 Y4.18191 Z5.35367

N39620 X-2.01191 Y4.18191 Z5.36643

N39630 X-2.12186 Y4.18191 Z5.40249

N39640 X-2.14854 Y4.18191 Z5.41104

N39650 X-2.29203 Y4.18191 Z5.45986

N39660 X-2.33388 Y4.18191 Z5.47337

N39670 X-2.42099 Y4.18191 Z5.50232

N39680 X-2.47542 Y4.18191 Z5.52092

N39690 X-2.50937 Y4.18191 Z5.53226

N39700 X-2.59174 Y4.18191 Z5.559

N39710 X-2.68213 Y4.18191 Z5.58826

N39720 X-2.72791 Y4.18191 Z5.60244

N39730 X-2.84359 Y4.18191 Z5.63856

N39740 X-2.89022 Y4.18191 Z5.65326

N39750 X-2.90767 Y4.18191 Z5.6589

N39760 X-3.04818 Y4.18191 Z5.70278

N39770 X-3.14435 Y4.18191 Z5.73387

N39780 X-3.20106 Y4.18191 Z5.7513

N39790 X-3.29843 Y4.18191 Z5.7829

N39800 X-3.40027 Y4.18191 Z5.81397

N39810 X-3.43589 Y4.18191 Z5.82533

N39820 X-3.46403 Y4.18191 Z5.83361

N39830 X-3.56032 Y4.18191 Z5.86001

N39840 X-3.64569 Y4.18191 Z5.88086

N39850 X-3.66798 Y4.18191 Z5.88571

N39860 X-3.70031 Y4.18191 Z5.89225

N39870 X-3.75823 Y4.18191 Z5.90458

N39880 X-3.81034 Y4.18191 Z5.91453

N39890 X-3.83963 Y4.18191 Z5.92003

N39900 X-3.85536 Y4.18191 Z5.92263

N39910 X-3.93141 Y4.18191 Z5.9362

N39920 X-3.96244 Y4.18191 Z5.94066

N39930 X-4.03934 Y4.18191 Z5.9532

N39940 X-4.07306 Y4.18191 Z5.95719

N39950 X-4.15012 Y4.18191 Z5.96829

N39960 X-4.19623 Y4.18191 Z5.97241

N39970 X-4.2565 Y4.18191 Z5.97925

N39980 X-4.34461 Y4.18191 Z5.98383

N39990 X-4.36099 Y4.18191 Z5.98491

N40000 X-4.38126 Y4.18191 Z5.98509

N40010 X-4.4666 Y4.18191 Z5.98467

N40020 X-4.57143 Y4.18191 Z5.97823

N40030 X-4.59111 Y4.18191 Z5.97679

N40040 X-4.70824 Y4.18191 Z5.96091

N40050 X-4.81493 Y4.18191 Z5.9418

N40060 X-4.82881 Y4.18191 Z5.93831

N40070 X-4.93273 Y4.18191 Z5.9116

N40080 X-5.02016 Y4.18191 Z5.88332

N40090 X-5.04088 Y4.18191 Z5.87639

N40100 X-5.1317 Y4.18191 Z5.83932

N40110 X-5.22552 Y4.18191 Z5.799

N40120 X-5.24051 Y4.18191 Z5.79122

N40130 X-5.33141 Y4.18191 Z5.74246

N40140 X-5.38924 Y4.18191 Z5.70923

N40150 X-5.42379 Y4.18191 Z5.68648

N40160 X-5.49343 Y4.18191 Z5.63917

N40170 X-5.51077 Y4.18191 Z5.62652

N40180 X-5.58172 Y4.18191 Z5.56733

N40190 X-5.62463 Y4.18191 Z5.52697

N40200 X-5.64613 Y4.18191 Z5.50465

N40210 X-5.66007 Y4.18191 Z5.48965

N40220 X-5.70436 Y4.18191 Z5.43807

N40230 X-5.75147 Y4.18191 Z5.38307

N40240 X-5.75987 Y4.18191 Z5.37245

N40250 X-5.78712 Y4.18191 Z5.33431

N40260 X-5.81767 Y4.18191 Z5.28931

N40270 X-5.82381 Y4.18191 Z5.278

N40280 X-5.86998 Y4.18191 Z5.19409

N40290 X-5.8795 Y4.18191 Z5.17376

N40300 X-5.91454 Y4.18191 Z5.09311

N40310 X-5.99285 Y4.18191 Z4.87919

N40320 X-6.00689 Y4.18191 Z4.837

N40330 X-6.03376 Y4.18191 Z4.74875

N40340 X-6.04874 Y4.18191 Z4.69594

N40350 X-6.05617 Y4.18191 Z4.67549

N40360 X-6.09829 Y4.18191 Z4.55527

N40370 X-6.16708 Y4.08706 Z4.53906

N40380 X-6.15295 Y4.08706 Z4.57864

N40390 X-6.11312 Y4.08706 Z4.68639

N40400 X-6.07699 Y4.08706 Z4.81041

N40410 X-6.07026 Y4.08706 Z4.8325

N40420 X-5.96868 Y4.08706 Z5.10765

N40430 X-5.9462 Y4.08706 Z5.15733

N40440 X-5.92109 Y4.08706 Z5.21125

N40450 X-5.87879 Y4.08706 Z5.28739

N40460 X-5.86614 Y4.08706 Z5.30983

N40470 X-5.8087 Y4.08706 Z5.39224

N40480 X-5.79516 Y4.08706 Z5.4099

N40490 X-5.74387 Y4.08706 Z5.47057

N40500 X-5.71128 Y4.08706 Z5.50667

N40510 X-5.67878 Y4.08706 Z5.54088

N40520 X-5.63649 Y4.08706 Z5.58244

N40530 X-5.60365 Y4.08706 Z5.61278

N40540 X-5.54045 Y4.08706 Z5.66418

N40550 X-5.51862 Y4.08706 Z5.68126

N40560 X-5.50864 Y4.08706 Z5.68833

N40570 X-5.42698 Y4.08706 Z5.74233

N40580 X-5.36353 Y4.08706 Z5.77992

N40590 X-5.32721 Y4.08706 Z5.79988

N40600 X-5.27474 Y4.08706 Z5.82505

N40610 X-5.22242 Y4.08706 Z5.84977

N40620 X-5.17638 Y4.08706 Z5.86957

N40630 X-5.11596 Y4.08706 Z5.89252

N40640 X-5.04768 Y4.08706 Z5.91576

N40650 X-5.00798 Y4.08706 Z5.92938

N40660 X-4.98302 Y4.08706 Z5.93757

N40670 X-4.89337 Y4.08706 Z5.96043

N40680 X-4.85209 Y4.08706 Z5.97012

N40690 X-4.73382 Y4.08706 Z5.98998

N40700 X-4.71252 Y4.08706 Z5.9938

N40710 X-4.69136 Y4.08706 Z5.99666

N40720 X-4.57908 Y4.08706 Z6.00779

N40730 X-4.49423 Y4.08706 Z6.01243

N40740 X-4.46712 Y4.08706 Z6.01322

N40750 X-4.43632 Y4.08706 Z6.01275

N40760 X-4.36401 Y4.08706 Z6.01212

N40770 X-4.28361 Y4.08706 Z6.0068

N40780 X-4.26481 Y4.08706 Z6.00545

N40790 X-4.24803 Y4.08706 Z6.00337

N40800 X-4.16159 Y4.08706 Z5.99356

N40810 X-4.08675 Y4.08706 Z5.98148

N40820 X-4.05442 Y4.08706 Z5.97685

N40830 X-3.95023 Y4.08706 Z5.95779

N40840 X-3.93887 Y4.08706 Z5.95563

N40850 X-3.8301 Y4.08706 Z5.93449

N40860 X-3.78124 Y4.08706 Z5.92338

N40870 X-3.71918 Y4.08706 Z5.91051

N40880 X-3.64181 Y4.08706 Z5.89188

N40890 X-3.60062 Y4.08706 Z5.88162

N40900 X-3.50189 Y4.08706 Z5.85307

N40910 X-3.46683 Y4.08706 Z5.8433

N40920 X-3.45243 Y4.08706 Z5.8389

N40930 X-3.33123 Y4.08706 Z5.79982

N40940 X-3.24814 Y4.08706 Z5.77191

N40950 X-3.2036 Y4.08706 Z5.75672

N40960 X-3.16671 Y4.08706 Z5.74373

N40970 X-3.07406 Y4.08706 Z5.71229

N40980 X-2.98133 Y4.08706 Z5.67997

N40990 X-2.95343 Y4.08706 Z5.67058

N41000 X-2.88519 Y4.08706 Z5.64725

N41010 X-2.83918 Y4.08706 Z5.63132

N41020 X-2.81841 Y4.08706 Z5.62385

N41030 X-2.70799 Y4.08706 Z5.58619

N41040 X-2.62186 Y4.08706 Z5.55511

N41050 X-2.55966 Y4.08706 Z5.53416

N41060 X-2.45246 Y4.08706 Z5.49507

N41070 X-2.42909 Y4.08706 Z5.48698

N41080 X-2.36485 Y4.08706 Z5.46347

N41090 X-2.29478 Y4.08706 Z5.43769

N41100 X-2.26201 Y4.08706 Z5.42546

N41110 X-2.14655 Y4.08706 Z5.3839

N41120 X-2.05039 Y4.08706 Z5.34961

N41130 X-1.99158 Y4.08706 Z5.32943

N41140 X-1.85844 Y4.08706 Z5.28506

N41150 X-1.84652 Y4.08706 Z5.28124

N41160 X-1.80955 Y4.08706 Z5.27

N41170 X-1.72123 Y4.08706 Z5.24325

N41180 X-1.70146 Y4.08706 Z5.23776

N41190 X-1.61805 Y4.08706 Z5.21551

N41200 X-1.51808 Y4.08706 Z5.19247

N41210 X-1.4858 Y4.08706 Z5.18592

N41220 X-1.42979 Y4.08706 Z5.17535

N41230 X-1.36838 Y4.08706 Z5.16593

N41240 X-1.35838 Y4.08706 Z5.16453

N41250 X-1.34209 Y4.08706 Z5.16274

N41260 X-1.28512 Y4.08706 Z5.15692

N41270 X-1.25014 Y4.08706 Z5.15454

N41280 X-1.19757 Y4.08706 Z5.15147

N41290 X-1.14736 Y4.08706 Z5.14951

N41300 X-1.11322 Y4.08706 Z5.14874

N41310 X-1.07908 Y4.08706 Z5.14906

N41320 X-1.04627 Y4.08706 Z5.15

N41330 X-1.02169 Y4.08706 Z5.1517

N41340 X-0.984257 Y4.08706 Z5.15507

N41350 X-0.955145 Y4.08706 Z5.15874

N41360 X-0.926971 Y4.08706 Z5.1629

N41370 X-0.894146 Y4.08706 Z5.16911

N41380 X-0.87593 Y4.08706 Z5.17284

N41390 X-0.825148 Y4.08706 Z5.1866

N41400 X-0.799989 Y4.08706 Z5.19456

N41410 X-0.721097 Y4.08706 Z5.22386

N41420 X-0.651437 Y4.08706 Z5.25242

N41430 X-0.63954 Y4.08706 Z5.25789

N41440 X-0.586494 Y4.08706 Z5.28283

N41450 X-0.570278 Y4.08706 Z5.2912

N41460 X-0.514257 Y4.08706 Z5.32148

N41470 X-0.501977 Y4.08706 Z5.32845

N41480 X-0.467508 Y4.08706 Z5.34933

N41490 X-0.416705 Y4.08706 Z5.38014

N41500 X-0.40161 Y4.08706 Z5.38937

N41510 X-0.287489 Y4.08706 Z5.4637

N41520 X-0.155202 Y4.08706 Z5.55235

N41530 X-0.0396483 Y4.08706 Z5.62879

N41540 X0.0224658 Y4.08706 Z5.66978

N41550 X0.070289 Y4.08706 Z5.69993

N41560 X0.187504 Y4.08706 Z5.77534

N41570 X0.307258 Y4.08706 Z5.8474

N41580 X0.364173 Y4.08706 Z5.88032

N41590 X0.417549 Y4.08706 Z5.91035

N41600 X0.475592 Y4.08706 Z5.94245

N41610 X0.519156 Y4.08706 Z5.96566

N41620 X0.549405 Y4.08706 Z5.98043

N41630 X0.631186 Y4.08706 Z6.01974

N41640 X0.692769 Y4.08706 Z6.0462

N41650 X0.773653 Y4.08706 Z6.07956

N41660 X0.828019 Y4.08706 Z6.10028

N41670 X0.939026 Y4.08706 Z6.13995

N41680 X0.968861 Y4.08706 Z6.15054

N41690 X1.03968 Y4.08706 Z6.17431

N41700 X1.10561 Y4.08706 Z6.19606

N41710 X1.1417 Y4.08706 Z6.20736

N41720 X1.23507 Y4.08706 Z6.23738

N41730 X1.35568 Y4.08706 Z6.27456

N41740 X1.37476 Y4.08706 Z6.28079

N41750 X1.49621 Y4.08706 Z6.31782

N41760 X1.50763 Y4.08706 Z6.32127

N41770 X1.60324 Y4.08706 Z6.34994

N41780 X1.68087 Y4.08706 Z6.37562

N41790 X1.69618 Y4.08706 Z6.38113

N41800 X1.76077 Y4.08706 Z6.40422

N41810 X1.77573 Y4.08706 Z6.40946

N41820 X1.78741 Y4.08706 Z6.41375

N41830 X1.85685 Y4.08706 Z6.43917

N41840 X1.88162 Y4.08706 Z6.4489

N41850 X1.99006 Y4.08706 Z6.48869

N41860 X2.00517 Y4.08706 Z6.49457

N41870 X2.04276 Y4.08706 Z6.50796

N41880 X2.1383 Y4.08706 Z6.54138

N41890 X2.16395 Y4.08706 Z6.54918

N41900 X2.20268 Y4.08706 Z6.56195

N41910 X2.28722 Y4.08706 Z6.59032

N41920 X2.34272 Y4.08706 Z6.60784

N41930 X2.39371 Y4.08706 Z6.62411

N41940 X2.4276 Y4.08706 Z6.63365

N41950 X2.45565 Y4.08706 Z6.6424

N41960 X2.51584 Y4.08706 Z6.65996

N41970 X2.5789 Y4.08706 Z6.67838

N41980 X2.60421 Y4.08706 Z6.6853

N41990 X2.68179 Y4.08706 Z6.70292

N42000 X2.70529 Y4.08706 Z6.70809

N42010 X2.80187 Y4.08706 Z6.72188

N42020 X2.85184 Y4.08706 Z6.72721

N42030 X2.92655 Y4.08706 Z6.73695

N42040 X2.98897 Y4.08706 Z6.73999

N42050 X3.05021 Y4.08706 Z6.74434

N42060 X3.14611 Y4.08706 Z6.74506

N42070 X3.16053 Y4.08706 Z6.74504

N42080 X3.17756 Y4.08706 Z6.74363

N42090 X3.27125 Y4.08706 Z6.73221

N42100 X3.34606 Y4.08706 Z6.71935

N42110 X3.36731 Y4.08706 Z6.71443

N42120 X3.3944 Y4.08706 Z6.70783

N42130 X3.45379 Y4.08706 Z6.69198

N42140 X3.49426 Y4.08706 Z6.68235

N42150 X3.53466 Y4.08706 Z6.66884

N42160 X3.58226 Y4.08706 Z6.65556

N42170 X3.61222 Y4.08706 Z6.64641

N42180 X3.63756 Y4.08706 Z6.64068

N42190 X3.68938 Y4.08706 Z6.62275

N42200 X3.76603 Y4.08706 Z6.60422

N42210 X3.7924 Y4.08706 Z6.59661

N42220 X3.86 Y4.08706 Z6.57703

N42230 X3.87516 Y4.08706 Z6.57381

N42240 X3.96388 Y4.08706 Z6.54606

N42250 X4.05086 Y4.08706 Z6.5125

N42260 X4.13584 Y4.08706 Z6.47634

N42270 X4.21663 Y4.08706 Z6.43247

N42280 X4.26095 Y4.08706 Z6.40782

N42290 X4.29206 Y4.08706 Z6.38693

N42300 X4.35812 Y4.08706 Z6.34218

N42310 X4.37007 Y4.08706 Z6.3339

N42320 X4.43661 Y4.08706 Z6.27971

N42330 X4.46591 Y4.08706 Z6.2553

N42340 X4.50943 Y4.08706 Z6.2143

N42350 X4.5532 Y4.08706 Z6.17142

N42360 X4.58218 Y4.08706 Z6.13935

N42370 X4.63569 Y4.08706 Z6.07809

N42380 X4.65434 Y4.08706 Z6.05382

N42390 X4.7122 Y4.08706 Z5.97689

N42400 X4.72895 Y4.08706 Z5.9519

N42410 X4.78518 Y4.08706 Z5.86585

N42420 X4.81096 Y4.08706 Z5.82279

N42430 X4.85862 Y4.08706 Z5.74007

N42440 X4.91893 Y4.08706 Z5.62818

N42450 X4.94709 Y4.08706 Z5.57402

N42460 X4.96707 Y4.08706 Z5.53595

N42470 X5.05875 Y4.08706 Z5.35902

N42480 X5.14 Y4.08706 Z5.18752

N42490 X5.21479 Y4.08706 Z5.02465

N42500 X5.30586 Y4.08706 Z4.81073

N42510 X5.3293 Y4.08706 Z4.7536

N42520 X5.34904 Y4.08706 Z4.70845

N42530 X5.36692 Y4.08706 Z4.66507

N42540 X5.39923 Y4.08706 Z4.59389

N42550 X5.41734 Y4.08706 Z4.55335

N42560 X5.5607 Y3.98999 Z4.45937

N42570 X5.5241 Y3.98999 Z4.54265

N42580 X5.49147 Y3.98999 Z4.61644

N42590 X5.46432 Y3.98999 Z4.68327

N42600 X5.43436 Y3.98999 Z4.75463

N42610 X5.38696 Y3.98999 Z4.87087

N42620 X5.30031 Y3.98999 Z5.06942

N42630 X5.27797 Y3.98999 Z5.11892

N42640 X5.22524 Y3.98999 Z5.23045

N42650 X5.17665 Y3.98999 Z5.33159

N42660 X5.15363 Y3.98999 Z5.37834

N42670 X5.0947 Y3.98999 Z5.49671

N42680 X5.08075 Y3.98999 Z5.52398

N42690 X5.06556 Y3.98999 Z5.5537

N42700 X5.01182 Y3.98999 Z5.65611

N42710 X4.99533 Y3.98999 Z5.68783

N42720 X4.94683 Y3.98999 Z5.77547

N42730 X4.91427 Y3.98999 Z5.83161

N42740 X4.88011 Y3.98999 Z5.88953

N42750 X4.82983 Y3.98999 Z5.96675

N42760 X4.81185 Y3.98999 Z5.994

N42770 X4.75215 Y3.98999 Z6.07437

N42780 X4.73882 Y3.98999 Z6.09211

N42790 X4.67566 Y3.98999 Z6.16593

N42800 X4.65941 Y3.98999 Z6.18465

N42810 X4.59861 Y3.98999 Z6.24596

N42820 X4.57273 Y3.98999 Z6.27156

N42830 X4.52042 Y3.98999 Z6.31665

N42840 X4.47798 Y3.98999 Z6.35253

N42850 X4.44167 Y3.98999 Z6.37884

N42860 X4.37343 Y3.98999 Z6.42743

N42870 X4.36299 Y3.98999 Z6.43364

N42880 X4.33725 Y3.98999 Z6.44862

N42890 X4.28072 Y3.98999 Z6.48106

N42900 X4.25637 Y3.98999 Z6.49474

N42910 X4.19795 Y3.98999 Z6.52151

N42920 X4.12733 Y3.98999 Z6.55263

N42930 X4.11532 Y3.98999 Z6.55692

N42940 X4.09858 Y3.98999 Z6.56246

N42950 X4.03173 Y3.98999 Z6.58508

N42960 X3.97126 Y3.98999 Z6.60374

N42970 X3.95164 Y3.98999 Z6.60863

N42980 X3.93596 Y3.98999 Z6.61199

N42990 X3.87393 Y3.98999 Z6.62671

N43000 X3.817 Y3.98999 Z6.6398

N43010 X3.79848 Y3.98999 Z6.64277

N43020 X3.76872 Y3.98999 Z6.64898

N43030 X3.71759 Y3.98999 Z6.65882

N43040 X3.69175 Y3.98999 Z6.66584

N43050 X3.64037 Y3.98999 Z6.67472

N43060 X3.57941 Y3.98999 Z6.68993

N43070 X3.56096 Y3.98999 Z6.69476

N43080 X3.49676 Y3.98999 Z6.70787

N43090 X3.42161 Y3.98999 Z6.72668

N43100 X3.40965 Y3.98999 Z6.72906

N43110 X3.33828 Y3.98999 Z6.7424

N43120 X3.29445 Y3.98999 Z6.75075

N43130 X3.24914 Y3.98999 Z6.75608

N43140 X3.1601 Y3.98999 Z6.76502

N43150 X3.06968 Y3.98999 Z6.76318

N43160 X3.04588 Y3.98999 Z6.76156

N43170 X2.96212 Y3.98999 Z6.75456

N43180 X2.92759 Y3.98999 Z6.74994

N43190 X2.83492 Y3.98999 Z6.73797

N43200 X2.80363 Y3.98999 Z6.73244

N43210 X2.70195 Y3.98999 Z6.71497

N43220 X2.66405 Y3.98999 Z6.70716

N43230 X2.61052 Y3.98999 Z6.69444

N43240 X2.54622 Y3.98999 Z6.67674

N43250 X2.49849 Y3.98999 Z6.66273

N43260 X2.43595 Y3.98999 Z6.64339

N43270 X2.36475 Y3.98999 Z6.6212

N43280 X2.31809 Y3.98999 Z6.60592

N43290 X2.22022 Y3.98999 Z6.57262

N43300 X2.16765 Y3.98999 Z6.55359

N43310 X2.085 Y3.98999 Z6.52289

N43320 X2.04406 Y3.98999 Z6.50831

N43330 X1.97995 Y3.98999 Z6.48336

N43340 X1.92263 Y3.98999 Z6.4607

N43350 X1.89327 Y3.98999 Z6.44857

N43360 X1.79818 Y3.98999 Z6.41113

N43370 X1.76045 Y3.98999 Z6.39572

N43380 X1.68137 Y3.98999 Z6.36556

N43390 X1.65901 Y3.98999 Z6.35664

N43400 X1.56296 Y3.98999 Z6.32203

N43410 X1.54335 Y3.98999 Z6.31447

N43420 X1.43795 Y3.98999 Z6.27917

N43430 X1.41409 Y3.98999 Z6.27127

N43440 X1.39983 Y3.98999 Z6.26661

N43450 X1.27161 Y3.98999 Z6.22384

N43460 X1.22572 Y3.98999 Z6.20781

N43470 X1.14272 Y3.98999 Z6.18018

N43480 X1.05519 Y3.98999 Z6.15

N43490 X1.03061 Y3.98999 Z6.14128

N43500 X1.01594 Y3.98999 Z6.1359

N43510 X0.905682 Y3.98999 Z6.09652

N43520 X0.860753 Y3.98999 Z6.07957

N43530 X0.783163 Y3.98999 Z6.04997

N43540 X0.733709 Y3.98999 Z6.03015

N43550 X0.671834 Y3.98999 Z6.00363

N43560 X0.634838 Y3.98999 Z5.987

N43570 X0.545566 Y3.98999 Z5.94408

N43580 X0.468993 Y3.98999 Z5.90245

N43590 X0.340769 Y3.98999 Z5.83041

N43600 X0.304237 Y3.98999 Z5.80842

N43610 X0.180589 Y3.98999 Z5.73473

N43620 X0.141131 Y3.98999 Z5.70934

N43630 X0.0175777 Y3.98999 Z5.63145

N43640 X-0.155965 Y3.98999 Z5.51654

N43650 X-0.206237 Y3.98999 Z5.48282

N43660 X-0.280892 Y3.98999 Z5.43322

N43670 X-0.355844 Y3.98999 Z5.38452

N43680 X-0.394504 Y3.98999 Z5.35986

N43690 X-0.434721 Y3.98999 Z5.33506

N43700 X-0.476052 Y3.98999 Z5.31002

N43710 X-0.525851 Y3.98999 Z5.28176

N43720 X-0.53802 Y3.98999 Z5.27504

N43730 X-0.551166 Y3.98999 Z5.26823

N43740 X-0.59484 Y3.98999 Z5.24603

N43750 X-0.652746 Y3.98999 Z5.2192

N43760 X-0.669085 Y3.98999 Z5.21246

N43770 X-0.732817 Y3.98999 Z5.18651

N43780 X-0.761356 Y3.98999 Z5.1753

N43790 X-0.798987 Y3.98999 Z5.16153

N43800 X-0.834542 Y3.98999 Z5.14883

N43810 X-0.866988 Y3.98999 Z5.13949

N43820 X-0.877247 Y3.98999 Z5.13672

N43830 X-0.894322 Y3.98999 Z5.13321

N43840 X-0.91947 Y3.98999 Z5.12843

N43850 X-0.944473 Y3.98999 Z5.1248

N43860 X-0.970323 Y3.98999 Z5.12151

N43870 X-1.01264 Y3.98999 Z5.11766

N43880 X-1.02742 Y3.98999 Z5.11656

N43890 X-1.0409 Y3.98999 Z5.1161

N43900 X-1.0892 Y3.98999 Z5.11471

N43910 X-1.13922 Y3.98999 Z5.11514

N43920 X-1.15515 Y3.98999 Z5.11558

N43930 X-1.17244 Y3.98999 Z5.11655

N43940 X-1.22444 Y3.98999 Z5.11984

N43950 X-1.26694 Y3.98999 Z5.12364

N43960 X-1.29623 Y3.98999 Z5.1267

N43970 X-1.35671 Y3.98999 Z5.13441

N43980 X-1.37805 Y3.98999 Z5.13737

N43990 X-1.45062 Y3.98999 Z5.14978

N44000 X-1.47853 Y3.98999 Z5.15516

N44010 X-1.53296 Y3.98999 Z5.16659

N44020 X-1.59873 Y3.98999 Z5.18236

N44030 X-1.60952 Y3.98999 Z5.18528

N44040 X-1.70953 Y3.98999 Z5.21488

N44050 X-1.84201 Y3.98999 Z5.25659

N44060 X-1.85844 Y3.98999 Z5.26181

N44070 X-1.98754 Y3.98999 Z5.30683

N44080 X-2.02403 Y3.98999 Z5.31965

N44090 X-2.04347 Y3.98999 Z5.32699

N44100 X-2.1686 Y3.98999 Z5.37429

N44110 X-2.20341 Y3.98999 Z5.38806

N44120 X-2.34398 Y3.98999 Z5.44325

N44130 X-2.42841 Y3.98999 Z5.47643

N44140 X-2.49961 Y3.98999 Z5.50455

N44150 X-2.56527 Y3.98999 Z5.52995

N44160 X-2.62572 Y3.98999 Z5.55387

N44170 X-2.70133 Y3.98999 Z5.58321

N44180 X-2.73289 Y3.98999 Z5.59531

N44190 X-2.74756 Y3.98999 Z5.60071

N44200 X-2.83966 Y3.98999 Z5.63606

N44210 X-2.91801 Y3.98999 Z5.66523

N44220 X-2.95835 Y3.98999 Z5.6809

N44230 X-3.01266 Y3.98999 Z5.70145

N44240 X-3.1024 Y3.98999 Z5.73481

N44250 X-3.14922 Y3.98999 Z5.75124

N44260 X-3.24177 Y3.98999 Z5.78489

N44270 X-3.31964 Y3.98999 Z5.81168

N44280 X-3.38594 Y3.98999 Z5.83446

N44290 X-3.51601 Y3.98999 Z5.87501

N44300 X-3.55385 Y3.98999 Z5.88687

N44310 X-3.61116 Y3.98999 Z5.90202

N44320 X-3.70387 Y3.98999 Z5.92701

N44330 X-3.76004 Y3.98999 Z5.93965

N44340 X-3.82866 Y3.98999 Z5.9562

N44350 X-3.94814 Y3.98999 Z5.98115

N44360 X-4.06038 Y3.98999 Z6.00291

N44370 X-4.13339 Y3.98999 Z6.01452

N44380 X-4.16697 Y3.98999 Z6.0201

N44390 X-4.19659 Y3.98999 Z6.0238

N44400 X-4.26669 Y3.98999 Z6.0325

N44410 X-4.33103 Y3.98999 Z6.03713

N44420 X-4.3652 Y3.98999 Z6.03969

N44430 X-4.39498 Y3.98999 Z6.04062

N44440 X-4.46486 Y3.98999 Z6.04167

N44450 X-4.56435 Y3.98999 Z6.03878

N44460 X-4.58192 Y3.98999 Z6.03814

N44470 X-4.70012 Y3.98999 Z6.0271

N44480 X-4.76004 Y3.98999 Z6.02025

N44490 X-4.84408 Y3.98999 Z6.00627

N44500 X-4.91619 Y3.98999 Z5.99183

N44510 X-4.98313 Y3.98999 Z5.97489

N44520 X-5.08121 Y3.98999 Z5.9459

N44530 X-5.10351 Y3.98999 Z5.93954

N44540 X-5.12631 Y3.98999 Z5.93138

N44550 X-5.22026 Y3.98999 Z5.89593

N44560 X-5.31384 Y3.98999 Z5.85415

N44570 X-5.33018 Y3.98999 Z5.84669

N44580 X-5.34089 Y3.98999 Z5.84083

N44590 X-5.43683 Y3.98999 Z5.78646

N44600 X-5.50487 Y3.98999 Z5.74148

N44610 X-5.53488 Y3.98999 Z5.72093

N44620 X-5.58758 Y3.98999 Z5.67946

N44630 X-5.6261 Y3.98999 Z5.64843

N44640 X-5.64766 Y3.98999 Z5.6285

N44650 X-5.70917 Y3.98999 Z5.57003

N44660 X-5.76949 Y3.98999 Z5.50626

N44670 X-5.78184 Y3.98999 Z5.49291

N44680 X-5.80156 Y3.98999 Z5.46931

N44690 X-5.84785 Y3.98999 Z5.41267

N44700 X-5.86236 Y3.98999 Z5.3924

N44710 X-5.91114 Y3.98999 Z5.32437

N44720 X-5.93172 Y3.98999 Z5.28879

N44730 X-5.9611 Y3.98999 Z5.23726

N44740 X-6.00951 Y3.98999 Z5.1401

N44750 X-6.01692 Y3.98999 Z5.12371

N44760 X-6.07941 Y3.98999 Z4.97401

N44770 X-6.12966 Y3.98999 Z4.82977

N44780 X-6.17569 Y3.98999 Z4.68274

N44790 X-6.17878 Y3.98999 Z4.67313

N44800 X-6.23222 Y3.98999 Z4.52338

N44810 X-6.29336 Y3.88964 Z4.51241

N44820 X-6.27306 Y3.88964 Z4.56649

N44830 X-6.24806 Y3.88964 Z4.63414

N44840 X-6.22746 Y3.88964 Z4.70174

N44850 X-6.20034 Y3.88964 Z4.7911

N44860 X-6.18871 Y3.88964 Z4.82783

N44870 X-6.1752 Y3.88964 Z4.86509

N44880 X-6.14039 Y3.88964 Z4.96143

N44890 X-6.10385 Y3.88964 Z5.04899

N44900 X-6.08491 Y3.88964 Z5.09395

N44910 X-6.04482 Y3.88964 Z5.17316

N44920 X-5.99726 Y3.88964 Z5.26459

N44930 X-5.9916 Y3.88964 Z5.27416

N44940 X-5.97849 Y3.88964 Z5.29424

N44950 X-5.92877 Y3.88964 Z5.37212

N44960 X-5.90874 Y3.88964 Z5.39998

N44970 X-5.84369 Y3.88964 Z5.48116

N44980 X-5.82514 Y3.88964 Z5.50335

N44990 X-5.79499 Y3.88964 Z5.53592

N45000 X-5.73783 Y3.88964 Z5.59668

N45010 X-5.64977 Y3.88964 Z5.67842

N45020 X-5.55295 Y3.88964 Z5.75434

N45030 X-5.49249 Y3.88964 Z5.79541

N45040 X-5.45142 Y3.88964 Z5.82258

N45050 X-5.36608 Y3.88964 Z5.87063

N45060 X-5.34214 Y3.88964 Z5.88372

N45070 X-5.32507 Y3.88964 Z5.89152

N45080 X-5.22908 Y3.88964 Z5.93423

N45090 X-5.11333 Y3.88964 Z5.97642

N45100 X-4.9874 Y3.88964 Z6.01225

N45110 X-4.94508 Y3.88964 Z6.02134

N45120 X-4.82476 Y3.88964 Z6.04486

N45130 X-4.80815 Y3.88964 Z6.04709

N45140 X-4.75922 Y3.88964 Z6.05266

N45150 X-4.68254 Y3.88964 Z6.06142

N45160 X-4.65751 Y3.88964 Z6.06376

N45170 X-4.56687 Y3.88964 Z6.06859

N45180 X-4.48139 Y3.88964 Z6.07073

N45190 X-4.46196 Y3.88964 Z6.07073

N45200 X-4.43721 Y3.88964 Z6.06997

N45210 X-4.36259 Y3.88964 Z6.06766

N45220 X-4.30271 Y3.88964 Z6.06316

N45230 X-4.26498 Y3.88964 Z6.05995

N45240 X-4.20927 Y3.88964 Z6.05303

N45250 X-4.16542 Y3.88964 Z6.04755

N45260 X-4.12531 Y3.88964 Z6.04089

N45270 X-4.05988 Y3.88964 Z6.03006

N45280 X-3.99401 Y3.88964 Z6.01697

N45290 X-3.94846 Y3.88964 Z6.00823

N45300 X-3.87494 Y3.88964 Z5.99206

N45310 X-3.83001 Y3.88964 Z5.98213

N45320 X-3.8031 Y3.88964 Z5.97555

N45330 X-3.70505 Y3.88964 Z5.95217

N45340 X-3.58215 Y3.88964 Z5.91842

N45350 X-3.55882 Y3.88964 Z5.91154

N45360 X-3.43181 Y3.88964 Z5.87286

N45370 X-3.35503 Y3.88964 Z5.84703

N45380 X-3.28766 Y3.88964 Z5.82433

N45390 X-3.14696 Y3.88964 Z5.77346

N45400 X-3.10084 Y3.88964 Z5.75584

N45410 X-2.99096 Y3.88964 Z5.71383

N45420 X-2.90015 Y3.88964 Z5.67727

N45430 X-2.82937 Y3.88964 Z5.64889

N45440 X-2.81591 Y3.88964 Z5.64344

N45450 X-2.66489 Y3.88964 Z5.58132

N45460 X-2.63357 Y3.88964 Z5.56854

N45470 X-2.47921 Y3.88964 Z5.50492

N45480 X-2.44483 Y3.88964 Z5.49094

N45490 X-2.30132 Y3.88964 Z5.43194

N45500 X-2.28326 Y3.88964 Z5.42459

N45510 X-2.16745 Y3.88964 Z5.37773

N45520 X-2.03521 Y3.88964 Z5.32406

N45530 X-2.0161 Y3.88964 Z5.31632

N45540 X-2.00498 Y3.88964 Z5.3119

N45550 X-1.90012 Y3.88964 Z5.2712

N45560 X-1.8547 Y3.88964 Z5.25475

N45570 X-1.80563 Y3.88964 Z5.23743

N45580 X-1.74346 Y3.88964 Z5.21729

N45590 X-1.72472 Y3.88964 Z5.2113

N45600 X-1.6484 Y3.88964 Z5.18903

N45610 X-1.63854 Y3.88964 Z5.18629

N45620 X-1.55804 Y3.88964 Z5.16451

N45630 X-1.51757 Y3.88964 Z5.154

N45640 X-1.47516 Y3.88964 Z5.14334

N45650 X-1.42245 Y3.88964 Z5.13141

N45660 X-1.40187 Y3.88964 Z5.12687

N45670 X-1.38807 Y3.88964 Z5.12415

N45680 X-1.33657 Y3.88964 Z5.11492

N45690 X-1.29245 Y3.88964 Z5.10773

N45700 X-1.27182 Y3.88964 Z5.10476

N45710 X-1.23801 Y3.88964 Z5.10038

N45720 X-1.2128 Y3.88964 Z5.09729

N45730 X-1.20207 Y3.88964 Z5.09623

N45740 X-1.15826 Y3.88964 Z5.09257

N45750 X-1.11566 Y3.88964 Z5.09046

N45760 X-1.10165 Y3.88964 Z5.0902

N45770 X-1.05716 Y3.88964 Z5.09107

N45780 X-1.00785 Y3.88964 Z5.0941

N45790 X-0.995498 Y3.88964 Z5.09511

N45800 X-0.942586 Y3.88964 Z5.10167

N45810 X-0.891193 Y3.88964 Z5.10957

N45820 X-0.878441 Y3.88964 Z5.1121

N45830 X-0.836917 Y3.88964 Z5.12118

N45840 X-0.811713 Y3.88964 Z5.12793

N45850 X-0.801342 Y3.88964 Z5.13101

N45860 X-0.782009 Y3.88964 Z5.13787

N45870 X-0.750019 Y3.88964 Z5.14961

N45880 X-0.728765 Y3.88964 Z5.15796

N45890 X-0.67385 Y3.88964 Z5.1805

N45900 X-0.643203 Y3.88964 Z5.19347

N45910 X-0.609141 Y3.88964 Z5.20883

N45920 X-0.578281 Y3.88964 Z5.22362

N45930 X-0.527129 Y3.88964 Z5.24905

N45940 X-0.513556 Y3.88964 Z5.25608

N45950 X-0.496993 Y3.88964 Z5.26523

N45960 X-0.453201 Y3.88964 Z5.28967

N45970 X-0.426163 Y3.88964 Z5.30558

N45980 X-0.379649 Y3.88964 Z5.33426

N45990 X-0.280332 Y3.88964 Z5.39761

N46000 X-0.264713 Y3.88964 Z5.40794

N46010 X-0.155788 Y3.88964 Z5.48003

N46020 X-0.103275 Y3.88964 Z5.5148

N46030 X-0.0504188 Y3.88964 Z5.55013

N46040 X0.0370681 Y3.88964 Z5.60702

N46050 X0.0526009 Y3.88964 Z5.61708

N46060 X0.0861178 Y3.88964 Z5.63821

N46070 X0.173099 Y3.88964 Z5.6921

N46080 X0.217848 Y3.88964 Z5.71877

N46090 X0.34766 Y3.88964 Z5.79612

N46100 X0.39594 Y3.88964 Z5.82294

N46110 X0.525833 Y3.88964 Z5.89435

N46120 X0.647015 Y3.88964 Z5.95467

N46130 X0.674086 Y3.88964 Z5.96664

N46140 X0.747001 Y3.88964 Z5.99893

N46150 X0.793771 Y3.88964 Z6.01771

N46160 X0.852559 Y3.88964 Z6.04156

N46170 X0.911648 Y3.88964 Z6.06389

N46180 X0.953283 Y3.88964 Z6.08011

N46190 X1.03596 Y3.88964 Z6.11058

N46200 X1.06043 Y3.88964 Z6.12002

N46210 X1.12773 Y3.88964 Z6.14442

N46220 X1.25089 Y3.88964 Z6.19021

N46230 X1.26805 Y3.88964 Z6.1962

N46240 X1.28198 Y3.88964 Z6.20085

N46250 X1.41885 Y3.88964 Z6.25173

N46260 X1.52238 Y3.88964 Z6.29125

N46270 X1.54738 Y3.88964 Z6.30174

N46280 X1.61836 Y3.88964 Z6.32986

N46290 X1.6673 Y3.88964 Z6.35057

N46300 X1.73588 Y3.88964 Z6.37858

N46310 X1.79357 Y3.88964 Z6.40322

N46320 X1.90933 Y3.88964 Z6.45151

N46330 X1.927 Y3.88964 Z6.45913

N46340 X2.04521 Y3.88964 Z6.50772

N46350 X2.14958 Y3.88964 Z6.54877

N46360 X2.16932 Y3.88964 Z6.5561

N46370 X2.2573 Y3.88964 Z6.58796

N46380 X2.29665 Y3.88964 Z6.60225

N46390 X2.4282 Y3.88964 Z6.64556

N46400 X2.46437 Y3.88964 Z6.65672

N46410 X2.56015 Y3.88964 Z6.68483

N46420 X2.60876 Y3.88964 Z6.69759

N46430 X2.66725 Y3.88964 Z6.7116

N46440 X2.75533 Y3.88964 Z6.73042

N46450 X2.77124 Y3.88964 Z6.73311

N46460 X2.8503 Y3.88964 Z6.74709

N46470 X2.88031 Y3.88964 Z6.75224

N46480 X2.99378 Y3.88964 Z6.76738

N46490 X3.00402 Y3.88964 Z6.76845

N46500 X3.106 Y3.88964 Z6.77561

N46510 X3.21392 Y3.88964 Z6.77311

N46520 X3.24215 Y3.88964 Z6.77157

N46530 X3.30144 Y3.88964 Z6.76415

N46540 X3.3725 Y3.88964 Z6.75516

N46550 X3.39275 Y3.88964 Z6.75141

N46560 X3.45198 Y3.88964 Z6.7405

N46570 X3.49275 Y3.88964 Z6.7347

N46580 X3.52052 Y3.88964 Z6.72859

N46590 X3.55248 Y3.88964 Z6.72355

N46600 X3.58868 Y3.88964 Z6.71762

N46610 X3.62219 Y3.88964 Z6.71387

N46620 X3.66046 Y3.88964 Z6.7067

N46630 X3.75006 Y3.88964 Z6.69526

N46640 X3.84921 Y3.88964 Z6.68047

N46650 X3.90172 Y3.88964 Z6.67145

N46660 X3.92888 Y3.88964 Z6.66739

N46670 X3.97243 Y3.88964 Z6.65915

N46680 X4.00619 Y3.88964 Z6.65136

N46690 X4.03732 Y3.88964 Z6.64294

N46700 X4.08509 Y3.88964 Z6.63017

N46710 X4.1174 Y3.88964 Z6.6207

N46720 X4.16642 Y3.88964 Z6.60312

N46730 X4.24278 Y3.88964 Z6.5744

N46740 X4.25197 Y3.88964 Z6.57016

N46750 X4.27624 Y3.88964 Z6.55856

N46760 X4.3402 Y3.88964 Z6.52754

N46770 X4.36192 Y3.88964 Z6.51669

N46780 X4.42807 Y3.88964 Z6.47662

N46790 X4.47298 Y3.88964 Z6.44879

N46800 X4.51483 Y3.88964 Z6.41792

N46810 X4.5745 Y3.88964 Z6.37324

N46820 X4.66677 Y3.88964 Z6.29195

N46830 X4.68153 Y3.88964 Z6.27691

N46840 X4.75073 Y3.88964 Z6.20608

N46850 X4.76081 Y3.88964 Z6.19426

N46860 X4.82709 Y3.88964 Z6.11658

N46870 X4.83781 Y3.88964 Z6.10224

N46880 X4.89656 Y3.88964 Z6.0241

N46890 X4.91444 Y3.88964 Z5.99724

N46900 X4.96029 Y3.88964 Z5.92837

N46910 X4.99679 Y3.88964 Z5.86841

N46920 X5.01945 Y3.88964 Z5.83021

N46930 X5.04684 Y3.88964 Z5.78202

N46940 X5.07412 Y3.88964 Z5.73059

N46950 X5.12396 Y3.88964 Z5.6353

N46960 X5.12966 Y3.88964 Z5.62415

N46970 X5.18417 Y3.88964 Z5.5166

N46980 X5.19232 Y3.88964 Z5.50069

N46990 X5.24536 Y3.88964 Z5.39505

N47000 X5.27456 Y3.88964 Z5.33685

N47010 X5.30847 Y3.88964 Z5.26847

N47020 X5.35039 Y3.88964 Z5.18217

N47030 X5.37778 Y3.88964 Z5.12313

N47040 X5.45831 Y3.88964 Z4.94724

N47050 X5.54948 Y3.88964 Z4.72736

N47060 X5.59708 Y3.88964 Z4.60953

N47070 X5.62295 Y3.88964 Z4.54537

N47080 X5.65921 Y3.88964 Z4.46195

N47090 X5.70161 Y3.88964 Z4.36469

N47100 X5.83599 Y3.78585 Z4.27049

N47110 X5.81725 Y3.78585 Z4.31608

N47120 X5.7871 Y3.78585 Z4.38931

N47130 X5.77541 Y3.78585 Z4.41646

N47140 X5.76529 Y3.78585 Z4.4405

N47150 X5.72738 Y3.78585 Z4.53317

N47160 X5.68137 Y3.78585 Z4.65163

N47170 X5.66861 Y3.78585 Z4.68355

N47180 X5.6078 Y3.78585 Z4.83281

N47190 X5.51894 Y3.78585 Z5.03213

N47200 X5.43577 Y3.78585 Z5.20374

N47210 X5.41992 Y3.78585 Z5.23714

N47220 X5.36387 Y3.78585 Z5.34884

N47230 X5.34547 Y3.78585 Z5.38479

N47240 X5.29363 Y3.78585 Z5.48625

N47250 X5.28233 Y3.78585 Z5.50816

N47260 X5.23326 Y3.78585 Z5.60379

N47270 X5.22651 Y3.78585 Z5.61707

N47280 X5.18076 Y3.78585 Z5.70671

N47290 X5.17241 Y3.78585 Z5.72325

N47300 X5.13377 Y3.78585 Z5.79768

N47310 X5.11043 Y3.78585 Z5.84072

N47320 X5.088 Y3.78585 Z5.87834

N47330 X5.07014 Y3.78585 Z5.90874

N47340 X5.03255 Y3.78585 Z5.96816

N47350 X4.98818 Y3.78585 Z6.0336

N47360 X4.97025 Y3.78585 Z6.05959

N47370 X4.91247 Y3.78585 Z6.1341

N47380 X4.9028 Y3.78585 Z6.14678

N47390 X4.83693 Y3.78585 Z6.22252

N47400 X4.82893 Y3.78585 Z6.23188

N47410 X4.75767 Y3.78585 Z6.30477

N47420 X4.7473 Y3.78585 Z6.31547

N47430 X4.67395 Y3.78585 Z6.38123

N47440 X4.65757 Y3.78585 Z6.3959

N47450 X4.58578 Y3.78585 Z6.45121

N47460 X4.55931 Y3.78585 Z6.47135

N47470 X4.4937 Y3.78585 Z6.51368

N47480 X4.45211 Y3.78585 Z6.53989

N47490 X4.39948 Y3.78585 Z6.56748

N47500 X4.33762 Y3.78585 Z6.59894

N47510 X4.30591 Y3.78585 Z6.6121

N47520 X4.21593 Y3.78585 Z6.64794

N47530 X4.20208 Y3.78585 Z6.65232

N47540 X4.13166 Y3.78585 Z6.674

N47550 X4.10525 Y3.78585 Z6.68193

N47560 X4.05275 Y3.78585 Z6.69421

N47570 X3.99955 Y3.78585 Z6.70649

N47580 X3.98239 Y3.78585 Z6.70922

N47590 X3.92777 Y3.78585 Z6.71735

N47600 X3.88778 Y3.78585 Z6.72166

N47610 X3.84818 Y3.78585 Z6.72642

N47620 X3.76184 Y3.78585 Z6.73457

N47630 X3.69964 Y3.78585 Z6.73943

N47640 X3.66986 Y3.78585 Z6.74145

N47650 X3.6542 Y3.78585 Z6.7427

N47660 X3.59792 Y3.78585 Z6.74688

N47670 X3.5807 Y3.78585 Z6.74881

N47680 X3.54096 Y3.78585 Z6.75158

N47690 X3.49752 Y3.78585 Z6.75548

N47700 X3.46826 Y3.78585 Z6.75846

N47710 X3.44157 Y3.78585 Z6.76231

N47720 X3.39496 Y3.78585 Z6.76622

N47730 X3.33968 Y3.78585 Z6.7732

N47740 X3.32617 Y3.78585 Z6.77404

N47750 X3.26928 Y3.78585 Z6.77838

N47760 X3.2038 Y3.78585 Z6.78167

N47770 X3.17387 Y3.78585 Z6.78192

N47780 X3.06604 Y3.78585 Z6.77842

N47790 X3.00812 Y3.78585 Z6.77242

N47800 X2.94896 Y3.78585 Z6.76614

N47810 X2.83675 Y3.78585 Z6.74836

N47820 X2.80554 Y3.78585 Z6.743

N47830 X2.71629 Y3.78585 Z6.72533

N47840 X2.67528 Y3.78585 Z6.71651

N47850 X2.61448 Y3.78585 Z6.70202

N47860 X2.52946 Y3.78585 Z6.67936

N47870 X2.50574 Y3.78585 Z6.67267

N47880 X2.42576 Y3.78585 Z6.64777

N47890 X2.38615 Y3.78585 Z6.63555

N47900 X2.37592 Y3.78585 Z6.6322

N47910 X2.25879 Y3.78585 Z6.59117

N47920 X2.2295 Y3.78585 Z6.58053

N47930 X2.13142 Y3.78585 Z6.54236

N47940 X2.07776 Y3.78585 Z6.52091

N47950 X2.00693 Y3.78585 Z6.49122

N47960 X1.8992 Y3.78585 Z6.44509

N47970 X1.88741 Y3.78585 Z6.4399

N47980 X1.87425 Y3.78585 Z6.43395

N47990 X1.75298 Y3.78585 Z6.38086

N48000 X1.67596 Y3.78585 Z6.34641

N48010 X1.62381 Y3.78585 Z6.32397

N48020 X1.5452 Y3.78585 Z6.2896

N48030 X1.52741 Y3.78585 Z6.2821

N48040 X1.46345 Y3.78585 Z6.25381

N48050 X1.40928 Y3.78585 Z6.23126

N48060 X1.37547 Y3.78585 Z6.21622

N48070 X1.28544 Y3.78585 Z6.17918

N48080 X1.18004 Y3.78585 Z6.13547

N48090 X1.12164 Y3.78585 Z6.11302

N48100 X1.05839 Y3.78585 Z6.08626

N48110 X0.964802 Y3.78585 Z6.05017

N48120 X0.898022 Y3.78585 Z6.02224

N48130 X0.849069 Y3.78585 Z6.00285

N48140 X0.813801 Y3.78585 Z5.98853

N48150 X0.697492 Y3.78585 Z5.93693

N48160 X0.664751 Y3.78585 Z5.92245

N48170 X0.642024 Y3.78585 Z5.91114

N48180 X0.503648 Y3.78585 Z5.842

N48190 X0.330499 Y3.78585 Z5.74601

N48200 X0.300668 Y3.78585 Z5.72823

N48210 X0.165009 Y3.78585 Z5.64737

N48220 X0.0268614 Y3.78585 Z5.56127

N48230 X-0.119923 Y3.78585 Z5.46589

N48240 X-0.146579 Y3.78585 Z5.44824

N48250 X-0.16843 Y3.78585 Z5.43378

N48260 X-0.269495 Y3.78585 Z5.36863

N48270 X-0.345447 Y3.78585 Z5.31998

N48280 X-0.366668 Y3.78585 Z5.30711

N48290 X-0.406759 Y3.78585 Z5.28335

N48300 X-0.434575 Y3.78585 Z5.26699

N48310 X-0.445259 Y3.78585 Z5.26102

N48320 X-0.498263 Y3.78585 Z5.2338

N48330 X-0.515935 Y3.78585 Z5.22497

N48340 X-0.583871 Y3.78585 Z5.19397

N48350 X-0.597715 Y3.78585 Z5.18823

N48360 X-0.655745 Y3.78585 Z5.16437

N48370 X-0.684056 Y3.78585 Z5.15407

N48380 X-0.721989 Y3.78585 Z5.14034

N48390 X-0.770165 Y3.78585 Z5.12568

N48400 X-0.783037 Y3.78585 Z5.12176

N48410 X-0.7942 Y3.78585 Z5.11911

N48420 X-0.852419 Y3.78585 Z5.10548

N48430 X-0.900692 Y3.78585 Z5.09636

N48440 X-0.923611 Y3.78585 Z5.09241

N48450 X-0.982694 Y3.78585 Z5.08484

N48460 X-1.01349 Y3.78585 Z5.08277

N48470 X-1.03169 Y3.78585 Z5.08241

N48480 X-1.04727 Y3.78585 Z5.0827

N48490 X-1.09734 Y3.78585 Z5.08582

N48500 X-1.14205 Y3.78585 Z5.09125

N48510 X-1.16619 Y3.78585 Z5.09438

N48520 X-1.25042 Y3.78585 Z5.10933

N48530 X-1.27384 Y3.78585 Z5.11416

N48540 X-1.36467 Y3.78585 Z5.13329

N48550 X-1.38456 Y3.78585 Z5.13761

N48560 X-1.48225 Y3.78585 Z5.16174

N48570 X-1.51883 Y3.78585 Z5.17131

N48580 X-1.59862 Y3.78585 Z5.1936

N48590 X-1.63377 Y3.78585 Z5.20388

N48600 X-1.73474 Y3.78585 Z5.23623

N48610 X-1.75816 Y3.78585 Z5.24413

N48620 X-1.89912 Y3.78585 Z5.2953

N48630 X-1.99328 Y3.78585 Z5.33179

N48640 X-2.0442 Y3.78585 Z5.35154

N48650 X-2.11721 Y3.78585 Z5.38163

N48660 X-2.35086 Y3.78585 Z5.47932

N48670 X-2.54172 Y3.78585 Z5.55818

N48680 X-2.57294 Y3.78585 Z5.57125

N48690 X-2.59297 Y3.78585 Z5.57955

N48700 X-2.77178 Y3.78585 Z5.65386

N48710 X-2.87933 Y3.78585 Z5.69781

N48720 X-2.97599 Y3.78585 Z5.7353

N48730 X-3.04958 Y3.78585 Z5.76429

N48740 X-3.19045 Y3.78585 Z5.81664

N48750 X-3.32629 Y3.78585 Z5.86474

N48760 X-3.42093 Y3.78585 Z5.89599

N48770 X-3.46027 Y3.78585 Z5.90891

N48780 X-3.50277 Y3.78585 Z5.92168

N48790 X-3.58248 Y3.78585 Z5.94606

N48800 X-3.65414 Y3.78585 Z5.96544

N48810 X-3.70435 Y3.78585 Z5.97898

N48820 X-3.7668 Y3.78585 Z5.99445

N48830 X-3.82741 Y3.78585 Z6.00928

N48840 X-3.86743 Y3.78585 Z6.01812

N48850 X-3.94466 Y3.78585 Z6.03559

N48860 X-4.0451 Y3.78585 Z6.05596

N48870 X-4.0627 Y3.78585 Z6.05927

N48880 X-4.15565 Y3.78585 Z6.07457

N48890 X-4.22241 Y3.78585 Z6.08319

N48900 X-4.25781 Y3.78585 Z6.08759

N48910 X-4.31206 Y3.78585 Z6.0922

N48920 X-4.35791 Y3.78585 Z6.09594

N48930 X-4.38906 Y3.78585 Z6.09748

N48940 X-4.45703 Y3.78585 Z6.09959

N48950 X-4.55252 Y3.78585 Z6.09958

N48960 X-4.5634 Y3.78585 Z6.09945

N48970 X-4.66868 Y3.78585 Z6.09393

N48980 X-4.73491 Y3.78585 Z6.08901

N48990 X-4.79127 Y3.78585 Z6.0826

N49000 X-4.87464 Y3.78585 Z6.0714

N49010 X-4.89678 Y3.78585 Z6.06848

N49020 X-4.91176 Y3.78585 Z6.06598

N49030 X-5.00319 Y3.78585 Z6.04647

N49040 X-5.03407 Y3.78585 Z6.03866

N49050 X-5.11765 Y3.78585 Z6.01345

N49060 X-5.14016 Y3.78585 Z6.00668

N49070 X-5.15514 Y3.78585 Z6.00125

N49080 X-5.24725 Y3.78585 Z5.9662

N49090 X-5.31454 Y3.78585 Z5.93544

N49100 X-5.35785 Y3.78585 Z5.91558

N49110 X-5.44744 Y3.78585 Z5.8658

N49120 X-5.47526 Y3.78585 Z5.8495

N49130 X-5.57334 Y3.78585 Z5.78343

N49140 X-5.63283 Y3.78585 Z5.73819

N49150 X-5.67112 Y3.78585 Z5.70799

N49160 X-5.72618 Y3.78585 Z5.65854

N49170 X-5.76446 Y3.78585 Z5.6244

N49180 X-5.78394 Y3.78585 Z5.60496

N49190 X-5.85177 Y3.78585 Z5.53361

N49200 X-5.89807 Y3.78585 Z5.47909

N49210 X-5.93962 Y3.78585 Z5.42718

N49220 X-5.98628 Y3.78585 Z5.36184

N49230 X-6.01648 Y3.78585 Z5.31561

N49240 X-6.05048 Y3.78585 Z5.25807

N49250 X-6.07482 Y3.78585 Z5.21852

N49260 X-6.09381 Y3.78585 Z5.18445

N49270 X-6.12106 Y3.78585 Z5.13098

N49280 X-6.18407 Y3.78585 Z4.991

N49290 X-6.19774 Y3.78585 Z4.95883

N49300 X-6.20644 Y3.78585 Z4.93646

N49310 X-6.24773 Y3.78585 Z4.82321

N49320 X-6.27572 Y3.78585 Z4.73157

N49330 X-6.28439 Y3.78585 Z4.70142

N49340 X-6.30832 Y3.78585 Z4.62207

N49350 X-6.31637 Y3.78585 Z4.59441

N49360 X-6.33983 Y3.78585 Z4.52991

N49370 X-6.35073 Y3.78585 Z4.50315

N49380 X-6.40406 Y3.67933 Z4.49377

N49390 X-6.40005 Y3.67933 Z4.50312

N49400 X-6.37935 Y3.67933 Z4.56142

N49410 X-6.35263 Y3.67933 Z4.6546

N49420 X-6.32077 Y3.67933 Z4.76496

N49430 X-6.2827 Y3.67933 Z4.88157

N49440 X-6.26935 Y3.67933 Z4.91655

N49450 X-6.17514 Y3.67933 Z5.13067

N49460 X-6.1611 Y3.67933 Z5.15919

N49470 X-6.11748 Y3.67933 Z5.23701

N49480 X-6.11199 Y3.67933 Z5.24652

N49490 X-6.05789 Y3.67933 Z5.33264

N49500 X-6.03281 Y3.67933 Z5.36881

N49510 X-5.97834 Y3.67933 Z5.44498

N49520 X-5.95107 Y3.67933 Z5.48017

N49530 X-5.89543 Y3.67933 Z5.54543

N49540 X-5.87456 Y3.67933 Z5.56832

N49550 X-5.82039 Y3.67933 Z5.62234

N49560 X-5.7913 Y3.67933 Z5.65138

N49570 X-5.77334 Y3.67933 Z5.6674

N49580 X-5.70276 Y3.67933 Z5.72819

N49590 X-5.60727 Y3.67933 Z5.80101

N49600 X-5.59566 Y3.67933 Z5.80984

N49610 X-5.47713 Y3.67933 Z5.88749

N49620 X-5.44678 Y3.67933 Z5.90461

N49630 X-5.36977 Y3.67933 Z5.94708

N49640 X-5.31121 Y3.67933 Z5.97451

N49650 X-5.26523 Y3.67933 Z5.99588

N49660 X-5.17807 Y3.67933 Z6.03045

N49670 X-5.16401 Y3.67933 Z6.03543

N49680 X-5.08056 Y3.67933 Z6.06251

N49690 X-5.03745 Y3.67933 Z6.07378

N49700 X-4.98767 Y3.67933 Z6.08619

N49710 X-4.91176 Y3.67933 Z6.10045

N49720 X-4.89761 Y3.67933 Z6.10281

N49730 X-4.87184 Y3.67933 Z6.10622

N49740 X-4.80114 Y3.67933 Z6.1146

N49750 X-4.7696 Y3.67933 Z6.11761

N49760 X-4.66746 Y3.67933 Z6.12537

N49770 X-4.65602 Y3.67933 Z6.12622

N49780 X-4.55302 Y3.67933 Z6.12888

N49790 X-4.47803 Y3.67933 Z6.12906

N49800 X-4.45196 Y3.67933 Z6.12861

N49810 X-4.41641 Y3.67933 Z6.12701

N49820 X-4.34912 Y3.67933 Z6.12369

N49830 X-4.30504 Y3.67933 Z6.12009

N49840 X-4.24263 Y3.67933 Z6.11392

N49850 X-4.15372 Y3.67933 Z6.10293

N49860 X-4.13694 Y3.67933 Z6.10057

N49870 X-4.09023 Y3.67933 Z6.0929

N49880 X-4.04107 Y3.67933 Z6.08462

N49890 X-3.99209 Y3.67933 Z6.07448

N49900 X-3.94164 Y3.67933 Z6.06373

N49910 X-3.92302 Y3.67933 Z6.05935

N49920 X-3.81777 Y3.67933 Z6.03473

N49930 X-3.73229 Y3.67933 Z6.01384

N49940 X-3.69227 Y3.67933 Z6.00393

N49950 X-3.65325 Y3.67933 Z5.99341

N49960 X-3.56745 Y3.67933 Z5.96991

N49970 X-3.48314 Y3.67933 Z5.94481

N49980 X-3.46135 Y3.67933 Z5.93804

N49990 X-3.35931 Y3.67933 Z5.9047

N50000 X-3.24653 Y3.67933 Z5.86601

N50010 X-3.20606 Y3.67933 Z5.8519

N50020 X-3.16921 Y3.67933 Z5.83819

N50030 X-3.03358 Y3.67933 Z5.78953

N50040 X-2.91334 Y3.67933 Z5.74276

N50050 X-2.81577 Y3.67933 Z5.7074

N50060 X-2.63346 Y3.67933 Z5.63385

N50070 X-2.61862 Y3.67933 Z5.62829

N50080 X-2.57318 Y3.67933 Z5.6099

N50090 X-2.41329 Y3.67933 Z5.54517

N50100 X-2.27642 Y3.67933 Z5.48915

N50110 X-2.19038 Y3.67933 Z5.45458

N50120 X-2.12104 Y3.67933 Z5.42722

N50130 X-1.94501 Y3.67933 Z5.35848

N50140 X-1.76375 Y3.67933 Z5.2899

N50150 X-1.69077 Y3.67933 Z5.2635

N50160 X-1.55289 Y3.67933 Z5.21556

N50170 X-1.47851 Y3.67933 Z5.19214

N50180 X-1.39971 Y3.67933 Z5.16835

N50190 X-1.35657 Y3.67933 Z5.15676

N50200 X-1.28335 Y3.67933 Z5.13773

N50210 X-1.2655 Y3.67933 Z5.13352

N50220 X-1.21082 Y3.67933 Z5.12097

N50230 X-1.1872 Y3.67933 Z5.11563

N50240 X-1.12003 Y3.67933 Z5.1029

N50250 X-1.06311 Y3.67933 Z5.09518

N50260 X-1.00066 Y3.67933 Z5.09046

N50270 X-0.984724 Y3.67933 Z5.08995

N50280 X-0.949116 Y3.67933 Z5.08932

N50290 X-0.915855 Y3.67933 Z5.09057

N50300 X-0.88795 Y3.67933 Z5.09308

N50310 X-0.862087 Y3.67933 Z5.096

N50320 X-0.847002 Y3.67933 Z5.0986

N50330 X-0.797947 Y3.67933 Z5.10755

N50340 X-0.745316 Y3.67933 Z5.1202

N50350 X-0.725711 Y3.67933 Z5.12506

N50360 X-0.695059 Y3.67933 Z5.13442

N50370 X-0.653736 Y3.67933 Z5.14705

N50380 X-0.624188 Y3.67933 Z5.15775

N50390 X-0.579326 Y3.67933 Z5.17374

N50400 X-0.512575 Y3.67933 Z5.20076

N50410 X-0.445905 Y3.67933 Z5.23068

N50420 X-0.426405 Y3.67933 Z5.24071

N50430 X-0.381084 Y3.67933 Z5.26465

N50440 X-0.359338 Y3.67933 Z5.27754

N50450 X-0.263042 Y3.67933 Z5.33592

N50460 X-0.134417 Y3.67933 Z5.41778

N50470 X-0.0262895 Y3.67933 Z5.48748

N50480 X0.0207077 Y3.67933 Z5.51635

N50490 X0.192413 Y3.67933 Z5.62242

N50500 X0.292398 Y3.67933 Z5.68197

N50510 X0.370917 Y3.67933 Z5.72748

N50520 X0.473013 Y3.67933 Z5.78417

N50530 X0.555469 Y3.67933 Z5.82775

N50540 X0.637381 Y3.67933 Z5.86881

N50550 X0.732605 Y3.67933 Z5.91437

N50560 X0.792899 Y3.67933 Z5.94106

N50570 X0.941805 Y3.67933 Z6.00668

N50580 X0.952946 Y3.67933 Z6.01181

N50590 X1.09351 Y3.67933 Z6.07387

N50600 X1.1411 Y3.67933 Z6.09515

N50610 X1.17448 Y3.67933 Z6.11073

N50620 X1.22668 Y3.67933 Z6.13374

N50630 X1.28389 Y3.67933 Z6.16069

N50640 X1.31926 Y3.67933 Z6.17625

N50650 X1.383 Y3.67933 Z6.20583

N50660 X1.41831 Y3.67933 Z6.2222

N50670 X1.45768 Y3.67933 Z6.24123

N50680 X1.54291 Y3.67933 Z6.28037

N50690 X1.5984 Y3.67933 Z6.30555

N50700 X1.69992 Y3.67933 Z6.35427

N50710 X1.77349 Y3.67933 Z6.3877

N50720 X1.83332 Y3.67933 Z6.41561

N50730 X1.90543 Y3.67933 Z6.44759

N50740 X1.98589 Y3.67933 Z6.48342

N50750 X2.04539 Y3.67933 Z6.50856

N50760 X2.13801 Y3.67933 Z6.54735

N50770 X2.18117 Y3.67933 Z6.56427

N50780 X2.27891 Y3.67933 Z6.60188

N50790 X2.31122 Y3.67933 Z6.61328

N50800 X2.42337 Y3.67933 Z6.65145

N50810 X2.44685 Y3.67933 Z6.65876

N50820 X2.49 Y3.67933 Z6.67094

N50830 X2.56094 Y3.67933 Z6.69182

N50840 X2.60308 Y3.67933 Z6.70244

N50850 X2.64466 Y3.67933 Z6.7126

N50860 X2.7074 Y3.67933 Z6.72679

N50870 X2.7499 Y3.67933 Z6.73521

N50880 X2.81779 Y3.67933 Z6.7484

N50890 X2.90661 Y3.67933 Z6.76233

N50900 X2.92313 Y3.67933 Z6.76494

N50910 X2.98998 Y3.67933 Z6.77271

N50920 X3.03385 Y3.67933 Z6.7779

N50930 X3.04549 Y3.67933 Z6.77883

N50940 X3.14825 Y3.67933 Z6.78536

N50950 X3.20261 Y3.67933 Z6.78656

N50960 X3.26843 Y3.67933 Z6.78673

N50970 X3.30803 Y3.67933 Z6.7844

N50980 X3.40972 Y3.67933 Z6.78217

N50990 X3.42217 Y3.67933 Z6.78121

N51000 X3.4356 Y3.67933 Z6.78052

N51010 X3.53013 Y3.67933 Z6.77749

N51020 X3.59063 Y3.67933 Z6.7752

N51030 X3.63448 Y3.67933 Z6.77514

N51040 X3.68863 Y3.67933 Z6.7732

N51050 X3.76901 Y3.67933 Z6.77107

N51060 X3.79033 Y3.67933 Z6.77033

N51070 X3.8704 Y3.67933 Z6.76555

N51080 X3.89573 Y3.67933 Z6.76318

N51090 X3.96828 Y3.67933 Z6.7563

N51100 X3.9978 Y3.67933 Z6.75153

N51110 X4.07979 Y3.67933 Z6.7377

N51120 X4.11737 Y3.67933 Z6.72846

N51130 X4.17478 Y3.67933 Z6.71382

N51140 X4.19101 Y3.67933 Z6.70958

N51150 X4.26493 Y3.67933 Z6.68499

N51160 X4.30303 Y3.67933 Z6.67198

N51170 X4.36063 Y3.67933 Z6.64759

N51180 X4.41787 Y3.67933 Z6.62251

N51190 X4.46006 Y3.67933 Z6.60005

N51200 X4.52928 Y3.67933 Z6.56189

N51210 X4.55912 Y3.67933 Z6.54237

N51220 X4.6329 Y3.67933 Z6.49294

N51230 X4.65402 Y3.67933 Z6.47646

N51240 X4.72832 Y3.67933 Z6.41806

N51250 X4.74313 Y3.67933 Z6.40465

N51260 X4.81617 Y3.67933 Z6.3391

N51270 X4.82648 Y3.67933 Z6.32852

N51280 X4.89634 Y3.67933 Z6.25826

N51290 X4.90468 Y3.67933 Z6.24872

N51300 X4.96942 Y3.67933 Z6.17633

N51310 X4.9802 Y3.67933 Z6.16271

N51320 X5.03818 Y3.67933 Z6.08925

N51330 X5.06073 Y3.67933 Z6.0575

N51340 X5.10286 Y3.67933 Z5.99452

N51350 X5.15316 Y3.67933 Z5.91455

N51360 X5.16001 Y3.67933 Z5.90255

N51370 X5.17666 Y3.67933 Z5.8733

N51380 X5.21365 Y3.67933 Z5.80519

N51390 X5.22225 Y3.67933 Z5.78831

N51400 X5.26966 Y3.67933 Z5.69854

N51410 X5.27917 Y3.67933 Z5.67982

N51420 X5.3262 Y3.67933 Z5.59144

N51430 X5.33375 Y3.67933 Z5.57684

N51440 X5.38854 Y3.67933 Z5.47186

N51450 X5.39636 Y3.67933 Z5.45671

N51460 X5.4658 Y3.67933 Z5.32043

N51470 X5.4721 Y3.67933 Z5.30779

N51480 X5.51263 Y3.67933 Z5.22762

N51490 X5.55633 Y3.67933 Z5.1401

N51500 X5.56417 Y3.67933 Z5.12494

N51510 X5.6574 Y3.67933 Z4.93325

N51520 X5.73472 Y3.67933 Z4.75925

N51530 X5.79865 Y3.67933 Z4.5989

N51540 X5.80731 Y3.67933 Z4.57713

N51550 X5.82971 Y3.67933 Z4.521

N51560 X5.85679 Y3.67933 Z4.45131

N51570 X5.86665 Y3.67933 Z4.42659

N51580 X5.89822 Y3.67933 Z4.34191

N51590 X5.93249 Y3.67933 Z4.25892

N51600 X5.96334 Y3.67933 Z4.18014

N51610 X6.07886 Y3.57081 Z4.10119

N51620 X6.04042 Y3.57081 Z4.20409

N51630 X5.98217 Y3.57081 Z4.35196

N51640 X5.91707 Y3.57081 Z4.53141

N51650 X5.84892 Y3.57081 Z4.70188

N51660 X5.84259 Y3.57081 Z4.71634

N51670 X5.77707 Y3.57081 Z4.86336

N51680 X5.6719 Y3.57081 Z5.07214

N51690 X5.58779 Y3.57081 Z5.23535

N51700 X5.5023 Y3.57081 Z5.40358

N51710 X5.49054 Y3.57081 Z5.42667

N51720 X5.43657 Y3.57081 Z5.53137

N51730 X5.42467 Y3.57081 Z5.55387

N51740 X5.38584 Y3.57081 Z5.62362

N51750 X5.37177 Y3.57081 Z5.6504

N51760 X5.32441 Y3.57081 Z5.73639

N51770 X5.30565 Y3.57081 Z5.77186

N51780 X5.2497 Y3.57081 Z5.87284

N51790 X5.23597 Y3.57081 Z5.89697

N51800 X5.21121 Y3.57081 Z5.94033

N51810 X5.16967 Y3.57081 Z6.00914

N51820 X5.1364 Y3.57081 Z6.05921

N51830 X5.10213 Y3.57081 Z6.10813

N51840 X5.04676 Y3.57081 Z6.17906

N51850 X5.03172 Y3.57081 Z6.19763

N51860 X4.96704 Y3.57081 Z6.26941

N51870 X4.95713 Y3.57081 Z6.28042

N51880 X4.88811 Y3.57081 Z6.34851

N51890 X4.87628 Y3.57081 Z6.36044

N51900 X4.80583 Y3.57081 Z6.42277

N51910 X4.78872 Y3.57081 Z6.43824

N51920 X4.71793 Y3.57081 Z6.49413

N51930 X4.69525 Y3.57081 Z6.5121

N51940 X4.62265 Y3.57081 Z6.56215

N51950 X4.595 Y3.57081 Z6.58083

N51960 X4.52047 Y3.57081 Z6.62395

N51970 X4.48669 Y3.57081 Z6.64277

N51980 X4.4153 Y3.57081 Z6.67586

N51990 X4.37272 Y3.57081 Z6.6948

N52000 X4.3128 Y3.57081 Z6.71632

N52010 X4.26015 Y3.57081 Z6.73468

N52020 X4.21594 Y3.57081 Z6.74675

N52030 X4.1513 Y3.57081 Z6.76412

N52040 X4.12155 Y3.57081 Z6.76983

N52050 X4.03509 Y3.57081 Z6.78587

N52060 X4.02326 Y3.57081 Z6.78703

N52070 X3.93269 Y3.57081 Z6.79563

N52080 X3.91201 Y3.57081 Z6.79753

N52090 X3.89991 Y3.57081 Z6.79796

N52100 X3.79777 Y3.57081 Z6.80073

N52110 X3.76474 Y3.57081 Z6.80059

N52120 X3.68015 Y3.57081 Z6.8004

N52130 X3.61222 Y3.57081 Z6.80069

N52140 X3.53677 Y3.57081 Z6.79911

N52150 X3.38994 Y3.57081 Z6.79781

N52160 X3.24392 Y3.57081 Z6.7941

N52170 X3.19178 Y3.57081 Z6.7922

N52180 X3.10828 Y3.57081 Z6.78697

N52190 X3.03349 Y3.57081 Z6.78064

N52200 X2.98615 Y3.57081 Z6.77576

N52210 X2.91172 Y3.57081 Z6.76644

N52220 X2.88709 Y3.57081 Z6.76291

N52230 X2.82331 Y3.57081 Z6.75318

N52240 X2.7556 Y3.57081 Z6.74067

N52250 X2.71851 Y3.57081 Z6.73364

N52260 X2.61832 Y3.57081 Z6.71123

N52270 X2.59597 Y3.57081 Z6.70544

N52280 X2.52617 Y3.57081 Z6.68789

N52290 X2.48624 Y3.57081 Z6.676

N52300 X2.42197 Y3.57081 Z6.65736

N52310 X2.3257 Y3.57081 Z6.62422

N52320 X2.29894 Y3.57081 Z6.61508

N52330 X2.18639 Y3.57081 Z6.57185

N52340 X2.16822 Y3.57081 Z6.56484

N52350 X2.04549 Y3.57081 Z6.51359

N52360 X2.02369 Y3.57081 Z6.50442

N52370 X1.89899 Y3.57081 Z6.44867

N52380 X1.85925 Y3.57081 Z6.4309

N52390 X1.74588 Y3.57081 Z6.37677

N52400 X1.67605 Y3.57081 Z6.34422

N52410 X1.56248 Y3.57081 Z6.28675

N52420 X1.50859 Y3.57081 Z6.26085

N52430 X1.40321 Y3.57081 Z6.20573

N52440 X1.3219 Y3.57081 Z6.16575

N52450 X1.27933 Y3.57081 Z6.143

N52460 X1.2082 Y3.57081 Z6.10777

N52470 X1.17021 Y3.57081 Z6.08891

N52480 X1.15489 Y3.57081 Z6.08176

N52490 X1.06582 Y3.57081 Z6.03726

N52500 X1.03793 Y3.57081 Z6.02419

N52510 X0.951393 Y3.57081 Z5.98152

N52520 X0.926934 Y3.57081 Z5.9703

N52530 X0.829987 Y3.57081 Z5.92253

N52540 X0.798374 Y3.57081 Z5.90829

N52550 X0.718568 Y3.57081 Z5.86926

N52560 X0.658497 Y3.57081 Z5.84013

N52570 X0.601867 Y3.57081 Z5.81152

N52580 X0.436337 Y3.57081 Z5.72236

N52590 X0.358161 Y3.57081 Z5.67704

N52600 X0.244691 Y3.57081 Z5.61127

N52610 X0.0741633 Y3.57081 Z5.50803

N52620 X-0.104198 Y3.57081 Z5.39834

N52630 X-0.121317 Y3.57081 Z5.3873

N52640 X-0.141767 Y3.57081 Z5.37429

N52650 X-0.269246 Y3.57081 Z5.29775

N52660 X-0.323967 Y3.57081 Z5.26452

N52670 X-0.36531 Y3.57081 Z5.2428

N52680 X-0.392475 Y3.57081 Z5.22846

N52690 X-0.440588 Y3.57081 Z5.20707

N52700 X-0.451279 Y3.57081 Z5.20264

N52710 X-0.511069 Y3.57081 Z5.17922

N52720 X-0.56161 Y3.57081 Z5.1619

N52730 X-0.590006 Y3.57081 Z5.15185

N52740 X-0.625057 Y3.57081 Z5.14124

N52750 X-0.666225 Y3.57081 Z5.12902

N52760 X-0.699034 Y3.57081 Z5.12105

N52770 X-0.730838 Y3.57081 Z5.11328

N52780 X-0.76082 Y3.57081 Z5.10773

N52790 X-0.780027 Y3.57081 Z5.10431

N52800 X-0.792315 Y3.57081 Z5.10293

N52810 X-0.814417 Y3.57081 Z5.10045

N52820 X-0.840408 Y3.57081 Z5.09942

N52830 X-0.857152 Y3.57081 Z5.09931

N52840 X-0.898062 Y3.57081 Z5.10011

N52850 X-0.945911 Y3.57081 Z5.10358

N52860 X-0.957664 Y3.57081 Z5.10439

N52870 X-0.969951 Y3.57081 Z5.10578

N52880 X-1.01694 Y3.57081 Z5.11122

N52890 X-1.06981 Y3.57081 Z5.11993

N52900 X-1.08118 Y3.57081 Z5.12198

N52910 X-1.13741 Y3.57081 Z5.13395

N52920 X-1.18695 Y3.57081 Z5.14584

N52930 X-1.20789 Y3.57081 Z5.15119

N52940 X-1.25834 Y3.57081 Z5.16516

N52950 X-1.29027 Y3.57081 Z5.17405

N52960 X-1.31074 Y3.57081 Z5.18024

N52970 X-1.40002 Y3.57081 Z5.20856

N52980 X-1.52946 Y3.57081 Z5.25333

N52990 X-1.54647 Y3.57081 Z5.25924

N53000 X-1.77275 Y3.57081 Z5.34315

N53010 X-1.79596 Y3.57081 Z5.35192

N53020 X-2.02002 Y3.57081 Z5.4378

N53030 X-2.17535 Y3.57081 Z5.49861

N53040 X-2.25787 Y3.57081 Z5.53076

N53050 X-2.36358 Y3.57081 Z5.57242

N53060 X-2.46087 Y3.57081 Z5.6113

N53070 X-2.53154 Y3.57081 Z5.63918

N53080 X-2.61692 Y3.57081 Z5.67198

N53090 X-2.78162 Y3.57081 Z5.73339

N53100 X-2.79143 Y3.57081 Z5.73677

N53110 X-2.80185 Y3.57081 Z5.74027

N53120 X-3.00911 Y3.57081 Z5.81441

N53130 X-3.09581 Y3.57081 Z5.84344

N53140 X-3.21616 Y3.57081 Z5.88604

N53150 X-3.33543 Y3.57081 Z5.92642

N53160 X-3.39545 Y3.57081 Z5.94586

N53170 X-3.51811 Y3.57081 Z5.98393

N53180 X-3.54961 Y3.57081 Z5.99298

N53190 X-3.64912 Y3.57081 Z6.01979

N53200 X-3.77306 Y3.57081 Z6.05179

N53210 X-3.80436 Y3.57081 Z6.05944

N53220 X-3.82421 Y3.57081 Z6.06408

N53230 X-3.95239 Y3.57081 Z6.09428

N53240 X-3.97175 Y3.57081 Z6.0984

N53250 X-4.07736 Y3.57081 Z6.11895

N53260 X-4.09152 Y3.57081 Z6.12128

N53270 X-4.22214 Y3.57081 Z6.13946

N53280 X-4.24352 Y3.57081 Z6.14157

N53290 X-4.34142 Y3.57081 Z6.15071

N53300 X-4.40282 Y3.57081 Z6.15443

N53310 X-4.44681 Y3.57081 Z6.1564

N53320 X-4.50319 Y3.57081 Z6.15737

N53330 X-4.54817 Y3.57081 Z6.15774

N53340 X-4.58365 Y3.57081 Z6.15737

N53350 X-4.6476 Y3.57081 Z6.15542

N53360 X-4.74129 Y3.57081 Z6.15125

N53370 X-4.75242 Y3.57081 Z6.15019

N53380 X-4.85009 Y3.57081 Z6.14056

N53390 X-4.86457 Y3.57081 Z6.13824

N53400 X-4.94993 Y3.57081 Z6.1243

N53410 X-4.98015 Y3.57081 Z6.11879

N53420 X-5.06256 Y3.57081 Z6.09968

N53430 X-5.08413 Y3.57081 Z6.09407

N53440 X-5.15262 Y3.57081 Z6.07231

N53450 X-5.17948 Y3.57081 Z6.06379

N53460 X-5.18895 Y3.57081 Z6.06028

N53470 X-5.26938 Y3.57081 Z6.02865

N53480 X-5.31145 Y3.57081 Z6.00986

N53490 X-5.36845 Y3.57081 Z5.98317

N53500 X-5.41489 Y3.57081 Z5.95838

N53510 X-5.47479 Y3.57081 Z5.9251

N53520 X-5.53596 Y3.57081 Z5.88857

N53530 X-5.58524 Y3.57081 Z5.85632

N53540 X-5.64796 Y3.57081 Z5.81302

N53550 X-5.7221 Y3.57081 Z5.75705

N53560 X-5.74106 Y3.57081 Z5.74216

N53570 X-5.76288 Y3.57081 Z5.72356

N53580 X-5.82804 Y3.57081 Z5.66693

N53590 X-5.90798 Y3.57081 Z5.58974

N53600 X-5.97958 Y3.57081 Z5.50968

N53610 X-6.03411 Y3.57081 Z5.43973

N53620 X-6.05085 Y3.57081 Z5.41771

N53630 X-6.11939 Y3.57081 Z5.31892

N53640 X-6.16969 Y3.57081 Z5.23862

N53650 X-6.19404 Y3.57081 Z5.19573

N53660 X-6.21318 Y3.57081 Z5.16134

N53670 X-6.21956 Y3.57081 Z5.14889

N53680 X-6.26065 Y3.57081 Z5.06667

N53690 X-6.26649 Y3.57081 Z5.05353

N53700 X-6.30889 Y3.57081 Z4.95411

N53710 X-6.35202 Y3.57081 Z4.83224

N53720 X-6.381 Y3.57081 Z4.73358

N53730 X-6.38781 Y3.57081 Z4.70999

N53740 X-6.40991 Y3.57081 Z4.62507

N53750 X-6.42011 Y3.57081 Z4.58955

N53760 X-6.43672 Y3.57081 Z4.53013

N53770 X-6.443 Y3.57081 Z4.51244

N53780 X-6.45492 Y3.57081 Z4.48463

N53790 X-6.5066 Y3.4569 Z4.46642

N53800 X-6.49689 Y3.4569 Z4.48885

N53810 X-6.48794 Y3.4569 Z4.51602

N53820 X-6.47113 Y3.4569 Z4.57959

N53830 X-6.46361 Y3.4569 Z4.60859

N53840 X-6.45371 Y3.4569 Z4.64856

N53850 X-6.43394 Y3.4569 Z4.72089

N53860 X-6.41279 Y3.4569 Z4.79847

N53870 X-6.40541 Y3.4569 Z4.8232

N53880 X-6.37194 Y3.4569 Z4.9257

N53890 X-6.25796 Y3.4569 Z5.17631

N53900 X-6.21026 Y3.4569 Z5.25931

N53910 X-6.15689 Y3.4569 Z5.33935

N53920 X-6.13271 Y3.4569 Z5.37578

N53930 X-6.12254 Y3.4569 Z5.3904

N53940 X-6.09381 Y3.4569 Z5.42818

N53950 X-6.03416 Y3.4569 Z5.50605

N53960 X-5.98453 Y3.4569 Z5.56193

N53970 X-5.95251 Y3.4569 Z5.59758

N53980 X-5.92655 Y3.4569 Z5.62297

N53990 X-5.87126 Y3.4569 Z5.67607

N54000 X-5.81594 Y3.4569 Z5.72478

N54010 X-5.79025 Y3.4569 Z5.74668

N54020 X-5.75483 Y3.4569 Z5.7745

N54030 X-5.70461 Y3.4569 Z5.81347

N54040 X-5.67959 Y3.4569 Z5.83152

N54050 X-5.60961 Y3.4569 Z5.87963

N54060 X-5.57181 Y3.4569 Z5.90333

N54070 X-5.49464 Y3.4569 Z5.94956

N54080 X-5.47102 Y3.4569 Z5.96246

N54090 X-5.44535 Y3.4569 Z5.9763

N54100 X-5.38376 Y3.4569 Z6.00872

N54110 X-5.33614 Y3.4569 Z6.03197

N54120 X-5.31047 Y3.4569 Z6.04345

N54130 X-5.25635 Y3.4569 Z6.06544

N54140 X-5.20106 Y3.4569 Z6.08616

N54150 X-5.1697 Y3.4569 Z6.09725

N54160 X-5.08131 Y3.4569 Z6.12431

N54170 X-5.06801 Y3.4569 Z6.12778

N54180 X-4.98517 Y3.4569 Z6.14635

N54190 X-4.90169 Y3.4569 Z6.16192

N54200 X-4.81551 Y3.4569 Z6.17281

N54210 X-4.76215 Y3.4569 Z6.17751

N54220 X-4.73447 Y3.4569 Z6.17936

N54230 X-4.71374 Y3.4569 Z6.18052

N54240 X-4.64423 Y3.4569 Z6.1832

N54250 X-4.55471 Y3.4569 Z6.18544

N54260 X-4.53764 Y3.4569 Z6.18547

N54270 X-4.44277 Y3.4569 Z6.18316

N54280 X-4.34931 Y3.4569 Z6.17844

N54290 X-4.33509 Y3.4569 Z6.17752

N54300 X-4.31866 Y3.4569 Z6.17612

N54310 X-4.22851 Y3.4569 Z6.16692

N54320 X-4.1401 Y3.4569 Z6.15576

N54330 X-4.11642 Y3.4569 Z6.15245

N54340 X-4.02211 Y3.4569 Z6.13573

N54350 X-3.98909 Y3.4569 Z6.12932

N54360 X-3.9006 Y3.4569 Z6.10983

N54370 X-3.84033 Y3.4569 Z6.09564

N54380 X-3.75631 Y3.4569 Z6.07455

N54390 X-3.59375 Y3.4569 Z6.03311

N54400 X-3.57823 Y3.4569 Z6.0288

N54410 X-3.41336 Y3.4569 Z5.98115

N54420 X-3.26437 Y3.4569 Z5.93285

N54430 X-3.19683 Y3.4569 Z5.91156

N54440 X-2.97107 Y3.4569 Z5.83744

N54450 X-2.75646 Y3.4569 Z5.76551

N54460 X-2.66174 Y3.4569 Z5.73293

N54470 X-2.59938 Y3.4569 Z5.71217

N54480 X-2.52451 Y3.4569 Z5.68542

N54490 X-2.44356 Y3.4569 Z5.65505

N54500 X-2.33863 Y3.4569 Z5.61461

N54510 X-2.26842 Y3.4569 Z5.58769

N54520 X-2.2165 Y3.4569 Z5.56797

N54530 X-2.05284 Y3.4569 Z5.50437

N54540 X-1.85088 Y3.4569 Z5.42756

N54550 X-1.82332 Y3.4569 Z5.41701

N54560 X-1.75576 Y3.4569 Z5.39156

N54570 X-1.5953 Y3.4569 Z5.33084

N54580 X-1.54866 Y3.4569 Z5.31343

N54590 X-1.41785 Y3.4569 Z5.2651

N54600 X-1.36138 Y3.4569 Z5.24463

N54610 X-1.30756 Y3.4569 Z5.22574

N54620 X-1.20752 Y3.4569 Z5.19245

N54630 X-1.13206 Y3.4569 Z5.16956

N54640 X-1.09278 Y3.4569 Z5.15881

N54650 X-1.06571 Y3.4569 Z5.15178

N54660 X-1.02506 Y3.4569 Z5.1427

N54670 X-1.00313 Y3.4569 Z5.13739

N54680 X-0.987185 Y3.4569 Z5.13433

N54690 X-0.933876 Y3.4569 Z5.12445

N54700 X-0.88231 Y3.4569 Z5.11825

N54710 X-0.859855 Y3.4569 Z5.11504

N54720 X-0.798722 Y3.4569 Z5.11053

N54730 X-0.750153 Y3.4569 Z5.10951

N54740 X-0.720464 Y3.4569 Z5.11119

N54750 X-0.680301 Y3.4569 Z5.11714

N54760 X-0.668827 Y3.4569 Z5.1189

N54770 X-0.611659 Y3.4569 Z5.13131

N54780 X-0.567341 Y3.4569 Z5.14311

N54790 X-0.528238 Y3.4569 Z5.15415

N54800 X-0.491511 Y3.4569 Z5.16668

N54810 X-0.441057 Y3.4569 Z5.18289

N54820 X-0.399516 Y3.4569 Z5.19866

N54830 X-0.384286 Y3.4569 Z5.20464

N54840 X-0.355427 Y3.4569 Z5.2172

N54850 X-0.29155 Y3.4569 Z5.25037

N54860 X-0.275259 Y3.4569 Z5.25815

N54870 X-0.265119 Y3.4569 Z5.26325

N54880 X-0.13783 Y3.4569 Z5.33738

N54890 X-0.0757497 Y3.4569 Z5.37292

N54900 X0.179349 Y3.4569 Z5.5281

N54910 X0.296855 Y3.4569 Z5.59861

N54920 X0.34311 Y3.4569 Z5.6254

N54930 X0.453408 Y3.4569 Z5.69039

N54940 X0.534592 Y3.4569 Z5.73474

N54950 X0.590896 Y3.4569 Z5.76671

N54960 X0.694267 Y3.4569 Z5.82077

N54970 X0.718477 Y3.4569 Z5.83392

N54980 X0.79144 Y3.4569 Z5.8718

N54990 X0.857992 Y3.4569 Z5.90636

N55000 X0.887793 Y3.4569 Z5.92236

N55010 X0.9797 Y3.4569 Z5.97041

N55020 X1.00246 Y3.4569 Z5.98296

N55030 X1.09988 Y3.4569 Z6.03474

N55040 X1.13513 Y3.4569 Z6.05498

N55050 X1.20215 Y3.4569 Z6.09329

N55060 X1.24801 Y3.4569 Z6.11823

N55070 X1.3624 Y3.4569 Z6.18265

N55080 X1.49335 Y3.4569 Z6.25432

N55090 X1.527 Y3.4569 Z6.27137

N55100 X1.63021 Y3.4569 Z6.32687

N55110 X1.74673 Y3.4569 Z6.38621

N55120 X1.77216 Y3.4569 Z6.39839

N55130 X1.88101 Y3.4569 Z6.45121

N55140 X1.92293 Y3.4569 Z6.47103

N55150 X2.00268 Y3.4569 Z6.50674

N55160 X2.06488 Y3.4569 Z6.53412

N55170 X2.13275 Y3.4569 Z6.56208

N55180 X2.18991 Y3.4569 Z6.58539

N55190 X2.28621 Y3.4569 Z6.62132

N55200 X2.30655 Y3.4569 Z6.62886

N55210 X2.41951 Y3.4569 Z6.66667

N55220 X2.4489 Y3.4569 Z6.67514

N55230 X2.5289 Y3.4569 Z6.69844

N55240 X2.59632 Y3.4569 Z6.71504

N55250 X2.63806 Y3.4569 Z6.72524

N55260 X2.73343 Y3.4569 Z6.74496

N55270 X2.75493 Y3.4569 Z6.74919

N55280 X2.86846 Y3.4569 Z6.76805

N55290 X2.88357 Y3.4569 Z6.77033

N55300 X3.00923 Y3.4569 Z6.78624

N55310 X3.0269 Y3.4569 Z6.78823

N55320 X3.16288 Y3.4569 Z6.80064

N55330 X3.18792 Y3.4569 Z6.80269

N55340 X3.33089 Y3.4569 Z6.81148

N55350 X3.3691 Y3.4569 Z6.81363

N55360 X3.50674 Y3.4569 Z6.81904

N55370 X3.5767 Y3.4569 Z6.82156

N55380 X3.68533 Y3.4569 Z6.82411

N55390 X3.78768 Y3.4569 Z6.82554

N55400 X3.83921 Y3.4569 Z6.82648

N55410 X3.931 Y3.4569 Z6.82458

N55420 X3.97291 Y3.4569 Z6.82336

N55430 X4.04608 Y3.4569 Z6.81601

N55440 X4.09666 Y3.4569 Z6.81025

N55450 X4.15102 Y3.4569 Z6.79956

N55460 X4.20904 Y3.4569 Z6.7879

N55470 X4.25179 Y3.4569 Z6.776

N55480 X4.32026 Y3.4569 Z6.75661

N55490 X4.35655 Y3.4569 Z6.74341

N55500 X4.43531 Y3.4569 Z6.71358

N55510 X4.46682 Y3.4569 Z6.69887

N55520 X4.54771 Y3.4569 Z6.6592

N55530 X4.57727 Y3.4569 Z6.64204

N55540 X4.65215 Y3.4569 Z6.5969

N55550 X4.68105 Y3.4569 Z6.5769

N55560 X4.74941 Y3.4569 Z6.52922

N55570 X4.77457 Y3.4569 Z6.5092

N55580 X4.84318 Y3.4569 Z6.45592

N55590 X4.86041 Y3.4569 Z6.44054

N55600 X4.9341 Y3.4569 Z6.37659

N55610 X4.94382 Y3.4569 Z6.36696

N55620 X5.01833 Y3.4569 Z6.29323

N55630 X5.02764 Y3.4569 Z6.283

N55640 X5.09425 Y3.4569 Z6.20693

N55650 X5.11337 Y3.4569 Z6.1831

N55660 X5.16294 Y3.4569 Z6.11804

N55670 X5.20748 Y3.4569 Z6.05429

N55680 X5.22885 Y3.4569 Z6.02284

N55690 X5.24742 Y3.4569 Z5.99339

N55700 X5.29122 Y3.4569 Z5.91751

N55710 X5.33284 Y3.4569 Z5.84609

N55720 X5.3682 Y3.4569 Z5.78318

N55730 X5.41466 Y3.4569 Z5.69973

N55740 X5.44768 Y3.4569 Z5.63878

N55750 X5.51043 Y3.4569 Z5.52536

N55760 X5.52457 Y3.4569 Z5.4993

N55770 X5.60569 Y3.4569 Z5.34557

N55780 X5.62598 Y3.4569 Z5.30674

N55790 X5.63366 Y3.4569 Z5.29215

N55800 X5.7206 Y3.4569 Z5.12824

N55810 X5.75881 Y3.4569 Z5.05852

N55820 X5.86703 Y3.4569 Z4.84979

N55830 X5.89017 Y3.4569 Z4.80388

N55840 X5.95423 Y3.4569 Z4.65835

N55850 X5.96757 Y3.4569 Z4.62777

N55860 X6.0358 Y3.4569 Z4.4447

N55870 X6.06431 Y3.4569 Z4.36395

N55880 X6.15517 Y3.4569 Z4.12083

N55890 X6.17403 Y3.4569 Z4.06538

N55900 X6.1859 Y3.4569 Z4.03182

N55910 X6.27806 Y3.34245 Z3.98362

N55920 X6.26942 Y3.34245 Z4.00973

N55930 X6.22613 Y3.34245 Z4.1321

N55940 X6.17133 Y3.34245 Z4.29324

N55950 X6.15484 Y3.34245 Z4.3449

N55960 X6.14645 Y3.34245 Z4.36943

N55970 X6.13311 Y3.34245 Z4.40786

N55980 X6.0769 Y3.34245 Z4.56725

N55990 X6.05268 Y3.34245 Z4.62176

N56000 X5.99916 Y3.34245 Z4.74463

N56010 X5.9751 Y3.34245 Z4.79087

N56020 X5.86935 Y3.34245 Z4.99165

N56030 X5.79772 Y3.34245 Z5.12062

N56040 X5.75234 Y3.34245 Z5.20139

N56050 X5.71679 Y3.34245 Z5.266

N56060 X5.6272 Y3.34245 Z5.42962

N56070 X5.61692 Y3.34245 Z5.4491

N56080 X5.59516 Y3.34245 Z5.48918

N56090 X5.52108 Y3.34245 Z5.62249

N56100 X5.49751 Y3.34245 Z5.66422

N56110 X5.43309 Y3.34245 Z5.77944

N56120 X5.40657 Y3.34245 Z5.8241

N56130 X5.35529 Y3.34245 Z5.9124

N56140 X5.32986 Y3.34245 Z5.95212

N56150 X5.28174 Y3.34245 Z6.03049

N56160 X5.26732 Y3.34245 Z6.0514

N56170 X5.22197 Y3.34245 Z6.11441

N56180 X5.17986 Y3.34245 Z6.17131

N56190 X5.15436 Y3.34245 Z6.20374

N56200 X5.08815 Y3.34245 Z6.28323

N56210 X5.07945 Y3.34245 Z6.29303

N56220 X4.99268 Y3.34245 Z6.38194

N56230 X4.90908 Y3.34245 Z6.45458

N56240 X4.89535 Y3.34245 Z6.46664

N56250 X4.82352 Y3.34245 Z6.5205

N56260 X4.79426 Y3.34245 Z6.54355

N56270 X4.7342 Y3.34245 Z6.58474

N56280 X4.69792 Y3.34245 Z6.61009

N56290 X4.63036 Y3.34245 Z6.65203

N56300 X4.59793 Y3.34245 Z6.6715

N56310 X4.51549 Y3.34245 Z6.71419

N56320 X4.48963 Y3.34245 Z6.72686

N56330 X4.39745 Y3.34245 Z6.76392

N56340 X4.3741 Y3.34245 Z6.7728

N56350 X4.28442 Y3.34245 Z6.79934

N56360 X4.25858 Y3.34245 Z6.80673

N56370 X4.17786 Y3.34245 Z6.82352

N56380 X4.14618 Y3.34245 Z6.82998

N56390 X4.07444 Y3.34245 Z6.83923

N56400 X4.03168 Y3.34245 Z6.84417

N56410 X3.96882 Y3.34245 Z6.84773

N56420 X3.90917 Y3.34245 Z6.84951

N56430 X3.85343 Y3.34245 Z6.84975

N56440 X3.73601 Y3.34245 Z6.84764

N56450 X3.69315 Y3.34245 Z6.84677

N56460 X3.65512 Y3.34245 Z6.84566

N56470 X3.48757 Y3.34245 Z6.83961

N56480 X3.43118 Y3.34245 Z6.83718

N56490 X3.29787 Y3.34245 Z6.83006

N56500 X3.20076 Y3.34245 Z6.82291

N56510 X3.13988 Y3.34245 Z6.81818

N56520 X3.00598 Y3.34245 Z6.80493

N56530 X2.99523 Y3.34245 Z6.80375

N56540 X2.88872 Y3.34245 Z6.78951

N56550 X2.84705 Y3.34245 Z6.78398

N56560 X2.69947 Y3.34245 Z6.75729

N56570 X2.56996 Y3.34245 Z6.72783

N56580 X2.53969 Y3.34245 Z6.71943

N56590 X2.4472 Y3.34245 Z6.69403

N56600 X2.36903 Y3.34245 Z6.66901

N56610 X2.33208 Y3.34245 Z6.65726

N56620 X2.26412 Y3.34245 Z6.63269

N56630 X2.219 Y3.34245 Z6.61679

N56640 X2.19962 Y3.34245 Z6.60908

N56650 X2.10916 Y3.34245 Z6.57342

N56660 X2.05995 Y3.34245 Z6.55199

N56670 X2.01258 Y3.34245 Z6.53134

N56680 X1.9697 Y3.34245 Z6.51161

N56690 X1.87676 Y3.34245 Z6.46727

N56700 X1.86282 Y3.34245 Z6.46018

N56710 X1.77807 Y3.34245 Z6.41762

N56720 X1.70111 Y3.34245 Z6.3773

N56730 X1.67679 Y3.34245 Z6.36429

N56740 X1.65415 Y3.34245 Z6.35145

N56750 X1.57435 Y3.34245 Z6.30811

N56760 X1.47399 Y3.34245 Z6.25029

N56770 X1.46451 Y3.34245 Z6.2451

N56780 X1.44439 Y3.34245 Z6.23341

N56790 X1.35935 Y3.34245 Z6.18363

N56800 X1.32482 Y3.34245 Z6.16209

N56810 X1.25775 Y3.34245 Z6.12259

N56820 X1.16338 Y3.34245 Z6.0649

N56830 X1.09409 Y3.34245 Z6.02195

N56840 X1.06873 Y3.34245 Z6.00679

N56850 X0.986098 Y3.34245 Z5.95589

N56860 X0.945077 Y3.34245 Z5.93226

N56870 X0.854591 Y3.34245 Z5.87938

N56880 X0.830171 Y3.34245 Z5.8659

N56890 X0.71812 Y3.34245 Z5.80274

N56900 X0.603311 Y3.34245 Z5.73778

N56910 X0.542277 Y3.34245 Z5.70139

N56920 X0.46805 Y3.34245 Z5.6592

N56930 X0.385205 Y3.34245 Z5.60913

N56940 X0.354688 Y3.34245 Z5.59119

N56950 X0.27789 Y3.34245 Z5.54475

N56960 X0.163449 Y3.34245 Z5.47534

N56970 X0.127362 Y3.34245 Z5.45324

N56980 X-0.047029 Y3.34245 Z5.34795

N56990 X-0.0955512 Y3.34245 Z5.32078

N57000 X-0.161063 Y3.34245 Z5.28327

N57010 X-0.191912 Y3.34245 Z5.26531

N57020 X-0.270548 Y3.34245 Z5.22569

N57030 X-0.30171 Y3.34245 Z5.21082

N57040 X-0.337607 Y3.34245 Z5.19614

N57050 X-0.368126 Y3.34245 Z5.18425

N57060 X-0.405992 Y3.34245 Z5.17183

N57070 X-0.446055 Y3.34245 Z5.15863

N57080 X-0.494234 Y3.34245 Z5.14597

N57090 X-0.537537 Y3.34245 Z5.13533

N57100 X-0.569227 Y3.34245 Z5.12923

N57110 X-0.608668 Y3.34245 Z5.12261

N57120 X-0.626344 Y3.34245 Z5.12068

N57130 X-0.661763 Y3.34245 Z5.11764

N57140 X-0.674247 Y3.34245 Z5.11743

N57150 X-0.710315 Y3.34245 Z5.11731

N57160 X-0.726158 Y3.34245 Z5.11852

N57170 X-0.766435 Y3.34245 Z5.12141

N57180 X-0.793081 Y3.34245 Z5.12523

N57190 X-0.835829 Y3.34245 Z5.13089

N57200 X-0.871518 Y3.34245 Z5.13798

N57210 X-0.920933 Y3.34245 Z5.14886

N57220 X-0.939082 Y3.34245 Z5.15334

N57230 X-0.975348 Y3.34245 Z5.16372

N57240 X-1.00011 Y3.34245 Z5.17001

N57250 X-1.01422 Y3.34245 Z5.17419

N57260 X-1.06859 Y3.34245 Z5.19072

N57270 X-1.13822 Y3.34245 Z5.21461

N57280 X-1.15172 Y3.34245 Z5.21928

N57290 X-1.1785 Y3.34245 Z5.22941

N57300 X-1.27593 Y3.34245 Z5.26394

N57310 X-1.34032 Y3.34245 Z5.28775

N57320 X-1.4427 Y3.34245 Z5.32667

N57330 X-1.59362 Y3.34245 Z5.38357

N57340 X-1.63237 Y3.34245 Z5.39793

N57350 X-1.70828 Y3.34245 Z5.42682

N57360 X-1.84646 Y3.34245 Z5.47854

N57370 X-1.92554 Y3.34245 Z5.50885

N57380 X-2.05763 Y3.34245 Z5.55787

N57390 X-2.22782 Y3.34245 Z5.62322

N57400 X-2.24372 Y3.34245 Z5.62898

N57410 X-2.27226 Y3.34245 Z5.63965

N57420 X-2.38768 Y3.34245 Z5.68206

N57430 X-2.49314 Y3.34245 Z5.71895

N57440 X-2.52298 Y3.34245 Z5.72892

N57450 X-2.53717 Y3.34245 Z5.73344

N57460 X-2.67643 Y3.34245 Z5.77812

N57470 X-2.73071 Y3.34245 Z5.79481

N57480 X-2.82676 Y3.34245 Z5.82505

N57490 X-2.90005 Y3.34245 Z5.84846

N57500 X-3.0864 Y3.34245 Z5.90635

N57510 X-3.12327 Y3.34245 Z5.91845

N57520 X-3.22555 Y3.34245 Z5.9507

N57530 X-3.34744 Y3.34245 Z5.9881

N57540 X-3.40471 Y3.34245 Z6.00492

N57550 X-3.53189 Y3.34245 Z6.0413

N57560 X-3.68096 Y3.34245 Z6.08176

N57570 X-3.69842 Y3.34245 Z6.08631

N57580 X-3.70833 Y3.34245 Z6.08886

N57590 X-3.84004 Y3.34245 Z6.12058

N57600 X-3.90344 Y3.34245 Z6.13522

N57610 X-3.96132 Y3.34245 Z6.14768

N57620 X-4.04405 Y3.34245 Z6.16412

N57630 X-4.06646 Y3.34245 Z6.16805

N57640 X-4.15763 Y3.34245 Z6.18275

N57650 X-4.23257 Y3.34245 Z6.191

N57660 X-4.33419 Y3.34245 Z6.20099

N57670 X-4.3447 Y3.34245 Z6.20167

N57680 X-4.43959 Y3.34245 Z6.2065

N57690 X-4.52021 Y3.34245 Z6.20851

N57700 X-4.54555 Y3.34245 Z6.20879

N57710 X-4.57232 Y3.34245 Z6.2085

N57720 X-4.64722 Y3.34245 Z6.20651

N57730 X-4.71697 Y3.34245 Z6.20302

N57740 X-4.74394 Y3.34245 Z6.20144

N57750 X-4.77039 Y3.34245 Z6.19931

N57760 X-4.8367 Y3.34245 Z6.1926

N57770 X-4.92706 Y3.34245 Z6.18068

N57780 X-5.01395 Y3.34245 Z6.16387

N57790 X-5.05613 Y3.34245 Z6.15465

N57800 X-5.09791 Y3.34245 Z6.14397

N57810 X-5.14715 Y3.34245 Z6.12899

N57820 X-5.20082 Y3.34245 Z6.11139

N57830 X-5.23468 Y3.34245 Z6.09916

N57840 X-5.32339 Y3.34245 Z6.0644

N57850 X-5.39582 Y3.34245 Z6.03085

N57860 X-5.45872 Y3.34245 Z6.00035

N57870 X-5.4748 Y3.34245 Z5.99169

N57880 X-5.51202 Y3.34245 Z5.97135

N57890 X-5.55843 Y3.34245 Z5.94559

N57900 X-5.57541 Y3.34245 Z5.93604

N57910 X-5.65188 Y3.34245 Z5.88803

N57920 X-5.67269 Y3.34245 Z5.87437

N57930 X-5.76012 Y3.34245 Z5.81172

N57940 X-5.83763 Y3.34245 Z5.74877

N57950 X-5.89966 Y3.34245 Z5.69514

N57960 X-5.91704 Y3.34245 Z5.67953

N57970 X-5.9386 Y3.34245 Z5.65901

N57980 X-5.99725 Y3.34245 Z5.60154

N57990 X-6.05333 Y3.34245 Z5.53966

N58000 X-6.07961 Y3.34245 Z5.51067

N58010 X-6.10177 Y3.34245 Z5.48358

N58020 X-6.155 Y3.34245 Z5.41472

N58030 X-6.20627 Y3.34245 Z5.3439

N58040 X-6.23378 Y3.34245 Z5.30257

N58050 X-6.27607 Y3.34245 Z5.23565

N58060 X-6.30446 Y3.34245 Z5.18552

N58070 X-6.33551 Y3.34245 Z5.1275

N58080 X-6.42263 Y3.34245 Z4.916

N58090 X-6.45062 Y3.34245 Z4.8247

N58100 X-6.47863 Y3.34245 Z4.72416

N58110 X-6.49572 Y3.34245 Z4.66104

N58120 X-6.50679 Y3.34245 Z4.61638

N58130 X-6.52728 Y3.34245 Z4.53738

N58140 X-6.53503 Y3.34245 Z4.50698

N58150 X-6.5572 Y3.34245 Z4.44148

N58160 X-6.60292 Y3.22703 Z4.41115

N58170 X-6.59662 Y3.22703 Z4.43404

N58180 X-6.57134 Y3.22703 Z4.51744

N58190 X-6.56361 Y3.22703 Z4.55012

N58200 X-6.54289 Y3.22703 Z4.63582

N58210 X-6.51982 Y3.22703 Z4.726

N58220 X-6.51535 Y3.22703 Z4.74177

N58230 X-6.51086 Y3.22703 Z4.75753

N58240 X-6.48599 Y3.22703 Z4.84127

N58250 X-6.45911 Y3.22703 Z4.9296

N58260 X-6.40144 Y3.22703 Z5.08347

N58270 X-6.39435 Y3.22703 Z5.09986

N58280 X-6.35523 Y3.22703 Z5.17853

N58290 X-6.34815 Y3.22703 Z5.19173

N58300 X-6.30029 Y3.22703 Z5.27358

N58310 X-6.29141 Y3.22703 Z5.28761

N58320 X-6.24016 Y3.22703 Z5.36363

N58330 X-6.22091 Y3.22703 Z5.3904

N58340 X-6.17865 Y3.22703 Z5.44546

N58350 X-6.1226 Y3.22703 Z5.51467

N58360 X-6.11419 Y3.22703 Z5.52453

N58370 X-6.10448 Y3.22703 Z5.53509

N58380 X-6.04128 Y3.22703 Z5.60257

N58390 X-5.98012 Y3.22703 Z5.66139

N58400 X-5.95815 Y3.22703 Z5.68228

N58410 X-5.935 Y3.22703 Z5.70308

N58420 X-5.87566 Y3.22703 Z5.75478

N58430 X-5.8215 Y3.22703 Z5.79927

N58440 X-5.80073 Y3.22703 Z5.8158

N58450 X-5.76263 Y3.22703 Z5.84444

N58460 X-5.72554 Y3.22703 Z5.87154

N58470 X-5.71177 Y3.22703 Z5.88115

N58480 X-5.64337 Y3.22703 Z5.92592

N58490 X-5.62396 Y3.22703 Z5.93728

N58500 X-5.5419 Y3.22703 Z5.98411

N58510 X-5.45738 Y3.22703 Z6.02589

N58520 X-5.42189 Y3.22703 Z6.04322

N58530 X-5.37269 Y3.22703 Z6.06449

N58540 X-5.29199 Y3.22703 Z6.09816

N58550 X-5.26221 Y3.22703 Z6.10869

N58560 X-5.1888 Y3.22703 Z6.13425

N58570 X-5.16932 Y3.22703 Z6.14071

N58580 X-5.08169 Y3.22703 Z6.16521

N58590 X-5.05837 Y3.22703 Z6.17114

N58600 X-4.9801 Y3.22703 Z6.18758

N58610 X-4.95695 Y3.22703 Z6.19239

N58620 X-4.85805 Y3.22703 Z6.20708

N58630 X-4.77321 Y3.22703 Z6.21665

N58640 X-4.7566 Y3.22703 Z6.21824

N58650 X-4.73796 Y3.22703 Z6.21947

N58660 X-4.65203 Y3.22703 Z6.22465

N58670 X-4.56307 Y3.22703 Z6.22707

N58680 X-4.5447 Y3.22703 Z6.22737

N58690 X-4.52722 Y3.22703 Z6.22713

N58700 X-4.43521 Y3.22703 Z6.22487

N58710 X-4.33091 Y3.22703 Z6.21911

N58720 X-4.22441 Y3.22703 Z6.20862

N58730 X-4.14904 Y3.22703 Z6.19944

N58740 X-4.12491 Y3.22703 Z6.19624

N58750 X-4.08033 Y3.22703 Z6.18918

N58760 X-4.0242 Y3.22703 Z6.1798

N58770 X-3.99937 Y3.22703 Z6.17509

N58780 X-3.9156 Y3.22703 Z6.15822

N58790 X-3.82419 Y3.22703 Z6.13819

N58800 X-3.7893 Y3.22703 Z6.13

N58810 X-3.72381 Y3.22703 Z6.11401

N58820 X-3.64184 Y3.22703 Z6.09293

N58830 X-3.58459 Y3.22703 Z6.078

N58840 X-3.47295 Y3.22703 Z6.04712

N58850 X-3.29297 Y3.22703 Z5.99634

N58860 X-3.27753 Y3.22703 Z5.99185

N58870 X-3.10238 Y3.22703 Z5.93714

N58880 X-2.9798 Y3.22703 Z5.90172

N58890 X-2.93613 Y3.22703 Z5.88826

N58900 X-2.82598 Y3.22703 Z5.85508

N58910 X-2.74871 Y3.22703 Z5.83227

N58920 X-2.68752 Y3.22703 Z5.81447

N58930 X-2.58595 Y3.22703 Z5.78359

N58940 X-2.54714 Y3.22703 Z5.77206

N58950 X-2.42351 Y3.22703 Z5.73378

N58960 X-2.40306 Y3.22703 Z5.72726

N58970 X-2.23349 Y3.22703 Z5.66973

N58980 X-2.20068 Y3.22703 Z5.65776

N58990 X-2.05299 Y3.22703 Z5.60314

N59000 X-1.98753 Y3.22703 Z5.58017

N59010 X-1.85901 Y3.22703 Z5.53158

N59020 X-1.67668 Y3.22703 Z5.46455

N59030 X-1.64656 Y3.22703 Z5.45308

N59040 X-1.61147 Y3.22703 Z5.44009

N59050 X-1.46881 Y3.22703 Z5.38629

N59060 X-1.36506 Y3.22703 Z5.3459

N59070 X-1.32326 Y3.22703 Z5.32983

N59080 X-1.21574 Y3.22703 Z5.28618

N59090 X-1.14472 Y3.22703 Z5.25941

N59100 X-1.10761 Y3.22703 Z5.24423

N59110 X-1.08222 Y3.22703 Z5.2346

N59120 X-1.02274 Y3.22703 Z5.21208

N59130 X-0.956183 Y3.22703 Z5.18944

N59140 X-0.892299 Y3.22703 Z5.16841

N59150 X-0.865624 Y3.22703 Z5.16062

N59160 X-0.833397 Y3.22703 Z5.15177

N59170 X-0.793451 Y3.22703 Z5.1418

N59180 X-0.774555 Y3.22703 Z5.13793

N59190 X-0.736089 Y3.22703 Z5.12995

N59200 X-0.714724 Y3.22703 Z5.12708

N59210 X-0.683847 Y3.22703 Z5.12236

N59220 X-0.654705 Y3.22703 Z5.12049

N59230 X-0.629827 Y3.22703 Z5.1183

N59240 X-0.593084 Y3.22703 Z5.11891

N59250 X-0.565981 Y3.22703 Z5.11988

N59260 X-0.532641 Y3.22703 Z5.12311

N59270 X-0.501689 Y3.22703 Z5.12672

N59280 X-0.470203 Y3.22703 Z5.13243

N59290 X-0.432106 Y3.22703 Z5.13993

N59300 X-0.400726 Y3.22703 Z5.1481

N59310 X-0.361214 Y3.22703 Z5.15877

N59320 X-0.332096 Y3.22703 Z5.16843

N59330 X-0.301734 Y3.22703 Z5.17845

N59340 X-0.270484 Y3.22703 Z5.19114

N59350 X-0.241616 Y3.22703 Z5.20272

N59360 X-0.188122 Y3.22703 Z5.22943

N59370 X-0.15702 Y3.22703 Z5.2439

N59380 X-0.102773 Y3.22703 Z5.27268

N59390 X-0.0876567 Y3.22703 Z5.28114

N59400 X-0.0084506 Y3.22703 Z5.32747

N59410 X0.190733 Y3.22703 Z5.4496

N59420 X0.24102 Y3.22703 Z5.48149

N59430 X0.340261 Y3.22703 Z5.54317

N59440 X0.35715 Y3.22703 Z5.55361

N59450 X0.366681 Y3.22703 Z5.55936

N59460 X0.482912 Y3.22703 Z5.63232

N59470 X0.510649 Y3.22703 Z5.64892

N59480 X0.615485 Y3.22703 Z5.71411

N59490 X0.630823 Y3.22703 Z5.72315

N59500 X0.656086 Y3.22703 Z5.73839

N59510 X0.735987 Y3.22703 Z5.78746

N59520 X0.820082 Y3.22703 Z5.84043

N59530 X0.83132 Y3.22703 Z5.84732

N59540 X0.840439 Y3.22703 Z5.85316

N59550 X0.913592 Y3.22703 Z5.90042

N59560 X0.952343 Y3.22703 Z5.92628

N59570 X1.01116 Y3.22703 Z5.96419

N59580 X1.0523 Y3.22703 Z5.99174

N59590 X1.13353 Y3.22703 Z6.0445

N59600 X1.14797 Y3.22703 Z6.05431

N59610 X1.18099 Y3.22703 Z6.07628

N59620 X1.23929 Y3.22703 Z6.1146

N59630 X1.26962 Y3.22703 Z6.13369

N59640 X1.34171 Y3.22703 Z6.18218

N59650 X1.44307 Y3.22703 Z6.2452

N59660 X1.45484 Y3.22703 Z6.25255

N59670 X1.55491 Y3.22703 Z6.31366

N59680 X1.60367 Y3.22703 Z6.34138

N59690 X1.65369 Y3.22703 Z6.37091

N59700 X1.72209 Y3.22703 Z6.40879

N59710 X1.73228 Y3.22703 Z6.41453

N59720 X1.80442 Y3.22703 Z6.45358

N59730 X1.86013 Y3.22703 Z6.483

N59740 X1.90221 Y3.22703 Z6.50445

N59750 X1.96225 Y3.22703 Z6.5334

N59760 X2.00226 Y3.22703 Z6.55333

N59770 X2.03446 Y3.22703 Z6.56805

N59780 X2.11256 Y3.22703 Z6.6032

N59790 X2.17135 Y3.22703 Z6.62626

N59800 X2.23481 Y3.22703 Z6.65198

N59810 X2.31608 Y3.22703 Z6.68049

N59820 X2.34881 Y3.22703 Z6.6922

N59830 X2.42063 Y3.22703 Z6.71444

N59840 X2.46249 Y3.22703 Z6.72772

N59850 X2.49867 Y3.22703 Z6.73734

N59860 X2.58804 Y3.22703 Z6.76201

N59870 X2.63167 Y3.22703 Z6.77199

N59880 X2.73389 Y3.22703 Z6.79385

N59890 X2.77696 Y3.22703 Z6.80177

N59900 X2.88511 Y3.22703 Z6.81887

N59910 X2.92226 Y3.22703 Z6.82371

N59920 X3.03203 Y3.22703 Z6.83579

N59930 X3.0547 Y3.22703 Z6.83798

N59940 X3.10526 Y3.22703 Z6.84165

N59950 X3.18366 Y3.22703 Z6.84828

N59960 X3.26815 Y3.22703 Z6.85325

N59970 X3.34121 Y3.22703 Z6.85752

N59980 X3.37006 Y3.22703 Z6.85831

N59990 X3.54215 Y3.22703 Z6.8653

N60000 X3.55457 Y3.22703 Z6.86554

N60010 X3.5976 Y3.22703 Z6.86667

N60020 X3.70031 Y3.22703 Z6.86967

N60030 X3.74616 Y3.22703 Z6.8706

N60040 X3.81204 Y3.22703 Z6.87139

N60050 X3.85131 Y3.22703 Z6.87083

N60060 X3.91336 Y3.22703 Z6.87038

N60070 X3.97016 Y3.22703 Z6.86821

N60080 X4.00848 Y3.22703 Z6.86597

N60090 X4.08212 Y3.22703 Z6.86029

N60100 X4.10156 Y3.22703 Z6.85775

N60110 X4.19429 Y3.22703 Z6.84456

N60120 X4.30792 Y3.22703 Z6.81963

N60130 X4.42413 Y3.22703 Z6.78311

N60140 X4.53386 Y3.22703 Z6.73624

N60150 X4.55911 Y3.22703 Z6.72329

N60160 X4.63482 Y3.22703 Z6.68235

N60170 X4.67753 Y3.22703 Z6.65622

N60180 X4.73228 Y3.22703 Z6.62221

N60190 X4.78144 Y3.22703 Z6.58814

N60200 X4.843 Y3.22703 Z6.54844

N60210 X4.86838 Y3.22703 Z6.52895

N60220 X4.9541 Y3.22703 Z6.46481

N60230 X4.9649 Y3.22703 Z6.45513

N60240 X5.04625 Y3.22703 Z6.38183

N60250 X5.08976 Y3.22703 Z6.33595

N60260 X5.13577 Y3.22703 Z6.28621

N60270 X5.14389 Y3.22703 Z6.2766

N60280 X5.20903 Y3.22703 Z6.19543

N60290 X5.25058 Y3.22703 Z6.14084

N60300 X5.28227 Y3.22703 Z6.09782

N60310 X5.35317 Y3.22703 Z5.99641

N60320 X5.36133 Y3.22703 Z5.98481

N60330 X5.43918 Y3.22703 Z5.86307

N60340 X5.44691 Y3.22703 Z5.85112

N60350 X5.5227 Y3.22703 Z5.72352

N60360 X5.52938 Y3.22703 Z5.71257

N60370 X5.56985 Y3.22703 Z5.64182

N60380 X5.61125 Y3.22703 Z5.56854

N60390 X5.62183 Y3.22703 Z5.54949

N60400 X5.70777 Y3.22703 Z5.39592

N60410 X5.75375 Y3.22703 Z5.31347

N60420 X5.82453 Y3.22703 Z5.19211

N60430 X5.85778 Y3.22703 Z5.1335

N60440 X5.92325 Y3.22703 Z5.02024

N60450 X6.00817 Y3.22703 Z4.86716

N60460 X6.03295 Y3.22703 Z4.82255

N60470 X6.0824 Y3.22703 Z4.72704

N60480 X6.1168 Y3.22703 Z4.65619

N60490 X6.13948 Y3.22703 Z4.6039

N60500 X6.1674 Y3.22703 Z4.53415

N60510 X6.18678 Y3.22703 Z4.48463

N60520 X6.19926 Y3.22703 Z4.45258

N60530 X6.23703 Y3.22703 Z4.34135

N60540 X6.24879 Y3.22703 Z4.30385

N60550 X6.25962 Y3.22703 Z4.26622

N60560 X6.28735 Y3.22703 Z4.16955

N60570 X6.30729 Y3.22703 Z4.09671

N60580 X6.32629 Y3.22703 Z4.03929

N60590 X6.361 Y3.22703 Z3.93531

N60600 X6.4366 Y3.10687 Z3.90107

N60610 X6.41599 Y3.10687 Z3.96447

N60620 X6.39158 Y3.10687 Z4.03693

N60630 X6.37747 Y3.10687 Z4.09081

N60640 X6.36213 Y3.10687 Z4.14799

N60650 X6.3486 Y3.10687 Z4.19966

N60660 X6.33185 Y3.10687 Z4.26375

N60670 X6.32317 Y3.10687 Z4.29492

N60680 X6.30684 Y3.10687 Z4.35202

N60690 X6.29993 Y3.10687 Z4.37371

N60700 X6.26932 Y3.10687 Z4.45406

N60710 X6.24764 Y3.10687 Z4.51127

N60720 X6.22713 Y3.10687 Z4.55879

N60730 X6.19983 Y3.10687 Z4.62237

N60740 X6.1812 Y3.10687 Z4.66467

N60750 X6.16777 Y3.10687 Z4.69295

N60760 X6.12662 Y3.10687 Z4.77445

N60770 X6.08502 Y3.10687 Z4.85114

N60780 X5.89059 Y3.10687 Z5.18489

N60790 X5.82928 Y3.10687 Z5.29091

N60800 X5.77883 Y3.10687 Z5.37551

N60810 X5.72966 Y3.10687 Z5.46154

N60820 X5.68507 Y3.10687 Z5.53915

N60830 X5.63211 Y3.10687 Z5.63208

N60840 X5.62036 Y3.10687 Z5.65201

N60850 X5.59665 Y3.10687 Z5.69132

N60860 X5.55228 Y3.10687 Z5.76415

N60870 X5.54383 Y3.10687 Z5.77837

N60880 X5.46566 Y3.10687 Z5.89924

N60890 X5.4537 Y3.10687 Z5.91794

N60900 X5.35493 Y3.10687 Z6.05842

N60910 X5.3453 Y3.10687 Z6.0722

N60920 X5.32887 Y3.10687 Z6.0945

N60930 X5.24814 Y3.10687 Z6.20094

N60940 X5.17676 Y3.10687 Z6.28908

N60950 X5.16851 Y3.10687 Z6.29833

N60960 X5.10361 Y3.10687 Z6.3696

N60970 X5.0827 Y3.10687 Z6.39039

N60980 X5.02268 Y3.10687 Z6.44702

N60990 X4.99944 Y3.10687 Z6.46594

N61000 X4.92266 Y3.10687 Z6.52631

N61010 X4.91448 Y3.10687 Z6.53262

N61020 X4.8363 Y3.10687 Z6.58434

N61030 X4.78364 Y3.10687 Z6.61936

N61040 X4.74809 Y3.10687 Z6.64014

N61050 X4.71717 Y3.10687 Z6.65849

N61060 X4.65314 Y3.10687 Z6.69693

N61070 X4.58933 Y3.10687 Z6.7313

N61080 X4.55976 Y3.10687 Z6.74688

N61090 X4.48468 Y3.10687 Z6.7811

N61100 X4.45722 Y3.10687 Z6.7926

N61110 X4.36017 Y3.10687 Z6.8256

N61120 X4.33689 Y3.10687 Z6.83171

N61130 X4.25101 Y3.10687 Z6.85226

N61140 X4.2327 Y3.10687 Z6.85578

N61150 X4.1454 Y3.10687 Z6.87024

N61160 X4.13372 Y3.10687 Z6.87188

N61170 X4.04644 Y3.10687 Z6.88074

N61180 X4.03054 Y3.10687 Z6.88203

N61190 X3.96107 Y3.10687 Z6.88633

N61200 X3.9071 Y3.10687 Z6.88864

N61210 X3.85795 Y3.10687 Z6.89125

N61220 X3.80326 Y3.10687 Z6.89219

N61230 X3.76011 Y3.10687 Z6.89308

N61240 X3.70827 Y3.10687 Z6.89257

N61250 X3.61858 Y3.10687 Z6.89218

N61260 X3.58499 Y3.10687 Z6.8913

N61270 X3.46329 Y3.10687 Z6.88898

N61280 X3.44937 Y3.10687 Z6.88879

N61290 X3.3817 Y3.10687 Z6.88693

N61300 X3.30523 Y3.10687 Z6.8847

N61310 X3.1871 Y3.10687 Z6.8797

N61320 X3.11109 Y3.10687 Z6.87458

N61330 X3.07952 Y3.10687 Z6.87275

N61340 X3.05605 Y3.10687 Z6.87087

N61350 X2.97933 Y3.10687 Z6.86381

N61360 X2.94147 Y3.10687 Z6.85967

N61370 X2.84297 Y3.10687 Z6.84656

N61380 X2.81969 Y3.10687 Z6.84348

N61390 X2.79003 Y3.10687 Z6.83863

N61400 X2.70448 Y3.10687 Z6.82354

N61410 X2.64958 Y3.10687 Z6.8123

N61420 X2.6093 Y3.10687 Z6.80295

N61430 X2.55841 Y3.10687 Z6.79057

N61440 X2.45706 Y3.10687 Z6.76246

N61450 X2.35946 Y3.10687 Z6.73184

N61460 X2.30521 Y3.10687 Z6.71273

N61470 X2.25639 Y3.10687 Z6.69543

N61480 X2.19328 Y3.10687 Z6.67006

N61490 X2.13751 Y3.10687 Z6.64751

N61500 X2.07854 Y3.10687 Z6.62078

N61510 X1.98208 Y3.10687 Z6.57515

N61520 X1.96428 Y3.10687 Z6.5661

N61530 X1.9207 Y3.10687 Z6.54307

N61540 X1.8526 Y3.10687 Z6.50779

N61550 X1.82133 Y3.10687 Z6.49096

N61560 X1.73888 Y3.10687 Z6.44451

N61570 X1.68942 Y3.10687 Z6.41657

N61580 X1.61403 Y3.10687 Z6.37153

N61590 X1.57453 Y3.10687 Z6.34838

N61600 X1.46018 Y3.10687 Z6.27665

N61610 X1.44815 Y3.10687 Z6.2687

N61620 X1.34023 Y3.10687 Z6.1982

N61630 X1.31125 Y3.10687 Z6.17743

N61640 X1.20985 Y3.10687 Z6.10499

N61650 X1.19469 Y3.10687 Z6.09449

N61660 X1.18453 Y3.10687 Z6.08735

N61670 X1.09947 Y3.10687 Z6.02628

N61680 X1.04759 Y3.10687 Z5.98895

N61690 X1.00883 Y3.10687 Z5.96237

N61700 X0.915259 Y3.10687 Z5.89701

N61710 X0.827808 Y3.10687 Z5.83607

N61720 X0.786669 Y3.10687 Z5.80713

N61730 X0.755857 Y3.10687 Z5.78606

N61740 X0.701793 Y3.10687 Z5.74811

N61750 X0.666693 Y3.10687 Z5.72484

N61760 X0.598701 Y3.10687 Z5.67715

N61770 X0.531473 Y3.10687 Z5.63321

N61780 X0.488763 Y3.10687 Z5.60355

N61790 X0.383877 Y3.10687 Z5.53454

N61800 X0.371557 Y3.10687 Z5.52603

N61810 X0.304608 Y3.10687 Z5.48221

N61820 X0.238722 Y3.10687 Z5.43914

N61830 X0.229316 Y3.10687 Z5.43329

N61840 X0.210133 Y3.10687 Z5.42113

N61850 X0.102428 Y3.10687 Z5.35222

N61860 X-0.0569573 Y3.10687 Z5.25531

N61870 X-0.0816621 Y3.10687 Z5.24088

N61880 X-0.139738 Y3.10687 Z5.21018

N61890 X-0.199445 Y3.10687 Z5.18271

N61900 X-0.215158 Y3.10687 Z5.17667

N61910 X-0.251161 Y3.10687 Z5.16199

N61920 X-0.275637 Y3.10687 Z5.15388

N61930 X-0.289258 Y3.10687 Z5.14929

N61940 X-0.32349 Y3.10687 Z5.13952

N61950 X-0.3454 Y3.10687 Z5.13361

N61960 X-0.384504 Y3.10687 Z5.12526

N61970 X-0.408184 Y3.10687 Z5.12068

N61980 X-0.442712 Y3.10687 Z5.11641

N61990 X-0.498406 Y3.10687 Z5.1122

N62000 X-0.538272 Y3.10687 Z5.1119

N62010 X-0.558883 Y3.10687 Z5.1121

N62020 X-0.580355 Y3.10687 Z5.11484

N62030 X-0.612773 Y3.10687 Z5.11677

N62040 X-0.655836 Y3.10687 Z5.12418

N62050 X-0.676152 Y3.10687 Z5.12875

N62060 X-0.7041 Y3.10687 Z5.13492

N62070 X-0.749957 Y3.10687 Z5.14708

N62080 X-0.765653 Y3.10687 Z5.15208

N62090 X-0.801358 Y3.10687 Z5.16246

N62100 X-0.816465 Y3.10687 Z5.16735

N62110 X-0.857921 Y3.10687 Z5.18121

N62120 X-0.905181 Y3.10687 Z5.19872

N62130 X-0.918213 Y3.10687 Z5.20366

N62140 X-0.94544 Y3.10687 Z5.2148

N62150 X-0.984181 Y3.10687 Z5.22951

N62160 X-1.01024 Y3.10687 Z5.24017

N62170 X-1.06149 Y3.10687 Z5.26152

N62180 X-1.1533 Y3.10687 Z5.30132

N62190 X-1.2523 Y3.10687 Z5.34337

N62200 X-1.33537 Y3.10687 Z5.37778

N62210 X-1.37223 Y3.10687 Z5.39311

N62220 X-1.49205 Y3.10687 Z5.44057

N62230 X-1.52701 Y3.10687 Z5.45412

N62240 X-1.67813 Y3.10687 Z5.50963

N62250 X-1.76574 Y3.10687 Z5.5426

N62260 X-1.86527 Y3.10687 Z5.57813

N62270 X-2.05384 Y3.10687 Z5.64525

N62280 X-2.19556 Y3.10687 Z5.69287

N62290 X-2.25703 Y3.10687 Z5.71349

N62300 X-2.42297 Y3.10687 Z5.76469

N62310 X-2.45124 Y3.10687 Z5.77284

N62320 X-2.57926 Y3.10687 Z5.81002

N62330 X-2.64407 Y3.10687 Z5.82781

N62340 X-2.73996 Y3.10687 Z5.85494

N62350 X-2.86032 Y3.10687 Z5.8878

N62360 X-2.92337 Y3.10687 Z5.90578

N62370 X-2.99918 Y3.10687 Z5.92868

N62380 X-3.11321 Y3.10687 Z5.9601

N62390 X-3.24877 Y3.10687 Z6.00151

N62400 X-3.2592 Y3.10687 Z6.00444

N62410 X-3.40942 Y3.10687 Z6.04586

N62420 X-3.5059 Y3.10687 Z6.07276

N62430 X-3.58913 Y3.10687 Z6.09469

N62440 X-3.70258 Y3.10687 Z6.12447

N62450 X-3.74746 Y3.10687 Z6.13583

N62460 X-3.77598 Y3.10687 Z6.14272

N62470 X-3.88359 Y3.10687 Z6.16688

N62480 X-3.98371 Y3.10687 Z6.18732

N62490 X-3.99989 Y3.10687 Z6.19042

N62500 X-4.02496 Y3.10687 Z6.19459

N62510 X-4.10667 Y3.10687 Z6.20749

N62520 X-4.15699 Y3.10687 Z6.21423

N62530 X-4.21055 Y3.10687 Z6.22096

N62540 X-4.28736 Y3.10687 Z6.22887

N62550 X-4.31676 Y3.10687 Z6.23166

N62560 X-4.34009 Y3.10687 Z6.23333

N62570 X-4.42727 Y3.10687 Z6.23855

N62580 X-4.53129 Y3.10687 Z6.24142

N62590 X-4.5497 Y3.10687 Z6.24147

N62600 X-4.6543 Y3.10687 Z6.23873

N62610 X-4.77239 Y3.10687 Z6.23057

N62620 X-4.88122 Y3.10687 Z6.21709

N62630 X-4.96523 Y3.10687 Z6.20311

N62640 X-4.99556 Y3.10687 Z6.19754

N62650 X-5.11613 Y3.10687 Z6.16985

N62660 X-5.22812 Y3.10687 Z6.13663

N62670 X-5.25175 Y3.10687 Z6.12905

N62680 X-5.3319 Y3.10687 Z6.09965

N62690 X-5.38708 Y3.10687 Z6.07812

N62700 X-5.42794 Y3.10687 Z6.0603

N62710 X-5.5182 Y3.10687 Z6.01902

N62720 X-5.53148 Y3.10687 Z6.01216

N62730 X-5.60221 Y3.10687 Z5.97471

N62740 X-5.63243 Y3.10687 Z5.95736

N62750 X-5.68116 Y3.10687 Z5.92805

N62760 X-5.73354 Y3.10687 Z5.89313

N62770 X-5.75529 Y3.10687 Z5.87842

N62780 X-5.78739 Y3.10687 Z5.85538

N62790 X-5.8253 Y3.10687 Z5.82681

N62800 X-5.85117 Y3.10687 Z5.80646

N62810 X-5.89532 Y3.10687 Z5.77132

N62820 X-5.92975 Y3.10687 Z5.74268

N62830 X-5.9647 Y3.10687 Z5.71151

N62840 X-6.01621 Y3.10687 Z5.66574

N62850 X-6.04627 Y3.10687 Z5.63736

N62860 X-6.08672 Y3.10687 Z5.59845

N62870 X-6.1396 Y3.10687 Z5.5425

N62880 X-6.15353 Y3.10687 Z5.52686

N62890 X-6.20143 Y3.10687 Z5.46874

N62900 X-6.24331 Y3.10687 Z5.41667

N62910 X-6.25476 Y3.10687 Z5.40144

N62920 X-6.28609 Y3.10687 Z5.357

N62930 X-6.30842 Y3.10687 Z5.32475

N62940 X-6.31434 Y3.10687 Z5.31511

N62950 X-6.36086 Y3.10687 Z5.23742

N62960 X-6.36807 Y3.10687 Z5.22391

N62970 X-6.40898 Y3.10687 Z5.14397

N62980 X-6.42018 Y3.10687 Z5.11943

N62990 X-6.47913 Y3.10687 Z4.97222

N63000 X-6.48257 Y3.10687 Z4.96227

N63010 X-6.48613 Y3.10687 Z4.95168

N63020 X-6.51311 Y3.10687 Z4.8688

N63030 X-6.54069 Y3.10687 Z4.78288

N63040 X-6.54488 Y3.10687 Z4.76817

N63050 X-6.54987 Y3.10687 Z4.75058

N63060 X-6.5738 Y3.10687 Z4.66197

N63070 X-6.58976 Y3.10687 Z4.60283

N63080 X-6.59993 Y3.10687 Z4.55983

N63090 X-6.61244 Y3.10687 Z4.49687

N63100 X-6.6368 Y3.10687 Z4.40958

N63110 X-6.64198 Y3.10687 Z4.38909

N63120 X-6.67441 Y2.98459 Z4.37284

N63130 X-6.66718 Y2.98459 Z4.40224

N63140 X-6.64774 Y2.98459 Z4.47679

N63150 X-6.63575 Y2.98459 Z4.53975

N63160 X-6.62834 Y2.98459 Z4.57407

N63170 X-6.61515 Y2.98459 Z4.62722

N63180 X-6.59984 Y2.98459 Z4.68534

N63190 X-6.58242 Y2.98459 Z4.74601

N63200 X-6.56799 Y2.98459 Z4.79392

N63210 X-6.55516 Y2.98459 Z4.83509

N63220 X-6.53187 Y2.98459 Z4.90191

N63230 X-6.45247 Y2.98459 Z5.1048

N63240 X-6.42859 Y2.98459 Z5.1579

N63250 X-6.40509 Y2.98459 Z5.20492

N63260 X-6.37523 Y2.98459 Z5.26109

N63270 X-6.35144 Y2.98459 Z5.30172

N63280 X-6.32356 Y2.98459 Z5.34683

N63290 X-6.28089 Y2.98459 Z5.40849

N63300 X-6.27275 Y2.98459 Z5.41945

N63310 X-6.26554 Y2.98459 Z5.42884

N63320 X-6.21877 Y2.98459 Z5.48641

N63330 X-6.16906 Y2.98459 Z5.54554

N63340 X-6.14857 Y2.98459 Z5.56737

N63350 X-6.09692 Y2.98459 Z5.62041

N63360 X-6.08135 Y2.98459 Z5.63526

N63370 X-6.02184 Y2.98459 Z5.68937

N63380 X-6.0003 Y2.98459 Z5.70864

N63390 X-5.94271 Y2.98459 Z5.75642

N63400 X-5.91639 Y2.98459 Z5.7788

N63410 X-5.86027 Y2.98459 Z5.82208

N63420 X-5.84172 Y2.98459 Z5.83605

N63430 X-5.80346 Y2.98459 Z5.86395

N63440 X-5.76346 Y2.98459 Z5.89204

N63450 X-5.72906 Y2.98459 Z5.91453

N63460 X-5.67894 Y2.98459 Z5.94698

N63470 X-5.6337 Y2.98459 Z5.97349

N63480 X-5.58862 Y2.98459 Z5.99891

N63490 X-5.49662 Y2.98459 Z6.04552

N63500 X-5.39406 Y2.98459 Z6.08969

N63510 X-5.35609 Y2.98459 Z6.1046

N63520 X-5.28715 Y2.98459 Z6.1294

N63530 X-5.2101 Y2.98459 Z6.15411

N63540 X-5.16928 Y2.98459 Z6.16602

N63550 X-5.04916 Y2.98459 Z6.19582

N63560 X-5.02983 Y2.98459 Z6.20024

N63570 X-4.99829 Y2.98459 Z6.2061

N63580 X-4.89448 Y2.98459 Z6.2252

N63590 X-4.79923 Y2.98459 Z6.2375

N63600 X-4.77271 Y2.98459 Z6.24074

N63610 X-4.74522 Y2.98459 Z6.2427

N63620 X-4.65157 Y2.98459 Z6.249

N63630 X-4.56526 Y2.98459 Z6.25089

N63640 X-4.53283 Y2.98459 Z6.25119

N63650 X-4.49958 Y2.98459 Z6.2502

N63660 X-4.41642 Y2.98459 Z6.24732

N63670 X-4.34774 Y2.98459 Z6.24291

N63680 X-4.30504 Y2.98459 Z6.23953

N63690 X-4.25113 Y2.98459 Z6.23437

N63700 X-4.19913 Y2.98459 Z6.22885

N63710 X-4.16409 Y2.98459 Z6.22449

N63720 X-4.09234 Y2.98459 Z6.21452

N63730 X-3.98239 Y2.98459 Z6.19677

N63740 X-3.85318 Y2.98459 Z6.16867

N63750 X-3.76204 Y2.98459 Z6.14749

N63760 X-3.71195 Y2.98459 Z6.13497

N63770 X-3.63316 Y2.98459 Z6.11429

N63780 X-3.53959 Y2.98459 Z6.08917

N63790 X-3.45667 Y2.98459 Z6.06682

N63800 X-3.33444 Y2.98459 Z6.03286

N63810 X-3.17309 Y2.98459 Z5.98766

N63820 X-3.09308 Y2.98459 Z5.96668

N63830 X-2.9498 Y2.98459 Z5.92604

N63840 X-2.83668 Y2.98459 Z5.89775

N63850 X-2.72272 Y2.98459 Z5.86735

N63860 X-2.63849 Y2.98459 Z5.84617

N63870 X-2.51864 Y2.98459 Z5.81403

N63880 X-2.4681 Y2.98459 Z5.8007

N63890 X-2.30472 Y2.98459 Z5.75453

N63900 X-2.14556 Y2.98459 Z5.70617

N63910 X-2.12202 Y2.98459 Z5.6989

N63920 X-1.99365 Y2.98459 Z5.65721

N63930 X-1.94386 Y2.98459 Z5.64091

N63940 X-1.86397 Y2.98459 Z5.61446

N63950 X-1.80209 Y2.98459 Z5.59394

N63960 X-1.73236 Y2.98459 Z5.56864

N63970 X-1.65186 Y2.98459 Z5.53954

N63980 X-1.53785 Y2.98459 Z5.49735

N63990 X-1.51857 Y2.98459 Z5.48987

N64000 X-1.49639 Y2.98459 Z5.48098

N64010 X-1.37986 Y2.98459 Z5.43368

N64020 X-1.26557 Y2.98459 Z5.38507

N64030 X-1.24857 Y2.98459 Z5.37784

N64040 X-1.22596 Y2.98459 Z5.36789

N64050 X-1.13141 Y2.98459 Z5.32491

N64060 X-1.08247 Y2.98459 Z5.30224

N64070 X-1.03369 Y2.98459 Z5.27986

N64080 X-0.953221 Y2.98459 Z5.24336

N64090 X-0.856692 Y2.98459 Z5.20076

N64100 X-0.827193 Y2.98459 Z5.18864

N64110 X-0.776955 Y2.98459 Z5.16975

N64120 X-0.765839 Y2.98459 Z5.16537

N64130 X-0.717799 Y2.98459 Z5.14791

N64140 X-0.678275 Y2.98459 Z5.13547

N64150 X-0.66848 Y2.98459 Z5.13261

N64160 X-0.633247 Y2.98459 Z5.12179

N64170 X-0.600874 Y2.98459 Z5.11441

N64180 X-0.582275 Y2.98459 Z5.1101

N64190 X-0.553107 Y2.98459 Z5.10395

N64200 X-0.524832 Y2.98459 Z5.10103

N64210 X-0.510154 Y2.98459 Z5.09857

N64220 X-0.498149 Y2.98459 Z5.09782

N64230 X-0.463697 Y2.98459 Z5.09568

N64240 X-0.430282 Y2.98459 Z5.09603

N64250 X-0.415696 Y2.98459 Z5.09654

N64260 X-0.384671 Y2.98459 Z5.09839

N64270 X-0.370877 Y2.98459 Z5.10012

N64280 X-0.337917 Y2.98459 Z5.10441

N64290 X-0.312141 Y2.98459 Z5.10984

N64300 X-0.266522 Y2.98459 Z5.12151

N64310 X-0.247708 Y2.98459 Z5.12734

N64320 X-0.217478 Y2.98459 Z5.1366

N64330 X-0.171184 Y2.98459 Z5.15439

N64340 X-0.124655 Y2.98459 Z5.17522

N64350 X-0.103587 Y2.98459 Z5.18627

N64360 X-0.0703328 Y2.98459 Z5.20372

N64370 X0.0918968 Y2.98459 Z5.30272

N64380 X0.191203 Y2.98459 Z5.36829

N64390 X0.271465 Y2.98459 Z5.42307

N64400 X0.285842 Y2.98459 Z5.43311

N64410 X0.301342 Y2.98459 Z5.44439

N64420 X0.380927 Y2.98459 Z5.4999

N64430 X0.458916 Y2.98459 Z5.55736

N64440 X0.480958 Y2.98459 Z5.57343

N64450 X0.55636 Y2.98459 Z5.62964

N64460 X0.584354 Y2.98459 Z5.6517

N64470 X0.650421 Y2.98459 Z5.7007

N64480 X0.702395 Y2.98459 Z5.74111

N64490 X0.753871 Y2.98459 Z5.7787

N64500 X0.816649 Y2.98459 Z5.82494

N64510 X0.854912 Y2.98459 Z5.85422

N64520 X0.919425 Y2.98459 Z5.90258

N64530 X0.947389 Y2.98459 Z5.9232

N64540 X0.959865 Y2.98459 Z5.93231

N64550 X1.03981 Y2.98459 Z5.99359

N64560 X1.13717 Y2.98459 Z6.06867

N64570 X1.25075 Y2.98459 Z6.15507

N64580 X1.26433 Y2.98459 Z6.1654

N64590 X1.38065 Y2.98459 Z6.24818

N64600 X1.49798 Y2.98459 Z6.32646

N64610 X1.53046 Y2.98459 Z6.34669

N64620 X1.6221 Y2.98459 Z6.40436

N64630 X1.67355 Y2.98459 Z6.43502

N64640 X1.75984 Y2.98459 Z6.48649

N64650 X1.80272 Y2.98459 Z6.51081

N64660 X1.91427 Y2.98459 Z6.57317

N64670 X1.9326 Y2.98459 Z6.5826

N64680 X2.02828 Y2.98459 Z6.63169

N64690 X2.05858 Y2.98459 Z6.64667

N64700 X2.13172 Y2.98459 Z6.67998

N64710 X2.18615 Y2.98459 Z6.70455

N64720 X2.2164 Y2.98459 Z6.71674

N64730 X2.28734 Y2.98459 Z6.74431

N64740 X2.31729 Y2.98459 Z6.75485

N64750 X2.37563 Y2.98459 Z6.77435

N64760 X2.45914 Y2.98459 Z6.80061

N64770 X2.55413 Y2.98459 Z6.82681

N64780 X2.63454 Y2.98459 Z6.84652

N64790 X2.72056 Y2.98459 Z6.86386

N64800 X2.79499 Y2.98459 Z6.87568

N64810 X2.80717 Y2.98459 Z6.87767

N64820 X2.8278 Y2.98459 Z6.88041

N64830 X2.89942 Y2.98459 Z6.88938

N64840 X2.93588 Y2.98459 Z6.89353

N64850 X2.99051 Y2.98459 Z6.89942

N64860 X3.06652 Y2.98459 Z6.90566

N64870 X3.07926 Y2.98459 Z6.90666

N64880 X3.17464 Y2.98459 Z6.91139

N64890 X3.24389 Y2.98459 Z6.91342

N64900 X3.28484 Y2.98459 Z6.91499

N64910 X3.36066 Y2.98459 Z6.91626

N64920 X3.38256 Y2.98459 Z6.91646

N64930 X3.40721 Y2.98459 Z6.91642

N64940 X3.48105 Y2.98459 Z6.91651

N64950 X3.51798 Y2.98459 Z6.9171

N64960 X3.60699 Y2.98459 Z6.9159

N64970 X3.66485 Y2.98459 Z6.91603

N64980 X3.72058 Y2.98459 Z6.91431

N64990 X3.77795 Y2.98459 Z6.91304

N65000 X3.81634 Y2.98459 Z6.91097

N65010 X3.90218 Y2.98459 Z6.90641

N65020 X3.93207 Y2.98459 Z6.9038

N65030 X3.98471 Y2.98459 Z6.89937

N65040 X4.03584 Y2.98459 Z6.89538

N65050 X4.06753 Y2.98459 Z6.89263

N65060 X4.1179 Y2.98459 Z6.88663

N65070 X4.17296 Y2.98459 Z6.87892

N65080 X4.21676 Y2.98459 Z6.87126

N65090 X4.27405 Y2.98459 Z6.86013

N65100 X4.33077 Y2.98459 Z6.84631

N65110 X4.37416 Y2.98459 Z6.83473

N65120 X4.4473 Y2.98459 Z6.80955

N65130 X4.47142 Y2.98459 Z6.8013

N65140 X4.49292 Y2.98459 Z6.79239

N65150 X4.56274 Y2.98459 Z6.76116

N65160 X4.6053 Y2.98459 Z6.73949

N65170 X4.65499 Y2.98459 Z6.7143

N65180 X4.72367 Y2.98459 Z6.6773

N65190 X4.73752 Y2.98459 Z6.66909

N65200 X4.74754 Y2.98459 Z6.66322

N65210 X4.81903 Y2.98459 Z6.61991

N65220 X4.88647 Y2.98459 Z6.57808

N65230 X4.8978 Y2.98459 Z6.57037

N65240 X4.91574 Y2.98459 Z6.55776

N65250 X4.97809 Y2.98459 Z6.51266

N65260 X5.01743 Y2.98459 Z6.48146

N65270 X5.05514 Y2.98459 Z6.45001

N65280 X5.08819 Y2.98459 Z6.41838

N65290 X5.12871 Y2.98459 Z6.38001

N65300 X5.18998 Y2.98459 Z6.31503

N65310 X5.21117 Y2.98459 Z6.2917

N65320 X5.25832 Y2.98459 Z6.23629

N65330 X5.31963 Y2.98459 Z6.1608

N65340 X5.32926 Y2.98459 Z6.14808

N65350 X5.39512 Y2.98459 Z6.06098

N65360 X5.45526 Y2.98459 Z5.97921

N65370 X5.49179 Y2.98459 Z5.92727

N65380 X5.55863 Y2.98459 Z5.82866

N65390 X5.59158 Y2.98459 Z5.77758

N65400 X5.66156 Y2.98459 Z5.66782

N65410 X5.67004 Y2.98459 Z5.65439

N65420 X5.68002 Y2.98459 Z5.63802

N65430 X5.74144 Y2.98459 Z5.53445

N65440 X5.79974 Y2.98459 Z5.43584

N65450 X5.82001 Y2.98459 Z5.40095

N65460 X5.83571 Y2.98459 Z5.3747

N65470 X5.91876 Y2.98459 Z5.23262

N65480 X5.98667 Y2.98459 Z5.11848

N65490 X6.11749 Y2.98459 Z4.8944

N65500 X6.16309 Y2.98459 Z4.81179

N65510 X6.19294 Y2.98459 Z4.75709

N65520 X6.19783 Y2.98459 Z4.7471

N65530 X6.20813 Y2.98459 Z4.72598

N65540 X6.25397 Y2.98459 Z4.63125

N65550 X6.2916 Y2.98459 Z4.55105

N65560 X6.30339 Y2.98459 Z4.52324

N65570 X6.31358 Y2.98459 Z4.49858

N65580 X6.34355 Y2.98459 Z4.4236

N65590 X6.36154 Y2.98459 Z4.36897

N65600 X6.37844 Y2.98459 Z4.31832

N65610 X6.39357 Y2.98459 Z4.26232

N65620 X6.40664 Y2.98459 Z4.21526

N65630 X6.42232 Y2.98459 Z4.15464

N65640 X6.43362 Y2.98459 Z4.11067

N65650 X6.45099 Y2.98459 Z4.0451

N65660 X6.46054 Y2.98459 Z4.00673

N65670 X6.48676 Y2.98459 Z3.92565

N65680 X6.50197 Y2.98459 Z3.87795

N65690 X6.5608 Y2.85607 Z3.86317

N65700 X6.55009 Y2.85607 Z3.8964

N65710 X6.51981 Y2.85607 Z3.9933

N65720 X6.51394 Y2.85607 Z4.01835

N65730 X6.49281 Y2.85607 Z4.10267

N65740 X6.48469 Y2.85607 Z4.13479

N65750 X6.46426 Y2.85607 Z4.21181

N65760 X6.44954 Y2.85607 Z4.26399

N65770 X6.43329 Y2.85607 Z4.32101

N65780 X6.40381 Y2.85607 Z4.40699

N65790 X6.39267 Y2.85607 Z4.43981

N65800 X6.35376 Y2.85607 Z4.53355

N65810 X6.34036 Y2.85607 Z4.56494

N65820 X6.33247 Y2.85607 Z4.5808

N65830 X6.27685 Y2.85607 Z4.69323

N65840 X6.186 Y2.85607 Z4.8602

N65850 X6.1721 Y2.85607 Z4.88579

N65860 X6.16479 Y2.85607 Z4.8986

N65870 X6.04505 Y2.85607 Z5.10548

N65880 X5.99173 Y2.85607 Z5.19669

N65890 X5.93527 Y2.85607 Z5.29155

N65900 X5.84779 Y2.85607 Z5.44012

N65910 X5.83603 Y2.85607 Z5.45955

N65920 X5.74208 Y2.85607 Z5.61509

N65930 X5.73608 Y2.85607 Z5.62466

N65940 X5.65554 Y2.85607 Z5.74961

N65950 X5.61317 Y2.85607 Z5.81326

N65960 X5.56134 Y2.85607 Z5.88951

N65970 X5.51274 Y2.85607 Z5.95665

N65980 X5.46975 Y2.85607 Z6.01544

N65990 X5.42787 Y2.85607 Z6.06879

N66000 X5.37905 Y2.85607 Z6.1319

N66010 X5.36384 Y2.85607 Z6.15012

N66020 X5.30709 Y2.85607 Z6.21798

N66030 X5.28659 Y2.85607 Z6.2409

N66040 X5.22807 Y2.85607 Z6.30681

N66050 X5.19928 Y2.85607 Z6.3361

N66060 X5.14859 Y2.85607 Z6.38786

N66070 X5.08763 Y2.85607 Z6.44344

N66080 X5.06509 Y2.85607 Z6.46398

N66090 X5.03704 Y2.85607 Z6.48664

N66100 X4.98115 Y2.85607 Z6.5306

N66110 X4.9115 Y2.85607 Z6.5799

N66120 X4.89678 Y2.85607 Z6.59008

N66130 X4.88305 Y2.85607 Z6.59883

N66140 X4.81736 Y2.85607 Z6.63885

N66150 X4.77577 Y2.85607 Z6.66251

N66160 X4.73678 Y2.85607 Z6.68418

N66170 X4.67463 Y2.85607 Z6.71804

N66180 X4.64974 Y2.85607 Z6.73067

N66190 X4.62517 Y2.85607 Z6.74217

N66200 X4.55546 Y2.85607 Z6.77474

N66210 X4.47629 Y2.85607 Z6.80724

N66220 X4.45318 Y2.85607 Z6.81606

N66230 X4.40598 Y2.85607 Z6.83091

N66240 X4.34465 Y2.85607 Z6.85027

N66250 X4.31754 Y2.85607 Z6.85693

N66260 X4.23193 Y2.85607 Z6.87687

N66270 X4.18371 Y2.85607 Z6.88534

N66280 X4.11089 Y2.85607 Z6.89779

N66290 X4.05411 Y2.85607 Z6.9046

N66300 X3.95358 Y2.85607 Z6.91692

N66310 X3.92934 Y2.85607 Z6.91896

N66320 X3.85929 Y2.85607 Z6.92509

N66330 X3.80808 Y2.85607 Z6.92976

N66340 X3.78155 Y2.85607 Z6.93181

N66350 X3.69884 Y2.85607 Z6.93685

N66360 X3.64343 Y2.85607 Z6.93858

N66370 X3.57144 Y2.85607 Z6.9425

N66380 X3.49771 Y2.85607 Z6.94361

N66390 X3.38814 Y2.85607 Z6.94638

N66400 X3.37701 Y2.85607 Z6.9464

N66410 X3.36598 Y2.85607 Z6.9463

N66420 X3.25917 Y2.85607 Z6.9459

N66430 X3.18338 Y2.85607 Z6.94433

N66440 X3.14558 Y2.85607 Z6.94329

N66450 X3.06504 Y2.85607 Z6.93993

N66460 X3.05346 Y2.85607 Z6.93916

N66470 X2.98138 Y2.85607 Z6.93271

N66480 X2.88118 Y2.85607 Z6.92263

N66490 X2.86908 Y2.85607 Z6.9212

N66500 X2.7572 Y2.85607 Z6.90687

N66510 X2.72834 Y2.85607 Z6.90166

N66520 X2.658 Y2.85607 Z6.88895

N66530 X2.6438 Y2.85607 Z6.88568

N66540 X2.56923 Y2.85607 Z6.86737

N66550 X2.5036 Y2.85607 Z6.84962

N66560 X2.48121 Y2.85607 Z6.84321

N66570 X2.45757 Y2.85607 Z6.8362

N66580 X2.39675 Y2.85607 Z6.81733

N66590 X2.33849 Y2.85607 Z6.79897

N66600 X2.31704 Y2.85607 Z6.79167

N66610 X2.28301 Y2.85607 Z6.77962

N66620 X2.24693 Y2.85607 Z6.76645

N66630 X2.2174 Y2.85607 Z6.7545

N66640 X2.1807 Y2.85607 Z6.73902

N66650 X2.14164 Y2.85607 Z6.72145

N66660 X2.10243 Y2.85607 Z6.70288

N66670 X2.08119 Y2.85607 Z6.69251

N66680 X1.99111 Y2.85607 Z6.64825

N66690 X1.97974 Y2.85607 Z6.64227

N66700 X1.94046 Y2.85607 Z6.6207

N66710 X1.87346 Y2.85607 Z6.58386

N66720 X1.84709 Y2.85607 Z6.56904

N66730 X1.75209 Y2.85607 Z6.51276

N66740 X1.69014 Y2.85607 Z6.47577

N66750 X1.61223 Y2.85607 Z6.42727

N66760 X1.54883 Y2.85607 Z6.38746

N66770 X1.46446 Y2.85607 Z6.33197

N66780 X1.42389 Y2.85607 Z6.3051

N66790 X1.32398 Y2.85607 Z6.23451

N66800 X1.31087 Y2.85607 Z6.22518

N66810 X1.23094 Y2.85607 Z6.16444

N66820 X1.19925 Y2.85607 Z6.14035

N66830 X1.13834 Y2.85607 Z6.09156

N66840 X1.08174 Y2.85607 Z6.04642

N66850 X0.985533 Y2.85607 Z5.96782

N66860 X0.882841 Y2.85607 Z5.8836

N66870 X0.796358 Y2.85607 Z5.81387

N66880 X0.749538 Y2.85607 Z5.77538

N66890 X0.708068 Y2.85607 Z5.74292

N66900 X0.619734 Y2.85607 Z5.67009

N66910 X0.552348 Y2.85607 Z5.61393

N66920 X0.523266 Y2.85607 Z5.5896

N66930 X0.451837 Y2.85607 Z5.5299

N66940 X0.423288 Y2.85607 Z5.50717

N66950 X0.382732 Y2.85607 Z5.47355

N66960 X0.357084 Y2.85607 Z5.45295

N66970 X0.308988 Y2.85607 Z5.4156

N66980 X0.227409 Y2.85607 Z5.35466

N66990 X0.15489 Y2.85607 Z5.3025

N67000 X0.112068 Y2.85607 Z5.27278

N67010 X0.080829 Y2.85607 Z5.25181

N67020 X0.00777525 Y2.85607 Z5.2045

N67030 X-0.0591836 Y2.85607 Z5.16458

N67040 X-0.0747732 Y2.85607 Z5.15639

N67050 X-0.111558 Y2.85607 Z5.13709

N67060 X-0.140192 Y2.85607 Z5.12465

N67070 X-0.152762 Y2.85607 Z5.11905

N67080 X-0.173395 Y2.85607 Z5.11159

N67090 X-0.194878 Y2.85607 Z5.10361

N67100 X-0.214808 Y2.85607 Z5.09793

N67110 X-0.241638 Y2.85607 Z5.08952

N67120 X-0.263558 Y2.85607 Z5.08448

N67130 X-0.294074 Y2.85607 Z5.07724

N67140 X-0.319409 Y2.85607 Z5.07431

N67150 X-0.332256 Y2.85607 Z5.07229

N67160 X-0.34236 Y2.85607 Z5.07185

N67170 X-0.355682 Y2.85607 Z5.07077

N67180 X-0.388928 Y2.85607 Z5.07064

N67190 X-0.426977 Y2.85607 Z5.07188

N67200 X-0.450957 Y2.85607 Z5.07472

N67210 X-0.465671 Y2.85607 Z5.07643

N67220 X-0.487521 Y2.85607 Z5.08115

N67230 X-0.501907 Y2.85607 Z5.08333

N67240 X-0.513656 Y2.85607 Z5.08632

N67250 X-0.539283 Y2.85607 Z5.09237

N67260 X-0.567372 Y2.85607 Z5.10159

N67270 X-0.580171 Y2.85607 Z5.10498

N67280 X-0.597604 Y2.85607 Z5.11099

N67290 X-0.627493 Y2.85607 Z5.12097

N67300 X-0.655048 Y2.85607 Z5.13203

N67310 X-0.68076 Y2.85607 Z5.1411

N67320 X-0.707512 Y2.85607 Z5.15198

N67330 X-0.738703 Y2.85607 Z5.16478

N67340 X-0.781298 Y2.85607 Z5.18408

N67350 X-0.798966 Y2.85607 Z5.19147

N67360 X-0.809668 Y2.85607 Z5.19633

N67370 X-0.863446 Y2.85607 Z5.22126

N67380 X-0.910598 Y2.85607 Z5.2443

N67390 X-0.935454 Y2.85607 Z5.2566

N67400 X-0.998316 Y2.85607 Z5.28837

N67410 X-1.02292 Y2.85607 Z5.30027

N67420 X-1.03681 Y2.85607 Z5.30698

N67430 X-1.1279 Y2.85607 Z5.35115

N67440 X-1.20587 Y2.85607 Z5.38799

N67450 X-1.24609 Y2.85607 Z5.40695

N67460 X-1.3123 Y2.85607 Z5.43699

N67470 X-1.36915 Y2.85607 Z5.46178

N67480 X-1.41618 Y2.85607 Z5.48138

N67490 X-1.48879 Y2.85607 Z5.511

N67500 X-1.54179 Y2.85607 Z5.53154

N67510 X-1.60084 Y2.85607 Z5.5536

N67520 X-1.68479 Y2.85607 Z5.58436

N67530 X-1.69563 Y2.85607 Z5.58799

N67540 X-1.77997 Y2.85607 Z5.61464

N67550 X-1.80694 Y2.85607 Z5.62393

N67560 X-1.89979 Y2.85607 Z5.65273

N67570 X-1.92519 Y2.85607 Z5.6605

N67580 X-2.01866 Y2.85607 Z5.68974

N67590 X-2.05279 Y2.85607 Z5.69992

N67600 X-2.0632 Y2.85607 Z5.70303

N67610 X-2.1919 Y2.85607 Z5.74091

N67620 X-2.21942 Y2.85607 Z5.74823

N67630 X-2.34993 Y2.85607 Z5.78447

N67640 X-2.38936 Y2.85607 Z5.7946

N67650 X-2.51929 Y2.85607 Z5.82817

N67660 X-2.58564 Y2.85607 Z5.84431

N67670 X-2.70854 Y2.85607 Z5.87471

N67680 X-2.79785 Y2.85607 Z5.89531

N67690 X-2.93262 Y2.85607 Z5.92933

N67700 X-3.01451 Y2.85607 Z5.94861

N67710 X-3.2091 Y2.85607 Z6.00029

N67720 X-3.26793 Y2.85607 Z6.01507

N67730 X-3.41925 Y2.85607 Z6.05654

N67740 X-3.50758 Y2.85607 Z6.08025

N67750 X-3.55024 Y2.85607 Z6.09188

N67760 X-3.71266 Y2.85607 Z6.13418

N67770 X-3.76828 Y2.85607 Z6.14757

N67780 X-3.88992 Y2.85607 Z6.17688

N67790 X-3.93466 Y2.85607 Z6.18755

N67800 X-3.96172 Y2.85607 Z6.19335

N67810 X-4.06501 Y2.85607 Z6.21256

N67820 X-4.16142 Y2.85607 Z6.22743

N67830 X-4.18142 Y2.85607 Z6.23

N67840 X-4.21174 Y2.85607 Z6.23343

N67850 X-4.28912 Y2.85607 Z6.24135

N67860 X-4.35117 Y2.85607 Z6.2471

N67870 X-4.40186 Y2.85607 Z6.25073

N67880 X-4.46123 Y2.85607 Z6.25348

N67890 X-4.52038 Y2.85607 Z6.25606

N67900 X-4.58142 Y2.85607 Z6.25629

N67910 X-4.64211 Y2.85607 Z6.25558

N67920 X-4.70809 Y2.85607 Z6.25169

N67930 X-4.76517 Y2.85607 Z6.24821

N67940 X-4.81177 Y2.85607 Z6.24314

N67950 X-4.88738 Y2.85607 Z6.23372

N67960 X-4.99055 Y2.85607 Z6.21613

N67970 X-5.00917 Y2.85607 Z6.21288

N67980 X-5.01926 Y2.85607 Z6.21072

N67990 X-5.12996 Y2.85607 Z6.18501

N68000 X-5.18452 Y2.85607 Z6.17007

N68010 X-5.24881 Y2.85607 Z6.15124

N68020 X-5.33562 Y2.85607 Z6.12188

N68030 X-5.36109 Y2.85607 Z6.11266

N68040 X-5.43025 Y2.85607 Z6.08446

N68050 X-5.46817 Y2.85607 Z6.06887

N68060 X-5.48236 Y2.85607 Z6.06231

N68070 X-5.57047 Y2.85607 Z6.01942

N68080 X-5.63566 Y2.85607 Z5.98391

N68090 X-5.66936 Y2.85607 Z5.96475

N68100 X-5.71302 Y2.85607 Z5.93718

N68110 X-5.76441 Y2.85607 Z5.90469

N68120 X-5.81465 Y2.85607 Z5.86995

N68130 X-5.8621 Y2.85607 Z5.8362

N68140 X-5.8858 Y2.85607 Z5.81813

N68150 X-5.96975 Y2.85607 Z5.75383

N68160 X-5.98811 Y2.85607 Z5.73851

N68170 X-6.07078 Y2.85607 Z5.66715

N68180 X-6.0784 Y2.85607 Z5.66017

N68190 X-6.1094 Y2.85607 Z5.62964

N68200 X-6.15852 Y2.85607 Z5.58086

N68210 X-6.17541 Y2.85607 Z5.56219

N68220 X-6.22841 Y2.85607 Z5.50144

N68230 X-6.28648 Y2.85607 Z5.42825

N68240 X-6.29393 Y2.85607 Z5.41866

N68250 X-6.30949 Y2.85607 Z5.39572

N68260 X-6.35667 Y2.85607 Z5.32566

N68270 X-6.37044 Y2.85607 Z5.30226

N68280 X-6.41843 Y2.85607 Z5.21827

N68290 X-6.43532 Y2.85607 Z5.18437

N68300 X-6.48103 Y2.85607 Z5.08938

N68310 X-6.54519 Y2.85607 Z4.93414

N68320 X-6.56521 Y2.85607 Z4.87981

N68330 X-6.5869 Y2.85607 Z4.81657

N68340 X-6.6229 Y2.85607 Z4.69905

N68350 X-6.63457 Y2.85607 Z4.65418

N68360 X-6.65351 Y2.85607 Z4.57709

N68370 X-6.66444 Y2.85607 Z4.52405

N68380 X-6.67336 Y2.85607 Z4.47415

N68390 X-6.70013 Y2.85607 Z4.3705

N68400 X-6.70266 Y2.85607 Z4.36005

N68410 X-6.72461 Y2.72479 Z4.34795

N68420 X-6.69297 Y2.72479 Z4.47857

N68430 X-6.66903 Y2.72479 Z4.59818

N68440 X-6.6493 Y2.72479 Z4.677

N68450 X-6.63931 Y2.72479 Z4.71397

N68460 X-6.62784 Y2.72479 Z4.75149

N68470 X-6.602 Y2.72479 Z4.83218

N68480 X-6.57359 Y2.72479 Z4.91095

N68490 X-6.55805 Y2.72479 Z4.95218

N68500 X-6.45936 Y2.72479 Z5.16953

N68510 X-6.4504 Y2.72479 Z5.18815

N68520 X-6.3986 Y2.72479 Z5.27935

N68530 X-6.38465 Y2.72479 Z5.30385

N68540 X-6.31158 Y2.72479 Z5.41266

N68550 X-6.28293 Y2.72479 Z5.44901

N68560 X-6.23019 Y2.72479 Z5.51591

N68570 X-6.17421 Y2.72479 Z5.57812

N68580 X-6.14196 Y2.72479 Z5.61274

N68590 X-6.05162 Y2.72479 Z5.69868

N68600 X-6.033 Y2.72479 Z5.71424

N68610 X-5.9487 Y2.72479 Z5.78331

N68620 X-5.93101 Y2.72479 Z5.79688

N68630 X-5.84756 Y2.72479 Z5.85702

N68640 X-5.78536 Y2.72479 Z5.89989

N68650 X-5.74939 Y2.72479 Z5.923

N68660 X-5.68971 Y2.72479 Z5.95901

N68670 X-5.64906 Y2.72479 Z5.98352

N68680 X-5.62282 Y2.72479 Z5.99787

N68690 X-5.54591 Y2.72479 Z6.03755

N68700 X-5.46736 Y2.72479 Z6.07412

N68710 X-5.44009 Y2.72479 Z6.08604

N68720 X-5.36664 Y2.72479 Z6.11482

N68730 X-5.33073 Y2.72479 Z6.12874

N68740 X-5.3174 Y2.72479 Z6.13329

N68750 X-5.21804 Y2.72479 Z6.1651

N68760 X-5.16459 Y2.72479 Z6.17998

N68770 X-5.10302 Y2.72479 Z6.19584

N68780 X-5.00199 Y2.72479 Z6.21804

N68790 X-4.98708 Y2.72479 Z6.221

N68800 X-4.9567 Y2.72479 Z6.22588

N68810 X-4.86936 Y2.72479 Z6.23942

N68820 X-4.81533 Y2.72479 Z6.24551

N68830 X-4.75006 Y2.72479 Z6.25165

N68840 X-4.6583 Y2.72479 Z6.25608

N68850 X-4.62901 Y2.72479 Z6.25741

N68860 X-4.60231 Y2.72479 Z6.25733

N68870 X-4.50771 Y2.72479 Z6.25564

N68880 X-4.41969 Y2.72479 Z6.25005

N68890 X-4.38418 Y2.72479 Z6.24783

N68900 X-4.34826 Y2.72479 Z6.24465

N68910 X-4.26291 Y2.72479 Z6.23486

N68920 X-4.15184 Y2.72479 Z6.22105

N68930 X-4.14038 Y2.72479 Z6.21949

N68940 X-4.01903 Y2.72479 Z6.19808

N68950 X-3.96636 Y2.72479 Z6.18689

N68960 X-3.89148 Y2.72479 Z6.16952

N68970 X-3.80617 Y2.72479 Z6.14861

N68980 X-3.71805 Y2.72479 Z6.12631

N68990 X-3.52837 Y2.72479 Z6.07742

N69000 X-3.50398 Y2.72479 Z6.07101

N69010 X-3.40755 Y2.72479 Z6.04586

N69020 X-3.2841 Y2.72479 Z6.01311

N69030 X-3.25789 Y2.72479 Z6.00662

N69040 X-3.07141 Y2.72479 Z5.9587

N69050 X-3.01241 Y2.72479 Z5.94519

N69060 X-2.86727 Y2.72479 Z5.91038

N69070 X-2.78377 Y2.72479 Z5.89269

N69080 X-2.65845 Y2.72479 Z5.86388

N69090 X-2.5732 Y2.72479 Z5.84508

N69100 X-2.4387 Y2.72479 Z5.81304

N69110 X-2.38963 Y2.72479 Z5.80099

N69120 X-2.25266 Y2.72479 Z5.76615

N69130 X-2.21027 Y2.72479 Z5.75556

N69140 X-2.10569 Y2.72479 Z5.72684

N69150 X-1.99373 Y2.72479 Z5.69543

N69160 X-1.97136 Y2.72479 Z5.68925

N69170 X-1.88805 Y2.72479 Z5.66494

N69180 X-1.81999 Y2.72479 Z5.64453

N69190 X-1.80853 Y2.72479 Z5.64099

N69200 X-1.7817 Y2.72479 Z5.63292

N69210 X-1.67885 Y2.72479 Z5.601

N69220 X-1.57695 Y2.72479 Z5.56768

N69230 X-1.56552 Y2.72479 Z5.56362

N69240 X-1.46009 Y2.72479 Z5.52338

N69250 X-1.37894 Y2.72479 Z5.48958

N69260 X-1.35178 Y2.72479 Z5.47768

N69270 X-1.33263 Y2.72479 Z5.46884

N69280 X-1.23902 Y2.72479 Z5.42489

N69290 X-1.15529 Y2.72479 Z5.38438

N69300 X-1.12487 Y2.72479 Z5.36949

N69310 X-1.06583 Y2.72479 Z5.33986

N69320 X-1.01802 Y2.72479 Z5.31507

N69330 X-0.999319 Y2.72479 Z5.30527

N69340 X-0.924376 Y2.72479 Z5.26583

N69350 X-0.869703 Y2.72479 Z5.23759

N69360 X-0.846661 Y2.72479 Z5.22565

N69370 X-0.808624 Y2.72479 Z5.20676

N69380 X-0.779477 Y2.72479 Z5.19159

N69390 X-0.763313 Y2.72479 Z5.18336

N69400 X-0.714215 Y2.72479 Z5.15915

N69410 X-0.653016 Y2.72479 Z5.13086

N69420 X-0.640174 Y2.72479 Z5.12537

N69430 X-0.584523 Y2.72479 Z5.10002

N69440 X-0.539805 Y2.72479 Z5.08287

N69450 X-0.529498 Y2.72479 Z5.07869

N69460 X-0.495359 Y2.72479 Z5.06581

N69470 X-0.459304 Y2.72479 Z5.05556

N69480 X-0.425621 Y2.72479 Z5.04681

N69490 X-0.406505 Y2.72479 Z5.04356

N69500 X-0.39122 Y2.72479 Z5.04097

N69510 X-0.364701 Y2.72479 Z5.03888

N69520 X-0.328716 Y2.72479 Z5.03681

N69530 X-0.31097 Y2.72479 Z5.03766

N69540 X-0.288142 Y2.72479 Z5.04004

N69550 X-0.271843 Y2.72479 Z5.04164

N69560 X-0.259436 Y2.72479 Z5.04414

N69570 X-0.227529 Y2.72479 Z5.05052

N69580 X-0.180033 Y2.72479 Z5.06477

N69590 X-0.14034 Y2.72479 Z5.07869

N69600 X-0.121619 Y2.72479 Z5.08706

N69610 X-0.10262 Y2.72479 Z5.09518

N69620 X-0.0590056 Y2.72479 Z5.11874

N69630 X-0.0489626 Y2.72479 Z5.12402

N69640 X0.0163011 Y2.72479 Z5.16408

N69650 X0.0278512 Y2.72479 Z5.17163

N69660 X0.0672954 Y2.72479 Z5.1984

N69670 X0.102234 Y2.72479 Z5.22315

N69680 X0.122316 Y2.72479 Z5.23791

N69690 X0.163616 Y2.72479 Z5.26962

N69700 X0.184896 Y2.72479 Z5.28545

N69710 X0.199141 Y2.72479 Z5.29643

N69720 X0.256818 Y2.72479 Z5.34305

N69730 X0.298774 Y2.72479 Z5.37746

N69740 X0.330402 Y2.72479 Z5.40593

N69750 X0.357886 Y2.72479 Z5.42984

N69760 X0.388866 Y2.72479 Z5.4587

N69770 X0.415793 Y2.72479 Z5.48257

N69780 X0.458225 Y2.72479 Z5.52282

N69790 X0.499682 Y2.72479 Z5.55948

N69800 X0.540056 Y2.72479 Z5.5978

N69810 X0.580724 Y2.72479 Z5.63378

N69820 X0.606563 Y2.72479 Z5.65774

N69830 X0.663227 Y2.72479 Z5.70807

N69840 X0.674712 Y2.72479 Z5.71798

N69850 X0.735599 Y2.72479 Z5.77138

N69860 X0.815838 Y2.72479 Z5.84185

N69870 X0.926283 Y2.72479 Z5.93825

N69880 X0.966493 Y2.72479 Z5.97419

N69890 X1.05154 Y2.72479 Z6.04601

N69900 X1.0719 Y2.72479 Z6.06292

N69910 X1.14394 Y2.72479 Z6.12264

N69920 X1.15205 Y2.72479 Z6.12913

N69930 X1.24372 Y2.72479 Z6.20204

N69940 X1.27372 Y2.72479 Z6.2247

N69950 X1.35092 Y2.72479 Z6.28204

N69960 X1.40798 Y2.72479 Z6.32212

N69970 X1.47041 Y2.72479 Z6.36478

N69980 X1.55478 Y2.72479 Z6.42021

N69990 X1.61155 Y2.72479 Z6.45635

N70000 X1.70327 Y2.72479 Z6.51341

N70010 X1.77993 Y2.72479 Z6.56004

N70020 X1.83429 Y2.72479 Z6.59209

N70030 X1.89975 Y2.72479 Z6.63036

N70040 X1.93177 Y2.72479 Z6.64854

N70050 X2.01006 Y2.72479 Z6.68964

N70060 X2.03143 Y2.72479 Z6.70078

N70070 X2.10668 Y2.72479 Z6.73789

N70080 X2.11891 Y2.72479 Z6.74364

N70090 X2.17039 Y2.72479 Z6.76739

N70100 X2.18801 Y2.72479 Z6.77534

N70110 X2.25165 Y2.72479 Z6.79999

N70120 X2.30939 Y2.72479 Z6.82129

N70130 X2.32869 Y2.72479 Z6.82797

N70140 X2.35346 Y2.72479 Z6.83617

N70150 X2.4121 Y2.72479 Z6.85459

N70160 X2.45581 Y2.72479 Z6.86749

N70170 X2.51013 Y2.72479 Z6.88278

N70180 X2.55666 Y2.72479 Z6.89631

N70190 X2.62275 Y2.72479 Z6.91337

N70200 X2.66989 Y2.72479 Z6.92433

N70210 X2.75441 Y2.72479 Z6.93891

N70220 X2.77521 Y2.72479 Z6.94253

N70230 X2.79795 Y2.72479 Z6.94544

N70240 X2.87871 Y2.72479 Z6.95463

N70250 X2.92409 Y2.72479 Z6.95956

N70260 X2.98799 Y2.72479 Z6.96492

N70270 X3.08331 Y2.72479 Z6.97171

N70280 X3.10759 Y2.72479 Z6.97322

N70290 X3.21613 Y2.72479 Z6.97605

N70300 X3.24239 Y2.72479 Z6.97655

N70310 X3.29112 Y2.72479 Z6.97632

N70320 X3.36402 Y2.72479 Z6.97583

N70330 X3.43583 Y2.72479 Z6.97344

N70340 X3.49208 Y2.72479 Z6.97057

N70350 X3.52905 Y2.72479 Z6.96764

N70360 X3.63614 Y2.72479 Z6.96162

N70370 X3.72338 Y2.72479 Z6.95323

N70380 X3.77497 Y2.72479 Z6.94925

N70390 X3.81588 Y2.72479 Z6.94551

N70400 X3.92325 Y2.72479 Z6.9329

N70410 X4.00937 Y2.72479 Z6.92192

N70420 X4.06327 Y2.72479 Z6.91379

N70430 X4.11785 Y2.72479 Z6.90369

N70440 X4.19941 Y2.72479 Z6.88966

N70450 X4.2894 Y2.72479 Z6.86852

N70460 X4.3221 Y2.72479 Z6.86087

N70470 X4.41082 Y2.72479 Z6.83419

N70480 X4.43778 Y2.72479 Z6.82604

N70490 X4.44758 Y2.72479 Z6.82246

N70500 X4.54851 Y2.72479 Z6.78396

N70510 X4.62937 Y2.72479 Z6.74821

N70520 X4.65222 Y2.72479 Z6.7375

N70530 X4.66944 Y2.72479 Z6.72877

N70540 X4.75127 Y2.72479 Z6.68636

N70550 X4.81111 Y2.72479 Z6.65357

N70560 X4.84162 Y2.72479 Z6.63622

N70570 X4.90491 Y2.72479 Z6.59748

N70580 X4.92737 Y2.72479 Z6.58245

N70590 X4.98383 Y2.72479 Z6.54254

N70600 X5.0606 Y2.72479 Z6.4825

N70610 X5.0834 Y2.72479 Z6.46428

N70620 X5.10303 Y2.72479 Z6.44673

N70630 X5.18961 Y2.72479 Z6.36913

N70640 X5.2243 Y2.72479 Z6.33393

N70650 X5.28646 Y2.72479 Z6.27075

N70660 X5.32898 Y2.72479 Z6.22329

N70670 X5.43088 Y2.72479 Z6.10528

N70680 X5.47288 Y2.72479 Z6.05517

N70690 X5.52136 Y2.72479 Z5.99324

N70700 X5.56367 Y2.72479 Z5.93872

N70710 X5.61317 Y2.72479 Z5.87051

N70720 X5.65124 Y2.72479 Z5.81683

N70730 X5.71511 Y2.72479 Z5.72197

N70740 X5.74127 Y2.72479 Z5.68196

N70750 X5.82531 Y2.72479 Z5.5484

N70760 X5.84618 Y2.72479 Z5.5149

N70770 X5.9328 Y2.72479 Z5.37071

N70780 X5.97955 Y2.72479 Z5.29361

N70790 X6.04203 Y2.72479 Z5.18753

N70800 X6.26602 Y2.72479 Z4.79986

N70810 X6.31978 Y2.72479 Z4.70211

N70820 X6.36169 Y2.72479 Z4.61988

N70830 X6.38472 Y2.72479 Z4.57415

N70840 X6.43535 Y2.72479 Z4.45857

N70850 X6.47803 Y2.72479 Z4.33717

N70860 X6.49435 Y2.72479 Z4.28225

N70870 X6.51037 Y2.72479 Z4.22651

N70880 X6.53691 Y2.72479 Z4.12712

N70890 X6.54083 Y2.72479 Z4.11174

N70900 X6.5595 Y2.72479 Z4.03573

N70910 X6.56979 Y2.72479 Z3.99182

N70920 X6.57772 Y2.72479 Z3.96704

N70930 X6.61069 Y2.72479 Z3.85894

N70940 X6.65183 Y2.59239 Z3.86343

N70950 X6.62793 Y2.59239 Z3.93794

N70960 X6.60092 Y2.59239 Z4.03427

N70970 X6.57581 Y2.59239 Z4.14107

N70980 X6.54608 Y2.59239 Z4.24875

N70990 X6.52577 Y2.59239 Z4.31571

N71000 X6.51333 Y2.59239 Z4.35584

N71010 X6.49447 Y2.59239 Z4.40956

N71020 X6.4753 Y2.59239 Z4.46028

N71030 X6.45896 Y2.59239 Z4.49961

N71040 X6.43383 Y2.59239 Z4.55701

N71050 X6.398 Y2.59239 Z4.62925

N71060 X6.37837 Y2.59239 Z4.66775

N71070 X6.32327 Y2.59239 Z4.7699

N71080 X6.1297 Y2.59239 Z5.10814

N71090 X6.09495 Y2.59239 Z5.16735

N71100 X6.02753 Y2.59239 Z5.27898

N71110 X5.9604 Y2.59239 Z5.39035

N71120 X5.91925 Y2.59239 Z5.45568

N71130 X5.83049 Y2.59239 Z5.5986

N71140 X5.80416 Y2.59239 Z5.63918

N71150 X5.73061 Y2.59239 Z5.75107

N71160 X5.6982 Y2.59239 Z5.79804

N71170 X5.64332 Y2.59239 Z5.87505

N71180 X5.6062 Y2.59239 Z5.92451

N71190 X5.55628 Y2.59239 Z5.98881

N71200 X5.51946 Y2.59239 Z6.03403

N71210 X5.45689 Y2.59239 Z6.10869

N71220 X5.42733 Y2.59239 Z6.14256

N71230 X5.38074 Y2.59239 Z6.19359

N71240 X5.34061 Y2.59239 Z6.23814

N71250 X5.25876 Y2.59239 Z6.32072

N71260 X5.24821 Y2.59239 Z6.33144

N71270 X5.14604 Y2.59239 Z6.42504

N71280 X5.03941 Y2.59239 Z6.51182

N71290 X4.95656 Y2.59239 Z6.57167

N71300 X4.93847 Y2.59239 Z6.58364

N71310 X4.87704 Y2.59239 Z6.6224

N71320 X4.80119 Y2.59239 Z6.6656

N71330 X4.77139 Y2.59239 Z6.68227

N71340 X4.73785 Y2.59239 Z6.69913

N71350 X4.65775 Y2.59239 Z6.73945

N71360 X4.58134 Y2.59239 Z6.77338

N71370 X4.5439 Y2.59239 Z6.78941

N71380 X4.47098 Y2.59239 Z6.81611

N71390 X4.42585 Y2.59239 Z6.83257

N71400 X4.40545 Y2.59239 Z6.83874

N71410 X4.30139 Y2.59239 Z6.8698

N71420 X4.23755 Y2.59239 Z6.88499

N71430 X4.16975 Y2.59239 Z6.90127

N71440 X4.06036 Y2.59239 Z6.92271

N71450 X4.04354 Y2.59239 Z6.92583

N71460 X4.02589 Y2.59239 Z6.92849

N71470 X3.91431 Y2.59239 Z6.94575

N71480 X3.86131 Y2.59239 Z6.95302

N71490 X3.76617 Y2.59239 Z6.96421

N71500 X3.69588 Y2.59239 Z6.97309

N71510 X3.59341 Y2.59239 Z6.98372

N71520 X3.56096 Y2.59239 Z6.98764

N71530 X3.44619 Y2.59239 Z6.99793

N71540 X3.34818 Y2.59239 Z7.00315

N71550 X3.25006 Y2.59239 Z7.0054

N71560 X3.14998 Y2.59239 Z7.00289

N71570 X3.12628 Y2.59239 Z7.00159

N71580 X3.01769 Y2.59239 Z6.99488

N71590 X2.99449 Y2.59239 Z6.99296

N71600 X2.95121 Y2.59239 Z6.98939

N71610 X2.87436 Y2.59239 Z6.98202

N71620 X2.80597 Y2.59239 Z6.97397

N71630 X2.76475 Y2.59239 Z6.96845

N71640 X2.70498 Y2.59239 Z6.95758

N71650 X2.65022 Y2.59239 Z6.94724

N71660 X2.61845 Y2.59239 Z6.93912

N71670 X2.544 Y2.59239 Z6.9193

N71680 X2.50115 Y2.59239 Z6.90735

N71690 X2.43266 Y2.59239 Z6.88783

N71700 X2.40372 Y2.59239 Z6.87879

N71710 X2.34038 Y2.59239 Z6.85773

N71720 X2.31903 Y2.59239 Z6.85043

N71730 X2.30701 Y2.59239 Z6.84619

N71740 X2.23617 Y2.59239 Z6.81873

N71750 X2.18135 Y2.59239 Z6.79635

N71760 X2.14396 Y2.59239 Z6.78023

N71770 X2.07121 Y2.59239 Z6.7461

N71780 X2.0354 Y2.59239 Z6.72865

N71790 X1.94428 Y2.59239 Z6.68118

N71800 X1.90559 Y2.59239 Z6.65983

N71810 X1.80473 Y2.59239 Z6.60172

N71820 X1.69045 Y2.59239 Z6.53321

N71830 X1.65206 Y2.59239 Z6.51007

N71840 X1.51901 Y2.59239 Z6.4254

N71850 X1.49742 Y2.59239 Z6.41137

N71860 X1.38831 Y2.59239 Z6.33686

N71870 X1.35315 Y2.59239 Z6.31184

N71880 X1.27763 Y2.59239 Z6.256

N71890 X1.22133 Y2.59239 Z6.21202

N71900 X1.17507 Y2.59239 Z6.17542

N71910 X1.09451 Y2.59239 Z6.10882

N71920 X1.08336 Y2.59239 Z6.0996

N71930 X1.05297 Y2.59239 Z6.07349

N71940 X0.992319 Y2.59239 Z6.02159

N71950 X0.970158 Y2.59239 Z6.00181

N71960 X0.885424 Y2.59239 Z5.92608

N71970 X0.860988 Y2.59239 Z5.90333

N71980 X0.799762 Y2.59239 Z5.84563

N71990 X0.756888 Y2.59239 Z5.80613

N72000 X0.734755 Y2.59239 Z5.78612

N72010 X0.634826 Y2.59239 Z5.69052

N72020 X0.588257 Y2.59239 Z5.64535

N72030 X0.576764 Y2.59239 Z5.63464

N72040 X0.557826 Y2.59239 Z5.61645

N72050 X0.530539 Y2.59239 Z5.58958

N72060 X0.484344 Y2.59239 Z5.5438

N72070 X0.47657 Y2.59239 Z5.5362

N72080 X0.418075 Y2.59239 Z5.47559

N72090 X0.369569 Y2.59239 Z5.42593

N72100 X0.354783 Y2.59239 Z5.41176

N72110 X0.321072 Y2.59239 Z5.3787

N72120 X0.29848 Y2.59239 Z5.35634

N72130 X0.230384 Y2.59239 Z5.29288

N72140 X0.201906 Y2.59239 Z5.2662

N72150 X0.174435 Y2.59239 Z5.24225

N72160 X0.13609 Y2.59239 Z5.21015

N72170 X0.0957356 Y2.59239 Z5.17788

N72180 X0.0846751 Y2.59239 Z5.16882

N72190 X0.0408503 Y2.59239 Z5.13558

N72200 X0.0134557 Y2.59239 Z5.11583

N72210 X-0.0215906 Y2.59239 Z5.09258

N72220 X-0.0619609 Y2.59239 Z5.06799

N72230 X-0.078142 Y2.59239 Z5.0595

N72240 X-0.102006 Y2.59239 Z5.04678

N72250 X-0.134098 Y2.59239 Z5.03243

N72260 X-0.157936 Y2.59239 Z5.02419

N72270 X-0.172126 Y2.59239 Z5.01938

N72280 X-0.207678 Y2.59239 Z5.00899

N72290 X-0.257588 Y2.59239 Z5.00055

N72300 X-0.292814 Y2.59239 Z4.99814

N72310 X-0.340531 Y2.59239 Z5.00246

N72320 X-0.364238 Y2.59239 Z5.00629

N72330 X-0.389409 Y2.59239 Z5.01169

N72340 X-0.418422 Y2.59239 Z5.02019

N72350 X-0.433712 Y2.59239 Z5.0253

N72360 X-0.459853 Y2.59239 Z5.03378

N72370 X-0.481164 Y2.59239 Z5.04263

N72380 X-0.512019 Y2.59239 Z5.05643

N72390 X-0.541606 Y2.59239 Z5.06931

N72400 X-0.623766 Y2.59239 Z5.11135

N72410 X-0.721012 Y2.59239 Z5.163

N72420 X-0.736809 Y2.59239 Z5.17126

N72430 X-0.822909 Y2.59239 Z5.2181

N72440 X-0.832955 Y2.59239 Z5.22378

N72450 X-0.912576 Y2.59239 Z5.2664

N72460 X-0.964705 Y2.59239 Z5.29472

N72470 X-1.0124 Y2.59239 Z5.32056

N72480 X-1.11892 Y2.59239 Z5.37684

N72490 X-1.13241 Y2.59239 Z5.38358

N72500 X-1.23527 Y2.59239 Z5.43374

N72510 X-1.28774 Y2.59239 Z5.45895

N72520 X-1.35018 Y2.59239 Z5.48764

N72530 X-1.42467 Y2.59239 Z5.5209

N72540 X-1.43965 Y2.59239 Z5.52699

N72550 X-1.52154 Y2.59239 Z5.55719

N72560 X-1.56236 Y2.59239 Z5.57153

N72570 X-1.63034 Y2.59239 Z5.5934

N72580 X-1.72766 Y2.59239 Z5.62406

N72590 X-1.7476 Y2.59239 Z5.62987

N72600 X-1.79634 Y2.59239 Z5.64432

N72610 X-1.87938 Y2.59239 Z5.66769

N72620 X-1.90871 Y2.59239 Z5.67589

N72630 X-2.03409 Y2.59239 Z5.70964

N72640 X-2.08889 Y2.59239 Z5.72314

N72650 X-2.24359 Y2.59239 Z5.76278

N72660 X-2.26349 Y2.59239 Z5.76719

N72670 X-2.28023 Y2.59239 Z5.7715

N72680 X-2.43464 Y2.59239 Z5.80825

N72690 X-2.47092 Y2.59239 Z5.81728

N72700 X-2.54165 Y2.59239 Z5.83229

N72710 X-2.63996 Y2.59239 Z5.85366

N72720 X-2.74433 Y2.59239 Z5.87313

N72730 X-2.86579 Y2.59239 Z5.89934

N72740 X-2.94054 Y2.59239 Z5.91498

N72750 X-3.07335 Y2.59239 Z5.94608

N72760 X-3.12532 Y2.59239 Z5.9583

N72770 X-3.31164 Y2.59239 Z6.00543

N72780 X-3.33015 Y2.59239 Z6.01

N72790 X-3.41748 Y2.59239 Z6.03278

N72800 X-3.55915 Y2.59239 Z6.06912

N72810 X-3.58227 Y2.59239 Z6.07521

N72820 X-3.75402 Y2.59239 Z6.11964

N72830 X-3.7942 Y2.59239 Z6.12978

N72840 X-3.91325 Y2.59239 Z6.15852

N72850 X-4.02644 Y2.59239 Z6.18339

N72860 X-4.07948 Y2.59239 Z6.19491

N72870 X-4.19796 Y2.59239 Z6.21474

N72880 X-4.25305 Y2.59239 Z6.22439

N72890 X-4.38815 Y2.59239 Z6.24163

N72900 X-4.50459 Y2.59239 Z6.25038

N72910 X-4.59911 Y2.59239 Z6.25299

N72920 X-4.61858 Y2.59239 Z6.25339

N72930 X-4.63327 Y2.59239 Z6.25306

N72940 X-4.73262 Y2.59239 Z6.24926

N72950 X-4.81762 Y2.59239 Z6.24247

N72960 X-4.84697 Y2.59239 Z6.23955

N72970 X-4.90287 Y2.59239 Z6.23178

N72980 X-4.96099 Y2.59239 Z6.22333

N72990 X-4.98661 Y2.59239 Z6.21874

N73000 X-5.07438 Y2.59239 Z6.20078

N73010 X-5.14345 Y2.59239 Z6.18427

N73020 X-5.18741 Y2.59239 Z6.17268

N73030 X-5.29925 Y2.59239 Z6.13895

N73040 X-5.31172 Y2.59239 Z6.13439

N73050 X-5.40828 Y2.59239 Z6.09854

N73060 X-5.44297 Y2.59239 Z6.08436

N73070 X-5.51497 Y2.59239 Z6.05256

N73080 X-5.59427 Y2.59239 Z6.01435

N73090 X-5.61903 Y2.59239 Z6.00149

N73100 X-5.66453 Y2.59239 Z5.97598

N73110 X-5.71953 Y2.59239 Z5.94488

N73120 X-5.74691 Y2.59239 Z5.92861

N73130 X-5.81673 Y2.59239 Z5.88343

N73140 X-5.89097 Y2.59239 Z5.83341

N73150 X-5.9136 Y2.59239 Z5.81698

N73160 X-5.97763 Y2.59239 Z5.76771

N73170 X-6.01055 Y2.59239 Z5.74226

N73180 X-6.02253 Y2.59239 Z5.73243

N73190 X-6.10668 Y2.59239 Z5.65707

N73200 X-6.14731 Y2.59239 Z5.61787

N73210 X-6.20086 Y2.59239 Z5.56096

N73220 X-6.25658 Y2.59239 Z5.49767

N73230 X-6.30105 Y2.59239 Z5.44096

N73240 X-6.33999 Y2.59239 Z5.3888

N73250 X-6.39499 Y2.59239 Z5.30566

N73260 X-6.41073 Y2.59239 Z5.28089

N73270 X-6.4489 Y2.59239 Z5.21356

N73280 X-6.47159 Y2.59239 Z5.17317

N73290 X-6.47938 Y2.59239 Z5.15741

N73300 X-6.56252 Y2.59239 Z4.97643

N73310 X-6.57178 Y2.59239 Z4.9542

N73320 X-6.58156 Y2.59239 Z4.92799

N73330 X-6.61415 Y2.59239 Z4.83776

N73340 X-6.64266 Y2.59239 Z4.74854

N73350 X-6.65153 Y2.59239 Z4.7191

N73360 X-6.6657 Y2.59239 Z4.66398

N73370 X-6.68395 Y2.59239 Z4.59114

N73380 X-6.68778 Y2.59239 Z4.57078

N73390 X-6.70904 Y2.59239 Z4.4667

N73400 X-6.72428 Y2.59239 Z4.40869

N73410 X-6.74264 Y2.59239 Z4.3362

N73420 X-6.75594 Y2.46016 Z4.32442

N73430 X-6.74961 Y2.46016 Z4.34894

N73440 X-6.72135 Y2.46016 Z4.45338

N73450 X-6.70156 Y2.46016 Z4.54362

N73460 X-6.69307 Y2.46016 Z4.58277

N73470 X-6.67031 Y2.46016 Z4.67424

N73480 X-6.6601 Y2.46016 Z4.71355

N73490 X-6.65264 Y2.46016 Z4.73886

N73500 X-6.62292 Y2.46016 Z4.83494

N73510 X-6.58743 Y2.46016 Z4.93503

N73520 X-6.58248 Y2.46016 Z4.94864

N73530 X-6.57786 Y2.46016 Z4.95993

N73540 X-6.5373 Y2.46016 Z5.05453

N73550 X-6.48842 Y2.46016 Z5.15705

N73560 X-6.47149 Y2.46016 Z5.18802

N73570 X-6.43307 Y2.46016 Z5.25733

N73580 X-6.4131 Y2.46016 Z5.29018

N73590 X-6.37101 Y2.46016 Z5.35612

N73600 X-6.32541 Y2.46016 Z5.421

N73610 X-6.30113 Y2.46016 Z5.45357

N73620 X-6.23615 Y2.46016 Z5.53214

N73630 X-6.21675 Y2.46016 Z5.55417

N73640 X-6.14966 Y2.46016 Z5.62234

N73650 X-6.10859 Y2.46016 Z5.66181

N73660 X-6.06223 Y2.46016 Z5.70187

N73670 X-5.98194 Y2.46016 Z5.76793

N73680 X-5.9709 Y2.46016 Z5.7762

N73690 X-5.94646 Y2.46016 Z5.79374

N73700 X-5.87714 Y2.46016 Z5.84301

N73710 X-5.84513 Y2.46016 Z5.86484

N73720 X-5.78181 Y2.46016 Z5.90438

N73730 X-5.70174 Y2.46016 Z5.95261

N73740 X-5.68436 Y2.46016 Z5.96215

N73750 X-5.64389 Y2.46016 Z5.98347

N73760 X-5.58321 Y2.46016 Z6.01496

N73770 X-5.555 Y2.46016 Z6.02872

N73780 X-5.47841 Y2.46016 Z6.06295

N73790 X-5.409 Y2.46016 Z6.09175

N73800 X-5.37108 Y2.46016 Z6.10608

N73810 X-5.26441 Y2.46016 Z6.1431

N73820 X-5.15128 Y2.46016 Z6.17453

N73830 X-5.11732 Y2.46016 Z6.18286

N73840 X-5.03923 Y2.46016 Z6.19969

N73850 X-4.96779 Y2.46016 Z6.21295

N73860 X-4.92767 Y2.46016 Z6.21913

N73870 X-4.82537 Y2.46016 Z6.23212

N73880 X-4.71142 Y2.46016 Z6.23983

N73890 X-4.65841 Y2.46016 Z6.24153

N73900 X-4.6102 Y2.46016 Z6.2422

N73910 X-4.53405 Y2.46016 Z6.24074

N73920 X-4.51256 Y2.46016 Z6.24006

N73930 X-4.48649 Y2.46016 Z6.23808

N73940 X-4.41157 Y2.46016 Z6.23155

N73950 X-4.37327 Y2.46016 Z6.22677

N73960 X-4.30064 Y2.46016 Z6.21706

N73970 X-4.22156 Y2.46016 Z6.20394

N73980 X-4.18224 Y2.46016 Z6.19701

N73990 X-4.07893 Y2.46016 Z6.1741

N74000 X-4.00792 Y2.46016 Z6.15862

N74010 X-3.80886 Y2.46016 Z6.10965

N74020 X-3.78076 Y2.46016 Z6.10263

N74030 X-3.76049 Y2.46016 Z6.09737

N74040 X-3.46925 Y2.46016 Z6.02299

N74050 X-3.4487 Y2.46016 Z6.01763

N74060 X-3.38924 Y2.46016 Z6.00294

N74070 X-3.20526 Y2.46016 Z5.956

N74080 X-3.14154 Y2.46016 Z5.94025

N74090 X-3.02741 Y2.46016 Z5.91277

N74100 X-2.95759 Y2.46016 Z5.89691

N74110 X-2.85602 Y2.46016 Z5.8755

N74120 X-2.78863 Y2.46016 Z5.8619

N74130 X-2.66827 Y2.46016 Z5.84102

N74140 X-2.64638 Y2.46016 Z5.83673

N74150 X-2.61472 Y2.46016 Z5.83108

N74160 X-2.53508 Y2.46016 Z5.81593

N74170 X-2.49192 Y2.46016 Z5.80724

N74180 X-2.44635 Y2.46016 Z5.79692

N74190 X-2.41323 Y2.46016 Z5.78933

N74200 X-2.35555 Y2.46016 Z5.77517

N74210 X-2.25443 Y2.46016 Z5.75344

N74220 X-2.23407 Y2.46016 Z5.74821

N74230 X-2.20258 Y2.46016 Z5.74123

N74240 X-2.10342 Y2.46016 Z5.71751

N74250 X-2.04987 Y2.46016 Z5.70606

N74260 X-1.97537 Y2.46016 Z5.68738

N74270 X-1.915 Y2.46016 Z5.67156

N74280 X-1.8213 Y2.46016 Z5.64654

N74290 X-1.78564 Y2.46016 Z5.63651

N74300 X-1.67903 Y2.46016 Z5.60568

N74310 X-1.66524 Y2.46016 Z5.60151

N74320 X-1.54949 Y2.46016 Z5.56322

N74330 X-1.51747 Y2.46016 Z5.55221

N74340 X-1.43282 Y2.46016 Z5.52046

N74350 X-1.39956 Y2.46016 Z5.50686

N74360 X-1.30752 Y2.46016 Z5.46824

N74370 X-1.29575 Y2.46016 Z5.46277

N74380 X-1.20457 Y2.46016 Z5.42069

N74390 X-1.16869 Y2.46016 Z5.40312

N74400 X-1.11451 Y2.46016 Z5.37586

N74410 X-1.08441 Y2.46016 Z5.35963

N74420 X-1.02002 Y2.46016 Z5.32503

N74430 X-0.958013 Y2.46016 Z5.29044

N74440 X-0.917169 Y2.46016 Z5.26762

N74450 X-0.817831 Y2.46016 Z5.21172

N74460 X-0.796712 Y2.46016 Z5.19943

N74470 X-0.787552 Y2.46016 Z5.19403

N74480 X-0.66483 Y2.46016 Z5.12374

N74490 X-0.560657 Y2.46016 Z5.06415

N74500 X-0.545453 Y2.46016 Z5.05581

N74510 X-0.500395 Y2.46016 Z5.03123

N74520 X-0.430384 Y2.46016 Z4.9985

N74530 X-0.393923 Y2.46016 Z4.98501

N74540 X-0.356984 Y2.46016 Z4.97326

N74550 X-0.326316 Y2.46016 Z4.9658

N74560 X-0.313634 Y2.46016 Z4.96323

N74570 X-0.291486 Y2.46016 Z4.95892

N74580 X-0.274269 Y2.46016 Z4.95729

N74590 X-0.260967 Y2.46016 Z4.9572

N74600 X-0.242582 Y2.46016 Z4.95717

N74610 X-0.209091 Y2.46016 Z4.96021

N74620 X-0.195635 Y2.46016 Z4.96215

N74630 X-0.183215 Y2.46016 Z4.96528

N74640 X-0.169599 Y2.46016 Z4.96918

N74650 X-0.154704 Y2.46016 Z4.97374

N74660 X-0.135632 Y2.46016 Z4.98048

N74670 X-0.116012 Y2.46016 Z4.98978

N74680 X-0.0824552 Y2.46016 Z5.00809

N74690 X-0.0512927 Y2.46016 Z5.02743

N74700 X-0.039986 Y2.46016 Z5.03499

N74710 X-0.0171535 Y2.46016 Z5.05112

N74720 X-0.00365919 Y2.46016 Z5.06094

N74730 X0.0338385 Y2.46016 Z5.09008

N74740 X0.0821888 Y2.46016 Z5.13091

N74750 X0.135071 Y2.46016 Z5.17812

N74760 X0.184385 Y2.46016 Z5.225

N74770 X0.220222 Y2.46016 Z5.26086

N74780 X0.231388 Y2.46016 Z5.27211

N74790 X0.248223 Y2.46016 Z5.29006

N74800 X0.292055 Y2.46016 Z5.33529

N74810 X0.317729 Y2.46016 Z5.36254

N74820 X0.354941 Y2.46016 Z5.40445

N74830 X0.401357 Y2.46016 Z5.45652

N74840 X0.408151 Y2.46016 Z5.46418

N74850 X0.470794 Y2.46016 Z5.53221

N74860 X0.538514 Y2.46016 Z5.60453

N74870 X0.561407 Y2.46016 Z5.62901

N74880 X0.637509 Y2.46016 Z5.70817

N74890 X0.658051 Y2.46016 Z5.72802

N74900 X0.74911 Y2.46016 Z5.82055

N74910 X0.805512 Y2.46016 Z5.87464

N74920 X0.838038 Y2.46016 Z5.90639

N74930 X0.916115 Y2.46016 Z5.97904

N74940 X0.935557 Y2.46016 Z5.99701

N74950 X1.0177 Y2.46016 Z6.07028

N74960 X1.02976 Y2.46016 Z6.08075

N74970 X1.09725 Y2.46016 Z6.13849

N74980 X1.16296 Y2.46016 Z6.19272

N74990 X1.20146 Y2.46016 Z6.22447

N75000 X1.29539 Y2.46016 Z6.29763

N75010 X1.30692 Y2.46016 Z6.30632

N75020 X1.36627 Y2.46016 Z6.34848

N75030 X1.4226 Y2.46016 Z6.38856

N75040 X1.43516 Y2.46016 Z6.39715

N75050 X1.56828 Y2.46016 Z6.48365

N75060 X1.5881 Y2.46016 Z6.49626

N75070 X1.70856 Y2.46016 Z6.56885

N75080 X1.77222 Y2.46016 Z6.6071

N75090 X1.78113 Y2.46016 Z6.61232

N75100 X1.79992 Y2.46016 Z6.62277

N75110 X1.95146 Y2.46016 Z6.70656

N75120 X2.01229 Y2.46016 Z6.73658

N75130 X2.08556 Y2.46016 Z6.77222

N75140 X2.15843 Y2.46016 Z6.804

N75150 X2.20585 Y2.46016 Z6.82427

N75160 X2.29786 Y2.46016 Z6.86072

N75170 X2.31509 Y2.46016 Z6.86713

N75180 X2.39631 Y2.46016 Z6.89559

N75190 X2.44019 Y2.46016 Z6.90922

N75200 X2.49456 Y2.46016 Z6.92596

N75210 X2.55036 Y2.46016 Z6.94208

N75220 X2.58867 Y2.46016 Z6.95209

N75230 X2.66155 Y2.46016 Z6.97074

N75240 X2.67225 Y2.46016 Z6.97291

N75250 X2.75006 Y2.46016 Z6.98628

N75260 X2.81229 Y2.46016 Z6.99538

N75270 X2.85171 Y2.46016 Z7.00018

N75280 X2.91949 Y2.46016 Z7.00753

N75290 X2.95545 Y2.46016 Z7.01104

N75300 X2.9736 Y2.46016 Z7.01268

N75310 X3.06202 Y2.46016 Z7.01916

N75320 X3.10948 Y2.46016 Z7.02165

N75330 X3.16911 Y2.46016 Z7.0246

N75340 X3.2572 Y2.46016 Z7.02578

N75350 X3.27132 Y2.46016 Z7.02565

N75360 X3.37812 Y2.46016 Z7.0205

N75370 X3.38903 Y2.46016 Z7.01965

N75380 X3.41332 Y2.46016 Z7.0178

N75390 X3.48903 Y2.46016 Z7.01039

N75400 X3.57287 Y2.46016 Z7.00043

N75410 X3.61424 Y2.46016 Z6.99534

N75420 X3.72776 Y2.46016 Z6.98079

N75430 X3.7795 Y2.46016 Z6.97379

N75440 X3.87046 Y2.46016 Z6.96127

N75450 X3.94081 Y2.46016 Z6.94996

N75460 X3.98984 Y2.46016 Z6.94163

N75470 X4.11801 Y2.46016 Z6.91674

N75480 X4.13515 Y2.46016 Z6.91335

N75490 X4.14779 Y2.46016 Z6.91046

N75500 X4.27702 Y2.46016 Z6.87911

N75510 X4.34293 Y2.46016 Z6.85935

N75520 X4.4098 Y2.46016 Z6.83926

N75530 X4.52157 Y2.46016 Z6.79938

N75540 X4.53918 Y2.46016 Z6.79293

N75550 X4.5689 Y2.46016 Z6.7802

N75560 X4.6627 Y2.46016 Z6.74015

N75570 X4.74494 Y2.46016 Z6.69974

N75580 X4.77973 Y2.46016 Z6.68225

N75590 X4.8087 Y2.46016 Z6.66605

N75600 X4.88836 Y2.46016 Z6.62112

N75610 X4.96137 Y2.46016 Z6.5743

N75620 X4.99136 Y2.46016 Z6.55481

N75630 X5.07396 Y2.46016 Z6.49421

N75640 X5.09734 Y2.46016 Z6.47683

N75650 X5.17982 Y2.46016 Z6.40849

N75660 X5.20118 Y2.46016 Z6.39071

N75670 X5.28515 Y2.46016 Z6.314

N75680 X5.29533 Y2.46016 Z6.30474

N75690 X5.32824 Y2.46016 Z6.27252

N75700 X5.37013 Y2.46016 Z6.23121

N75710 X5.39744 Y2.46016 Z6.20212

N75720 X5.43691 Y2.46016 Z6.16001

N75730 X5.45123 Y2.46016 Z6.14454

N75740 X5.51385 Y2.46016 Z6.07265

N75750 X5.5419 Y2.46016 Z6.03972

N75760 X5.5935 Y2.46016 Z5.97626

N75770 X5.62808 Y2.46016 Z5.93222

N75780 X5.67478 Y2.46016 Z5.87004

N75790 X5.71099 Y2.46016 Z5.82011

N75800 X5.76824 Y2.46016 Z5.73745

N75810 X5.79917 Y2.46016 Z5.6922

N75820 X5.89582 Y2.46016 Z5.54413

N75830 X5.91061 Y2.46016 Z5.52167

N75840 X5.92969 Y2.46016 Z5.49217

N75850 X6.02451 Y2.46016 Z5.34093

N75860 X6.08853 Y2.46016 Z5.23821

N75870 X6.10519 Y2.46016 Z5.21052

N75880 X6.18289 Y2.46016 Z5.07788

N75890 X6.20089 Y2.46016 Z5.04689

N75900 X6.29829 Y2.46016 Z4.8752

N75910 X6.37998 Y2.46016 Z4.7253

N75920 X6.44517 Y2.46016 Z4.59628

N75930 X6.45959 Y2.46016 Z4.56472

N75940 X6.49587 Y2.46016 Z4.48293

N75950 X6.53484 Y2.46016 Z4.38402

N75960 X6.54078 Y2.46016 Z4.36714

N75970 X6.57412 Y2.46016 Z4.26524

N75980 X6.59475 Y2.46016 Z4.1961

N75990 X6.60323 Y2.46016 Z4.16695

N76000 X6.62166 Y2.46016 Z4.09304

N76010 X6.64377 Y2.46016 Z4.01208

N76020 X6.65207 Y2.46016 Z3.97818

N76030 X6.67489 Y2.46016 Z3.90815

N76040 X6.68749 Y2.46016 Z3.86701

N76050 X6.7148 Y2.32841 Z3.87867

N76060 X6.71088 Y2.32841 Z3.89148

N76070 X6.66454 Y2.32841 Z4.03634

N76080 X6.64789 Y2.32841 Z4.10013

N76090 X6.61563 Y2.32841 Z4.21007

N76100 X6.60336 Y2.32841 Z4.25164

N76110 X6.58076 Y2.32841 Z4.3182

N76120 X6.55875 Y2.32841 Z4.3826

N76130 X6.5268 Y2.32841 Z4.46255

N76140 X6.50808 Y2.32841 Z4.50932

N76150 X6.49523 Y2.32841 Z4.53747

N76160 X6.44799 Y2.32841 Z4.63995

N76170 X6.39411 Y2.32841 Z4.74602

N76180 X6.37351 Y2.32841 Z4.78643

N76190 X6.16373 Y2.32841 Z5.1608

N76200 X6.14283 Y2.32841 Z5.19673

N76210 X6.04537 Y2.32841 Z5.35444

N76220 X5.96804 Y2.32841 Z5.47538

N76230 X5.94321 Y2.32841 Z5.51376

N76240 X5.9109 Y2.32841 Z5.56285

N76250 X5.84886 Y2.32841 Z5.65511

N76260 X5.82001 Y2.32841 Z5.69711

N76270 X5.76523 Y2.32841 Z5.77674

N76280 X5.72491 Y2.32841 Z5.83357

N76290 X5.68862 Y2.32841 Z5.88358

N76300 X5.65015 Y2.32841 Z5.93451

N76310 X5.60692 Y2.32841 Z5.98969

N76320 X5.57792 Y2.32841 Z6.02487

N76330 X5.51968 Y2.32841 Z6.09347

N76340 X5.50281 Y2.32841 Z6.11217

N76350 X5.43022 Y2.32841 Z6.19073

N76360 X5.42092 Y2.32841 Z6.20009

N76370 X5.34011 Y2.32841 Z6.28008

N76380 X5.31921 Y2.32841 Z6.29918

N76390 X5.24767 Y2.32841 Z6.36425

N76400 X5.20467 Y2.32841 Z6.40012

N76410 X5.146 Y2.32841 Z6.44898

N76420 X5.09447 Y2.32841 Z6.48778

N76430 X5.03663 Y2.32841 Z6.5308

N76440 X4.98172 Y2.32841 Z6.5672

N76450 X4.92296 Y2.32841 Z6.6055

N76460 X4.85253 Y2.32841 Z6.64579

N76470 X4.80436 Y2.32841 Z6.67306

N76480 X4.68399 Y2.32841 Z6.73167

N76490 X4.66623 Y2.32841 Z6.7404

N76500 X4.64832 Y2.32841 Z6.74805

N76510 X4.52672 Y2.32841 Z6.79825

N76520 X4.44788 Y2.32841 Z6.82639

N76530 X4.39928 Y2.32841 Z6.84326

N76540 X4.36212 Y2.32841 Z6.85523

N76550 X4.28491 Y2.32841 Z6.87838

N76560 X4.24412 Y2.32841 Z6.88933

N76570 X4.15105 Y2.32841 Z6.91284

N76580 X4.04257 Y2.32841 Z6.93533

N76590 X4.01972 Y2.32841 Z6.93984

N76600 X3.90932 Y2.32841 Z6.95979

N76610 X3.82681 Y2.32841 Z6.97332

N76620 X3.76654 Y2.32841 Z6.98274

N76630 X3.59981 Y2.32841 Z7.00591

N76640 X3.58821 Y2.32841 Z7.00726

N76650 X3.44317 Y2.32841 Z7.02411

N76660 X3.3107 Y2.32841 Z7.03309

N76670 X3.27767 Y2.32841 Z7.03523

N76680 X3.26381 Y2.32841 Z7.03533

N76690 X3.1302 Y2.32841 Z7.03581

N76700 X3.09728 Y2.32841 Z7.0342

N76710 X3.01532 Y2.32841 Z7.02944

N76720 X2.95854 Y2.32841 Z7.02463

N76730 X2.91642 Y2.32841 Z7.02028

N76740 X2.83377 Y2.32841 Z7.01064

N76750 X2.80904 Y2.32841 Z7.00749

N76760 X2.71514 Y2.32841 Z6.99223

N76770 X2.67322 Y2.32841 Z6.98382

N76780 X2.60408 Y2.32841 Z6.96876

N76790 X2.55938 Y2.32841 Z6.95694

N76800 X2.48372 Y2.32841 Z6.93599

N76810 X2.44348 Y2.32841 Z6.92349

N76820 X2.33641 Y2.32841 Z6.88729

N76830 X2.30538 Y2.32841 Z6.87621

N76840 X2.27504 Y2.32841 Z6.86439

N76850 X2.15249 Y2.32841 Z6.81622

N76860 X2.11428 Y2.32841 Z6.79956

N76870 X2.01642 Y2.32841 Z6.75594

N76880 X1.95324 Y2.32841 Z6.72481

N76890 X1.89115 Y2.32841 Z6.69406

N76900 X1.83458 Y2.32841 Z6.66336

N76910 X1.76537 Y2.32841 Z6.62485

N76920 X1.66642 Y2.32841 Z6.56685

N76930 X1.60031 Y2.32841 Z6.52713

N76940 X1.51186 Y2.32841 Z6.47192

N76950 X1.44005 Y2.32841 Z6.42523

N76960 X1.35973 Y2.32841 Z6.37163

N76970 X1.32933 Y2.32841 Z6.35003

N76980 X1.22478 Y2.32841 Z6.27103

N76990 X1.18181 Y2.32841 Z6.23558

N77000 X1.11171 Y2.32841 Z6.17774

N77010 X1.10267 Y2.32841 Z6.17001

N77020 X1.04428 Y2.32841 Z6.11921

N77030 X0.990323 Y2.32841 Z6.07235

N77040 X0.981542 Y2.32841 Z6.06452

N77050 X0.91508 Y2.32841 Z6.0031

N77060 X0.873346 Y2.32841 Z5.96462

N77070 X0.865763 Y2.32841 Z5.9573

N77080 X0.766526 Y2.32841 Z5.86089

N77090 X0.709422 Y2.32841 Z5.8018

N77100 X0.647484 Y2.32841 Z5.73882

N77110 X0.536003 Y2.32841 Z5.61531

N77120 X0.526677 Y2.32841 Z5.60554

N77130 X0.490928 Y2.32841 Z5.56471

N77140 X0.415584 Y2.32841 Z5.47797

N77150 X0.401458 Y2.32841 Z5.46008

N77160 X0.316039 Y2.32841 Z5.35983

N77170 X0.263666 Y2.32841 Z5.29753

N77180 X0.24188 Y2.32841 Z5.2731

N77190 X0.207123 Y2.32841 Z5.23309

N77200 X0.16272 Y2.32841 Z5.18553

N77210 X0.144812 Y2.32841 Z5.16643

N77220 X0.134286 Y2.32841 Z5.15583

N77230 X0.0833955 Y2.32841 Z5.10453

N77240 X0.0750959 Y2.32841 Z5.09655

N77250 X-0.00804983 Y2.32841 Z5.0214

N77260 X-0.0242618 Y2.32841 Z5.00794

N77270 X-0.0654421 Y2.32841 Z4.97675

N77280 X-0.0855233 Y2.32841 Z4.96348

N77290 X-0.104497 Y2.32841 Z4.95095

N77300 X-0.134988 Y2.32841 Z4.93344

N77310 X-0.145449 Y2.32841 Z4.92904

N77320 X-0.16567 Y2.32841 Z4.92239

N77330 X-0.185698 Y2.32841 Z4.91755

N77340 X-0.197022 Y2.32841 Z4.91572

N77350 X-0.216323 Y2.32841 Z4.91423

N77360 X-0.243348 Y2.32841 Z4.91481

N77370 X-0.265652 Y2.32841 Z4.91728

N77380 X-0.287995 Y2.32841 Z4.92215

N77390 X-0.29783 Y2.32841 Z4.92441

N77400 X-0.329582 Y2.32841 Z4.93271

N77410 X-0.359584 Y2.32841 Z4.94322

N77420 X-0.373478 Y2.32841 Z4.9488

N77430 X-0.405704 Y2.32841 Z4.96149

N77440 X-0.432128 Y2.32841 Z4.97416

N77450 X-0.45975 Y2.32841 Z4.98786

N77460 X-0.512021 Y2.32841 Z5.01657

N77470 X-0.600338 Y2.32841 Z5.06896

N77480 X-0.618145 Y2.32841 Z5.08019

N77490 X-0.706346 Y2.32841 Z5.13347

N77500 X-0.729511 Y2.32841 Z5.14793

N77510 X-0.840281 Y2.32841 Z5.21425

N77520 X-0.956621 Y2.32841 Z5.28077

N77530 X-0.998365 Y2.32841 Z5.30452

N77540 X-1.07528 Y2.32841 Z5.34787

N77550 X-1.09518 Y2.32841 Z5.35862

N77560 X-1.1459 Y2.32841 Z5.38447

N77570 X-1.18035 Y2.32841 Z5.40188

N77580 X-1.19794 Y2.32841 Z5.40992

N77590 X-1.26049 Y2.32841 Z5.43832

N77600 X-1.33068 Y2.32841 Z5.46816

N77610 X-1.3451 Y2.32841 Z5.47396

N77620 X-1.42686 Y2.32841 Z5.50612

N77630 X-1.45485 Y2.32841 Z5.51697

N77640 X-1.48109 Y2.32841 Z5.52623

N77650 X-1.57308 Y2.32841 Z5.55847

N77660 X-1.58814 Y2.32841 Z5.56327

N77670 X-1.69676 Y2.32841 Z5.59628

N77680 X-1.74575 Y2.32841 Z5.60951

N77690 X-1.83066 Y2.32841 Z5.63233

N77700 X-1.93198 Y2.32841 Z5.65738

N77710 X-1.96783 Y2.32841 Z5.66621

N77720 X-2.01762 Y2.32841 Z5.67779

N77730 X-2.10881 Y2.32841 Z5.69698

N77740 X-2.22973 Y2.32841 Z5.72301

N77750 X-2.25344 Y2.32841 Z5.72782

N77760 X-2.26826 Y2.32841 Z5.73123

N77770 X-2.39384 Y2.32841 Z5.75578

N77780 X-2.47629 Y2.32841 Z5.77322

N77790 X-2.52407 Y2.32841 Z5.78216

N77800 X-2.6272 Y2.32841 Z5.80148

N77810 X-2.68043 Y2.32841 Z5.81124

N77820 X-2.76368 Y2.32841 Z5.82587

N77830 X-2.84042 Y2.32841 Z5.84071

N77840 X-2.89581 Y2.32841 Z5.85145

N77850 X-2.98042 Y2.32841 Z5.87013

N77860 X-3.02966 Y2.32841 Z5.88139

N77870 X-3.12714 Y2.32841 Z5.90556

N77880 X-3.20137 Y2.32841 Z5.92444

N77890 X-3.28918 Y2.32841 Z5.94709

N77900 X-3.40806 Y2.32841 Z5.97793

N77910 X-3.4893 Y2.32841 Z5.99805

N77920 X-3.70436 Y2.32841 Z6.05322

N77930 X-3.75755 Y2.32841 Z6.06682

N77940 X-3.98138 Y2.32841 Z6.12288

N77950 X-4.03805 Y2.32841 Z6.13705

N77960 X-4.14664 Y2.32841 Z6.16258

N77970 X-4.21018 Y2.32841 Z6.17712

N77980 X-4.27984 Y2.32841 Z6.19009

N77990 X-4.34246 Y2.32841 Z6.2013

N78000 X-4.36171 Y2.32841 Z6.2039

N78010 X-4.45117 Y2.32841 Z6.21458

N78020 X-4.49177 Y2.32841 Z6.21819

N78030 X-4.54557 Y2.32841 Z6.22121

N78040 X-4.58363 Y2.32841 Z6.22185

N78050 X-4.66301 Y2.32841 Z6.22221

N78060 X-4.67752 Y2.32841 Z6.22184

N78070 X-4.72028 Y2.32841 Z6.22008

N78080 X-4.77704 Y2.32841 Z6.2173

N78090 X-4.79742 Y2.32841 Z6.21607

N78100 X-4.88294 Y2.32841 Z6.20744

N78110 X-4.9398 Y2.32841 Z6.20059

N78120 X-4.99365 Y2.32841 Z6.19198

N78130 X-5.08247 Y2.32841 Z6.1756

N78140 X-5.10588 Y2.32841 Z6.17042

N78150 X-5.19006 Y2.32841 Z6.14958

N78160 X-5.22447 Y2.32841 Z6.14071

N78170 X-5.32652 Y2.32841 Z6.10834

N78180 X-5.36608 Y2.32841 Z6.09476

N78190 X-5.43622 Y2.32841 Z6.0678

N78200 X-5.50721 Y2.32841 Z6.03863

N78210 X-5.54413 Y2.32841 Z6.02186

N78220 X-5.63945 Y2.32841 Z5.97626

N78230 X-5.64981 Y2.32841 Z5.97117

N78240 X-5.74593 Y2.32841 Z5.91762

N78250 X-5.79475 Y2.32841 Z5.88934

N78260 X-5.8407 Y2.32841 Z5.86025

N78270 X-5.92608 Y2.32841 Z5.80407

N78280 X-5.93585 Y2.32841 Z5.79745

N78290 X-6.02187 Y2.32841 Z5.7324

N78300 X-6.06706 Y2.32841 Z5.69676

N78310 X-6.10517 Y2.32841 Z5.66366

N78320 X-6.18502 Y2.32841 Z5.59054

N78330 X-6.26644 Y2.32841 Z5.5017

N78340 X-6.28913 Y2.32841 Z5.47263

N78350 X-6.33229 Y2.32841 Z5.41664

N78360 X-6.3439 Y2.32841 Z5.40022

N78370 X-6.39228 Y2.32841 Z5.32815

N78380 X-6.42631 Y2.32841 Z5.27291

N78390 X-6.44859 Y2.32841 Z5.23524

N78400 X-6.48656 Y2.32841 Z5.16502

N78410 X-6.50003 Y2.32841 Z5.13964

N78420 X-6.54599 Y2.32841 Z5.04128

N78430 X-6.59087 Y2.32841 Z4.93322

N78440 X-6.62766 Y2.32841 Z4.82717

N78450 X-6.65912 Y2.32841 Z4.7236

N78460 X-6.66378 Y2.32841 Z4.70761

N78470 X-6.66938 Y2.32841 Z4.68647

N78480 X-6.69708 Y2.32841 Z4.57691

N78490 X-6.71379 Y2.32841 Z4.50591

N78500 X-6.72856 Y2.32841 Z4.44267

N78510 X-6.764 Y2.32841 Z4.31005

N78520 X-6.76777 Y2.19476 Z4.29839

N78530 X-6.7499 Y2.19476 Z4.36458

N78540 X-6.73088 Y2.19476 Z4.43239

N78550 X-6.72202 Y2.19476 Z4.4686

N78560 X-6.697 Y2.19476 Z4.56882

N78570 X-6.66562 Y2.19476 Z4.69103

N78580 X-6.6616 Y2.19476 Z4.70547

N78590 X-6.6281 Y2.19476 Z4.81695

N78600 X-6.59329 Y2.19476 Z4.91906

N78610 X-6.58844 Y2.19476 Z4.93211

N78620 X-6.54953 Y2.19476 Z5.02699

N78630 X-6.50877 Y2.19476 Z5.116

N78640 X-6.50048 Y2.19476 Z5.13252

N78650 X-6.45597 Y2.19476 Z5.21609

N78660 X-6.40192 Y2.19476 Z5.30672

N78670 X-6.3484 Y2.19476 Z5.38712

N78680 X-6.33273 Y2.19476 Z5.40856

N78690 X-6.28285 Y2.19476 Z5.47349

N78700 X-6.26423 Y2.19476 Z5.49497

N78710 X-6.21758 Y2.19476 Z5.54842

N78720 X-6.15388 Y2.19476 Z5.61193

N78730 X-6.13678 Y2.19476 Z5.62745

N78740 X-6.07185 Y2.19476 Z5.68245

N78750 X-5.98792 Y2.19476 Z5.74777

N78760 X-5.90861 Y2.19476 Z5.80396

N78770 X-5.89871 Y2.19476 Z5.81088

N78780 X-5.80831 Y2.19476 Z5.86726

N78790 X-5.74289 Y2.19476 Z5.90554

N78800 X-5.71291 Y2.19476 Z5.9217

N78810 X-5.65402 Y2.19476 Z5.95168

N78820 X-5.61134 Y2.19476 Z5.97318

N78830 X-5.59104 Y2.19476 Z5.98305

N78840 X-5.50194 Y2.19476 Z6.02201

N78850 X-5.45371 Y2.19476 Z6.0421

N78860 X-5.38545 Y2.19476 Z6.06754

N78870 X-5.31647 Y2.19476 Z6.09151

N78880 X-5.27211 Y2.19476 Z6.10498

N78890 X-5.17533 Y2.19476 Z6.13208

N78900 X-5.16152 Y2.19476 Z6.13532

N78910 X-5.10154 Y2.19476 Z6.14822

N78920 X-5.05 Y2.19476 Z6.15911

N78930 X-5.03779 Y2.19476 Z6.16141

N78940 X-4.93714 Y2.19476 Z6.17611

N78950 X-4.90138 Y2.19476 Z6.18056

N78960 X-4.82729 Y2.19476 Z6.18643

N78970 X-4.76533 Y2.19476 Z6.19042

N78980 X-4.72241 Y2.19476 Z6.1913

N78990 X-4.63917 Y2.19476 Z6.19245

N79000 X-4.61965 Y2.19476 Z6.19188

N79010 X-4.52525 Y2.19476 Z6.1886

N79020 X-4.50941 Y2.19476 Z6.18737

N79030 X-4.41511 Y2.19476 Z6.17834

N79040 X-4.3673 Y2.19476 Z6.17187

N79050 X-4.30303 Y2.19476 Z6.16098

N79060 X-4.21759 Y2.19476 Z6.14334

N79070 X-4.15911 Y2.19476 Z6.12967

N79080 X-4.01036 Y2.19476 Z6.09393

N79090 X-3.94885 Y2.19476 Z6.07843

N79100 X-3.76093 Y2.19476 Z6.02994

N79110 X-3.6841 Y2.19476 Z6.01033

N79120 X-3.48666 Y2.19476 Z5.95864

N79130 X-3.46184 Y2.19476 Z5.95233

N79140 X-3.27308 Y2.19476 Z5.90182

N79150 X-3.12125 Y2.19476 Z5.86208

N79160 X-3.09853 Y2.19476 Z5.85609

N79170 X-2.96698 Y2.19476 Z5.82355

N79180 X-2.95218 Y2.19476 Z5.82034

N79190 X-2.8424 Y2.19476 Z5.797

N79200 X-2.81597 Y2.19476 Z5.79205

N79210 X-2.69715 Y2.19476 Z5.77009

N79220 X-2.66548 Y2.19476 Z5.76437

N79230 X-2.5185 Y2.19476 Z5.73824

N79240 X-2.49493 Y2.19476 Z5.73375

N79250 X-2.41032 Y2.19476 Z5.71857

N79260 X-2.31393 Y2.19476 Z5.70083

N79270 X-2.27366 Y2.19476 Z5.69416

N79280 X-2.1256 Y2.19476 Z5.66581

N79290 X-1.97604 Y2.19476 Z5.63674

N79300 X-1.91914 Y2.19476 Z5.6245

N79310 X-1.87555 Y2.19476 Z5.61434

N79320 X-1.74222 Y2.19476 Z5.58386

N79330 X-1.66512 Y2.19476 Z5.56339

N79340 X-1.60371 Y2.19476 Z5.54704

N79350 X-1.52041 Y2.19476 Z5.52065

N79360 X-1.48837 Y2.19476 Z5.51008

N79370 X-1.36814 Y2.19476 Z5.46571

N79380 X-1.35628 Y2.19476 Z5.46127

N79390 X-1.22493 Y2.19476 Z5.40675

N79400 X-1.20731 Y2.19476 Z5.39879

N79410 X-1.13025 Y2.19476 Z5.36056

N79420 X-1.09762 Y2.19476 Z5.34444

N79430 X-1.08704 Y2.19476 Z5.33864

N79440 X-0.992125 Y2.19476 Z5.28501

N79450 X-0.886871 Y2.19476 Z5.22411

N79460 X-0.875135 Y2.19476 Z5.21722

N79470 X-0.862374 Y2.19476 Z5.20969

N79480 X-0.770487 Y2.19476 Z5.15338

N79490 X-0.714016 Y2.19476 Z5.11832

N79500 X-0.671843 Y2.19476 Z5.09165

N79510 X-0.582157 Y2.19476 Z5.03531

N79520 X-0.539314 Y2.19476 Z5.00951

N79530 X-0.480848 Y2.19476 Z4.97373

N79540 X-0.471335 Y2.19476 Z4.9685

N79550 X-0.453028 Y2.19476 Z4.95943

N79560 X-0.343515 Y2.19476 Z4.90332

N79570 X-0.308837 Y2.19476 Z4.88971

N79580 X-0.276338 Y2.19476 Z4.88071

N79590 X-0.251425 Y2.19476 Z4.87507

N79600 X-0.230681 Y2.19476 Z4.87278

N79610 X-0.220637 Y2.19476 Z4.87196

N79620 X-0.199948 Y2.19476 Z4.87194

N79630 X-0.189203 Y2.19476 Z4.87325

N79640 X-0.175494 Y2.19476 Z4.87642

N79650 X-0.164212 Y2.19476 Z4.87971

N79660 X-0.145677 Y2.19476 Z4.88691

N79670 X-0.135709 Y2.19476 Z4.89159

N79680 X-0.123678 Y2.19476 Z4.89773

N79690 X-0.113398 Y2.19476 Z4.90475

N79700 X-0.0905487 Y2.19476 Z4.92198

N79710 X-0.0816101 Y2.19476 Z4.92877

N79720 X-0.0662165 Y2.19476 Z4.94157

N79730 X0.0263789 Y2.19476 Z5.02708

N79740 X0.106044 Y2.19476 Z5.11206

N79750 X0.119281 Y2.19476 Z5.12719

N79760 X0.156172 Y2.19476 Z5.16967

N79770 X0.193692 Y2.19476 Z5.21513

N79780 X0.208587 Y2.19476 Z5.23281

N79790 X0.271364 Y2.19476 Z5.31153

N79800 X0.295567 Y2.19476 Z5.34001

N79810 X0.390132 Y2.19476 Z5.45976

N79820 X0.420317 Y2.19476 Z5.49452

N79830 X0.522507 Y2.19476 Z5.62018

N79840 X0.56382 Y2.19476 Z5.66611

N79850 X0.640855 Y2.19476 Z5.75467

N79860 X0.675129 Y2.19476 Z5.79138

N79870 X0.797387 Y2.19476 Z5.91643

N79880 X0.909272 Y2.19476 Z6.02444

N79890 X0.944988 Y2.19476 Z6.05737

N79900 X1.02322 Y2.19476 Z6.12796

N79910 X1.06696 Y2.19476 Z6.16602

N79920 X1.14562 Y2.19476 Z6.23325

N79930 X1.17994 Y2.19476 Z6.26157

N79940 X1.25023 Y2.19476 Z6.31836

N79950 X1.2703 Y2.19476 Z6.33346

N79960 X1.3306 Y2.19476 Z6.37656

N79970 X1.41559 Y2.19476 Z6.43348

N79980 X1.44008 Y2.19476 Z6.44923

N79990 X1.45907 Y2.19476 Z6.46125

N80000 X1.57376 Y2.19476 Z6.53232

N80010 X1.61944 Y2.19476 Z6.55967

N80020 X1.70043 Y2.19476 Z6.60697

N80030 X1.76159 Y2.19476 Z6.64117

N80040 X1.82176 Y2.19476 Z6.67407

N80050 X1.88741 Y2.19476 Z6.7077

N80060 X1.93493 Y2.19476 Z6.73094

N80070 X2.03502 Y2.19476 Z6.77788

N80080 X2.06466 Y2.19476 Z6.79108

N80090 X2.18037 Y2.19476 Z6.8405

N80100 X2.20689 Y2.19476 Z6.85092

N80110 X2.30667 Y2.19476 Z6.88871

N80120 X2.40229 Y2.19476 Z6.92064

N80130 X2.4286 Y2.19476 Z6.92952

N80140 X2.53371 Y2.19476 Z6.95878

N80150 X2.55309 Y2.19476 Z6.96415

N80160 X2.66394 Y2.19476 Z6.98904

N80170 X2.69812 Y2.19476 Z6.99535

N80180 X2.78146 Y2.19476 Z7.00981

N80190 X2.80946 Y2.19476 Z7.01383

N80200 X2.88449 Y2.19476 Z7.02371

N80210 X2.96361 Y2.19476 Z7.03265

N80220 X2.98001 Y2.19476 Z7.03425

N80230 X3.00459 Y2.19476 Z7.03626

N80240 X3.06997 Y2.19476 Z7.04073

N80250 X3.1684 Y2.19476 Z7.04353

N80260 X3.27823 Y2.19476 Z7.04145

N80270 X3.33016 Y2.19476 Z7.03848

N80280 X3.39843 Y2.19476 Z7.03433

N80290 X3.48424 Y2.19476 Z7.02533

N80300 X3.52519 Y2.19476 Z7.02056

N80310 X3.58948 Y2.19476 Z7.01198

N80320 X3.66879 Y2.19476 Z7.00072

N80330 X3.70608 Y2.19476 Z6.99498

N80340 X3.81832 Y2.19476 Z6.97682

N80350 X3.91368 Y2.19476 Z6.95995

N80360 X3.95915 Y2.19476 Z6.9515

N80370 X4.08642 Y2.19476 Z6.92609

N80380 X4.19935 Y2.19476 Z6.8996

N80390 X4.2371 Y2.19476 Z6.89008

N80400 X4.29903 Y2.19476 Z6.87314

N80410 X4.39161 Y2.19476 Z6.84527

N80420 X4.41041 Y2.19476 Z6.83891

N80430 X4.50519 Y2.19476 Z6.80572

N80440 X4.55571 Y2.19476 Z6.78703

N80450 X4.628 Y2.19476 Z6.7575

N80460 X4.71444 Y2.19476 Z6.72042

N80470 X4.76254 Y2.19476 Z6.69691

N80480 X4.84705 Y2.19476 Z6.65443

N80490 X4.88748 Y2.19476 Z6.63126

N80500 X4.96953 Y2.19476 Z6.58306

N80510 X5.00147 Y2.19476 Z6.56189

N80520 X5.08445 Y2.19476 Z6.5058

N80530 X5.11116 Y2.19476 Z6.48569

N80540 X5.18973 Y2.19476 Z6.42611

N80550 X5.22518 Y2.19476 Z6.3965

N80560 X5.28445 Y2.19476 Z6.34716

N80570 X5.37305 Y2.19476 Z6.26605

N80580 X5.38131 Y2.19476 Z6.2585

N80590 X5.48143 Y2.19476 Z6.15782

N80600 X5.56134 Y2.19476 Z6.06895

N80610 X5.58185 Y2.19476 Z6.04531

N80620 X5.6303 Y2.19476 Z5.98616

N80630 X5.67392 Y2.19476 Z5.93051

N80640 X5.69413 Y2.19476 Z5.90366

N80650 X5.74538 Y2.19476 Z5.83362

N80660 X5.75153 Y2.19476 Z5.82498

N80670 X5.80379 Y2.19476 Z5.75019

N80680 X5.85668 Y2.19476 Z5.67437

N80690 X5.88381 Y2.19476 Z5.63537

N80700 X5.92688 Y2.19476 Z5.57278

N80710 X5.97107 Y2.19476 Z5.50714

N80720 X6.0227 Y2.19476 Z5.42774

N80730 X6.0725 Y2.19476 Z5.35076

N80740 X6.09422 Y2.19476 Z5.31633

N80750 X6.1982 Y2.19476 Z5.14584

N80760 X6.20622 Y2.19476 Z5.13229

N80770 X6.22914 Y2.19476 Z5.09195

N80780 X6.30335 Y2.19476 Z4.96085

N80790 X6.34395 Y2.19476 Z4.88605

N80800 X6.38741 Y2.19476 Z4.80446

N80810 X6.42124 Y2.19476 Z4.73769

N80820 X6.46091 Y2.19476 Z4.65932

N80830 X6.50799 Y2.19476 Z4.55765

N80840 X6.52336 Y2.19476 Z4.52439

N80850 X6.53303 Y2.19476 Z4.50045

N80860 X6.57824 Y2.19476 Z4.38777

N80870 X6.62184 Y2.19476 Z4.26041

N80880 X6.62927 Y2.19476 Z4.23864

N80890 X6.63559 Y2.19476 Z4.21726

N80900 X6.67832 Y2.19476 Z4.07281

N80910 X6.7165 Y2.19476 Z3.95507

N80920 X6.73462 Y2.19476 Z3.89675

N80930 X6.74874 Y2.06307 Z3.91549

N80940 X6.71528 Y2.06307 Z4.02123

N80950 X6.69342 Y2.06307 Z4.08932

N80960 X6.6506 Y2.06307 Z4.23382

N80970 X6.64458 Y2.06307 Z4.25372

N80980 X6.64107 Y2.06307 Z4.26399

N80990 X6.59379 Y2.06307 Z4.40135

N81000 X6.56162 Y2.06307 Z4.48073

N81010 X6.54033 Y2.06307 Z4.5323

N81020 X6.51445 Y2.06307 Z4.58779

N81030 X6.48065 Y2.06307 Z4.66011

N81040 X6.41441 Y2.06307 Z4.79208

N81050 X6.37424 Y2.06307 Z4.86894

N81060 X6.267 Y2.06307 Z5.06412

N81070 X6.25963 Y2.06307 Z5.07683

N81080 X6.17802 Y2.06307 Z5.21425

N81090 X6.16086 Y2.06307 Z5.24215

N81100 X6.07793 Y2.06307 Z5.37424

N81110 X6.06913 Y2.06307 Z5.38779

N81120 X6.04145 Y2.06307 Z5.42987

N81130 X5.99261 Y2.06307 Z5.50324

N81140 X5.95306 Y2.06307 Z5.5607

N81150 X5.91766 Y2.06307 Z5.61117

N81160 X5.89536 Y2.06307 Z5.64277

N81170 X5.82901 Y2.06307 Z5.73616

N81180 X5.76822 Y2.06307 Z5.82249

N81190 X5.74671 Y2.06307 Z5.85225

N81200 X5.68793 Y2.06307 Z5.93299

N81210 X5.68112 Y2.06307 Z5.94212

N81220 X5.62791 Y2.06307 Z6.00837

N81230 X5.55851 Y2.06307 Z6.09055

N81240 X5.54232 Y2.06307 Z6.10899

N81250 X5.45248 Y2.06307 Z6.20535

N81260 X5.37154 Y2.06307 Z6.28407

N81270 X5.36381 Y2.06307 Z6.29113

N81280 X5.29913 Y2.06307 Z6.34879

N81290 X5.22289 Y2.06307 Z6.41165

N81300 X5.16352 Y2.06307 Z6.45674

N81310 X5.12578 Y2.06307 Z6.48517

N81320 X5.04566 Y2.06307 Z6.54018

N81330 X5.02054 Y2.06307 Z6.55742

N81340 X4.91627 Y2.06307 Z6.62025

N81350 X4.90086 Y2.06307 Z6.62929

N81360 X4.80797 Y2.06307 Z6.67738

N81370 X4.77207 Y2.06307 Z6.69541

N81380 X4.69544 Y2.06307 Z6.72922

N81390 X4.63266 Y2.06307 Z6.75613

N81400 X4.58184 Y2.06307 Z6.77541

N81410 X4.48306 Y2.06307 Z6.81204

N81420 X4.47289 Y2.06307 Z6.81542

N81430 X4.4484 Y2.06307 Z6.82333

N81440 X4.36646 Y2.06307 Z6.84962

N81450 X4.3302 Y2.06307 Z6.86051

N81460 X4.26263 Y2.06307 Z6.87896

N81470 X4.17218 Y2.06307 Z6.90196

N81480 X4.15061 Y2.06307 Z6.90696

N81490 X4.081 Y2.06307 Z6.92245

N81500 X4.02363 Y2.06307 Z6.93497

N81510 X4.00333 Y2.06307 Z6.93912

N81520 X3.88433 Y2.06307 Z6.96224

N81530 X3.81341 Y2.06307 Z6.97521

N81540 X3.73846 Y2.06307 Z6.98817

N81550 X3.61102 Y2.06307 Z7.00879

N81560 X3.59409 Y2.06307 Z7.0113

N81570 X3.55493 Y2.06307 Z7.01662

N81580 X3.46486 Y2.06307 Z7.02803

N81590 X3.42965 Y2.06307 Z7.03216

N81600 X3.34656 Y2.06307 Z7.03882

N81610 X3.3037 Y2.06307 Z7.0407

N81620 X3.21336 Y2.06307 Z7.043

N81630 X3.18871 Y2.06307 Z7.04309

N81640 X3.15267 Y2.06307 Z7.04283

N81650 X3.07758 Y2.06307 Z7.04168

N81660 X2.98481 Y2.06307 Z7.03587

N81670 X2.97046 Y2.06307 Z7.03488

N81680 X2.9497 Y2.06307 Z7.03273

N81690 X2.86249 Y2.06307 Z7.02291

N81700 X2.80603 Y2.06307 Z7.01515

N81710 X2.74697 Y2.06307 Z7.00664

N81720 X2.63612 Y2.06307 Z6.98687

N81730 X2.62546 Y2.06307 Z6.98485

N81740 X2.57729 Y2.06307 Z6.97398

N81750 X2.50239 Y2.06307 Z6.95686

N81760 X2.49163 Y2.06307 Z6.95387

N81770 X2.39284 Y2.06307 Z6.92687

N81780 X2.38221 Y2.06307 Z6.92341

N81790 X2.29878 Y2.06307 Z6.895

N81800 X2.21184 Y2.06307 Z6.86372

N81810 X2.10412 Y2.06307 Z6.81871

N81820 X2.08771 Y2.06307 Z6.81171

N81830 X1.97469 Y2.06307 Z6.7605

N81840 X1.93148 Y2.06307 Z6.7403

N81850 X1.84735 Y2.06307 Z6.6992

N81860 X1.77541 Y2.06307 Z6.66245

N81870 X1.72607 Y2.06307 Z6.63639

N81880 X1.63009 Y2.06307 Z6.5831

N81890 X1.60901 Y2.06307 Z6.57108

N81900 X1.51965 Y2.06307 Z6.51824

N81910 X1.48946 Y2.06307 Z6.50002

N81920 X1.39433 Y2.06307 Z6.43983

N81930 X1.34736 Y2.06307 Z6.40962

N81940 X1.3111 Y2.06307 Z6.38543

N81950 X1.26687 Y2.06307 Z6.35324

N81960 X1.22673 Y2.06307 Z6.32388

N81970 X1.19569 Y2.06307 Z6.29899

N81980 X1.13624 Y2.06307 Z6.25033

N81990 X1.05899 Y2.06307 Z6.18451

N82000 X1.02211 Y2.06307 Z6.15258

N82010 X0.938375 Y2.06307 Z6.07708

N82020 X0.88508 Y2.06307 Z6.02727

N82030 X0.826008 Y2.06307 Z5.97067

N82040 X0.723634 Y2.06307 Z5.86832

N82050 X0.708708 Y2.06307 Z5.85233

N82060 X0.623228 Y2.06307 Z5.76154

N82070 X0.609429 Y2.06307 Z5.74632

N82080 X0.571128 Y2.06307 Z5.70126

N82090 X0.522162 Y2.06307 Z5.64454

N82100 X0.441785 Y2.06307 Z5.54316

N82110 X0.409873 Y2.06307 Z5.50381

N82120 X0.340931 Y2.06307 Z5.41371

N82130 X0.299582 Y2.06307 Z5.35843

N82140 X0.236826 Y2.06307 Z5.27898

N82150 X0.203004 Y2.06307 Z5.23446

N82160 X0.161888 Y2.06307 Z5.18167

N82170 X0.128406 Y2.06307 Z5.13963

N82180 X0.0886844 Y2.06307 Z5.08961

N82190 X0.076638 Y2.06307 Z5.07502

N82200 X-0.00265229 Y2.06307 Z4.98288

N82210 X-0.0333851 Y2.06307 Z4.94987

N82220 X-0.0512569 Y2.06307 Z4.93083

N82230 X-0.0853071 Y2.06307 Z4.89815

N82240 X-0.11691 Y2.06307 Z4.87073

N82250 X-0.128491 Y2.06307 Z4.86236

N82260 X-0.138709 Y2.06307 Z4.85624

N82270 X-0.15558 Y2.06307 Z4.84709

N82280 X-0.172819 Y2.06307 Z4.83909

N82290 X-0.186591 Y2.06307 Z4.83416

N82300 X-0.197094 Y2.06307 Z4.83182

N82310 X-0.211084 Y2.06307 Z4.83073

N82320 X-0.237321 Y2.06307 Z4.83224

N82330 X-0.249352 Y2.06307 Z4.83391

N82340 X-0.272798 Y2.06307 Z4.83922

N82350 X-0.296236 Y2.06307 Z4.84601

N82360 X-0.32207 Y2.06307 Z4.8568

N82370 X-0.357993 Y2.06307 Z4.87427

N82380 X-0.395819 Y2.06307 Z4.89606

N82390 X-0.44207 Y2.06307 Z4.92138

N82400 X-0.607095 Y2.06307 Z5.02406

N82410 X-0.624338 Y2.06307 Z5.03525

N82420 X-0.709363 Y2.06307 Z5.08936

N82430 X-0.760592 Y2.06307 Z5.12162

N82440 X-0.859767 Y2.06307 Z5.18367

N82450 X-0.897774 Y2.06307 Z5.20668

N82460 X-1.03073 Y2.06307 Z5.28575

N82470 X-1.10014 Y2.06307 Z5.32408

N82480 X-1.14381 Y2.06307 Z5.34739

N82490 X-1.23065 Y2.06307 Z5.38867

N82500 X-1.25242 Y2.06307 Z5.39866

N82510 X-1.29277 Y2.06307 Z5.41483

N82520 X-1.3478 Y2.06307 Z5.43653

N82530 X-1.39341 Y2.06307 Z5.45335

N82540 X-1.44761 Y2.06307 Z5.47191

N82550 X-1.5056 Y2.06307 Z5.48963

N82560 X-1.5649 Y2.06307 Z5.50806

N82570 X-1.60663 Y2.06307 Z5.51919

N82580 X-1.71267 Y2.06307 Z5.54502

N82590 X-1.78925 Y2.06307 Z5.56232

N82600 X-1.94146 Y2.06307 Z5.59294

N82610 X-1.98959 Y2.06307 Z5.60219

N82620 X-2.14554 Y2.06307 Z5.6274

N82630 X-2.31716 Y2.06307 Z5.65497

N82640 X-2.36304 Y2.06307 Z5.66155

N82650 X-2.38335 Y2.06307 Z5.6644

N82660 X-2.59835 Y2.06307 Z5.70002

N82670 X-2.656 Y2.06307 Z5.7103

N82680 X-2.76718 Y2.06307 Z5.72994

N82690 X-2.90101 Y2.06307 Z5.75645

N82700 X-2.92392 Y2.06307 Z5.76121

N82710 X-2.98573 Y2.06307 Z5.77648

N82720 X-3.05113 Y2.06307 Z5.79183

N82730 X-3.08159 Y2.06307 Z5.79993

N82740 X-3.18106 Y2.06307 Z5.82589

N82750 X-3.22994 Y2.06307 Z5.83923

N82760 X-3.43343 Y2.06307 Z5.89621

N82770 X-3.48712 Y2.06307 Z5.91139

N82780 X-3.65796 Y2.06307 Z5.95959

N82790 X-3.67682 Y2.06307 Z5.96461

N82800 X-3.8252 Y2.06307 Z6.00433

N82810 X-3.9369 Y2.06307 Z6.03333

N82820 X-4.02602 Y2.06307 Z6.05615

N82830 X-4.16607 Y2.06307 Z6.08979

N82840 X-4.20174 Y2.06307 Z6.09837

N82850 X-4.33114 Y2.06307 Z6.12469

N82860 X-4.35014 Y2.06307 Z6.1283

N82870 X-4.46949 Y2.06307 Z6.14519

N82880 X-4.54793 Y2.06307 Z6.1514

N82890 X-4.5927 Y2.06307 Z6.15483

N82900 X-4.6678 Y2.06307 Z6.15695

N82910 X-4.71881 Y2.06307 Z6.15802

N82920 X-4.77395 Y2.06307 Z6.15681

N82930 X-4.85083 Y2.06307 Z6.15414

N82940 X-4.8799 Y2.06307 Z6.15179

N82950 X-4.98177 Y2.06307 Z6.14118

N82960 X-4.99401 Y2.06307 Z6.13937

N82970 X-5.05741 Y2.06307 Z6.12852

N82980 X-5.11401 Y2.06307 Z6.11853

N82990 X-5.21425 Y2.06307 Z6.09487

N83000 X-5.26026 Y2.06307 Z6.08287

N83010 X-5.32594 Y2.06307 Z6.06283

N83020 X-5.39312 Y2.06307 Z6.04077

N83030 X-5.44902 Y2.06307 Z6.01987

N83040 X-5.58152 Y2.06307 Z5.96494

N83050 X-5.6878 Y2.06307 Z5.91584

N83060 X-5.69944 Y2.06307 Z5.90957

N83070 X-5.7865 Y2.06307 Z5.86271

N83080 X-5.85873 Y2.06307 Z5.81956

N83090 X-5.87405 Y2.06307 Z5.81

N83100 X-5.89626 Y2.06307 Z5.79494

N83110 X-5.96111 Y2.06307 Z5.75057

N83120 X-5.99875 Y2.06307 Z5.72234

N83130 X-6.0471 Y2.06307 Z5.6852

N83140 X-6.12426 Y2.06307 Z5.62023

N83150 X-6.13349 Y2.06307 Z5.61192

N83160 X-6.20626 Y2.06307 Z5.54006

N83170 X-6.25648 Y2.06307 Z5.48393

N83180 X-6.27659 Y2.06307 Z5.46145

N83190 X-6.34446 Y2.06307 Z5.37464

N83200 X-6.40222 Y2.06307 Z5.28962

N83210 X-6.41957 Y2.06307 Z5.26081

N83220 X-6.45583 Y2.06307 Z5.19996

N83230 X-6.50436 Y2.06307 Z5.10846

N83240 X-6.58299 Y2.06307 Z4.92627

N83250 X-6.58731 Y2.06307 Z4.9153

N83260 X-6.59063 Y2.06307 Z4.9058

N83270 X-6.62318 Y2.06307 Z4.80965

N83280 X-6.65491 Y2.06307 Z4.70376

N83290 X-6.6587 Y2.06307 Z4.69071

N83300 X-6.66268 Y2.06307 Z4.67586

N83310 X-6.69219 Y2.06307 Z4.56215

N83320 X-6.72455 Y2.06307 Z4.4361

N83330 X-6.72808 Y2.06307 Z4.4221

N83340 X-6.76682 Y2.06307 Z4.28588

N83350 X-6.762 Y1.9338 Z4.26833

N83360 X-6.7275 Y1.9338 Z4.38906

N83370 X-6.72199 Y1.9338 Z4.40842

N83380 X-6.7038 Y1.9338 Z4.48071

N83390 X-6.68493 Y1.9338 Z4.55364

N83400 X-6.68086 Y1.9338 Z4.56967

N83410 X-6.66501 Y1.9338 Z4.62879

N83420 X-6.64568 Y1.9338 Z4.70026

N83430 X-6.63656 Y1.9338 Z4.73125

N83440 X-6.61184 Y1.9338 Z4.81056

N83450 X-6.57929 Y1.9338 Z4.90558

N83460 X-6.57442 Y1.9338 Z4.91828

N83470 X-6.53899 Y1.9338 Z5.00534

N83480 X-6.50828 Y1.9338 Z5.07288

N83490 X-6.49762 Y1.9338 Z5.09568

N83500 X-6.48524 Y1.9338 Z5.11931

N83510 X-6.45058 Y1.9338 Z5.18425

N83520 X-6.4063 Y1.9338 Z5.25792

N83530 X-6.3986 Y1.9338 Z5.2706

N83540 X-6.36662 Y1.9338 Z5.317

N83550 X-6.3385 Y1.9338 Z5.35744

N83560 X-6.33218 Y1.9338 Z5.36541

N83570 X-6.26717 Y1.9338 Z5.4464

N83580 X-6.25484 Y1.9338 Z5.45971

N83590 X-6.19146 Y1.9338 Z5.52892

N83600 X-6.12652 Y1.9338 Z5.5928

N83610 X-6.11086 Y1.9338 Z5.6079

N83620 X-6.02068 Y1.9338 Z5.6844

N83630 X-5.97052 Y1.9338 Z5.72233

N83640 X-5.93423 Y1.9338 Z5.74916

N83650 X-5.86047 Y1.9338 Z5.79907

N83660 X-5.78494 Y1.9338 Z5.84342

N83670 X-5.74855 Y1.9338 Z5.8622

N83680 X-5.68602 Y1.9338 Z5.89408

N83690 X-5.60417 Y1.9338 Z5.93163

N83700 X-5.56479 Y1.9338 Z5.94872

N83710 X-5.52803 Y1.9338 Z5.96304

N83720 X-5.44262 Y1.9338 Z5.99646

N83730 X-5.37809 Y1.9338 Z6.01785

N83740 X-5.32707 Y1.9338 Z6.0346

N83750 X-5.26519 Y1.9338 Z6.05238

N83760 X-5.20806 Y1.9338 Z6.06719

N83770 X-5.17127 Y1.9338 Z6.07561

N83780 X-5.11302 Y1.9338 Z6.08674

N83790 X-5.06373 Y1.9338 Z6.09617

N83800 X-5.03811 Y1.9338 Z6.10062

N83810 X-4.9381 Y1.9338 Z6.11381

N83820 X-4.91667 Y1.9338 Z6.11605

N83830 X-4.82966 Y1.9338 Z6.12101

N83840 X-4.79056 Y1.9338 Z6.12237

N83850 X-4.73036 Y1.9338 Z6.12196

N83860 X-4.66036 Y1.9338 Z6.12043

N83870 X-4.62323 Y1.9338 Z6.11839

N83880 X-4.5216 Y1.9338 Z6.11055

N83890 X-4.49127 Y1.9338 Z6.1073

N83900 X-4.38261 Y1.9338 Z6.09115

N83910 X-4.35953 Y1.9338 Z6.08787

N83920 X-4.3491 Y1.9338 Z6.08576

N83930 X-4.2184 Y1.9338 Z6.05913

N83940 X-4.15457 Y1.9338 Z6.04393

N83950 X-4.05609 Y1.9338 Z6.02018

N83960 X-3.93976 Y1.9338 Z5.99024

N83970 X-3.89389 Y1.9338 Z5.9779

N83980 X-3.78754 Y1.9338 Z5.94857

N83990 X-3.76972 Y1.9338 Z5.94346

N84000 X-3.64485 Y1.9338 Z5.90685

N84010 X-3.6258 Y1.9338 Z5.90091

N84020 X-3.54638 Y1.9338 Z5.8769

N84030 X-3.46363 Y1.9338 Z5.85205

N84040 X-3.42193 Y1.9338 Z5.84006

N84050 X-3.30097 Y1.9338 Z5.80415

N84060 X-3.22939 Y1.9338 Z5.7837

N84070 X-3.16357 Y1.9338 Z5.76539

N84080 X-3.08233 Y1.9338 Z5.74407

N84090 X-3.04631 Y1.9338 Z5.73433

N84100 X-2.93519 Y1.9338 Z5.70687

N84110 X-2.84643 Y1.9338 Z5.68807

N84120 X-2.82032 Y1.9338 Z5.68275

N84130 X-2.6932 Y1.9338 Z5.65965

N84140 X-2.56701 Y1.9338 Z5.63942

N84150 X-2.51175 Y1.9338 Z5.6306

N84160 X-2.43174 Y1.9338 Z5.61945

N84170 X-2.31039 Y1.9338 Z5.60242

N84180 X-2.29993 Y1.9338 Z5.60092

N84190 X-2.1396 Y1.9338 Z5.58014

N84200 X-2.01327 Y1.9338 Z5.56338

N84210 X-1.96951 Y1.9338 Z5.55636

N84220 X-1.83337 Y1.9338 Z5.53463

N84230 X-1.79337 Y1.9338 Z5.52661

N84240 X-1.69836 Y1.9338 Z5.50688

N84250 X-1.603 Y1.9338 Z5.48489

N84260 X-1.55008 Y1.9338 Z5.47196

N84270 X-1.48459 Y1.9338 Z5.45317

N84280 X-1.43165 Y1.9338 Z5.43768

N84290 X-1.37063 Y1.9338 Z5.4172

N84300 X-1.33373 Y1.9338 Z5.40429

N84310 X-1.31509 Y1.9338 Z5.39731

N84320 X-1.24175 Y1.9338 Z5.36725

N84330 X-1.20747 Y1.9338 Z5.35131

N84340 X-1.13645 Y1.9338 Z5.31637

N84350 X-1.10179 Y1.9338 Z5.29775

N84360 X-1.01686 Y1.9338 Z5.25004

N84370 X-0.975374 Y1.9338 Z5.22527

N84380 X-0.878933 Y1.9338 Z5.16621

N84390 X-0.811192 Y1.9338 Z5.12408

N84400 X-0.727391 Y1.9338 Z5.07068

N84410 X-0.539203 Y1.9338 Z4.95071

N84420 X-0.52495 Y1.9338 Z4.94175

N84430 X-0.449049 Y1.9338 Z4.89445

N84440 X-0.407418 Y1.9338 Z4.86895

N84450 X-0.372648 Y1.9338 Z4.84893

N84460 X-0.331783 Y1.9338 Z4.82536

N84470 X-0.298228 Y1.9338 Z4.80767

N84480 X-0.286862 Y1.9338 Z4.80347

N84490 X-0.26796 Y1.9338 Z4.79831

N84500 X-0.258306 Y1.9338 Z4.79567

N84510 X-0.247607 Y1.9338 Z4.79335

N84520 X-0.235809 Y1.9338 Z4.79242

N84530 X-0.224065 Y1.9338 Z4.79296

N84540 X-0.211608 Y1.9338 Z4.79529

N84550 X-0.200157 Y1.9338 Z4.80029

N84560 X-0.189277 Y1.9338 Z4.80574

N84570 X-0.180204 Y1.9338 Z4.81061

N84580 X-0.154449 Y1.9338 Z4.82566

N84590 X-0.131886 Y1.9338 Z4.8405

N84600 X-0.123352 Y1.9338 Z4.84652

N84610 X-0.113173 Y1.9338 Z4.85607

N84620 X-0.0875101 Y1.9338 Z4.88413

N84630 X-0.0558172 Y1.9338 Z4.9207

N84640 X-0.00967145 Y1.9338 Z4.97512

N84650 X0.120188 Y1.9338 Z5.13956

N84660 X0.1831 Y1.9338 Z5.22261

N84670 X0.19998 Y1.9338 Z5.24589

N84680 X0.272171 Y1.9338 Z5.34293

N84690 X0.28008 Y1.9338 Z5.35396

N84700 X0.294448 Y1.9338 Z5.3736

N84710 X0.366673 Y1.9338 Z5.47046

N84720 X0.437272 Y1.9338 Z5.56463

N84730 X0.456938 Y1.9338 Z5.58943

N84740 X0.521748 Y1.9338 Z5.67198

N84750 X0.560453 Y1.9338 Z5.71776

N84760 X0.586553 Y1.9338 Z5.74863

N84770 X0.646692 Y1.9338 Z5.81455

N84780 X0.657351 Y1.9338 Z5.82631

N84790 X0.740148 Y1.9338 Z5.91187

N84800 X0.809393 Y1.9338 Z5.98035

N84810 X0.845355 Y1.9338 Z6.0152

N84820 X0.953019 Y1.9338 Z6.11543

N84830 X0.962214 Y1.9338 Z6.12403

N84840 X1.08783 Y1.9338 Z6.23289

N84850 X1.0976 Y1.9338 Z6.24121

N84860 X1.11405 Y1.9338 Z6.25467

N84870 X1.20122 Y1.9338 Z6.3241

N84880 X1.26148 Y1.9338 Z6.36912

N84890 X1.27966 Y1.9338 Z6.38204

N84900 X1.33877 Y1.9338 Z6.42058

N84910 X1.37845 Y1.9338 Z6.44649

N84920 X1.43047 Y1.9338 Z6.47861

N84930 X1.50203 Y1.9338 Z6.52241

N84940 X1.53331 Y1.9338 Z6.5409

N84950 X1.6392 Y1.9338 Z6.60132

N84960 X1.76265 Y1.9338 Z6.66609

N84970 X1.80684 Y1.9338 Z6.68816

N84980 X1.89114 Y1.9338 Z6.72907

N84990 X1.98433 Y1.9338 Z6.77233

N85000 X2.02368 Y1.9338 Z6.79002

N85010 X2.13466 Y1.9338 Z6.83758

N85020 X2.15124 Y1.9338 Z6.84459

N85030 X2.26619 Y1.9338 Z6.88783

N85040 X2.36996 Y1.9338 Z6.9217

N85050 X2.39246 Y1.9338 Z6.92882

N85060 X2.40529 Y1.9338 Z6.93234

N85070 X2.55542 Y1.9338 Z6.97036

N85080 X2.58014 Y1.9338 Z6.97529

N85090 X2.70654 Y1.9338 Z6.9992

N85100 X2.78515 Y1.9338 Z7.00985

N85110 X2.84497 Y1.9338 Z7.01802

N85120 X2.91509 Y1.9338 Z7.02571

N85130 X2.96588 Y1.9338 Z7.03059

N85140 X3.00722 Y1.9338 Z7.03356

N85150 X3.06425 Y1.9338 Z7.03717

N85160 X3.12598 Y1.9338 Z7.03777

N85170 X3.17069 Y1.9338 Z7.03807

N85180 X3.23964 Y1.9338 Z7.03584

N85190 X3.29733 Y1.9338 Z7.03483

N85200 X3.37813 Y1.9338 Z7.03025

N85210 X3.41882 Y1.9338 Z7.02686

N85220 X3.53505 Y1.9338 Z7.01378

N85230 X3.67786 Y1.9338 Z6.99184

N85240 X3.73121 Y1.9338 Z6.98292

N85250 X3.82411 Y1.9338 Z6.96628

N85260 X3.92291 Y1.9338 Z6.94767

N85270 X3.97242 Y1.9338 Z6.9378

N85280 X4.10371 Y1.9338 Z6.91053

N85290 X4.1204 Y1.9338 Z6.90671

N85300 X4.2322 Y1.9338 Z6.88014

N85310 X4.28013 Y1.9338 Z6.86835

N85320 X4.32785 Y1.9338 Z6.85532

N85330 X4.41405 Y1.9338 Z6.82992

N85340 X4.42897 Y1.9338 Z6.8251

N85350 X4.47099 Y1.9338 Z6.81113

N85360 X4.53367 Y1.9338 Z6.79008

N85370 X4.55706 Y1.9338 Z6.78218

N85380 X4.63904 Y1.9338 Z6.75095

N85390 X4.69993 Y1.9338 Z6.72718

N85400 X4.74266 Y1.9338 Z6.70831

N85410 X4.83382 Y1.9338 Z6.66663

N85420 X4.84444 Y1.9338 Z6.66113

N85430 X4.89216 Y1.9338 Z6.63556

N85440 X4.94637 Y1.9338 Z6.60647

N85450 X4.95728 Y1.9338 Z6.60037

N85460 X5.04718 Y1.9338 Z6.54464

N85470 X5.06958 Y1.9338 Z6.53034

N85480 X5.1457 Y1.9338 Z6.47717

N85490 X5.16996 Y1.9338 Z6.45985

N85500 X5.24083 Y1.9338 Z6.40557

N85510 X5.25731 Y1.9338 Z6.39266

N85520 X5.32895 Y1.9338 Z6.33284

N85530 X5.33796 Y1.9338 Z6.32518

N85540 X5.41229 Y1.9338 Z6.25717

N85550 X5.4229 Y1.9338 Z6.24659

N85560 X5.48714 Y1.9338 Z6.18103

N85570 X5.50168 Y1.9338 Z6.16546

N85580 X5.56648 Y1.9338 Z6.0923

N85590 X5.60975 Y1.9338 Z6.0419

N85600 X5.65172 Y1.9338 Z5.99011

N85610 X5.72092 Y1.9338 Z5.90005

N85620 X5.75038 Y1.9338 Z5.86089

N85630 X5.82997 Y1.9338 Z5.75047

N85640 X5.87135 Y1.9338 Z5.69283

N85650 X5.9599 Y1.9338 Z5.56834

N85660 X5.97159 Y1.9338 Z5.55154

N85670 X6.0633 Y1.9338 Z5.41759

N85680 X6.15232 Y1.9338 Z5.2801

N85690 X6.16278 Y1.9338 Z5.26356

N85700 X6.2382 Y1.9338 Z5.14064

N85710 X6.25179 Y1.9338 Z5.11806

N85720 X6.38546 Y1.9338 Z4.88198

N85730 X6.39149 Y1.9338 Z4.87045

N85740 X6.43665 Y1.9338 Z4.78269

N85750 X6.46367 Y1.9338 Z4.72877

N85760 X6.50066 Y1.9338 Z4.65402

N85770 X6.53762 Y1.9338 Z4.57655

N85780 X6.56021 Y1.9338 Z4.52834

N85790 X6.59088 Y1.9338 Z4.45625

N85800 X6.60551 Y1.9338 Z4.42101

N85810 X6.65017 Y1.9338 Z4.2919

N85820 X6.69988 Y1.9338 Z4.12116

N85830 X6.71303 Y1.9338 Z4.07788

N85840 X6.75846 Y1.9338 Z3.9324

N85850 X6.76443 Y1.80482 Z3.94716

N85860 X6.75614 Y1.80482 Z3.97563

N85870 X6.69983 Y1.80482 Z4.16122

N85880 X6.69069 Y1.80482 Z4.19536

N85890 X6.64901 Y1.80482 Z4.33721

N85900 X6.63727 Y1.80482 Z4.37123

N85910 X6.59333 Y1.80482 Z4.48444

N85920 X6.57362 Y1.80482 Z4.52993

N85930 X6.5349 Y1.80482 Z4.61187

N85940 X6.50492 Y1.80482 Z4.67372

N85950 X6.46453 Y1.80482 Z4.75512

N85960 X6.44315 Y1.80482 Z4.79737

N85970 X6.39584 Y1.80482 Z4.88734

N85980 X6.32577 Y1.80482 Z5.01317

N85990 X6.23624 Y1.80482 Z5.16287

N86000 X6.16703 Y1.80482 Z5.27252

N86010 X6.14805 Y1.80482 Z5.30249

N86020 X6.12916 Y1.80482 Z5.33093

N86030 X6.05082 Y1.80482 Z5.4488

N86040 X6.04477 Y1.80482 Z5.45772

N86050 X5.94836 Y1.80482 Z5.59601

N86060 X5.92453 Y1.80482 Z5.62972

N86070 X5.83666 Y1.80482 Z5.75187

N86080 X5.80246 Y1.80482 Z5.79861

N86090 X5.70837 Y1.80482 Z5.92372

N86100 X5.68653 Y1.80482 Z5.95196

N86110 X5.66423 Y1.80482 Z5.97958

N86120 X5.58665 Y1.80482 Z6.07484

N86130 X5.53787 Y1.80482 Z6.13024

N86140 X5.50015 Y1.80482 Z6.17204

N86150 X5.43263 Y1.80482 Z6.24319

N86160 X5.36715 Y1.80482 Z6.30439

N86170 X5.34194 Y1.80482 Z6.32603

N86180 X5.29416 Y1.80482 Z6.36651

N86190 X5.25843 Y1.80482 Z6.39494

N86200 X5.21063 Y1.80482 Z6.43233

N86210 X5.16875 Y1.80482 Z6.46297

N86220 X5.11629 Y1.80482 Z6.50046

N86230 X5.07515 Y1.80482 Z6.52757

N86240 X5.01199 Y1.80482 Z6.56801

N86250 X4.97937 Y1.80482 Z6.58707

N86260 X4.89823 Y1.80482 Z6.63258

N86270 X4.88195 Y1.80482 Z6.64088

N86280 X4.82237 Y1.80482 Z6.66962

N86290 X4.78439 Y1.80482 Z6.68785

N86300 X4.77256 Y1.80482 Z6.69327

N86310 X4.68789 Y1.80482 Z6.7279

N86320 X4.63468 Y1.80482 Z6.74877

N86330 X4.58799 Y1.80482 Z6.76515

N86340 X4.48236 Y1.80482 Z6.80088

N86350 X4.46653 Y1.80482 Z6.80558

N86360 X4.36452 Y1.80482 Z6.83555

N86370 X4.34564 Y1.80482 Z6.84119

N86380 X4.28121 Y1.80482 Z6.85776

N86390 X4.22496 Y1.80482 Z6.87182

N86400 X4.17241 Y1.80482 Z6.88393

N86410 X4.09048 Y1.80482 Z6.90201

N86420 X4.04999 Y1.80482 Z6.91065

N86430 X3.93603 Y1.80482 Z6.93379

N86440 X3.85778 Y1.80482 Z6.94889

N86450 X3.77988 Y1.80482 Z6.96316

N86460 X3.67101 Y1.80482 Z6.98194

N86470 X3.63628 Y1.80482 Z6.98757

N86480 X3.5406 Y1.80482 Z7.00172

N86490 X3.51063 Y1.80482 Z7.00596

N86500 X3.49968 Y1.80482 Z7.00726

N86510 X3.39743 Y1.80482 Z7.01804

N86520 X3.33293 Y1.80482 Z7.02208

N86530 X3.28345 Y1.80482 Z7.0246

N86540 X3.24518 Y1.80482 Z7.02586

N86550 X3.17148 Y1.80482 Z7.02735

N86560 X3.08479 Y1.80482 Z7.02815

N86570 X3.0712 Y1.80482 Z7.02746

N86580 X2.9852 Y1.80482 Z7.02346

N86590 X2.92496 Y1.80482 Z7.01908

N86600 X2.87682 Y1.80482 Z7.01443

N86610 X2.78213 Y1.80482 Z7.00476

N86620 X2.73499 Y1.80482 Z6.9985

N86630 X2.66121 Y1.80482 Z6.98799

N86640 X2.58431 Y1.80482 Z6.97356

N86650 X2.5478 Y1.80482 Z6.96641

N86660 X2.44003 Y1.80482 Z6.94102

N86670 X2.42605 Y1.80482 Z6.93748

N86680 X2.32038 Y1.80482 Z6.90604

N86690 X2.26393 Y1.80482 Z6.88743

N86700 X2.20712 Y1.80482 Z6.86693

N86710 X2.12018 Y1.80482 Z6.83298

N86720 X2.08164 Y1.80482 Z6.81786

N86730 X2.05833 Y1.80482 Z6.808

N86740 X1.94738 Y1.80482 Z6.75973

N86750 X1.85521 Y1.80482 Z6.7175

N86760 X1.81115 Y1.80482 Z6.69686

N86770 X1.72319 Y1.80482 Z6.65349

N86780 X1.68346 Y1.80482 Z6.63365

N86790 X1.66848 Y1.80482 Z6.62581

N86800 X1.56832 Y1.80482 Z6.57166

N86810 X1.51454 Y1.80482 Z6.54138

N86820 X1.46188 Y1.80482 Z6.51039

N86830 X1.386 Y1.80482 Z6.46469

N86840 X1.36426 Y1.80482 Z6.45076

N86850 X1.27333 Y1.80482 Z6.39188

N86860 X1.17936 Y1.80482 Z6.32488

N86870 X1.16164 Y1.80482 Z6.31167

N86880 X1.07732 Y1.80482 Z6.24447

N86890 X1.01586 Y1.80482 Z6.19372

N86900 X0.962485 Y1.80482 Z6.1475

N86910 X0.876207 Y1.80482 Z6.06956

N86920 X0.851949 Y1.80482 Z6.04698

N86930 X0.808484 Y1.80482 Z6.00485

N86940 X0.74832 Y1.80482 Z5.94684

N86950 X0.720126 Y1.80482 Z5.91834

N86960 X0.657801 Y1.80482 Z5.8546

N86970 X0.585572 Y1.80482 Z5.7761

N86980 X0.517487 Y1.80482 Z5.69663

N86990 X0.475178 Y1.80482 Z5.64298

N87000 X0.448733 Y1.80482 Z5.60947

N87010 X0.387354 Y1.80482 Z5.52735

N87020 X0.352333 Y1.80482 Z5.48009

N87030 X0.3017 Y1.80482 Z5.41012

N87040 X0.218009 Y1.80482 Z5.29359

N87050 X0.161803 Y1.80482 Z5.21524

N87060 X0.142535 Y1.80482 Z5.18842

N87070 X0.134668 Y1.80482 Z5.17725

N87080 X0.0868766 Y1.80482 Z5.11148

N87090 X0.0640813 Y1.80482 Z5.08074

N87100 X0.0130545 Y1.80482 Z5.01471

N87110 X-0.0298024 Y1.80482 Z4.96148

N87120 X-0.0841136 Y1.80482 Z4.89548

N87130 X-0.0974388 Y1.80482 Z4.88087

N87140 X-0.126947 Y1.80482 Z4.84846

N87150 X-0.143684 Y1.80482 Z4.83284

N87160 X-0.155447 Y1.80482 Z4.82315

N87170 X-0.174088 Y1.80482 Z4.80891

N87180 X-0.211438 Y1.80482 Z4.78327

N87190 X-0.242452 Y1.80482 Z4.76548

N87200 X-0.25241 Y1.80482 Z4.7608

N87210 X-0.262403 Y1.80482 Z4.76388

N87220 X-0.273775 Y1.80482 Z4.76743

N87230 X-0.291228 Y1.80482 Z4.77436

N87240 X-0.31215 Y1.80482 Z4.78368

N87250 X-0.32319 Y1.80482 Z4.78934

N87260 X-0.367443 Y1.80482 Z4.81341

N87270 X-0.394171 Y1.80482 Z4.82875

N87280 X-0.446197 Y1.80482 Z4.85981

N87290 X-0.478184 Y1.80482 Z4.87942

N87300 X-0.545744 Y1.80482 Z4.92218

N87310 X-0.570676 Y1.80482 Z4.93786

N87320 X-0.624396 Y1.80482 Z4.97236

N87330 X-0.698236 Y1.80482 Z5.01922

N87340 X-0.850626 Y1.80482 Z5.11574

N87350 X-0.931994 Y1.80482 Z5.16522

N87360 X-0.980039 Y1.80482 Z5.19431

N87370 X-1.0946 Y1.80482 Z5.25885

N87380 X-1.14366 Y1.80482 Z5.2837

N87390 X-1.20645 Y1.80482 Z5.315

N87400 X-1.3017 Y1.80482 Z5.35682

N87410 X-1.31719 Y1.80482 Z5.36283

N87420 X-1.40811 Y1.80482 Z5.39538

N87430 X-1.45738 Y1.80482 Z5.41017

N87440 X-1.52528 Y1.80482 Z5.42941

N87450 X-1.61562 Y1.80482 Z5.45083

N87460 X-1.65653 Y1.80482 Z5.45979

N87470 X-1.69759 Y1.80482 Z5.46693

N87480 X-1.81084 Y1.80482 Z5.48704

N87490 X-1.87294 Y1.80482 Z5.49698

N87500 X-2.00619 Y1.80482 Z5.51428

N87510 X-2.05644 Y1.80482 Z5.52095

N87520 X-2.20238 Y1.80482 Z5.53729

N87530 X-2.23918 Y1.80482 Z5.54188

N87540 X-2.40929 Y1.80482 Z5.56175

N87550 X-2.44125 Y1.80482 Z5.56621

N87560 X-2.60034 Y1.80482 Z5.58774

N87570 X-2.63818 Y1.80482 Z5.59378

N87580 X-2.76203 Y1.80482 Z5.61367

N87590 X-2.79729 Y1.80482 Z5.62013

N87600 X-2.87338 Y1.80482 Z5.63465

N87610 X-2.91026 Y1.80482 Z5.64275

N87620 X-2.96039 Y1.80482 Z5.65438

N87630 X-3.04366 Y1.80482 Z5.67552

N87640 X-3.06808 Y1.80482 Z5.68158

N87650 X-3.08742 Y1.80482 Z5.68681

N87660 X-3.1899 Y1.80482 Z5.71521

N87670 X-3.29849 Y1.80482 Z5.74701

N87680 X-3.32965 Y1.80482 Z5.75649

N87690 X-3.38218 Y1.80482 Z5.77305

N87700 X-3.48251 Y1.80482 Z5.80382

N87710 X-3.56176 Y1.80482 Z5.82856

N87720 X-3.62184 Y1.80482 Z5.84804

N87730 X-3.74282 Y1.80482 Z5.88575

N87740 X-3.76323 Y1.80482 Z5.89245

N87750 X-3.90843 Y1.80482 Z5.93525

N87760 X-3.9216 Y1.80482 Z5.93914

N87770 X-4.07364 Y1.80482 Z5.98013

N87780 X-4.08988 Y1.80482 Z5.98455

N87790 X-4.11352 Y1.80482 Z5.99038

N87800 X-4.23352 Y1.80482 Z6.01973

N87810 X-4.30888 Y1.80482 Z6.03606

N87820 X-4.35753 Y1.80482 Z6.04614

N87830 X-4.46171 Y1.80482 Z6.06352

N87840 X-4.47674 Y1.80482 Z6.06575

N87850 X-4.53987 Y1.80482 Z6.07253

N87860 X-4.59878 Y1.80482 Z6.07886

N87870 X-4.69344 Y1.80482 Z6.0844

N87880 X-4.72683 Y1.80482 Z6.08569

N87890 X-4.78656 Y1.80482 Z6.08625

N87900 X-4.84699 Y1.80482 Z6.08566

N87910 X-4.87999 Y1.80482 Z6.08381

N87920 X-4.9524 Y1.80482 Z6.07832

N87930 X-4.99865 Y1.80482 Z6.07223

N87940 X-5.05915 Y1.80482 Z6.06387

N87950 X-5.15829 Y1.80482 Z6.04614

N87960 X-5.16906 Y1.80482 Z6.04362

N87970 X-5.26037 Y1.80482 Z6.02213

N87980 X-5.29471 Y1.80482 Z6.0123

N87990 X-5.36328 Y1.80482 Z5.9931

N88000 X-5.42622 Y1.80482 Z5.97249

N88010 X-5.46642 Y1.80482 Z5.95984

N88020 X-5.51394 Y1.80482 Z5.94233

N88030 X-5.57009 Y1.80482 Z5.92046

N88040 X-5.63869 Y1.80482 Z5.89069

N88050 X-5.68223 Y1.80482 Z5.87173

N88060 X-5.70727 Y1.80482 Z5.85998

N88070 X-5.79461 Y1.80482 Z5.81492

N88080 X-5.82533 Y1.80482 Z5.7972

N88090 X-5.89319 Y1.80482 Z5.75452

N88100 X-5.9406 Y1.80482 Z5.72105

N88110 X-5.98303 Y1.80482 Z5.68975

N88120 X-6.05228 Y1.80482 Z5.63216

N88130 X-6.07505 Y1.80482 Z5.61281

N88140 X-6.17604 Y1.80482 Z5.51492

N88150 X-6.18442 Y1.80482 Z5.50587

N88160 X-6.26103 Y1.80482 Z5.42276

N88170 X-6.27374 Y1.80482 Z5.40813

N88180 X-6.32787 Y1.80482 Z5.33994

N88190 X-6.33957 Y1.80482 Z5.32419

N88200 X-6.38757 Y1.80482 Z5.2549

N88210 X-6.39408 Y1.80482 Z5.2451

N88220 X-6.44164 Y1.80482 Z5.16667

N88230 X-6.4694 Y1.80482 Z5.11498

N88240 X-6.48629 Y1.80482 Z5.08271

N88250 X-6.55705 Y1.80482 Z4.92165

N88260 X-6.56136 Y1.80482 Z4.9108

N88270 X-6.56765 Y1.80482 Z4.89348

N88280 X-6.59461 Y1.80482 Z4.8175

N88290 X-6.61161 Y1.80482 Z4.76732

N88300 X-6.62643 Y1.80482 Z4.71917

N88310 X-6.64594 Y1.80482 Z4.65257

N88320 X-6.65349 Y1.80482 Z4.62618

N88330 X-6.66545 Y1.80482 Z4.58079

N88340 X-6.67961 Y1.80482 Z4.52505

N88350 X-6.68563 Y1.80482 Z4.50177

N88360 X-6.7121 Y1.80482 Z4.39516

N88370 X-6.73604 Y1.80482 Z4.31457

N88380 X-6.75438 Y1.80482 Z4.24944

N88390 X-6.74379 Y1.67638 Z4.22916

N88400 X-6.72324 Y1.67638 Z4.30217

N88410 X-6.69863 Y1.67638 Z4.386

N88420 X-6.68283 Y1.67638 Z4.44974

N88430 X-6.66371 Y1.67638 Z4.52349

N88440 X-6.64122 Y1.67638 Z4.61098

N88450 X-6.63552 Y1.67638 Z4.63209

N88460 X-6.63062 Y1.67638 Z4.64817

N88470 X-6.60514 Y1.67638 Z4.73165

N88480 X-6.57922 Y1.67638 Z4.80939

N88490 X-6.57381 Y1.67638 Z4.82392

N88500 X-6.56661 Y1.67638 Z4.84319

N88510 X-6.54058 Y1.67638 Z4.90999

N88520 X-6.52564 Y1.67638 Z4.94695

N88530 X-6.46902 Y1.67638 Z5.07201

N88540 X-6.45198 Y1.67638 Z5.10465

N88550 X-6.43009 Y1.67638 Z5.14537

N88560 X-6.38691 Y1.67638 Z5.21537

N88570 X-6.37128 Y1.67638 Z5.23885

N88580 X-6.33489 Y1.67638 Z5.29133

N88590 X-6.32273 Y1.67638 Z5.30784

N88600 X-6.28209 Y1.67638 Z5.36142

N88610 X-6.22827 Y1.67638 Z5.42497

N88620 X-6.21527 Y1.67638 Z5.43968

N88630 X-6.15925 Y1.67638 Z5.49825

N88640 X-6.10531 Y1.67638 Z5.55328

N88650 X-6.08568 Y1.67638 Z5.57227

N88660 X-6.01906 Y1.67638 Z5.63356

N88670 X-6.00549 Y1.67638 Z5.64483

N88680 X-5.93301 Y1.67638 Z5.70258

N88690 X-5.91232 Y1.67638 Z5.71709

N88700 X-5.84623 Y1.67638 Z5.76006

N88710 X-5.77911 Y1.67638 Z5.7984

N88720 X-5.75772 Y1.67638 Z5.80932

N88730 X-5.72433 Y1.67638 Z5.8255

N88740 X-5.67046 Y1.67638 Z5.85078

N88750 X-5.54038 Y1.67638 Z5.90361

N88760 X-5.48183 Y1.67638 Z5.92524

N88770 X-5.45093 Y1.67638 Z5.93482

N88780 X-5.3906 Y1.67638 Z5.95345

N88790 X-5.34357 Y1.67638 Z5.96633

N88800 X-5.28595 Y1.67638 Z5.98236

N88810 X-5.21544 Y1.67638 Z5.99926

N88820 X-5.17681 Y1.67638 Z6.00831

N88830 X-5.07848 Y1.67638 Z6.02668

N88840 X-5.06849 Y1.67638 Z6.02856

N88850 X-4.96968 Y1.67638 Z6.04175

N88860 X-4.92224 Y1.67638 Z6.04581

N88870 X-4.88565 Y1.67638 Z6.04861

N88880 X-4.83464 Y1.67638 Z6.05022

N88890 X-4.78411 Y1.67638 Z6.05052

N88900 X-4.75043 Y1.67638 Z6.04998

N88910 X-4.66022 Y1.67638 Z6.04575

N88920 X-4.56153 Y1.67638 Z6.0375

N88930 X-4.53097 Y1.67638 Z6.03408

N88940 X-4.453 Y1.67638 Z6.0233

N88950 X-4.39953 Y1.67638 Z6.01406

N88960 X-4.34026 Y1.67638 Z6.00225

N88970 X-4.27201 Y1.67638 Z5.98701

N88980 X-4.23868 Y1.67638 Z5.97884

N88990 X-4.17373 Y1.67638 Z5.96219

N89000 X-4.14148 Y1.67638 Z5.95425

N89010 X-4.03977 Y1.67638 Z5.92652

N89020 X-4.02416 Y1.67638 Z5.92231

N89030 X-4.00732 Y1.67638 Z5.91728

N89040 X-3.86419 Y1.67638 Z5.87505

N89050 X-3.83756 Y1.67638 Z5.86719

N89060 X-3.72364 Y1.67638 Z5.8298

N89070 X-3.66163 Y1.67638 Z5.80956

N89080 X-3.64198 Y1.67638 Z5.80257

N89090 X-3.4874 Y1.67638 Z5.7517

N89100 X-3.37531 Y1.67638 Z5.71466

N89110 X-3.33674 Y1.67638 Z5.7025

N89120 X-3.29196 Y1.67638 Z5.68887

N89130 X-3.21012 Y1.67638 Z5.66335

N89140 X-3.16624 Y1.67638 Z5.65051

N89150 X-3.09625 Y1.67638 Z5.6308

N89160 X-2.99886 Y1.67638 Z5.60518

N89170 X-2.98761 Y1.67638 Z5.60226

N89180 X-2.97576 Y1.67638 Z5.5995

N89190 X-2.89254 Y1.67638 Z5.58067

N89200 X-2.85079 Y1.67638 Z5.57269

N89210 X-2.76688 Y1.67638 Z5.5581

N89220 X-2.67993 Y1.67638 Z5.54415

N89230 X-2.57509 Y1.67638 Z5.52961

N89240 X-2.49241 Y1.67638 Z5.51842

N89250 X-2.3715 Y1.67638 Z5.5048

N89260 X-2.29142 Y1.67638 Z5.49545

N89270 X-2.16791 Y1.67638 Z5.48311

N89280 X-2.07242 Y1.67638 Z5.47242

N89290 X-1.97677 Y1.67638 Z5.46253

N89300 X-1.84943 Y1.67638 Z5.446

N89310 X-1.68686 Y1.67638 Z5.4218

N89320 X-1.63586 Y1.67638 Z5.41293

N89330 X-1.60057 Y1.67638 Z5.4052

N89340 X-1.50534 Y1.67638 Z5.38362

N89350 X-1.44036 Y1.67638 Z5.3645

N89360 X-1.38181 Y1.67638 Z5.34611

N89370 X-1.32381 Y1.67638 Z5.32363

N89380 X-1.27108 Y1.67638 Z5.30191

N89390 X-1.21433 Y1.67638 Z5.27582

N89400 X-1.17047 Y1.67638 Z5.25502

N89410 X-1.07235 Y1.67638 Z5.20567

N89420 X-0.951178 Y1.67638 Z5.13886

N89430 X-0.869846 Y1.67638 Z5.09041

N89440 X-0.825972 Y1.67638 Z5.06452

N89450 X-0.698615 Y1.67638 Z4.985

N89460 X-0.678155 Y1.67638 Z4.97202

N89470 X-0.580992 Y1.67638 Z4.91036

N89480 X-0.537692 Y1.67638 Z4.88307

N89490 X-0.487368 Y1.67638 Z4.85193

N89500 X-0.444479 Y1.67638 Z4.82632

N89510 X-0.41375 Y1.67638 Z4.80865

N89520 X-0.361723 Y1.67638 Z4.78123

N89530 X-0.321152 Y1.67638 Z4.76244

N89540 X-0.298532 Y1.67638 Z4.75332

N89550 X-0.289089 Y1.67638 Z4.74952

N89560 X-0.2788 Y1.67638 Z4.74604

N89570 X-0.268747 Y1.67638 Z4.75106

N89580 X-0.254551 Y1.67638 Z4.75983

N89590 X-0.234908 Y1.67638 Z4.77219

N89600 X-0.191285 Y1.67638 Z4.8039

N89610 X-0.174498 Y1.67638 Z4.81719

N89620 X-0.155913 Y1.67638 Z4.83271

N89630 X-0.130548 Y1.67638 Z4.8563

N89640 X-0.0977839 Y1.67638 Z4.89173

N89650 X-0.0727261 Y1.67638 Z4.92137

N89660 X-0.057972 Y1.67638 Z4.9391

N89670 X-0.0263031 Y1.67638 Z4.9788

N89680 X0.00655327 Y1.67638 Z5.0243

N89690 X0.0743126 Y1.67638 Z5.1198

N89700 X0.0946678 Y1.67638 Z5.14813

N89710 X0.159896 Y1.67638 Z5.2413

N89720 X0.220995 Y1.67638 Z5.32752

N89730 X0.240746 Y1.67638 Z5.35613

N89740 X0.262101 Y1.67638 Z5.38653

N89750 X0.331083 Y1.67638 Z5.48388

N89760 X0.387311 Y1.67638 Z5.56029

N89770 X0.400296 Y1.67638 Z5.57767

N89780 X0.446299 Y1.67638 Z5.63887

N89790 X0.494851 Y1.67638 Z5.69954

N89800 X0.508719 Y1.67638 Z5.71687

N89810 X0.523804 Y1.67638 Z5.73415

N89820 X0.574196 Y1.67638 Z5.79278

N89830 X0.625977 Y1.67638 Z5.84843

N89840 X0.65239 Y1.67638 Z5.87653

N89850 X0.701064 Y1.67638 Z5.92553

N89860 X0.741678 Y1.67638 Z5.96657

N89870 X0.841694 Y1.67638 Z6.06168

N89880 X0.915709 Y1.67638 Z6.12847

N89890 X0.950382 Y1.67638 Z6.15877

N89900 X1.05929 Y1.67638 Z6.24892

N89910 X1.15736 Y1.67638 Z6.32206

N89920 X1.19258 Y1.67638 Z6.34718

N89930 X1.27132 Y1.67638 Z6.40179

N89940 X1.29489 Y1.67638 Z6.417

N89950 X1.38981 Y1.67638 Z6.47783

N89960 X1.40392 Y1.67638 Z6.48618

N89970 X1.49663 Y1.67638 Z6.5398

N89980 X1.53276 Y1.67638 Z6.55988

N89990 X1.61032 Y1.67638 Z6.60114

N90000 X1.71008 Y1.67638 Z6.65179

N90010 X1.73454 Y1.67638 Z6.66385

N90020 X1.78646 Y1.67638 Z6.68817

N90030 X1.87076 Y1.67638 Z6.7271

N90040 X1.92338 Y1.67638 Z6.7501

N90050 X2.01529 Y1.67638 Z6.78945

N90060 X2.11501 Y1.67638 Z6.83006

N90070 X2.14315 Y1.67638 Z6.84133

N90080 X2.17067 Y1.67638 Z6.85155

N90090 X2.25291 Y1.67638 Z6.88099

N90100 X2.33386 Y1.67638 Z6.90753

N90110 X2.35018 Y1.67638 Z6.91203

N90120 X2.43364 Y1.67638 Z6.93338

N90130 X2.45144 Y1.67638 Z6.93764

N90140 X2.54639 Y1.67638 Z6.95793

N90150 X2.57613 Y1.67638 Z6.96386

N90160 X2.66968 Y1.67638 Z6.97997

N90170 X2.69064 Y1.67638 Z6.98282

N90180 X2.80683 Y1.67638 Z6.99712

N90190 X2.92985 Y1.67638 Z7.00713

N90200 X2.94422 Y1.67638 Z7.00821

N90210 X3.0561 Y1.67638 Z7.01312

N90220 X3.0781 Y1.67638 Z7.01335

N90230 X3.16767 Y1.67638 Z7.01305

N90240 X3.19248 Y1.67638 Z7.01273

N90250 X3.27916 Y1.67638 Z7.01092

N90260 X3.30024 Y1.67638 Z7.01023

N90270 X3.39478 Y1.67638 Z7.00399

N90280 X3.47064 Y1.67638 Z6.99584

N90290 X3.49265 Y1.67638 Z6.99336

N90300 X3.5567 Y1.67638 Z6.98446

N90310 X3.60696 Y1.67638 Z6.97717

N90320 X3.62588 Y1.67638 Z6.97418

N90330 X3.7463 Y1.67638 Z6.95415

N90340 X3.80494 Y1.67638 Z6.94366

N90350 X3.90847 Y1.67638 Z6.92436

N90360 X4.00149 Y1.67638 Z6.9057

N90370 X4.08015 Y1.67638 Z6.88939

N90380 X4.22771 Y1.67638 Z6.85644

N90390 X4.25442 Y1.67638 Z6.85028

N90400 X4.27461 Y1.67638 Z6.84524

N90410 X4.42237 Y1.67638 Z6.80778

N90420 X4.44925 Y1.67638 Z6.79977

N90430 X4.54288 Y1.67638 Z6.77171

N90440 X4.58022 Y1.67638 Z6.75977

N90450 X4.64108 Y1.67638 Z6.73817

N90460 X4.71935 Y1.67638 Z6.70927

N90470 X4.73473 Y1.67638 Z6.70294

N90480 X4.77332 Y1.67638 Z6.68611

N90490 X4.82771 Y1.67638 Z6.66218

N90500 X4.84663 Y1.67638 Z6.65322

N90510 X4.92089 Y1.67638 Z6.61503

N90520 X4.9586 Y1.67638 Z6.59456

N90530 X5.01256 Y1.67638 Z6.56284

N90540 X5.06285 Y1.67638 Z6.53221

N90550 X5.10312 Y1.67638 Z6.50558

N90560 X5.16087 Y1.67638 Z6.46638

N90570 X5.19156 Y1.67638 Z6.44391

N90580 X5.25055 Y1.67638 Z6.39978

N90590 X5.27503 Y1.67638 Z6.38032

N90600 X5.33224 Y1.67638 Z6.33365

N90610 X5.35016 Y1.67638 Z6.3183

N90620 X5.40852 Y1.67638 Z6.26511

N90630 X5.41894 Y1.67638 Z6.25561

N90640 X5.48238 Y1.67638 Z6.19031

N90650 X5.51867 Y1.67638 Z6.15195

N90660 X5.57283 Y1.67638 Z6.09074

N90670 X5.60536 Y1.67638 Z6.05294

N90680 X5.68148 Y1.67638 Z5.96054

N90690 X5.69157 Y1.67638 Z5.94805

N90700 X5.70454 Y1.67638 Z5.93127

N90710 X5.78463 Y1.67638 Z5.82726

N90720 X5.81529 Y1.67638 Z5.78611

N90730 X5.89787 Y1.67638 Z5.67335

N90740 X5.92668 Y1.67638 Z5.63333

N90750 X6.01891 Y1.67638 Z5.50316

N90760 X6.03236 Y1.67638 Z5.48387

N90770 X6.09923 Y1.67638 Z5.38531

N90780 X6.12749 Y1.67638 Z5.3436

N90790 X6.21662 Y1.67638 Z5.20473

N90800 X6.23952 Y1.67638 Z5.16844

N90810 X6.30346 Y1.67638 Z5.06573

N90820 X6.31732 Y1.67638 Z5.04317

N90830 X6.3228 Y1.67638 Z5.03406

N90840 X6.39755 Y1.67638 Z4.90278

N90850 X6.43812 Y1.67638 Z4.8268

N90860 X6.46047 Y1.67638 Z4.78246

N90870 X6.49624 Y1.67638 Z4.71136

N90880 X6.5217 Y1.67638 Z4.65842

N90890 X6.55319 Y1.67638 Z4.59334

N90900 X6.57925 Y1.67638 Z4.53625

N90910 X6.61061 Y1.67638 Z4.46413

N90920 X6.63317 Y1.67638 Z4.40553

N90930 X6.66696 Y1.67638 Z4.30774

N90940 X6.68253 Y1.67638 Z4.25699

N90950 X6.71915 Y1.67638 Z4.12213

N90960 X6.72448 Y1.67638 Z4.10025

N90970 X6.74606 Y1.67638 Z4.02853

N90980 X6.76596 Y1.67638 Z3.9602

N90990 X6.76011 Y1.54727 Z3.97678

N91000 X6.73175 Y1.54727 Z4.07819

N91010 X6.70274 Y1.54727 Z4.19189

N91020 X6.69222 Y1.54727 Z4.23089

N91030 X6.66141 Y1.54727 Z4.32912

N91040 X6.63858 Y1.54727 Z4.39749

N91050 X6.62039 Y1.54727 Z4.44456

N91060 X6.59307 Y1.54727 Z4.51031

N91070 X6.56798 Y1.54727 Z4.56557

N91080 X6.54071 Y1.54727 Z4.62706

N91090 X6.5116 Y1.54727 Z4.68784

N91100 X6.48404 Y1.54727 Z4.74675

N91110 X6.45273 Y1.54727 Z4.80903

N91120 X6.42425 Y1.54727 Z4.86414

N91130 X6.33433 Y1.54727 Z5.02236

N91140 X6.3155 Y1.54727 Z5.05302

N91150 X6.25612 Y1.54727 Z5.14831

N91160 X6.20355 Y1.54727 Z5.23194

N91170 X6.17101 Y1.54727 Z5.28246

N91180 X6.12896 Y1.54727 Z5.34637

N91190 X6.09572 Y1.54727 Z5.396

N91200 X6.01794 Y1.54727 Z5.50761

N91210 X5.99475 Y1.54727 Z5.54043

N91220 X5.9057 Y1.54727 Z5.66386

N91230 X5.80145 Y1.54727 Z5.80373

N91240 X5.7438 Y1.54727 Z5.87777

N91250 X5.71241 Y1.54727 Z5.91733

N91260 X5.6633 Y1.54727 Z5.97803

N91270 X5.62311 Y1.54727 Z6.02684

N91280 X5.60221 Y1.54727 Z6.05146

N91290 X5.54286 Y1.54727 Z6.11982

N91300 X5.50813 Y1.54727 Z6.1578

N91310 X5.46531 Y1.54727 Z6.20323

N91320 X5.43486 Y1.54727 Z6.23304

N91330 X5.38019 Y1.54727 Z6.28504

N91340 X5.36581 Y1.54727 Z6.29756

N91350 X5.29167 Y1.54727 Z6.36032

N91360 X5.27267 Y1.54727 Z6.37493

N91370 X5.20591 Y1.54727 Z6.42602

N91380 X5.12804 Y1.54727 Z6.48027

N91390 X5.11231 Y1.54727 Z6.49099

N91400 X5.04305 Y1.54727 Z6.53446

N91410 X5.0136 Y1.54727 Z6.55251

N91420 X4.95758 Y1.54727 Z6.5843

N91430 X4.91305 Y1.54727 Z6.6087

N91440 X4.87083 Y1.54727 Z6.63038

N91450 X4.80391 Y1.54727 Z6.66247

N91460 X4.77987 Y1.54727 Z6.6731

N91470 X4.72349 Y1.54727 Z6.69555

N91480 X4.68587 Y1.54727 Z6.71038

N91490 X4.66886 Y1.54727 Z6.71677

N91500 X4.58314 Y1.54727 Z6.74431

N91510 X4.53287 Y1.54727 Z6.76081

N91520 X4.44345 Y1.54727 Z6.78553

N91530 X4.41291 Y1.54727 Z6.79362

N91540 X4.38593 Y1.54727 Z6.80049

N91550 X4.25211 Y1.54727 Z6.8325

N91560 X4.16489 Y1.54727 Z6.8524

N91570 X4.06283 Y1.54727 Z6.87489

N91580 X3.9517 Y1.54727 Z6.89741

N91590 X3.87667 Y1.54727 Z6.91205

N91600 X3.75573 Y1.54727 Z6.93391

N91610 X3.70962 Y1.54727 Z6.94179

N91620 X3.5866 Y1.54727 Z6.96162

N91630 X3.5751 Y1.54727 Z6.96331

N91640 X3.54716 Y1.54727 Z6.96705

N91650 X3.46445 Y1.54727 Z6.97772

N91660 X3.44172 Y1.54727 Z6.98042

N91670 X3.36223 Y1.54727 Z6.98756

N91680 X3.33451 Y1.54727 Z6.98904

N91690 X3.23334 Y1.54727 Z6.99376

N91700 X3.22199 Y1.54727 Z6.99399

N91710 X3.10331 Y1.54727 Z6.99548

N91720 X3.01983 Y1.54727 Z6.99372

N91730 X2.97043 Y1.54727 Z6.9926

N91740 X2.84035 Y1.54727 Z6.98455

N91750 X2.82676 Y1.54727 Z6.98326

N91760 X2.71971 Y1.54727 Z6.97229

N91770 X2.66901 Y1.54727 Z6.96472

N91780 X2.60569 Y1.54727 Z6.95489

N91790 X2.56887 Y1.54727 Z6.94861

N91800 X2.46014 Y1.54727 Z6.92702

N91810 X2.42319 Y1.54727 Z6.91899

N91820 X2.34132 Y1.54727 Z6.89901

N91830 X2.3135 Y1.54727 Z6.89215

N91840 X2.29308 Y1.54727 Z6.88598

N91850 X2.21133 Y1.54727 Z6.86036

N91860 X2.10508 Y1.54727 Z6.82248

N91870 X2.09562 Y1.54727 Z6.81878

N91880 X1.97501 Y1.54727 Z6.7702

N91890 X1.93553 Y1.54727 Z6.75387

N91900 X1.83335 Y1.54727 Z6.70949

N91910 X1.79089 Y1.54727 Z6.69093

N91920 X1.77049 Y1.54727 Z6.68151

N91930 X1.66023 Y1.54727 Z6.62912

N91940 X1.56398 Y1.54727 Z6.58111

N91950 X1.53863 Y1.54727 Z6.56794

N91960 X1.49424 Y1.54727 Z6.54373

N91970 X1.42769 Y1.54727 Z6.50674

N91980 X1.40122 Y1.54727 Z6.49143

N91990 X1.32388 Y1.54727 Z6.44395

N92000 X1.26937 Y1.54727 Z6.40947

N92010 X1.22332 Y1.54727 Z6.37857

N92020 X1.14279 Y1.54727 Z6.32305

N92030 X1.1164 Y1.54727 Z6.30425

N92040 X1.07982 Y1.54727 Z6.2774

N92050 X1.01601 Y1.54727 Z6.22956

N92060 X0.928944 Y1.54727 Z6.15976

N92070 X0.920423 Y1.54727 Z6.1527

N92080 X0.89674 Y1.54727 Z6.13201

N92090 X0.817687 Y1.54727 Z6.06277

N92100 X0.789749 Y1.54727 Z6.03702

N92110 X0.727112 Y1.54727 Z5.97799

N92120 X0.663809 Y1.54727 Z5.91516

N92130 X0.638817 Y1.54727 Z5.89

N92140 X0.606267 Y1.54727 Z5.85536

N92150 X0.558099 Y1.54727 Z5.80444

N92160 X0.521663 Y1.54727 Z5.76299

N92170 X0.490244 Y1.54727 Z5.72701

N92180 X0.467418 Y1.54727 Z5.69849

N92190 X0.432576 Y1.54727 Z5.65584

N92200 X0.38841 Y1.54727 Z5.5978

N92210 X0.374741 Y1.54727 Z5.57987

N92220 X0.352219 Y1.54727 Z5.54883

N92230 X0.298633 Y1.54727 Z5.47634

N92240 X0.267534 Y1.54727 Z5.43239

N92250 X0.187337 Y1.54727 Z5.31639

N92260 X0.181558 Y1.54727 Z5.30796

N92270 X0.175078 Y1.54727 Z5.29846

N92280 X0.111013 Y1.54727 Z5.20635

N92290 X0.0644514 Y1.54727 Z5.1392

N92300 X-0.0700013 Y1.54727 Z4.95042

N92310 X-0.0940169 Y1.54727 Z4.91854

N92320 X-0.112227 Y1.54727 Z4.89645

N92330 X-0.12601 Y1.54727 Z4.88032

N92340 X-0.14362 Y1.54727 Z4.8629

N92350 X-0.165594 Y1.54727 Z4.84336

N92360 X-0.175816 Y1.54727 Z4.83478

N92370 X-0.207827 Y1.54727 Z4.80931

N92380 X-0.219712 Y1.54727 Z4.80035

N92390 X-0.264299 Y1.54727 Z4.76911

N92400 X-0.284107 Y1.54727 Z4.75717

N92410 X-0.297132 Y1.54727 Z4.74945

N92420 X-0.306328 Y1.54727 Z4.74519

N92430 X-0.317504 Y1.54727 Z4.74155

N92440 X-0.327532 Y1.54727 Z4.7446

N92450 X-0.337171 Y1.54727 Z4.74762

N92460 X-0.367691 Y1.54727 Z4.75917

N92470 X-0.396005 Y1.54727 Z4.77168

N92480 X-0.407442 Y1.54727 Z4.77685

N92490 X-0.451765 Y1.54727 Z4.79904

N92500 X-0.465078 Y1.54727 Z4.80635

N92510 X-0.500927 Y1.54727 Z4.82673

N92520 X-0.529018 Y1.54727 Z4.84327

N92530 X-0.577098 Y1.54727 Z4.87294

N92540 X-0.631404 Y1.54727 Z4.90619

N92550 X-0.790179 Y1.54727 Z5.00407

N92560 X-0.799798 Y1.54727 Z5.01003

N92570 X-0.917803 Y1.54727 Z5.07769

N92580 X-1.00581 Y1.54727 Z5.12496

N92590 X-1.03475 Y1.54727 Z5.14051

N92600 X-1.06481 Y1.54727 Z5.15585

N92610 X-1.14502 Y1.54727 Z5.19558

N92620 X-1.2197 Y1.54727 Z5.2317

N92630 X-1.23339 Y1.54727 Z5.23814

N92640 X-1.31882 Y1.54727 Z5.27474

N92650 X-1.34111 Y1.54727 Z5.28394

N92660 X-1.4262 Y1.54727 Z5.31378

N92670 X-1.47699 Y1.54727 Z5.3298

N92680 X-1.545 Y1.54727 Z5.34611

N92690 X-1.71882 Y1.54727 Z5.37983

N92700 X-1.76332 Y1.54727 Z5.38693

N92710 X-1.87201 Y1.54727 Z5.40081

N92720 X-1.91784 Y1.54727 Z5.40651

N92730 X-1.93263 Y1.54727 Z5.40803

N92740 X-2.107 Y1.54727 Z5.42609

N92750 X-2.16248 Y1.54727 Z5.43122

N92760 X-2.38136 Y1.54727 Z5.4528

N92770 X-2.49538 Y1.54727 Z5.46564

N92780 X-2.55589 Y1.54727 Z5.4727

N92790 X-2.72458 Y1.54727 Z5.49579

N92800 X-2.74709 Y1.54727 Z5.49887

N92810 X-2.7679 Y1.54727 Z5.50225

N92820 X-2.89872 Y1.54727 Z5.52514

N92830 X-2.96123 Y1.54727 Z5.53925

N92840 X-3.01555 Y1.54727 Z5.55192

N92850 X-3.07505 Y1.54727 Z5.56732

N92860 X-3.13413 Y1.54727 Z5.58346

N92870 X-3.21122 Y1.54727 Z5.60588

N92880 X-3.24098 Y1.54727 Z5.61533

N92890 X-3.31288 Y1.54727 Z5.63849

N92900 X-3.4756 Y1.54727 Z5.69415

N92910 X-3.49232 Y1.54727 Z5.69966

N92920 X-3.68859 Y1.54727 Z5.76948

N92930 X-3.74297 Y1.54727 Z5.78724

N92940 X-3.86299 Y1.54727 Z5.83005

N92950 X-3.99103 Y1.54727 Z5.8694

N92960 X-4.08252 Y1.54727 Z5.89632

N92970 X-4.14423 Y1.54727 Z5.91326

N92980 X-4.16139 Y1.54727 Z5.91809

N92990 X-4.17475 Y1.54727 Z5.92148

N93000 X-4.24135 Y1.54727 Z5.93954

N93010 X-4.29625 Y1.54727 Z5.9529

N93020 X-4.33471 Y1.54727 Z5.96253

N93030 X-4.42098 Y1.54727 Z5.9799

N93040 X-4.4591 Y1.54727 Z5.98652

N93050 X-4.53072 Y1.54727 Z5.99699

N93060 X-4.58765 Y1.54727 Z6.00352

N93070 X-4.63065 Y1.54727 Z6.0075

N93080 X-4.72434 Y1.54727 Z6.01313

N93090 X-4.80434 Y1.54727 Z6.01514

N93100 X-4.84103 Y1.54727 Z6.01431

N93110 X-4.88315 Y1.54727 Z6.0131

N93120 X-4.96014 Y1.54727 Z6.00711

N93130 X-4.97685 Y1.54727 Z6.00568

N93140 X-4.99219 Y1.54727 Z6.00363

N93150 X-5.08912 Y1.54727 Z5.99036

N93160 X-5.14622 Y1.54727 Z5.97965

N93170 X-5.20238 Y1.54727 Z5.96832

N93180 X-5.28287 Y1.54727 Z5.94902

N93190 X-5.32047 Y1.54727 Z5.93944

N93200 X-5.39637 Y1.54727 Z5.91865

N93210 X-5.42813 Y1.54727 Z5.90997

N93220 X-5.47747 Y1.54727 Z5.89467

N93230 X-5.5117 Y1.54727 Z5.88389

N93240 X-5.57353 Y1.54727 Z5.86151

N93250 X-5.58424 Y1.54727 Z5.85745

N93260 X-5.59425 Y1.54727 Z5.8534

N93270 X-5.6525 Y1.54727 Z5.82797

N93280 X-5.72059 Y1.54727 Z5.7976

N93290 X-5.7383 Y1.54727 Z5.78903

N93300 X-5.75844 Y1.54727 Z5.77874

N93310 X-5.82399 Y1.54727 Z5.74415

N93320 X-5.87392 Y1.54727 Z5.71431

N93330 X-5.89306 Y1.54727 Z5.70181

N93340 X-5.95243 Y1.54727 Z5.65699

N93350 X-5.96542 Y1.54727 Z5.64669

N93360 X-6.01441 Y1.54727 Z5.60388

N93370 X-6.03078 Y1.54727 Z5.58927

N93380 X-6.0827 Y1.54727 Z5.53858

N93390 X-6.1123 Y1.54727 Z5.50914

N93400 X-6.14258 Y1.54727 Z5.47813

N93410 X-6.18796 Y1.54727 Z5.42964

N93420 X-6.20341 Y1.54727 Z5.41278

N93430 X-6.21924 Y1.54727 Z5.39423

N93440 X-6.2645 Y1.54727 Z5.3396

N93450 X-6.30895 Y1.54727 Z5.28021

N93460 X-6.32534 Y1.54727 Z5.25796

N93470 X-6.33289 Y1.54727 Z5.24706

N93480 X-6.39096 Y1.54727 Z5.15987

N93490 X-6.42566 Y1.54727 Z5.09924

N93500 X-6.44099 Y1.54727 Z5.07239

N93510 X-6.48069 Y1.54727 Z4.9901

N93520 X-6.48927 Y1.54727 Z4.97122

N93530 X-6.5155 Y1.54727 Z4.90892

N93540 X-6.53653 Y1.54727 Z4.85854

N93550 X-6.55151 Y1.54727 Z4.81846

N93560 X-6.568 Y1.54727 Z4.77419

N93570 X-6.58714 Y1.54727 Z4.72147

N93580 X-6.61052 Y1.54727 Z4.64835

N93590 X-6.61704 Y1.54727 Z4.62626

N93600 X-6.63859 Y1.54727 Z4.54387

N93610 X-6.67308 Y1.54727 Z4.41218

N93620 X-6.68013 Y1.54727 Z4.38371

N93630 X-6.68874 Y1.54727 Z4.3544

N93640 X-6.72989 Y1.54727 Z4.20778

N93650 X-6.71233 Y1.41775 Z4.18373

N93660 X-6.68424 Y1.41775 Z4.28502

N93670 X-6.67044 Y1.41775 Z4.33362

N93680 X-6.64143 Y1.41775 Z4.4445

N93690 X-6.60493 Y1.41775 Z4.57996

N93700 X-6.57755 Y1.41775 Z4.67289

N93710 X-6.56698 Y1.41775 Z4.70412

N93720 X-6.53691 Y1.41775 Z4.78516

N93730 X-6.52437 Y1.41775 Z4.81597

N93740 X-6.49002 Y1.41775 Z4.90151

N93750 X-6.46168 Y1.41775 Z4.96491

N93760 X-6.44956 Y1.41775 Z4.99142

N93770 X-6.39759 Y1.41775 Z5.09072

N93780 X-6.3549 Y1.41775 Z5.16019

N93790 X-6.33461 Y1.41775 Z5.19057

N93800 X-6.30173 Y1.41775 Z5.23868

N93810 X-6.24339 Y1.41775 Z5.31826

N93820 X-6.21778 Y1.41775 Z5.3493

N93830 X-6.18271 Y1.41775 Z5.39099

N93840 X-6.15097 Y1.41775 Z5.42579

N93850 X-6.1241 Y1.41775 Z5.45451

N93860 X-6.10665 Y1.41775 Z5.4715

N93870 X-6.06601 Y1.41775 Z5.51137

N93880 X-6.02318 Y1.41775 Z5.55227

N93890 X-6.01191 Y1.41775 Z5.56283

N93900 X-5.99908 Y1.41775 Z5.57487

N93910 X-5.95797 Y1.41775 Z5.61036

N93920 X-5.9201 Y1.41775 Z5.64037

N93930 X-5.88248 Y1.41775 Z5.66828

N93940 X-5.84123 Y1.41775 Z5.69497

N93950 X-5.78577 Y1.41775 Z5.72857

N93960 X-5.75854 Y1.41775 Z5.74255

N93970 X-5.69476 Y1.41775 Z5.77344

N93980 X-5.65243 Y1.41775 Z5.79332

N93990 X-5.6157 Y1.41775 Z5.80849

N94000 X-5.54068 Y1.41775 Z5.83888

N94010 X-5.48738 Y1.41775 Z5.85714

N94020 X-5.46661 Y1.41775 Z5.86364

N94030 X-5.37594 Y1.41775 Z5.88987

N94040 X-5.36548 Y1.41775 Z5.89289

N94050 X-5.2333 Y1.41775 Z5.92665

N94060 X-5.11228 Y1.41775 Z5.95147

N94070 X-5.0835 Y1.41775 Z5.95624

N94080 X-5.00249 Y1.41775 Z5.96778

N94090 X-4.95016 Y1.41775 Z5.97316

N94100 X-4.89609 Y1.41775 Z5.977

N94110 X-4.86335 Y1.41775 Z5.97876

N94120 X-4.79007 Y1.41775 Z5.97984

N94130 X-4.70562 Y1.41775 Z5.97806

N94140 X-4.64282 Y1.41775 Z5.97335

N94150 X-4.60359 Y1.41775 Z5.97015

N94160 X-4.50831 Y1.41775 Z5.95809

N94170 X-4.45908 Y1.41775 Z5.9494

N94180 X-4.37957 Y1.41775 Z5.9356

N94190 X-4.36332 Y1.41775 Z5.93233

N94200 X-4.32749 Y1.41775 Z5.92336

N94210 X-4.25047 Y1.41775 Z5.90547

N94220 X-4.18483 Y1.41775 Z5.88724

N94230 X-4.15663 Y1.41775 Z5.87988

N94240 X-4.13295 Y1.41775 Z5.87295

N94250 X-4.06671 Y1.41775 Z5.85386

N94260 X-3.99684 Y1.41775 Z5.83186

N94270 X-3.98432 Y1.41775 Z5.82773

N94280 X-3.88315 Y1.41775 Z5.7947

N94290 X-3.82898 Y1.41775 Z5.7736

N94300 X-3.71337 Y1.41775 Z5.73258

N94310 X-3.59819 Y1.41775 Z5.68726

N94320 X-3.51662 Y1.41775 Z5.65821

N94330 X-3.39493 Y1.41775 Z5.61283

N94340 X-3.31938 Y1.41775 Z5.58638

N94350 X-3.26469 Y1.41775 Z5.56718

N94360 X-3.18804 Y1.41775 Z5.54273

N94370 X-3.16062 Y1.41775 Z5.53487

N94380 X-3.06345 Y1.41775 Z5.50876

N94390 X-2.91177 Y1.41775 Z5.47235

N94400 X-2.90183 Y1.41775 Z5.47025

N94410 X-2.8795 Y1.41775 Z5.4664

N94420 X-2.76126 Y1.41775 Z5.44717

N94430 X-2.61179 Y1.41775 Z5.42672

N94440 X-2.59777 Y1.41775 Z5.42494

N94450 X-2.43153 Y1.41775 Z5.40648

N94460 X-2.27609 Y1.41775 Z5.39132

N94470 X-2.25598 Y1.41775 Z5.38943

N94480 X-2.19697 Y1.41775 Z5.38391

N94490 X-2.06386 Y1.41775 Z5.37162

N94500 X-2.03238 Y1.41775 Z5.36836

N94510 X-1.89971 Y1.41775 Z5.35471

N94520 X-1.82542 Y1.41775 Z5.34548

N94530 X-1.76023 Y1.41775 Z5.33646

N94540 X-1.64193 Y1.41775 Z5.3167

N94550 X-1.62324 Y1.41775 Z5.31313

N94560 X-1.59183 Y1.41775 Z5.30632

N94570 X-1.50141 Y1.41775 Z5.28426

N94580 X-1.40558 Y1.41775 Z5.25429

N94590 X-1.38836 Y1.41775 Z5.2482

N94600 X-1.32845 Y1.41775 Z5.22419

N94610 X-1.26533 Y1.41775 Z5.19891

N94620 X-1.2431 Y1.41775 Z5.18925

N94630 X-1.13316 Y1.41775 Z5.1386

N94640 X-1.00223 Y1.41775 Z5.07469

N94650 X-0.909272 Y1.41775 Z5.02673

N94660 X-0.713268 Y1.41775 Z4.91701

N94670 X-0.650219 Y1.41775 Z4.8808

N94680 X-0.597984 Y1.41775 Z4.84895

N94690 X-0.560269 Y1.41775 Z4.82762

N94700 X-0.534089 Y1.41775 Z4.81215

N94710 X-0.504849 Y1.41775 Z4.79665

N94720 X-0.493468 Y1.41775 Z4.79054

N94730 X-0.482251 Y1.41775 Z4.78547

N94740 X-0.454739 Y1.41775 Z4.77213

N94750 X-0.438034 Y1.41775 Z4.76514

N94760 X-0.418747 Y1.41775 Z4.75722

N94770 X-0.390222 Y1.41775 Z4.74746

N94780 X-0.371244 Y1.41775 Z4.7421

N94790 X-0.359792 Y1.41775 Z4.74171

N94800 X-0.340723 Y1.41775 Z4.74907

N94810 X-0.319902 Y1.41775 Z4.76024

N94820 X-0.284555 Y1.41775 Z4.78383

N94830 X-0.265685 Y1.41775 Z4.79831

N94840 X-0.235004 Y1.41775 Z4.82185

N94850 X-0.193296 Y1.41775 Z4.85719

N94860 X-0.157202 Y1.41775 Z4.89183

N94870 X-0.139022 Y1.41775 Z4.91037

N94880 X-0.129938 Y1.41775 Z4.92035

N94890 X-0.109895 Y1.41775 Z4.94488

N94900 X0.0198471 Y1.41775 Z5.11893

N94910 X0.0904259 Y1.41775 Z5.22089

N94920 X0.106859 Y1.41775 Z5.24466

N94930 X0.152199 Y1.41775 Z5.31103

N94940 X0.182954 Y1.41775 Z5.35612

N94950 X0.223642 Y1.41775 Z5.41429

N94960 X0.280289 Y1.41775 Z5.49327

N94970 X0.298309 Y1.41775 Z5.51768

N94980 X0.325119 Y1.41775 Z5.55268

N94990 X0.3659 Y1.41775 Z5.60726

N95000 X0.386327 Y1.41775 Z5.63357

N95010 X0.424742 Y1.41775 Z5.68141

N95020 X0.454465 Y1.41775 Z5.71794

N95030 X0.513827 Y1.41775 Z5.78456

N95040 X0.536432 Y1.41775 Z5.81027

N95050 X0.563338 Y1.41775 Z5.83872

N95060 X0.637636 Y1.41775 Z5.91478

N95070 X0.677115 Y1.41775 Z5.95398

N95080 X0.771138 Y1.41775 Z6.04163

N95090 X0.86525 Y1.41775 Z6.1229

N95100 X0.913395 Y1.41775 Z6.16178

N95110 X0.958801 Y1.41775 Z6.19833

N95120 X1.00138 Y1.41775 Z6.23041

N95130 X1.04737 Y1.41775 Z6.26416

N95140 X1.13936 Y1.41775 Z6.32972

N95150 X1.15044 Y1.41775 Z6.33736

N95160 X1.19399 Y1.41775 Z6.36658

N95170 X1.25354 Y1.41775 Z6.4061

N95180 X1.27239 Y1.41775 Z6.4181

N95190 X1.35872 Y1.41775 Z6.47026

N95200 X1.42426 Y1.41775 Z6.50797

N95210 X1.46982 Y1.41775 Z6.53281

N95220 X1.55128 Y1.41775 Z6.57514

N95230 X1.58747 Y1.41775 Z6.59363

N95240 X1.61723 Y1.41775 Z6.60815

N95250 X1.71186 Y1.41775 Z6.65258

N95260 X1.83096 Y1.41775 Z6.70614

N95270 X1.96039 Y1.41775 Z6.75958

N95280 X1.97945 Y1.41775 Z6.76678

N95290 X2.04322 Y1.41775 Z6.79081

N95300 X2.09593 Y1.41775 Z6.81053

N95310 X2.1139 Y1.41775 Z6.817

N95320 X2.20103 Y1.41775 Z6.84592

N95330 X2.25768 Y1.41775 Z6.86326

N95340 X2.29854 Y1.41775 Z6.8746

N95350 X2.36444 Y1.41775 Z6.89051

N95360 X2.43227 Y1.41775 Z6.90584

N95370 X2.50307 Y1.41775 Z6.91989

N95380 X2.57653 Y1.41775 Z6.93322

N95390 X2.66999 Y1.41775 Z6.94726

N95400 X2.71015 Y1.41775 Z6.95261

N95410 X2.83025 Y1.41775 Z6.96414

N95420 X2.84745 Y1.41775 Z6.9656

N95430 X2.95331 Y1.41775 Z6.97078

N95440 X2.98482 Y1.41775 Z6.97228

N95450 X3.11314 Y1.41775 Z6.97276

N95460 X3.14962 Y1.41775 Z6.97241

N95470 X3.25459 Y1.41775 Z6.96968

N95480 X3.29252 Y1.41775 Z6.96811

N95490 X3.38855 Y1.41775 Z6.96067

N95500 X3.43129 Y1.41775 Z6.95673

N95510 X3.50433 Y1.41775 Z6.94913

N95520 X3.54414 Y1.41775 Z6.94449

N95530 X3.64833 Y1.41775 Z6.9289

N95540 X3.69683 Y1.41775 Z6.92154

N95550 X3.82151 Y1.41775 Z6.90006

N95560 X3.8929 Y1.41775 Z6.88701

N95570 X4.02091 Y1.41775 Z6.86195

N95580 X4.10753 Y1.41775 Z6.84348

N95590 X4.22673 Y1.41775 Z6.817

N95600 X4.35 Y1.41775 Z6.78686

N95610 X4.43087 Y1.41775 Z6.76682

N95620 X4.49233 Y1.41775 Z6.75028

N95630 X4.61064 Y1.41775 Z6.71758

N95640 X4.62818 Y1.41775 Z6.71185

N95650 X4.65741 Y1.41775 Z6.70172

N95660 X4.73215 Y1.41775 Z6.67587

N95670 X4.76718 Y1.41775 Z6.66145

N95680 X4.82479 Y1.41775 Z6.63559

N95690 X4.87461 Y1.41775 Z6.61124

N95700 X4.9101 Y1.41775 Z6.59276

N95710 X4.97143 Y1.41775 Z6.55988

N95720 X4.99261 Y1.41775 Z6.54773

N95730 X5.0695 Y1.41775 Z6.50323

N95740 X5.14854 Y1.41775 Z6.45205

N95750 X5.16425 Y1.41775 Z6.44154

N95760 X5.2265 Y1.41775 Z6.39676

N95770 X5.25075 Y1.41775 Z6.37915

N95780 X5.30215 Y1.41775 Z6.33864

N95790 X5.33589 Y1.41775 Z6.31168

N95800 X5.37382 Y1.41775 Z6.27855

N95810 X5.41669 Y1.41775 Z6.2403

N95820 X5.44322 Y1.41775 Z6.21428

N95830 X5.48858 Y1.41775 Z6.16833

N95840 X5.52089 Y1.41775 Z6.13313

N95850 X5.5557 Y1.41775 Z6.09426

N95860 X5.63551 Y1.41775 Z6.00176

N95870 X5.64375 Y1.41775 Z5.99199

N95880 X5.73969 Y1.41775 Z5.87415

N95890 X5.81472 Y1.41775 Z5.77843

N95900 X5.86588 Y1.41775 Z5.71284

N95910 X5.96338 Y1.41775 Z5.58161

N95920 X5.99025 Y1.41775 Z5.54474

N95930 X6.08897 Y1.41775 Z5.40507

N95940 X6.1147 Y1.41775 Z5.36737

N95950 X6.18755 Y1.41775 Z5.25809

N95960 X6.27946 Y1.41775 Z5.11478

N95970 X6.43199 Y1.41775 Z4.85146

N95980 X6.46273 Y1.41775 Z4.79192

N95990 X6.49786 Y1.41775 Z4.71827

N96000 X6.52359 Y1.41775 Z4.66328

N96010 X6.55718 Y1.41775 Z4.58297

N96020 X6.58396 Y1.41775 Z4.52214

N96030 X6.6252 Y1.41775 Z4.41561

N96040 X6.63449 Y1.41775 Z4.39259

N96050 X6.64574 Y1.41775 Z4.35986

N96060 X6.67721 Y1.41775 Z4.25961

N96070 X6.70998 Y1.41775 Z4.14148

N96080 X6.71592 Y1.41775 Z4.12125

N96090 X6.73137 Y1.41775 Z4.06927

N96100 X6.75031 Y1.41775 Z4.0038

N96110 X6.75305 Y1.41775 Z3.99398

N96120 X6.74046 Y1.28745 Z4.01105

N96130 X6.73576 Y1.28745 Z4.02684

N96140 X6.72394 Y1.28745 Z4.06709

N96150 X6.68383 Y1.28745 Z4.20052

N96160 X6.64801 Y1.28745 Z4.32437

N96170 X6.64128 Y1.28745 Z4.34682

N96180 X6.63405 Y1.28745 Z4.36613

N96190 X6.58839 Y1.28745 Z4.49296

N96200 X6.55599 Y1.28745 Z4.57795

N96210 X6.53386 Y1.28745 Z4.6309

N96220 X6.49282 Y1.28745 Z4.72565

N96230 X6.48405 Y1.28745 Z4.74401

N96240 X6.44303 Y1.28745 Z4.82599

N96250 X6.28856 Y1.28745 Z5.09744

N96260 X6.22558 Y1.28745 Z5.1968

N96270 X6.19528 Y1.28745 Z5.2444

N96280 X6.17993 Y1.28745 Z5.26721

N96290 X6.07084 Y1.28745 Z5.42702

N96300 X6.05586 Y1.28745 Z5.44812

N96310 X5.94243 Y1.28745 Z5.60376

N96320 X5.91547 Y1.28745 Z5.63943

N96330 X5.79183 Y1.28745 Z5.79804

N96340 X5.76289 Y1.28745 Z5.83407

N96350 X5.73851 Y1.28745 Z5.86287

N96360 X5.6517 Y1.28745 Z5.9667

N96370 X5.6277 Y1.28745 Z5.99552

N96380 X5.61796 Y1.28745 Z6.00686

N96390 X5.54764 Y1.28745 Z6.08561

N96400 X5.50845 Y1.28745 Z6.12869

N96410 X5.48796 Y1.28745 Z6.15106

N96420 X5.45822 Y1.28745 Z6.18229

N96430 X5.43625 Y1.28745 Z6.20426

N96440 X5.3752 Y1.28745 Z6.26065

N96450 X5.308 Y1.28745 Z6.31576

N96460 X5.29466 Y1.28745 Z6.32648

N96470 X5.23125 Y1.28745 Z6.37306

N96480 X5.20509 Y1.28745 Z6.39218

N96490 X5.14653 Y1.28745 Z6.43056

N96500 X5.11545 Y1.28745 Z6.45138

N96510 X5.04604 Y1.28745 Z6.49337

N96520 X5.0364 Y1.28745 Z6.49896

N96530 X5.02039 Y1.28745 Z6.50796

N96540 X4.95239 Y1.28745 Z6.54518

N96550 X4.93471 Y1.28745 Z6.55482

N96560 X4.85728 Y1.28745 Z6.5944

N96570 X4.83208 Y1.28745 Z6.6069

N96580 X4.75671 Y1.28745 Z6.64018

N96590 X4.7246 Y1.28745 Z6.65357

N96600 X4.6098 Y1.28745 Z6.69265

N96610 X4.59925 Y1.28745 Z6.6956

N96620 X4.46793 Y1.28745 Z6.73133

N96630 X4.38389 Y1.28745 Z6.75319

N96640 X4.24762 Y1.28745 Z6.78668

N96650 X4.15929 Y1.28745 Z6.80689

N96660 X4.02801 Y1.28745 Z6.83489

N96670 X3.93476 Y1.28745 Z6.85328

N96680 X3.81559 Y1.28745 Z6.87509

N96690 X3.72928 Y1.28745 Z6.88908

N96700 X3.61003 Y1.28745 Z6.90724

N96710 X3.54783 Y1.28745 Z6.91495

N96720 X3.43216 Y1.28745 Z6.92804

N96730 X3.3572 Y1.28745 Z6.93487

N96740 X3.27781 Y1.28745 Z6.94088

N96750 X3.20879 Y1.28745 Z6.94487

N96760 X3.15061 Y1.28745 Z6.94642

N96770 X3.08806 Y1.28745 Z6.94692

N96780 X3.00274 Y1.28745 Z6.94757

N96790 X2.96672 Y1.28745 Z6.94727

N96800 X2.87909 Y1.28745 Z6.94422

N96810 X2.84236 Y1.28745 Z6.94283

N96820 X2.83156 Y1.28745 Z6.942

N96830 X2.70982 Y1.28745 Z6.93148

N96840 X2.68835 Y1.28745 Z6.92897

N96850 X2.5768 Y1.28745 Z6.91386

N96860 X2.53944 Y1.28745 Z6.90788

N96870 X2.44852 Y1.28745 Z6.89119

N96880 X2.40048 Y1.28745 Z6.8812

N96890 X2.32795 Y1.28745 Z6.86483

N96900 X2.27224 Y1.28745 Z6.85057

N96910 X2.21611 Y1.28745 Z6.83497

N96920 X2.13125 Y1.28745 Z6.80902

N96930 X2.10509 Y1.28745 Z6.80027

N96940 X2.02079 Y1.28745 Z6.77116

N96950 X1.98571 Y1.28745 Z6.75896

N96960 X1.97133 Y1.28745 Z6.7538

N96970 X1.86799 Y1.28745 Z6.71444

N96980 X1.80009 Y1.28745 Z6.68673

N96990 X1.75713 Y1.28745 Z6.66855

N97000 X1.7116 Y1.28745 Z6.64833

N97010 X1.63835 Y1.28745 Z6.61506

N97020 X1.57934 Y1.28745 Z6.58656

N97030 X1.51861 Y1.28745 Z6.55692

N97040 X1.46736 Y1.28745 Z6.53074

N97050 X1.40154 Y1.28745 Z6.49551

N97060 X1.30012 Y1.28745 Z6.43809

N97070 X1.28478 Y1.28745 Z6.42913

N97080 X1.17936 Y1.28745 Z6.36203

N97090 X1.13795 Y1.28745 Z6.33455

N97100 X1.06658 Y1.28745 Z6.28551

N97110 X0.999485 Y1.28745 Z6.23813

N97120 X0.955864 Y1.28745 Z6.20604

N97130 X0.890864 Y1.28745 Z6.15722

N97140 X0.865509 Y1.28745 Z6.13692

N97150 X0.800723 Y1.28745 Z6.08437

N97160 X0.724672 Y1.28745 Z6.01837

N97170 X0.700641 Y1.28745 Z5.99753

N97180 X0.67429 Y1.28745 Z5.97287

N97190 X0.595266 Y1.28745 Z5.8985

N97200 X0.537455 Y1.28745 Z5.83947

N97210 X0.482269 Y1.28745 Z5.78226

N97220 X0.433633 Y1.28745 Z5.7274

N97230 X0.390533 Y1.28745 Z5.67751

N97240 X0.361432 Y1.28745 Z5.6418

N97250 X0.312348 Y1.28745 Z5.57968

N97260 X0.297686 Y1.28745 Z5.56168

N97270 X0.28637 Y1.28745 Z5.54734

N97280 X0.234127 Y1.28745 Z5.47863

N97290 X0.179461 Y1.28745 Z5.40463

N97300 X0.171851 Y1.28745 Z5.39394

N97310 X0.155965 Y1.28745 Z5.37139

N97320 X0.105476 Y1.28745 Z5.30145

N97330 X0.0765259 Y1.28745 Z5.26163

N97340 X-0.0310333 Y1.28745 Z5.11326

N97350 X-0.206112 Y1.28745 Z4.90024

N97360 X-0.214901 Y1.28745 Z4.89085

N97370 X-0.230876 Y1.28745 Z4.87525

N97380 X-0.260819 Y1.28745 Z4.84612

N97390 X-0.28255 Y1.28745 Z4.82688

N97400 X-0.312149 Y1.28745 Z4.80143

N97410 X-0.330915 Y1.28745 Z4.78657

N97420 X-0.354009 Y1.28745 Z4.76886

N97430 X-0.362432 Y1.28745 Z4.76277

N97440 X-0.37831 Y1.28745 Z4.75382

N97450 X-0.401431 Y1.28745 Z4.74352

N97460 X-0.414465 Y1.28745 Z4.7399

N97470 X-0.427431 Y1.28745 Z4.74146

N97480 X-0.446124 Y1.28745 Z4.7461

N97490 X-0.471452 Y1.28745 Z4.7536

N97500 X-0.487245 Y1.28745 Z4.75938

N97510 X-0.502996 Y1.28745 Z4.76525

N97520 X-0.535419 Y1.28745 Z4.7795

N97530 X-0.544565 Y1.28745 Z4.7841

N97540 X-0.578481 Y1.28745 Z4.80075

N97550 X-0.607726 Y1.28745 Z4.81745

N97560 X-0.65406 Y1.28745 Z4.84156

N97570 X-0.707282 Y1.28745 Z4.87189

N97580 X-0.792633 Y1.28745 Z4.91605

N97590 X-1.08353 Y1.28745 Z5.06066

N97600 X-1.11956 Y1.28745 Z5.07766

N97610 X-1.15675 Y1.28745 Z5.09439

N97620 X-1.29013 Y1.28745 Z5.15223

N97630 X-1.32089 Y1.28745 Z5.16421

N97640 X-1.37412 Y1.28745 Z5.18333

N97650 X-1.44615 Y1.28745 Z5.20954

N97660 X-1.48497 Y1.28745 Z5.22178

N97670 X-1.55358 Y1.28745 Z5.24172

N97680 X-1.60175 Y1.28745 Z5.25323

N97690 X-1.65829 Y1.28745 Z5.26549

N97700 X-1.75814 Y1.28745 Z5.28453

N97710 X-1.76987 Y1.28745 Z5.28649

N97720 X-1.79661 Y1.28745 Z5.29019

N97730 X-1.89688 Y1.28745 Z5.30385

N97740 X-1.9377 Y1.28745 Z5.30849

N97750 X-2.04691 Y1.28745 Z5.31977

N97760 X-2.16341 Y1.28745 Z5.33072

N97770 X-2.22707 Y1.28745 Z5.33668

N97780 X-2.3477 Y1.28745 Z5.348

N97790 X-2.41665 Y1.28745 Z5.35469

N97800 X-2.50125 Y1.28745 Z5.36372

N97810 X-2.59653 Y1.28745 Z5.37431

N97820 X-2.648 Y1.28745 Z5.38086

N97830 X-2.75388 Y1.28745 Z5.39482

N97840 X-2.86325 Y1.28745 Z5.41186

N97850 X-2.88489 Y1.28745 Z5.41559

N97860 X-2.98827 Y1.28745 Z5.43746

N97870 X-3.00302 Y1.28745 Z5.44072

N97880 X-3.12136 Y1.28745 Z5.4719

N97890 X-3.14848 Y1.28745 Z5.47949

N97900 X-3.2231 Y1.28745 Z5.50214

N97910 X-3.28099 Y1.28745 Z5.52256

N97920 X-3.3535 Y1.28745 Z5.54843

N97930 X-3.4536 Y1.28745 Z5.58587

N97940 X-3.54198 Y1.28745 Z5.61991

N97950 X-3.66776 Y1.28745 Z5.66951

N97960 X-3.71708 Y1.28745 Z5.6901

N97970 X-3.74598 Y1.28745 Z5.70254

N97980 X-3.90171 Y1.28745 Z5.76238

N97990 X-3.92198 Y1.28745 Z5.77028

N98000 X-3.93315 Y1.28745 Z5.77395

N98010 X-4.04956 Y1.28745 Z5.81407

N98020 X-4.08746 Y1.28745 Z5.82562

N98030 X-4.14579 Y1.28745 Z5.84335

N98040 X-4.24742 Y1.28745 Z5.87053

N98050 X-4.2593 Y1.28745 Z5.87329

N98060 X-4.3626 Y1.28745 Z5.89863

N98070 X-4.42168 Y1.28745 Z5.90952

N98080 X-4.47683 Y1.28745 Z5.91971

N98090 X-4.56278 Y1.28745 Z5.93161

N98100 X-4.57912 Y1.28745 Z5.93368

N98110 X-4.64825 Y1.28745 Z5.93932

N98120 X-4.69755 Y1.28745 Z5.94334

N98130 X-4.7174 Y1.28745 Z5.94368

N98140 X-4.79464 Y1.28745 Z5.94584

N98150 X-4.82667 Y1.28745 Z5.94521

N98160 X-4.8626 Y1.28745 Z5.94418

N98170 X-4.914 Y1.28745 Z5.94141

N98180 X-4.93999 Y1.28745 Z5.93943

N98190 X-5.03084 Y1.28745 Z5.92952

N98200 X-5.05093 Y1.28745 Z5.92603

N98210 X-5.14066 Y1.28745 Z5.91118

N98220 X-5.1798 Y1.28745 Z5.90319

N98230 X-5.27712 Y1.28745 Z5.88023

N98240 X-5.31407 Y1.28745 Z5.87082

N98250 X-5.40948 Y1.28745 Z5.8429

N98260 X-5.43675 Y1.28745 Z5.83505

N98270 X-5.5182 Y1.28745 Z5.80643

N98280 X-5.55089 Y1.28745 Z5.79488

N98290 X-5.56342 Y1.28745 Z5.7897

N98300 X-5.64861 Y1.28745 Z5.75362

N98310 X-5.7001 Y1.28745 Z5.72985

N98320 X-5.72446 Y1.28745 Z5.71783

N98330 X-5.78278 Y1.28745 Z5.68443

N98340 X-5.81492 Y1.28745 Z5.66511

N98350 X-5.86623 Y1.28745 Z5.63024

N98360 X-5.90743 Y1.28745 Z5.59978

N98370 X-5.95118 Y1.28745 Z5.56446

N98380 X-6.0222 Y1.28745 Z5.50163

N98390 X-6.05595 Y1.28745 Z5.4717

N98400 X-6.094 Y1.28745 Z5.43528

N98410 X-6.12421 Y1.28745 Z5.40499

N98420 X-6.15168 Y1.28745 Z5.37504

N98430 X-6.21562 Y1.28745 Z5.30101

N98440 X-6.2735 Y1.28745 Z5.22218

N98450 X-6.28359 Y1.28745 Z5.20683

N98460 X-6.32235 Y1.28745 Z5.14809

N98470 X-6.34745 Y1.28745 Z5.10959

N98480 X-6.4059 Y1.28745 Z5.00816

N98490 X-6.43854 Y1.28745 Z4.93785

N98500 X-6.45284 Y1.28745 Z4.90584

N98510 X-6.48458 Y1.28745 Z4.8354

N98520 X-6.49216 Y1.28745 Z4.81655

N98530 X-6.53058 Y1.28745 Z4.72167

N98540 X-6.56917 Y1.28745 Z4.60569

N98550 X-6.58191 Y1.28745 Z4.56467

N98560 X-6.60321 Y1.28745 Z4.48876

N98570 X-6.62546 Y1.28745 Z4.40896

N98580 X-6.63045 Y1.28745 Z4.38968

N98590 X-6.65463 Y1.28745 Z4.29063

N98600 X-6.66659 Y1.28745 Z4.24771

N98610 X-6.69026 Y1.28745 Z4.15856

N98620 X-6.66668 Y1.15862 Z4.13377

N98630 X-6.66378 Y1.15862 Z4.14457

N98640 X-6.6273 Y1.15862 Z4.27604

N98650 X-6.60978 Y1.15862 Z4.3503

N98660 X-6.59717 Y1.15862 Z4.39965

N98670 X-6.57774 Y1.15862 Z4.46858

N98680 X-6.54266 Y1.15862 Z4.58658

N98690 X-6.53775 Y1.15862 Z4.60199

N98700 X-6.50141 Y1.15862 Z4.70163

N98710 X-6.48125 Y1.15862 Z4.75342

N98720 X-6.45877 Y1.15862 Z4.80836

N98730 X-6.42859 Y1.15862 Z4.87795

N98740 X-6.41898 Y1.15862 Z4.89805

N98750 X-6.40459 Y1.15862 Z4.92968

N98760 X-6.36218 Y1.15862 Z5.01301

N98770 X-6.30307 Y1.15862 Z5.11229

N98780 X-6.29205 Y1.15862 Z5.1292

N98790 X-6.24182 Y1.15862 Z5.20556

N98800 X-6.17692 Y1.15862 Z5.29126

N98810 X-6.16966 Y1.15862 Z5.29971

N98820 X-6.11285 Y1.15862 Z5.36079

N98830 X-6.07403 Y1.15862 Z5.39988

N98840 X-6.04636 Y1.15862 Z5.42585

N98850 X-5.98063 Y1.15862 Z5.48441

N98860 X-5.96474 Y1.15862 Z5.49826

N98870 X-5.94572 Y1.15862 Z5.51394

N98880 X-5.87323 Y1.15862 Z5.57235

N98890 X-5.83588 Y1.15862 Z5.59832

N98900 X-5.78535 Y1.15862 Z5.6325

N98910 X-5.72814 Y1.15862 Z5.66633

N98920 X-5.69878 Y1.15862 Z5.68298

N98930 X-5.64316 Y1.15862 Z5.71022

N98940 X-5.60849 Y1.15862 Z5.72733

N98950 X-5.56909 Y1.15862 Z5.74417

N98960 X-5.50854 Y1.15862 Z5.76918

N98970 X-5.47054 Y1.15862 Z5.78262

N98980 X-5.39362 Y1.15862 Z5.81024

N98990 X-5.32973 Y1.15862 Z5.82899

N99000 X-5.27417 Y1.15862 Z5.84558

N99010 X-5.18908 Y1.15862 Z5.86562

N99020 X-5.14808 Y1.15862 Z5.87531

N99030 X-5.09068 Y1.15862 Z5.88564

N99040 X-5.04289 Y1.15862 Z5.89395

N99050 X-5.0013 Y1.15862 Z5.89904

N99060 X-4.9618 Y1.15862 Z5.90391

N99070 X-4.90483 Y1.15862 Z5.90826

N99080 X-4.87758 Y1.15862 Z5.91016

N99090 X-4.82225 Y1.15862 Z5.91205

N99100 X-4.77612 Y1.15862 Z5.91279

N99110 X-4.73121 Y1.15862 Z5.912

N99120 X-4.66373 Y1.15862 Z5.90915

N99130 X-4.61318 Y1.15862 Z5.90467

N99140 X-4.55923 Y1.15862 Z5.89926

N99150 X-4.48635 Y1.15862 Z5.88852

N99160 X-4.45999 Y1.15862 Z5.88456

N99170 X-4.41198 Y1.15862 Z5.87516

N99180 X-4.35315 Y1.15862 Z5.86431

N99190 X-4.31401 Y1.15862 Z5.85472

N99200 X-4.23407 Y1.15862 Z5.83595

N99210 X-4.16702 Y1.15862 Z5.81749

N99220 X-4.13239 Y1.15862 Z5.80787

N99230 X-4.04678 Y1.15862 Z5.78104

N99240 X-4.03649 Y1.15862 Z5.7779

N99250 X-3.92741 Y1.15862 Z5.73933

N99260 X-3.80854 Y1.15862 Z5.69111

N99270 X-3.7972 Y1.15862 Z5.68638

N99280 X-3.67536 Y1.15862 Z5.63493

N99290 X-3.6156 Y1.15862 Z5.6093

N99300 X-3.54627 Y1.15862 Z5.58058

N99310 X-3.4527 Y1.15862 Z5.54166

N99320 X-3.40386 Y1.15862 Z5.52274

N99330 X-3.3399 Y1.15862 Z5.49919

N99340 X-3.30966 Y1.15862 Z5.48853

N99350 X-3.21099 Y1.15862 Z5.45389

N99360 X-3.14786 Y1.15862 Z5.4357

N99370 X-3.09061 Y1.15862 Z5.41968

N99380 X-3.0486 Y1.15862 Z5.40861

N99390 X-2.95204 Y1.15862 Z5.3848

N99400 X-2.92743 Y1.15862 Z5.37944

N99410 X-2.82863 Y1.15862 Z5.36059

N99420 X-2.77973 Y1.15862 Z5.35238

N99430 X-2.71679 Y1.15862 Z5.34241

N99440 X-2.65721 Y1.15862 Z5.33448

N99450 X-2.57639 Y1.15862 Z5.32427

N99460 X-2.42381 Y1.15862 Z5.30724

N99470 X-2.39707 Y1.15862 Z5.30439

N99480 X-2.36416 Y1.15862 Z5.30119

N99490 X-2.21012 Y1.15862 Z5.28616

N99500 X-2.06809 Y1.15862 Z5.27245

N99510 X-2.04286 Y1.15862 Z5.26986

N99520 X-2.00309 Y1.15862 Z5.26519

N99530 X-1.9174 Y1.15862 Z5.25546

N99540 X-1.8793 Y1.15862 Z5.25027

N99550 X-1.80448 Y1.15862 Z5.23758

N99560 X-1.72673 Y1.15862 Z5.22343

N99570 X-1.68262 Y1.15862 Z5.21351

N99580 X-1.60548 Y1.15862 Z5.19554

N99590 X-1.56799 Y1.15862 Z5.18533

N99600 X-1.49783 Y1.15862 Z5.16417

N99610 X-1.3958 Y1.15862 Z5.13168

N99620 X-1.37521 Y1.15862 Z5.12518

N99630 X-1.35158 Y1.15862 Z5.11672

N99640 X-1.24277 Y1.15862 Z5.07481

N99650 X-1.17548 Y1.15862 Z5.04671

N99660 X-0.891787 Y1.15862 Z4.91453

N99670 X-0.82271 Y1.15862 Z4.88043

N99680 X-0.75396 Y1.15862 Z4.84834

N99690 X-0.702958 Y1.15862 Z4.82215

N99700 X-0.646737 Y1.15862 Z4.79605

N99710 X-0.624774 Y1.15862 Z4.78452

N99720 X-0.601896 Y1.15862 Z4.77454

N99730 X-0.584437 Y1.15862 Z4.76676

N99740 X-0.56893 Y1.15862 Z4.76157

N99750 X-0.556544 Y1.15862 Z4.75662

N99760 X-0.526465 Y1.15862 Z4.7471

N99770 X-0.503889 Y1.15862 Z4.74153

N99780 X-0.488654 Y1.15862 Z4.73855

N99790 X-0.477378 Y1.15862 Z4.73614

N99800 X-0.46475 Y1.15862 Z4.73492

N99810 X-0.442404 Y1.15862 Z4.74427

N99820 X-0.415289 Y1.15862 Z4.76193

N99830 X-0.383679 Y1.15862 Z4.78751

N99840 X-0.329834 Y1.15862 Z4.83606

N99850 X-0.320295 Y1.15862 Z4.84565

N99860 X-0.269523 Y1.15862 Z4.89711

N99870 X-0.188755 Y1.15862 Z4.9912

N99880 X-0.0818403 Y1.15862 Z5.12493

N99890 X0.0412304 Y1.15862 Z5.2842

N99900 X0.168533 Y1.15862 Z5.44745

N99910 X0.176228 Y1.15862 Z5.45696

N99920 X0.184493 Y1.15862 Z5.46705

N99930 X0.234838 Y1.15862 Z5.53036

N99940 X0.280506 Y1.15862 Z5.5863

N99950 X0.29217 Y1.15862 Z5.60009

N99960 X0.305322 Y1.15862 Z5.6151

N99970 X0.354433 Y1.15862 Z5.67313

N99980 X0.394878 Y1.15862 Z5.71916

N99990 X0.436683 Y1.15862 Z5.7641

N100000 X0.508643 Y1.15862 Z5.83878

N100010 X0.568135 Y1.15862 Z5.89527

N100020 X0.621759 Y1.15862 Z5.94608

N100030 X0.678151 Y1.15862 Z5.99498

N100040 X0.731742 Y1.15862 Z6.04178

N100050 X0.83729 Y1.15862 Z6.12584

N100060 X0.903583 Y1.15862 Z6.17505

N100070 X0.974543 Y1.15862 Z6.22725

N100080 X1.00777 Y1.15862 Z6.25022

N100090 X1.1055 Y1.15862 Z6.31697

N100100 X1.14624 Y1.15862 Z6.34318

N100110 X1.21803 Y1.15862 Z6.3884

N100120 X1.31767 Y1.15862 Z6.44721

N100130 X1.33485 Y1.15862 Z6.45694

N100140 X1.36118 Y1.15862 Z6.47104

N100150 X1.45585 Y1.15862 Z6.52078

N100160 X1.56915 Y1.15862 Z6.57747

N100170 X1.57878 Y1.15862 Z6.58201

N100180 X1.70459 Y1.15862 Z6.63814

N100190 X1.8372 Y1.15862 Z6.69095

N100200 X1.9522 Y1.15862 Z6.73197

N100210 X1.98581 Y1.15862 Z6.74405

N100220 X1.99978 Y1.15862 Z6.74891

N100230 X2.12973 Y1.15862 Z6.79068

N100240 X2.1687 Y1.15862 Z6.80266

N100250 X2.23809 Y1.15862 Z6.82208

N100260 X2.31013 Y1.15862 Z6.84072

N100270 X2.34472 Y1.15862 Z6.84878

N100280 X2.44101 Y1.15862 Z6.86914

N100290 X2.45871 Y1.15862 Z6.87242

N100300 X2.57935 Y1.15862 Z6.89207

N100310 X2.71054 Y1.15862 Z6.90744

N100320 X2.82542 Y1.15862 Z6.91601

N100330 X2.84871 Y1.15862 Z6.91694

N100340 X2.92523 Y1.15862 Z6.91852

N100350 X3.02571 Y1.15862 Z6.91927

N100360 X3.04157 Y1.15862 Z6.91922

N100370 X3.05279 Y1.15862 Z6.91899

N100380 X3.15635 Y1.15862 Z6.91617

N100390 X3.24459 Y1.15862 Z6.91174

N100400 X3.28796 Y1.15862 Z6.90944

N100410 X3.43196 Y1.15862 Z6.89678

N100420 X3.45217 Y1.15862 Z6.89504

N100430 X3.48789 Y1.15862 Z6.89115

N100440 X3.62826 Y1.15862 Z6.8738

N100450 X3.7298 Y1.15862 Z6.85933

N100460 X3.80951 Y1.15862 Z6.8464

N100470 X3.93148 Y1.15862 Z6.82485

N100480 X4.02968 Y1.15862 Z6.80553

N100490 X4.13046 Y1.15862 Z6.78394

N100500 X4.27344 Y1.15862 Z6.75134

N100510 X4.34731 Y1.15862 Z6.73386

N100520 X4.46993 Y1.15862 Z6.70218

N100530 X4.51633 Y1.15862 Z6.68893

N100540 X4.64166 Y1.15862 Z6.65084

N100550 X4.68755 Y1.15862 Z6.63351

N100560 X4.76701 Y1.15862 Z6.60306

N100570 X4.77657 Y1.15862 Z6.59883

N100580 X4.88369 Y1.15862 Z6.54879

N100590 X4.89398 Y1.15862 Z6.54349

N100600 X5.0142 Y1.15862 Z6.47923

N100610 X5.02477 Y1.15862 Z6.47329

N100620 X5.03645 Y1.15862 Z6.46652

N100630 X5.14696 Y1.15862 Z6.40205

N100640 X5.23869 Y1.15862 Z6.34082

N100650 X5.25767 Y1.15862 Z6.32713

N100660 X5.31631 Y1.15862 Z6.28171

N100670 X5.34666 Y1.15862 Z6.257

N100680 X5.38655 Y1.15862 Z6.22198

N100690 X5.44772 Y1.15862 Z6.16527

N100700 X5.45604 Y1.15862 Z6.1568

N100710 X5.51202 Y1.15862 Z6.09895

N100720 X5.57405 Y1.15862 Z6.03482

N100730 X5.58104 Y1.15862 Z6.02703

N100740 X5.59439 Y1.15862 Z6.01228

N100750 X5.65852 Y1.15862 Z5.94125

N100760 X5.68162 Y1.15862 Z5.91642

N100770 X5.7635 Y1.15862 Z5.82081

N100780 X5.77637 Y1.15862 Z5.80512

N100790 X5.87148 Y1.15862 Z5.68638

N100800 X5.89533 Y1.15862 Z5.65619

N100810 X5.99874 Y1.15862 Z5.51942

N100820 X6.01455 Y1.15862 Z5.49804

N100830 X6.12586 Y1.15862 Z5.34161

N100840 X6.13741 Y1.15862 Z5.32453

N100850 X6.21643 Y1.15862 Z5.20708

N100860 X6.2246 Y1.15862 Z5.19426

N100870 X6.2831 Y1.15862 Z5.09965

N100880 X6.35014 Y1.15862 Z4.98589

N100890 X6.36712 Y1.15862 Z4.95657

N100900 X6.44207 Y1.15862 Z4.81686

N100910 X6.48924 Y1.15862 Z4.71979

N100920 X6.50034 Y1.15862 Z4.69589

N100930 X6.51982 Y1.15862 Z4.64845

N100940 X6.55528 Y1.15862 Z4.56266

N100950 X6.57306 Y1.15862 Z4.51567

N100960 X6.60453 Y1.15862 Z4.424

N100970 X6.64599 Y1.15862 Z4.30147

N100980 X6.67965 Y1.15862 Z4.17573

N100990 X6.7132 Y1.15862 Z4.06433

N101000 X6.72588 Y1.15862 Z4.02148

N101010 X6.70865 Y1.0314 Z4.02782

N101020 X6.67482 Y1.0314 Z4.1442

N101030 X6.66472 Y1.0314 Z4.17867

N101040 X6.6213 Y1.0314 Z4.33329

N101050 X6.61442 Y1.0314 Z4.35947

N101060 X6.5882 Y1.0314 Z4.44237

N101070 X6.56551 Y1.0314 Z4.51076

N101080 X6.54311 Y1.0314 Z4.56835

N101090 X6.51306 Y1.0314 Z4.6449

N101100 X6.49866 Y1.0314 Z4.67814

N101110 X6.45558 Y1.0314 Z4.77049

N101120 X6.41706 Y1.0314 Z4.84923

N101130 X6.29077 Y1.0314 Z5.07539

N101140 X6.26496 Y1.0314 Z5.11726

N101150 X6.22055 Y1.0314 Z5.18631

N101160 X6.17697 Y1.0314 Z5.25355

N101170 X6.15658 Y1.0314 Z5.28377

N101180 X6.0748 Y1.0314 Z5.40246

N101190 X6.0525 Y1.0314 Z5.43421

N101200 X5.95978 Y1.0314 Z5.55932

N101210 X5.93651 Y1.0314 Z5.58901

N101220 X5.8513 Y1.0314 Z5.69701

N101230 X5.83314 Y1.0314 Z5.71891

N101240 X5.76707 Y1.0314 Z5.79793

N101250 X5.74405 Y1.0314 Z5.82534

N101260 X5.73742 Y1.0314 Z5.83314

N101270 X5.66807 Y1.0314 Z5.91018

N101280 X5.63374 Y1.0314 Z5.94739

N101290 X5.59659 Y1.0314 Z5.98512

N101300 X5.5317 Y1.0314 Z6.05094

N101310 X5.52213 Y1.0314 Z6.06071

N101320 X5.45722 Y1.0314 Z6.12164

N101330 X5.40369 Y1.0314 Z6.17204

N101340 X5.38822 Y1.0314 Z6.18506

N101350 X5.35369 Y1.0314 Z6.214

N101360 X5.31451 Y1.0314 Z6.24681

N101370 X5.29941 Y1.0314 Z6.2592

N101380 X5.22796 Y1.0314 Z6.3116

N101390 X5.20763 Y1.0314 Z6.32628

N101400 X5.16155 Y1.0314 Z6.35564

N101410 X5.11816 Y1.0314 Z6.38308

N101420 X5.08678 Y1.0314 Z6.40145

N101430 X5.02453 Y1.0314 Z6.43651

N101440 X4.91261 Y1.0314 Z6.49643

N101450 X4.79781 Y1.0314 Z6.55087

N101460 X4.68783 Y1.0314 Z6.59625

N101470 X4.66936 Y1.0314 Z6.60326

N101480 X4.5655 Y1.0314 Z6.63899

N101490 X4.52409 Y1.0314 Z6.65247

N101500 X4.4255 Y1.0314 Z6.6805

N101510 X4.36931 Y1.0314 Z6.69534

N101520 X4.22051 Y1.0314 Z6.73086

N101530 X4.18142 Y1.0314 Z6.73923

N101540 X4.11491 Y1.0314 Z6.75405

N101550 X3.98344 Y1.0314 Z6.78154

N101560 X3.86067 Y1.0314 Z6.80521

N101570 X3.84563 Y1.0314 Z6.80794

N101580 X3.81132 Y1.0314 Z6.81336

N101590 X3.70401 Y1.0314 Z6.82996

N101600 X3.66898 Y1.0314 Z6.835

N101610 X3.56299 Y1.0314 Z6.84915

N101620 X3.46547 Y1.0314 Z6.86017

N101630 X3.42915 Y1.0314 Z6.86413

N101640 X3.28441 Y1.0314 Z6.87656

N101650 X3.25276 Y1.0314 Z6.87824

N101660 X3.12454 Y1.0314 Z6.88468

N101670 X3.09725 Y1.0314 Z6.88543

N101680 X2.98973 Y1.0314 Z6.88762

N101690 X2.94213 Y1.0314 Z6.88777

N101700 X2.87508 Y1.0314 Z6.88702

N101710 X2.75696 Y1.0314 Z6.8827

N101720 X2.7466 Y1.0314 Z6.88184

N101730 X2.63729 Y1.0314 Z6.87194

N101740 X2.60026 Y1.0314 Z6.86762

N101750 X2.48933 Y1.0314 Z6.85307

N101760 X2.46859 Y1.0314 Z6.84969

N101770 X2.35526 Y1.0314 Z6.82865

N101780 X2.25365 Y1.0314 Z6.80429

N101790 X2.21812 Y1.0314 Z6.79537

N101800 X2.15523 Y1.0314 Z6.77758

N101810 X2.07079 Y1.0314 Z6.75338

N101820 X2.05003 Y1.0314 Z6.7467

N101830 X1.93694 Y1.0314 Z6.7106

N101840 X1.86965 Y1.0314 Z6.68727

N101850 X1.80135 Y1.0314 Z6.66349

N101860 X1.74154 Y1.0314 Z6.6412

N101870 X1.67713 Y1.0314 Z6.61618

N101880 X1.57912 Y1.0314 Z6.57356

N101890 X1.54436 Y1.0314 Z6.55795

N101900 X1.41647 Y1.0314 Z6.49463

N101910 X1.3976 Y1.0314 Z6.48519

N101920 X1.37539 Y1.0314 Z6.47352

N101930 X1.2626 Y1.0314 Z6.41225

N101940 X1.17125 Y1.0314 Z6.35882

N101950 X1.1468 Y1.0314 Z6.34418

N101960 X1.10636 Y1.0314 Z6.31842

N101970 X1.03189 Y1.0314 Z6.27051

N101980 X0.993311 Y1.0314 Z6.24416

N101990 X0.912919 Y1.0314 Z6.18974

N102000 X0.827527 Y1.0314 Z6.12805

N102010 X0.795619 Y1.0314 Z6.10466

N102020 X0.662411 Y1.0314 Z5.99939

N102030 X0.652559 Y1.0314 Z5.99079

N102040 X0.535519 Y1.0314 Z5.88958

N102050 X0.453653 Y1.0314 Z5.81087

N102060 X0.420793 Y1.0314 Z5.77972

N102070 X0.37798 Y1.0314 Z5.73522

N102080 X0.325546 Y1.0314 Z5.68029

N102090 X0.301914 Y1.0314 Z5.65373

N102100 X0.253595 Y1.0314 Z5.60071

N102110 X0.234583 Y1.0314 Z5.57864

N102120 X0.200859 Y1.0314 Z5.53939

N102130 X0.170106 Y1.0314 Z5.50443

N102140 X0.144177 Y1.0314 Z5.47462

N102150 X0.0998959 Y1.0314 Z5.42141

N102160 X0.0385324 Y1.0314 Z5.34741

N102170 X0.0293286 Y1.0314 Z5.33656

N102180 X-0.0677956 Y1.0314 Z5.2189

N102190 X-0.110706 Y1.0314 Z5.16451

N102200 X-0.261556 Y1.0314 Z4.98015

N102210 X-0.269744 Y1.0314 Z4.97008

N102220 X-0.279985 Y1.0314 Z4.95794

N102230 X-0.328089 Y1.0314 Z4.90155

N102240 X-0.36893 Y1.0314 Z4.85725

N102250 X-0.377844 Y1.0314 Z4.84813

N102260 X-0.417429 Y1.0314 Z4.80907

N102270 X-0.430771 Y1.0314 Z4.79675

N102280 X-0.455426 Y1.0314 Z4.77434

N102290 X-0.48452 Y1.0314 Z4.74957

N102300 X-0.506042 Y1.0314 Z4.73334

N102310 X-0.516113 Y1.0314 Z4.72672

N102320 X-0.529282 Y1.0314 Z4.72686

N102330 X-0.542113 Y1.0314 Z4.72879

N102340 X-0.55754 Y1.0314 Z4.73029

N102350 X-0.571245 Y1.0314 Z4.73331

N102360 X-0.58713 Y1.0314 Z4.73641

N102370 X-0.610493 Y1.0314 Z4.74288

N102380 X-0.630866 Y1.0314 Z4.74808

N102390 X-0.652777 Y1.0314 Z4.75423

N102400 X-0.687086 Y1.0314 Z4.76697

N102410 X-0.702001 Y1.0314 Z4.77315

N102420 X-0.750957 Y1.0314 Z4.79333

N102430 X-0.774954 Y1.0314 Z4.80456

N102440 X-0.841179 Y1.0314 Z4.83319

N102450 X-0.887299 Y1.0314 Z4.85486

N102460 X-0.944026 Y1.0314 Z4.88022

N102470 X-1.00708 Y1.0314 Z4.90986

N102480 X-1.04789 Y1.0314 Z4.92848

N102490 X-1.15017 Y1.0314 Z4.9753

N102500 X-1.25904 Y1.0314 Z5.02009

N102510 X-1.38482 Y1.0314 Z5.06617

N102520 X-1.39601 Y1.0314 Z5.06985

N102530 X-1.52702 Y1.0314 Z5.11011

N102540 X-1.56474 Y1.0314 Z5.1212

N102550 X-1.69479 Y1.0314 Z5.15664

N102560 X-1.7163 Y1.0314 Z5.1616

N102570 X-1.8305 Y1.0314 Z5.18728

N102580 X-1.84176 Y1.0314 Z5.18933

N102590 X-1.8646 Y1.0314 Z5.19321

N102600 X-1.96709 Y1.0314 Z5.21095

N102610 X-2.03865 Y1.0314 Z5.2201

N102620 X-2.06418 Y1.0314 Z5.2231

N102630 X-2.12932 Y1.0314 Z5.22978

N102640 X-2.2024 Y1.0314 Z5.23748

N102650 X-2.25352 Y1.0314 Z5.24303

N102660 X-2.38085 Y1.0314 Z5.25618

N102670 X-2.45891 Y1.0314 Z5.26485

N102680 X-2.55159 Y1.0314 Z5.27615

N102690 X-2.62863 Y1.0314 Z5.28631

N102700 X-2.68077 Y1.0314 Z5.29408

N102710 X-2.74356 Y1.0314 Z5.30432

N102720 X-2.81423 Y1.0314 Z5.31682

N102730 X-2.88346 Y1.0314 Z5.33011

N102740 X-2.94483 Y1.0314 Z5.34241

N102750 X-3.0293 Y1.0314 Z5.36265

N102760 X-3.05854 Y1.0314 Z5.37015

N102770 X-3.08192 Y1.0314 Z5.37622

N102780 X-3.18309 Y1.0314 Z5.40483

N102790 X-3.19501 Y1.0314 Z5.40824

N102800 X-3.27119 Y1.0314 Z5.43473

N102810 X-3.28228 Y1.0314 Z5.4389

N102820 X-3.34124 Y1.0314 Z5.46198

N102830 X-3.39126 Y1.0314 Z5.48174

N102840 X-3.40972 Y1.0314 Z5.48935

N102850 X-3.53059 Y1.0314 Z5.53996

N102860 X-3.64142 Y1.0314 Z5.58699

N102870 X-3.67873 Y1.0314 Z5.60301

N102880 X-3.71396 Y1.0314 Z5.61804

N102890 X-3.80977 Y1.0314 Z5.65937

N102900 X-3.89368 Y1.0314 Z5.69326

N102910 X-3.92981 Y1.0314 Z5.70815

N102920 X-3.9603 Y1.0314 Z5.71965

N102930 X-4.02472 Y1.0314 Z5.74338

N102940 X-4.09732 Y1.0314 Z5.767

N102950 X-4.11671 Y1.0314 Z5.77308

N102960 X-4.17638 Y1.0314 Z5.78965

N102970 X-4.21842 Y1.0314 Z5.8014

N102980 X-4.23176 Y1.0314 Z5.80475

N102990 X-4.33513 Y1.0314 Z5.82928

N103000 X-4.3656 Y1.0314 Z5.83553

N103010 X-4.4259 Y1.0314 Z5.84724

N103020 X-4.45258 Y1.0314 Z5.85246

N103030 X-4.51257 Y1.0314 Z5.86147

N103040 X-4.54689 Y1.0314 Z5.86639

N103050 X-4.64044 Y1.0314 Z5.87593

N103060 X-4.66412 Y1.0314 Z5.87717

N103070 X-4.74428 Y1.0314 Z5.88059

N103080 X-4.84628 Y1.0314 Z5.87919

N103090 X-4.87024 Y1.0314 Z5.87771

N103100 X-4.94272 Y1.0314 Z5.87266

N103110 X-4.97298 Y1.0314 Z5.86906

N103120 X-5.03513 Y1.0314 Z5.86144

N103130 X-5.11428 Y1.0314 Z5.84754

N103140 X-5.13442 Y1.0314 Z5.84392

N103150 X-5.18986 Y1.0314 Z5.83081

N103160 X-5.24048 Y1.0314 Z5.81857

N103170 X-5.25573 Y1.0314 Z5.81389

N103180 X-5.35427 Y1.0314 Z5.78417

N103190 X-5.38598 Y1.0314 Z5.77245

N103200 X-5.46511 Y1.0314 Z5.74366

N103210 X-5.5346 Y1.0314 Z5.71317

N103220 X-5.57802 Y1.0314 Z5.69461

N103230 X-5.61879 Y1.0314 Z5.67449

N103240 X-5.68201 Y1.0314 Z5.64142

N103250 X-5.74933 Y1.0314 Z5.60171

N103260 X-5.77469 Y1.0314 Z5.58622

N103270 X-5.86612 Y1.0314 Z5.52301

N103280 X-5.87452 Y1.0314 Z5.51624

N103290 X-5.94424 Y1.0314 Z5.45878

N103300 X-6.01396 Y1.0314 Z5.39795

N103310 X-6.08051 Y1.0314 Z5.33343

N103320 X-6.12339 Y1.0314 Z5.29036

N103330 X-6.13257 Y1.0314 Z5.28043

N103340 X-6.19104 Y1.0314 Z5.20941

N103350 X-6.22186 Y1.0314 Z5.16126

N103360 X-6.24562 Y1.0314 Z5.12565

N103370 X-6.272 Y1.0314 Z5.08526

N103380 X-6.30713 Y1.0314 Z5.03273

N103390 X-6.31398 Y1.0314 Z5.02119

N103400 X-6.34922 Y1.0314 Z4.95547

N103410 X-6.3676 Y1.0314 Z4.92

N103420 X-6.38582 Y1.0314 Z4.87996

N103430 X-6.42518 Y1.0314 Z4.79682

N103440 X-6.47048 Y1.0314 Z4.68422

N103450 X-6.47616 Y1.0314 Z4.66853

N103460 X-6.49561 Y1.0314 Z4.61138

N103470 X-6.52322 Y1.0314 Z4.52931

N103480 X-6.53666 Y1.0314 Z4.48537

N103490 X-6.56823 Y1.0314 Z4.36927

N103500 X-6.5865 Y1.0314 Z4.2975

N103510 X-6.6254 Y1.0314 Z4.15712

N103520 X-6.63814 Y1.0314 Z4.10978

N103530 X-6.60756 Y0.904923 Z4.08384

N103540 X-6.58308 Y0.904923 Z4.17143

N103550 X-6.56459 Y0.904923 Z4.23862

N103560 X-6.53709 Y0.904923 Z4.3403

N103570 X-6.50973 Y0.904923 Z4.44111

N103580 X-6.48124 Y0.904923 Z4.53586

N103590 X-6.46149 Y0.904923 Z4.59392

N103600 X-6.42926 Y0.904923 Z4.68289

N103610 X-6.41102 Y0.904923 Z4.72875

N103620 X-6.38663 Y0.904923 Z4.78341

N103630 X-6.34958 Y0.904923 Z4.86319

N103640 X-6.31584 Y0.904923 Z4.93077

N103650 X-6.30952 Y0.904923 Z4.94297

N103660 X-6.25498 Y0.904923 Z5.03522

N103670 X-6.2476 Y0.904923 Z5.04648

N103680 X-6.20291 Y0.904923 Z5.11284

N103690 X-6.19527 Y0.904923 Z5.1234

N103700 X-6.16028 Y0.904923 Z5.17167

N103710 X-6.13627 Y0.904923 Z5.20444

N103720 X-6.12827 Y0.904923 Z5.21423

N103730 X-6.09498 Y0.904923 Z5.25078

N103740 X-6.05737 Y0.904923 Z5.2919

N103750 X-6.04599 Y0.904923 Z5.30359

N103760 X-5.98807 Y0.904923 Z5.35797

N103770 X-5.94048 Y0.904923 Z5.40201

N103780 X-5.91593 Y0.904923 Z5.42418

N103790 X-5.88023 Y0.904923 Z5.45392

N103800 X-5.84555 Y0.904923 Z5.48186

N103810 X-5.77153 Y0.904923 Z5.53321

N103820 X-5.75896 Y0.904923 Z5.5419

N103830 X-5.69343 Y0.904923 Z5.58194

N103840 X-5.66507 Y0.904923 Z5.59917

N103850 X-5.55954 Y0.904923 Z5.65506

N103860 X-5.52705 Y0.904923 Z5.67137

N103870 X-5.43537 Y0.904923 Z5.7118

N103880 X-5.42079 Y0.904923 Z5.71785

N103890 X-5.33773 Y0.904923 Z5.74913

N103900 X-5.31706 Y0.904923 Z5.75677

N103910 X-5.20797 Y0.904923 Z5.7902

N103920 X-5.18961 Y0.904923 Z5.79465

N103930 X-5.10455 Y0.904923 Z5.81413

N103940 X-5.05355 Y0.904923 Z5.82337

N103950 X-5.0095 Y0.904923 Z5.83085

N103960 X-4.93697 Y0.904923 Z5.83962

N103970 X-4.91403 Y0.904923 Z5.84233

N103980 X-4.83238 Y0.904923 Z5.84739

N103990 X-4.81945 Y0.904923 Z5.84805

N104000 X-4.74867 Y0.904923 Z5.84846

N104010 X-4.72263 Y0.904923 Z5.84858

N104020 X-4.6227 Y0.904923 Z5.84335

N104030 X-4.60267 Y0.904923 Z5.84131

N104040 X-4.52247 Y0.904923 Z5.83237

N104050 X-4.49204 Y0.904923 Z5.82779

N104060 X-4.42296 Y0.904923 Z5.81645

N104070 X-4.39797 Y0.904923 Z5.8116

N104080 X-4.30926 Y0.904923 Z5.79341

N104090 X-4.28487 Y0.904923 Z5.78762

N104100 X-4.19576 Y0.904923 Z5.76525

N104110 X-4.15165 Y0.904923 Z5.75293

N104120 X-4.08592 Y0.904923 Z5.73259

N104130 X-4.02716 Y0.904923 Z5.71352

N104140 X-3.97046 Y0.904923 Z5.6925

N104150 X-3.92678 Y0.904923 Z5.67601

N104160 X-3.86636 Y0.904923 Z5.65112

N104170 X-3.80487 Y0.904923 Z5.62576

N104180 X-3.7264 Y0.904923 Z5.59203

N104190 X-3.66243 Y0.904923 Z5.56475

N104200 X-3.59553 Y0.904923 Z5.53602

N104210 X-3.50899 Y0.904923 Z5.49909

N104220 X-3.44423 Y0.904923 Z5.47207

N104230 X-3.36401 Y0.904923 Z5.43878

N104240 X-3.25823 Y0.904923 Z5.39659

N104250 X-3.24504 Y0.904923 Z5.39142

N104260 X-3.17241 Y0.904923 Z5.36465

N104270 X-3.13862 Y0.904923 Z5.35408

N104280 X-3.11599 Y0.904923 Z5.3476

N104290 X-3.10041 Y0.904923 Z5.34319

N104300 X-3.02451 Y0.904923 Z5.32348

N104310 X-2.96314 Y0.904923 Z5.30775

N104320 X-2.94648 Y0.904923 Z5.30372

N104330 X-2.9355 Y0.904923 Z5.30135

N104340 X-2.82424 Y0.904923 Z5.27901

N104350 X-2.81355 Y0.904923 Z5.27692

N104360 X-2.80195 Y0.904923 Z5.27473

N104370 X-2.67317 Y0.904923 Z5.25192

N104380 X-2.64163 Y0.904923 Z5.24662

N104390 X-2.5321 Y0.904923 Z5.23028

N104400 X-2.48617 Y0.904923 Z5.22375

N104410 X-2.37146 Y0.904923 Z5.20953

N104420 X-2.20304 Y0.904923 Z5.19001

N104430 X-2.18673 Y0.904923 Z5.18829

N104440 X-2.04023 Y0.904923 Z5.16945

N104450 X-2.02589 Y0.904923 Z5.16723

N104460 X-1.92003 Y0.904923 Z5.14816

N104470 X-1.84628 Y0.904923 Z5.13122

N104480 X-1.7947 Y0.904923 Z5.11958

N104490 X-1.67906 Y0.904923 Z5.08936

N104500 X-1.66654 Y0.904923 Z5.08595

N104510 X-1.51412 Y0.904923 Z5.04111

N104520 X-1.36054 Y0.904923 Z4.98934

N104530 X-1.34437 Y0.904923 Z4.98359

N104540 X-1.19208 Y0.904923 Z4.92452

N104550 X-1.11595 Y0.904923 Z4.89232

N104560 X-1.07885 Y0.904923 Z4.87544

N104570 X-1.00669 Y0.904923 Z4.84402

N104580 X-0.976149 Y0.904923 Z4.82998

N104590 X-0.894731 Y0.904923 Z4.79549

N104600 X-0.885266 Y0.904923 Z4.79123

N104610 X-0.861796 Y0.904923 Z4.78178

N104620 X-0.815962 Y0.904923 Z4.76312

N104630 X-0.800252 Y0.904923 Z4.75792

N104640 X-0.768376 Y0.904923 Z4.74602

N104650 X-0.739622 Y0.904923 Z4.73791

N104660 X-0.725287 Y0.904923 Z4.73418

N104670 X-0.693829 Y0.904923 Z4.72584

N104680 X-0.657076 Y0.904923 Z4.71929

N104690 X-0.627636 Y0.904923 Z4.71416

N104700 X-0.61075 Y0.904923 Z4.71353

N104710 X-0.599108 Y0.904923 Z4.71303

N104720 X-0.58933 Y0.904923 Z4.71703

N104730 X-0.580288 Y0.904923 Z4.7217

N104740 X-0.571379 Y0.904923 Z4.72765

N104750 X-0.56165 Y0.904923 Z4.73501

N104760 X-0.550134 Y0.904923 Z4.74374

N104770 X-0.532132 Y0.904923 Z4.75882

N104780 X-0.514234 Y0.904923 Z4.77516

N104790 X-0.50624 Y0.904923 Z4.78254

N104800 X-0.476402 Y0.904923 Z4.81211

N104810 X-0.463967 Y0.904923 Z4.82444

N104820 X-0.456929 Y0.904923 Z4.8322

N104830 X-0.40916 Y0.904923 Z4.88419

N104840 X-0.349864 Y0.904923 Z4.95311

N104850 X-0.338528 Y0.904923 Z4.96625

N104860 X-0.221 Y0.904923 Z5.10767

N104870 X-0.127536 Y0.904923 Z5.21906

N104880 X0.0793032 Y0.904923 Z5.45669

N104890 X0.109348 Y0.904923 Z5.49024

N104900 X0.158649 Y0.904923 Z5.54316

N104910 X0.225315 Y0.904923 Z5.61329

N104920 X0.246507 Y0.904923 Z5.63609

N104930 X0.256245 Y0.904923 Z5.64677

N104940 X0.292897 Y0.904923 Z5.68391

N104950 X0.355019 Y0.904923 Z5.74655

N104960 X0.380783 Y0.904923 Z5.77085

N104970 X0.442502 Y0.904923 Z5.82965

N104980 X0.506163 Y0.904923 Z5.88567

N104990 X0.544887 Y0.904923 Z5.91962

N105000 X0.635355 Y0.904923 Z5.99324

N105010 X0.66892 Y0.904923 Z6.01953

N105020 X0.742574 Y0.904923 Z6.07513

N105030 X0.846264 Y0.904923 Z6.14854

N105040 X0.959429 Y0.904923 Z6.22408

N105050 X1.02723 Y0.904923 Z6.26721

N105060 X1.07397 Y0.904923 Z6.29697

N105070 X1.15144 Y0.904923 Z6.34336

N105080 X1.18737 Y0.904923 Z6.36472

N105090 X1.20944 Y0.904923 Z6.37694

N105100 X1.30886 Y0.904923 Z6.43038

N105110 X1.39407 Y0.904923 Z6.47396

N105120 X1.45942 Y0.904923 Z6.5064

N105130 X1.58039 Y0.904923 Z6.56219

N105140 X1.59669 Y0.904923 Z6.56929

N105150 X1.67001 Y0.904923 Z6.59743

N105160 X1.75121 Y0.904923 Z6.62885

N105170 X1.76634 Y0.904923 Z6.63406

N105180 X1.79439 Y0.904923 Z6.6434

N105190 X1.90385 Y0.904923 Z6.68011

N105200 X1.97853 Y0.904923 Z6.70406

N105210 X1.99806 Y0.904923 Z6.71001

N105220 X2.02228 Y0.904923 Z6.71711

N105230 X2.07901 Y0.904923 Z6.73337

N105240 X2.11233 Y0.904923 Z6.74303

N105250 X2.16527 Y0.904923 Z6.75686

N105260 X2.25348 Y0.904923 Z6.77965

N105270 X2.26326 Y0.904923 Z6.78207

N105280 X2.35738 Y0.904923 Z6.80185

N105290 X2.39831 Y0.904923 Z6.80965

N105300 X2.47097 Y0.904923 Z6.82203

N105310 X2.54123 Y0.904923 Z6.83147

N105320 X2.59938 Y0.904923 Z6.83816

N105330 X2.66866 Y0.904923 Z6.84408

N105340 X2.71797 Y0.904923 Z6.84816

N105350 X2.78602 Y0.904923 Z6.85152

N105360 X2.82729 Y0.904923 Z6.85312

N105370 X2.85033 Y0.904923 Z6.85365

N105380 X2.9332 Y0.904923 Z6.85423

N105390 X2.99439 Y0.904923 Z6.85323

N105400 X3.05422 Y0.904923 Z6.85157

N105410 X3.14627 Y0.904923 Z6.84837

N105420 X3.19567 Y0.904923 Z6.84584

N105430 X3.29624 Y0.904923 Z6.83973

N105440 X3.33799 Y0.904923 Z6.83628

N105450 X3.44633 Y0.904923 Z6.82585

N105460 X3.48077 Y0.904923 Z6.82214

N105470 X3.61219 Y0.904923 Z6.80555

N105480 X3.62606 Y0.904923 Z6.80363

N105490 X3.67004 Y0.904923 Z6.79703

N105500 X3.78861 Y0.904923 Z6.77888

N105510 X3.83659 Y0.904923 Z6.77051

N105520 X3.96333 Y0.904923 Z6.747

N105530 X4.0884 Y0.904923 Z6.72108

N105540 X4.16149 Y0.904923 Z6.70479

N105550 X4.2469 Y0.904923 Z6.68651

N105560 X4.32018 Y0.904923 Z6.66939

N105570 X4.35863 Y0.904923 Z6.66017

N105580 X4.4393 Y0.904923 Z6.63865

N105590 X4.49175 Y0.904923 Z6.62231

N105600 X4.57311 Y0.904923 Z6.59606

N105610 X4.61109 Y0.904923 Z6.58245

N105620 X4.70624 Y0.904923 Z6.54648

N105630 X4.72974 Y0.904923 Z6.53684

N105640 X4.82977 Y0.904923 Z6.49359

N105650 X4.84786 Y0.904923 Z6.4852

N105660 X4.93454 Y0.904923 Z6.4434

N105670 X4.96175 Y0.904923 Z6.42911

N105680 X5.02288 Y0.904923 Z6.39566

N105690 X5.0813 Y0.904923 Z6.36205

N105700 X5.12289 Y0.904923 Z6.33769

N105710 X5.21496 Y0.904923 Z6.27795

N105720 X5.22604 Y0.904923 Z6.27078

N105730 X5.31155 Y0.904923 Z6.20763

N105740 X5.33748 Y0.904923 Z6.18861

N105750 X5.39943 Y0.904923 Z6.13677

N105760 X5.45776 Y0.904923 Z6.08806

N105770 X5.47852 Y0.904923 Z6.06866

N105780 X5.5045 Y0.904923 Z6.04459

N105790 X5.55149 Y0.904923 Z6.00156

N105800 X5.58861 Y0.904923 Z5.96666

N105810 X5.6241 Y0.904923 Z5.93071

N105820 X5.69571 Y0.904923 Z5.85568

N105830 X5.70307 Y0.904923 Z5.84753

N105840 X5.73429 Y0.904923 Z5.81215

N105850 X5.79045 Y0.904923 Z5.74841

N105860 X5.80328 Y0.904923 Z5.73349

N105870 X5.88495 Y0.904923 Z5.63465

N105880 X5.91616 Y0.904923 Z5.59583

N105890 X5.9764 Y0.904923 Z5.51866

N105900 X6.0428 Y0.904923 Z5.42928

N105910 X6.06186 Y0.904923 Z5.40283

N105920 X6.0782 Y0.904923 Z5.37937

N105930 X6.15317 Y0.904923 Z5.26956

N105940 X6.22605 Y0.904923 Z5.16043

N105950 X6.23692 Y0.904923 Z5.14352

N105960 X6.24736 Y0.904923 Z5.12658

N105970 X6.34796 Y0.904923 Z4.95616

N105980 X6.37055 Y0.904923 Z4.91481

N105990 X6.41265 Y0.904923 Z4.82944

N106000 X6.44147 Y0.904923 Z4.77127

N106010 X6.46826 Y0.904923 Z4.71163

N106020 X6.5115 Y0.904923 Z4.61378

N106030 X6.51973 Y0.904923 Z4.59329

N106040 X6.54085 Y0.904923 Z4.53817

N106050 X6.56142 Y0.904923 Z4.48282

N106060 X6.5923 Y0.904923 Z4.38489

N106070 X6.60017 Y0.904923 Z4.35928

N106080 X6.61038 Y0.904923 Z4.32143

N106090 X6.64619 Y0.904923 Z4.18653

N106100 X6.66371 Y0.904923 Z4.12673

N106110 X6.69072 Y0.904923 Z4.03236

N106120 X6.67179 Y0.780845 Z4.03331

N106130 X6.63755 Y0.780845 Z4.15268

N106140 X6.61475 Y0.780845 Z4.24123

N106150 X6.60623 Y0.780845 Z4.2732

N106160 X6.57294 Y0.780845 Z4.38662

N106170 X6.56581 Y0.780845 Z4.40984

N106180 X6.52664 Y0.780845 Z4.52415

N106190 X6.52235 Y0.780845 Z4.53535

N106200 X6.50747 Y0.780845 Z4.57239

N106210 X6.47406 Y0.780845 Z4.65483

N106220 X6.46386 Y0.780845 Z4.67902

N106230 X6.42162 Y0.780845 Z4.77154

N106240 X6.39425 Y0.780845 Z4.83118

N106250 X6.36856 Y0.780845 Z4.88273

N106260 X6.2831 Y0.780845 Z5.04181

N106270 X6.2643 Y0.780845 Z5.07259

N106280 X6.21876 Y0.780845 Z5.14423

N106290 X6.15121 Y0.780845 Z5.24895

N106300 X6.13698 Y0.780845 Z5.2705

N106310 X6.11239 Y0.780845 Z5.30565

N106320 X6.04229 Y0.780845 Z5.40472

N106330 X5.99062 Y0.780845 Z5.47458

N106340 X5.94157 Y0.780845 Z5.53863

N106350 X5.86573 Y0.780845 Z5.63324

N106360 X5.84425 Y0.780845 Z5.65917

N106370 X5.75072 Y0.780845 Z5.7682

N106380 X5.66571 Y0.780845 Z5.86026

N106390 X5.65237 Y0.780845 Z5.87423

N106400 X5.59189 Y0.780845 Z5.93436

N106410 X5.53729 Y0.780845 Z5.98471

N106420 X5.52169 Y0.780845 Z5.99853

N106430 X5.51191 Y0.780845 Z6.00647

N106440 X5.44868 Y0.780845 Z6.05936

N106450 X5.37734 Y0.780845 Z6.11854

N106460 X5.3414 Y0.780845 Z6.14475

N106470 X5.25204 Y0.780845 Z6.21012

N106480 X5.22619 Y0.780845 Z6.22655

N106490 X5.1325 Y0.780845 Z6.28724

N106500 X5.08242 Y0.780845 Z6.31582

N106510 X5.01641 Y0.780845 Z6.35409

N106520 X4.94619 Y0.780845 Z6.39085

N106530 X4.89798 Y0.780845 Z6.41558

N106540 X4.79333 Y0.780845 Z6.46412

N106550 X4.77084 Y0.780845 Z6.47428

N106560 X4.66288 Y0.780845 Z6.51858

N106570 X4.638 Y0.780845 Z6.52836

N106580 X4.54389 Y0.780845 Z6.56193

N106590 X4.50187 Y0.780845 Z6.57589

N106600 X4.43404 Y0.780845 Z6.59663

N106610 X4.33845 Y0.780845 Z6.62254

N106620 X4.32282 Y0.780845 Z6.62629

N106630 X4.29509 Y0.780845 Z6.63276

N106640 X4.18058 Y0.780845 Z6.65807

N106650 X4.11689 Y0.780845 Z6.67246

N106660 X3.96816 Y0.780845 Z6.70378

N106670 X3.9075 Y0.780845 Z6.71587

N106680 X3.73235 Y0.780845 Z6.74704

N106690 X3.71772 Y0.780845 Z6.74951

N106700 X3.67803 Y0.780845 Z6.75544

N106710 X3.54774 Y0.780845 Z6.7743

N106720 X3.50576 Y0.780845 Z6.77966

N106730 X3.39695 Y0.780845 Z6.7925

N106740 X3.32636 Y0.780845 Z6.79939

N106750 X3.25583 Y0.780845 Z6.80526

N106760 X3.16739 Y0.780845 Z6.81081

N106770 X3.1163 Y0.780845 Z6.81299

N106780 X3.01871 Y0.780845 Z6.81658

N106790 X2.97428 Y0.780845 Z6.81763

N106800 X2.90073 Y0.780845 Z6.81882

N106810 X2.82523 Y0.780845 Z6.8182

N106820 X2.80058 Y0.780845 Z6.81763

N106830 X2.7772 Y0.780845 Z6.81673

N106840 X2.69498 Y0.780845 Z6.813

N106850 X2.59138 Y0.780845 Z6.80648

N106860 X2.57735 Y0.780845 Z6.80501

N106870 X2.4737 Y0.780845 Z6.79243

N106880 X2.43921 Y0.780845 Z6.7865

N106890 X2.35422 Y0.780845 Z6.77148

N106900 X2.29676 Y0.780845 Z6.76039

N106910 X2.24507 Y0.780845 Z6.74898

N106920 X2.17657 Y0.780845 Z6.73338

N106930 X2.15456 Y0.780845 Z6.72814

N106940 X2.13223 Y0.780845 Z6.7226

N106950 X2.07268 Y0.780845 Z6.70672

N106960 X2.00255 Y0.780845 Z6.68717

N106970 X1.97944 Y0.780845 Z6.6806

N106980 X1.88961 Y0.780845 Z6.65318

N106990 X1.78561 Y0.780845 Z6.62136

N107000 X1.76043 Y0.780845 Z6.61269

N107010 X1.67179 Y0.780845 Z6.58216

N107020 X1.64891 Y0.780845 Z6.57371

N107030 X1.57139 Y0.780845 Z6.54196

N107040 X1.52178 Y0.780845 Z6.52155

N107050 X1.48606 Y0.780845 Z6.50509

N107060 X1.38692 Y0.780845 Z6.45836

N107070 X1.34701 Y0.780845 Z6.43879

N107080 X1.22833 Y0.780845 Z6.37864

N107090 X1.21094 Y0.780845 Z6.36947

N107100 X1.14391 Y0.780845 Z6.33267

N107110 X1.10564 Y0.780845 Z6.31148

N107120 X1.0917 Y0.780845 Z6.3032

N107130 X1.01217 Y0.780845 Z6.25531

N107140 X0.924379 Y0.780845 Z6.20101

N107150 X0.88881 Y0.780845 Z6.17838

N107160 X0.865393 Y0.780845 Z6.16276

N107170 X0.760395 Y0.780845 Z6.09439

N107180 X0.724135 Y0.780845 Z6.06899

N107190 X0.65266 Y0.780845 Z6.01881

N107200 X0.616472 Y0.780845 Z5.9916

N107210 X0.533125 Y0.780845 Z5.9277

N107220 X0.505756 Y0.780845 Z5.90542

N107230 X0.412624 Y0.780845 Z5.82764

N107240 X0.402705 Y0.780845 Z5.8189

N107250 X0.382604 Y0.780845 Z5.80042

N107260 X0.311572 Y0.780845 Z5.73638

N107270 X0.255651 Y0.780845 Z5.68412

N107280 X0.224202 Y0.780845 Z5.65357

N107290 X0.197373 Y0.780845 Z5.62661

N107300 X0.140914 Y0.780845 Z5.5716

N107310 X0.0789102 Y0.780845 Z5.50978

N107320 X0.058082 Y0.780845 Z5.48804

N107330 X0.0280222 Y0.780845 Z5.45596

N107340 X-0.033092 Y0.780845 Z5.39186

N107350 X-0.0675696 Y0.780845 Z5.35537

N107360 X-0.145339 Y0.780845 Z5.26767

N107370 X-0.194587 Y0.780845 Z5.21232

N107380 X-0.2761 Y0.780845 Z5.11666

N107390 X-0.299116 Y0.780845 Z5.08952

N107400 X-0.391912 Y0.780845 Z4.97932

N107410 X-0.419433 Y0.780845 Z4.94705

N107420 X-0.469478 Y0.780845 Z4.88861

N107430 X-0.537452 Y0.780845 Z4.81332

N107440 X-0.548361 Y0.780845 Z4.8017

N107450 X-0.563488 Y0.780845 Z4.78702

N107460 X-0.603805 Y0.780845 Z4.74774

N107470 X-0.615025 Y0.780845 Z4.73786

N107480 X-0.629114 Y0.780845 Z4.72726

N107490 X-0.638537 Y0.780845 Z4.7202

N107500 X-0.650048 Y0.780845 Z4.71172

N107510 X-0.662378 Y0.780845 Z4.70401

N107520 X-0.674582 Y0.780845 Z4.69716

N107530 X-0.685235 Y0.780845 Z4.69229

N107540 X-0.69484 Y0.780845 Z4.68877

N107550 X-0.708651 Y0.780845 Z4.68968

N107560 X-0.728592 Y0.780845 Z4.69086

N107570 X-0.763313 Y0.780845 Z4.69648

N107580 X-0.801632 Y0.780845 Z4.70354

N107590 X-0.847462 Y0.780845 Z4.71586

N107600 X-0.887266 Y0.780845 Z4.72757

N107610 X-0.929726 Y0.780845 Z4.74354

N107620 X-0.948405 Y0.780845 Z4.74962

N107630 X-0.986077 Y0.780845 Z4.76435

N107640 X-1.03647 Y0.780845 Z4.78369

N107650 X-1.05923 Y0.780845 Z4.79369

N107660 X-1.15896 Y0.780845 Z4.83464

N107670 X-1.17638 Y0.780845 Z4.84195

N107680 X-1.29829 Y0.780845 Z4.8913

N107690 X-1.31454 Y0.780845 Z4.89747

N107700 X-1.46203 Y0.780845 Z4.95285

N107710 X-1.64188 Y0.780845 Z5.01358

N107720 X-1.77645 Y0.780845 Z5.05278

N107730 X-1.83099 Y0.780845 Z5.06705

N107740 X-1.88931 Y0.780845 Z5.08243

N107750 X-1.97962 Y0.780845 Z5.10329

N107760 X-2.00512 Y0.780845 Z5.10915

N107770 X-2.01854 Y0.780845 Z5.11156

N107780 X-2.14311 Y0.780845 Z5.13449

N107790 X-2.20537 Y0.780845 Z5.14245

N107800 X-2.32551 Y0.780845 Z5.15834

N107810 X-2.36959 Y0.780845 Z5.16502

N107820 X-2.48247 Y0.780845 Z5.18152

N107830 X-2.52812 Y0.780845 Z5.18806

N107840 X-2.65495 Y0.780845 Z5.20939

N107850 X-2.68238 Y0.780845 Z5.21425

N107860 X-2.77274 Y0.780845 Z5.2313

N107870 X-2.85601 Y0.780845 Z5.24758

N107880 X-2.86594 Y0.780845 Z5.24958

N107890 X-2.92941 Y0.780845 Z5.26326

N107900 X-2.94756 Y0.780845 Z5.26765

N107910 X-2.98574 Y0.780845 Z5.27763

N107920 X-3.00969 Y0.780845 Z5.2844

N107930 X-3.07295 Y0.780845 Z5.3022

N107940 X-3.14006 Y0.780845 Z5.32219

N107950 X-3.15035 Y0.780845 Z5.32594

N107960 X-3.22636 Y0.780845 Z5.3537

N107970 X-3.2939 Y0.780845 Z5.38035

N107980 X-3.34438 Y0.780845 Z5.40032

N107990 X-3.45653 Y0.780845 Z5.44677

N108000 X-3.48506 Y0.780845 Z5.45867

N108010 X-3.50802 Y0.780845 Z5.46847

N108020 X-3.63922 Y0.780845 Z5.52467

N108030 X-3.74222 Y0.780845 Z5.56878

N108040 X-3.78859 Y0.780845 Z5.58871

N108050 X-3.83584 Y0.780845 Z5.6082

N108060 X-3.91726 Y0.780845 Z5.6418

N108070 X-4.0236 Y0.780845 Z5.68118

N108080 X-4.05092 Y0.780845 Z5.69101

N108090 X-4.0796 Y0.780845 Z5.69988

N108100 X-4.1726 Y0.780845 Z5.72854

N108110 X-4.20571 Y0.780845 Z5.73747

N108120 X-4.28604 Y0.780845 Z5.75746

N108130 X-4.33421 Y0.780845 Z5.76756

N108140 X-4.39777 Y0.780845 Z5.78065

N108150 X-4.43903 Y0.780845 Z5.78769

N108160 X-4.50277 Y0.780845 Z5.7982

N108170 X-4.54773 Y0.780845 Z5.80392

N108180 X-4.60531 Y0.780845 Z5.81024

N108190 X-4.67205 Y0.780845 Z5.81424

N108200 X-4.70813 Y0.780845 Z5.81607

N108210 X-4.80116 Y0.780845 Z5.81586

N108220 X-4.81198 Y0.780845 Z5.81546

N108230 X-4.89472 Y0.780845 Z5.80984

N108240 X-4.95861 Y0.780845 Z5.80238

N108250 X-4.9904 Y0.780845 Z5.7976

N108260 X-5.06068 Y0.780845 Z5.78523

N108270 X-5.11412 Y0.780845 Z5.77438

N108280 X-5.15884 Y0.780845 Z5.76389

N108290 X-5.23648 Y0.780845 Z5.74153

N108300 X-5.26738 Y0.780845 Z5.73195

N108310 X-5.33887 Y0.780845 Z5.70483

N108320 X-5.37592 Y0.780845 Z5.69087

N108330 X-5.44688 Y0.780845 Z5.66142

N108340 X-5.4634 Y0.780845 Z5.65423

N108350 X-5.49451 Y0.780845 Z5.63884

N108360 X-5.55176 Y0.780845 Z5.61009

N108370 X-5.5697 Y0.780845 Z5.60064

N108380 X-5.64587 Y0.780845 Z5.55666

N108390 X-5.69378 Y0.780845 Z5.52755

N108400 X-5.75095 Y0.780845 Z5.49096

N108410 X-5.79119 Y0.780845 Z5.46319

N108420 X-5.8536 Y0.780845 Z5.41568

N108430 X-5.87462 Y0.780845 Z5.39817

N108440 X-5.9525 Y0.780845 Z5.32785

N108450 X-5.96142 Y0.780845 Z5.31967

N108460 X-5.96977 Y0.780845 Z5.31123

N108470 X-6.04306 Y0.780845 Z5.23595

N108480 X-6.06999 Y0.780845 Z5.2065

N108490 X-6.11656 Y0.780845 Z5.15063

N108500 X-6.14209 Y0.780845 Z5.1154

N108510 X-6.18472 Y0.780845 Z5.05649

N108520 X-6.20482 Y0.780845 Z5.02665

N108530 X-6.26237 Y0.780845 Z4.9336

N108540 X-6.29438 Y0.780845 Z4.87641

N108550 X-6.30809 Y0.780845 Z4.84895

N108560 X-6.34803 Y0.780845 Z4.76997

N108570 X-6.35367 Y0.780845 Z4.75731

N108580 X-6.3876 Y0.780845 Z4.67804

N108590 X-6.39812 Y0.780845 Z4.65205

N108600 X-6.43204 Y0.780845 Z4.55776

N108610 X-6.44523 Y0.780845 Z4.51711

N108620 X-6.48546 Y0.780845 Z4.38426

N108630 X-6.49384 Y0.780845 Z4.35453

N108640 X-6.50736 Y0.780845 Z4.30759

N108650 X-6.5342 Y0.780845 Z4.20715

N108660 X-6.55431 Y0.780845 Z4.13101

N108670 X-6.55784 Y0.780845 Z4.11779

N108680 X-6.5752 Y0.780845 Z4.05441

N108690 X-6.54061 Y0.658959 Z4.02437

N108700 X-6.52893 Y0.658959 Z4.06727

N108710 X-6.51914 Y0.658959 Z4.10192

N108720 X-6.50307 Y0.658959 Z4.16388

N108730 X-6.48223 Y0.658959 Z4.24447

N108740 X-6.46849 Y0.658959 Z4.29217

N108750 X-6.43122 Y0.658959 Z4.42436

N108760 X-6.42719 Y0.658959 Z4.43766

N108770 X-6.38649 Y0.658959 Z4.5631

N108780 X-6.38237 Y0.658959 Z4.57456

N108790 X-6.34472 Y0.658959 Z4.66765

N108800 X-6.33123 Y0.658959 Z4.69918

N108810 X-6.30496 Y0.658959 Z4.75348

N108820 X-6.27939 Y0.658959 Z4.80607

N108830 X-6.26423 Y0.658959 Z4.83431

N108840 X-6.22535 Y0.658959 Z4.90468

N108850 X-6.21436 Y0.658959 Z4.92313

N108860 X-6.16772 Y0.658959 Z4.99641

N108870 X-6.15415 Y0.658959 Z5.01631

N108880 X-6.10228 Y0.658959 Z5.08811

N108890 X-6.08769 Y0.658959 Z5.1066

N108900 X-6.0262 Y0.658959 Z5.17997

N108910 X-6.00154 Y0.658959 Z5.20829

N108920 X-5.94909 Y0.658959 Z5.26169

N108930 X-5.90171 Y0.658959 Z5.30859

N108940 X-5.85814 Y0.658959 Z5.34854

N108950 X-5.81653 Y0.658959 Z5.38306

N108960 X-5.74722 Y0.658959 Z5.43586

N108970 X-5.72387 Y0.658959 Z5.45212

N108980 X-5.62969 Y0.658959 Z5.5127

N108990 X-5.61088 Y0.658959 Z5.52401

N109000 X-5.52653 Y0.658959 Z5.57221

N109010 X-5.46833 Y0.658959 Z5.60189

N109020 X-5.43614 Y0.658959 Z5.61781

N109030 X-5.39021 Y0.658959 Z5.63779

N109040 X-5.34097 Y0.658959 Z5.65874

N109050 X-5.31258 Y0.658959 Z5.67035

N109060 X-5.2468 Y0.658959 Z5.69495

N109070 X-5.19977 Y0.658959 Z5.71123

N109080 X-5.16032 Y0.658959 Z5.72261

N109090 X-5.10923 Y0.658959 Z5.7355

N109100 X-5.02038 Y0.658959 Z5.75391

N109110 X-5.00928 Y0.658959 Z5.75603

N109120 X-4.99933 Y0.658959 Z5.7576

N109130 X-4.90078 Y0.658959 Z5.77219

N109140 X-4.83847 Y0.658959 Z5.77806

N109150 X-4.799 Y0.658959 Z5.78096

N109160 X-4.7708 Y0.658959 Z5.78135

N109170 X-4.69407 Y0.658959 Z5.7823

N109180 X-4.6171 Y0.658959 Z5.77822

N109190 X-4.58774 Y0.658959 Z5.77622

N109200 X-4.48604 Y0.658959 Z5.7638

N109210 X-4.38107 Y0.658959 Z5.74601

N109220 X-4.26471 Y0.658959 Z5.72191

N109230 X-4.1484 Y0.658959 Z5.69055

N109240 X-4.10586 Y0.658959 Z5.67744

N109250 X-4.02643 Y0.658959 Z5.65166

N109260 X-3.93259 Y0.658959 Z5.61711

N109270 X-3.90244 Y0.658959 Z5.60595

N109280 X-3.86667 Y0.658959 Z5.59119

N109290 X-3.7654 Y0.658959 Z5.54937

N109300 X-3.61544 Y0.658959 Z5.48518

N109310 X-3.46532 Y0.658959 Z5.42127

N109320 X-3.33822 Y0.658959 Z5.36854

N109330 X-3.31794 Y0.658959 Z5.36039

N109340 X-3.21972 Y0.658959 Z5.32191

N109350 X-3.18931 Y0.658959 Z5.31093

N109360 X-3.13279 Y0.658959 Z5.29084

N109370 X-3.04597 Y0.658959 Z5.26302

N109380 X-3.03577 Y0.658959 Z5.26001

N109390 X-2.93829 Y0.658959 Z5.23245

N109400 X-2.92707 Y0.658959 Z5.22952

N109410 X-2.87212 Y0.658959 Z5.21606

N109420 X-2.85582 Y0.658959 Z5.21256

N109430 X-2.80718 Y0.658959 Z5.20185

N109440 X-2.76108 Y0.658959 Z5.19249

N109450 X-2.73449 Y0.658959 Z5.18715

N109460 X-2.71461 Y0.658959 Z5.18302

N109470 X-2.63286 Y0.658959 Z5.16753

N109480 X-2.57068 Y0.658959 Z5.15546

N109490 X-2.51193 Y0.658959 Z5.14547

N109500 X-2.40825 Y0.658959 Z5.12745

N109510 X-2.3774 Y0.658959 Z5.12294

N109520 X-2.31624 Y0.658959 Z5.11368

N109530 X-2.2236 Y0.658959 Z5.09801

N109540 X-2.17545 Y0.658959 Z5.08757

N109550 X-1.98602 Y0.658959 Z5.047

N109560 X-1.97631 Y0.658959 Z5.04446

N109570 X-1.88031 Y0.658959 Z5.01893

N109580 X-1.79194 Y0.658959 Z4.99346

N109590 X-1.64823 Y0.658959 Z4.94638

N109600 X-1.53997 Y0.658959 Z4.9068

N109610 X-1.43419 Y0.658959 Z4.86566

N109620 X-1.39506 Y0.658959 Z4.85085

N109630 X-1.27439 Y0.658959 Z4.80152

N109640 X-1.25327 Y0.658959 Z4.79298

N109650 X-1.15105 Y0.658959 Z4.7513

N109660 X-1.13371 Y0.658959 Z4.74425

N109670 X-1.06743 Y0.658959 Z4.71884

N109680 X-1.03421 Y0.658959 Z4.70735

N109690 X-1.00322 Y0.658959 Z4.69685

N109700 X-0.965404 Y0.658959 Z4.68619

N109710 X-0.950628 Y0.658959 Z4.68129

N109720 X-0.939108 Y0.658959 Z4.67837

N109730 X-0.903434 Y0.658959 Z4.66896

N109740 X-0.869063 Y0.658959 Z4.66282

N109750 X-0.856508 Y0.658959 Z4.66039

N109760 X-0.824567 Y0.658959 Z4.65536

N109770 X-0.793997 Y0.658959 Z4.65325

N109780 X-0.777647 Y0.658959 Z4.65245

N109790 X-0.767322 Y0.658959 Z4.65527

N109800 X-0.748774 Y0.658959 Z4.66713

N109810 X-0.727777 Y0.658959 Z4.68159

N109820 X-0.71922 Y0.658959 Z4.68897

N109830 X-0.705971 Y0.658959 Z4.69852

N109840 X-0.685085 Y0.658959 Z4.7175

N109850 X-0.663299 Y0.658959 Z4.73605

N109860 X-0.633819 Y0.658959 Z4.76799

N109870 X-0.620145 Y0.658959 Z4.78124

N109880 X-0.560727 Y0.658959 Z4.85048

N109890 X-0.552916 Y0.658959 Z4.85938

N109900 X-0.493576 Y0.658959 Z4.93093

N109910 X-0.348595 Y0.658959 Z5.10321

N109920 X-0.297666 Y0.658959 Z5.16231

N109930 X-0.243875 Y0.658959 Z5.22349

N109940 X-0.200626 Y0.658959 Z5.27204

N109950 X-0.13869 Y0.658959 Z5.33774

N109960 X-0.0859437 Y0.658959 Z5.39343

N109970 X-0.044185 Y0.658959 Z5.43456

N109980 X0.0241067 Y0.658959 Z5.50173

N109990 X0.0416745 Y0.658959 Z5.51911

N110000 X0.123815 Y0.658959 Z5.59611

N110010 X0.184328 Y0.658959 Z5.65113

N110020 X0.2058 Y0.658959 Z5.67114

N110030 X0.230744 Y0.658959 Z5.6938

N110040 X0.29376 Y0.658959 Z5.74841

N110050 X0.38125 Y0.658959 Z5.82212

N110060 X0.389723 Y0.658959 Z5.82939

N110070 X0.495868 Y0.658959 Z5.91264

N110080 X0.528619 Y0.658959 Z5.93768

N110090 X0.607066 Y0.658959 Z5.99353

N110100 X0.65629 Y0.658959 Z6.02807

N110110 X0.722931 Y0.658959 Z6.07151

N110120 X0.783602 Y0.658959 Z6.111

N110130 X0.846838 Y0.658959 Z6.14936

N110140 X0.948872 Y0.658959 Z6.21197

N110150 X0.960427 Y0.658959 Z6.21863

N110160 X1.00639 Y0.658959 Z6.24499

N110170 X1.0796 Y0.658959 Z6.28703

N110180 X1.09435 Y0.658959 Z6.29513

N110190 X1.21746 Y0.658959 Z6.36012

N110200 X1.325 Y0.658959 Z6.41289

N110210 X1.35637 Y0.658959 Z6.42824

N110220 X1.4845 Y0.658959 Z6.48611

N110230 X1.53794 Y0.658959 Z6.50883

N110240 X1.59205 Y0.658959 Z6.5311

N110250 X1.6227 Y0.658959 Z6.54229

N110260 X1.69476 Y0.658959 Z6.56862

N110270 X1.74626 Y0.658959 Z6.58528

N110280 X1.78246 Y0.658959 Z6.59629

N110290 X1.82917 Y0.658959 Z6.61073

N110300 X1.88177 Y0.658959 Z6.62569

N110310 X1.95493 Y0.658959 Z6.64683

N110320 X1.98347 Y0.658959 Z6.65487

N110330 X1.99739 Y0.658959 Z6.65885

N110340 X2.09631 Y0.658959 Z6.68444

N110350 X2.11169 Y0.658959 Z6.68825

N110360 X2.14181 Y0.658959 Z6.69542

N110370 X2.21747 Y0.658959 Z6.71208

N110380 X2.33236 Y0.658959 Z6.73558

N110390 X2.34773 Y0.658959 Z6.73855

N110400 X2.38448 Y0.658959 Z6.74489

N110410 X2.47376 Y0.658959 Z6.75943

N110420 X2.50634 Y0.658959 Z6.76323

N110430 X2.57466 Y0.658959 Z6.76981

N110440 X2.64488 Y0.658959 Z6.77491

N110450 X2.68033 Y0.658959 Z6.77696

N110460 X2.71151 Y0.658959 Z6.77814

N110470 X2.78654 Y0.658959 Z6.78036

N110480 X2.86646 Y0.658959 Z6.78141

N110490 X2.89799 Y0.658959 Z6.78152

N110500 X2.96066 Y0.658959 Z6.7805

N110510 X3.02774 Y0.658959 Z6.77892

N110520 X3.04735 Y0.658959 Z6.7782

N110530 X3.15081 Y0.658959 Z6.77377

N110540 X3.18862 Y0.658959 Z6.77193

N110550 X3.32954 Y0.658959 Z6.75953

N110560 X3.37139 Y0.658959 Z6.75484

N110570 X3.47995 Y0.658959 Z6.74149

N110580 X3.58498 Y0.658959 Z6.72668

N110590 X3.64897 Y0.658959 Z6.71712

N110600 X3.83596 Y0.658959 Z6.68551

N110610 X4.05411 Y0.658959 Z6.64212

N110620 X4.22523 Y0.658959 Z6.60349

N110630 X4.26723 Y0.658959 Z6.59421

N110640 X4.36051 Y0.658959 Z6.57068

N110650 X4.44787 Y0.658959 Z6.5471

N110660 X4.47621 Y0.658959 Z6.5385

N110670 X4.59353 Y0.658959 Z6.49948

N110680 X4.73049 Y0.658959 Z6.44573

N110690 X4.87204 Y0.658959 Z6.3821

N110700 X4.89744 Y0.658959 Z6.36947

N110710 X5.01292 Y0.658959 Z6.31111

N110720 X5.12264 Y0.658959 Z6.24854

N110730 X5.14713 Y0.658959 Z6.23497

N110740 X5.27875 Y0.658959 Z6.15111

N110750 X5.29829 Y0.658959 Z6.13703

N110760 X5.41612 Y0.658959 Z6.05111

N110770 X5.43054 Y0.658959 Z6.0391

N110780 X5.52947 Y0.658959 Z5.95866

N110790 X5.54527 Y0.658959 Z5.94467

N110800 X5.6118 Y0.658959 Z5.88394

N110810 X5.63161 Y0.658959 Z5.86418

N110820 X5.70548 Y0.658959 Z5.78845

N110830 X5.71739 Y0.658959 Z5.77553

N110840 X5.80009 Y0.658959 Z5.68252

N110850 X5.80947 Y0.658959 Z5.67196

N110860 X5.90589 Y0.658959 Z5.55515

N110870 X6.0322 Y0.658959 Z5.39046

N110880 X6.07922 Y0.658959 Z5.32413

N110890 X6.11996 Y0.658959 Z5.26593

N110900 X6.17428 Y0.658959 Z5.18363

N110910 X6.1999 Y0.658959 Z5.14417

N110920 X6.20608 Y0.658959 Z5.13424

N110930 X6.25227 Y0.658959 Z5.05917

N110940 X6.2755 Y0.658959 Z5.02078

N110950 X6.34583 Y0.658959 Z4.88748

N110960 X6.37039 Y0.658959 Z4.8362

N110970 X6.41907 Y0.658959 Z4.73126

N110980 X6.42341 Y0.658959 Z4.7214

N110990 X6.43625 Y0.658959 Z4.69091

N111000 X6.47384 Y0.658959 Z4.60171

N111010 X6.48678 Y0.658959 Z4.56979

N111020 X6.52112 Y0.658959 Z4.47726

N111030 X6.54181 Y0.658959 Z4.41692

N111040 X6.5656 Y0.658959 Z4.34247

N111050 X6.58459 Y0.658959 Z4.27778

N111060 X6.60358 Y0.658959 Z4.21118

N111070 X6.62073 Y0.658959 Z4.14672

N111080 X6.62952 Y0.658959 Z4.11463

N111090 X6.64308 Y0.658959 Z4.06579

N111100 X6.6533 Y0.658959 Z4.02909

N111110 X6.63522 Y0.538845 Z4.0243

N111120 X6.62324 Y0.538845 Z4.06765

N111130 X6.61586 Y0.538845 Z4.09435

N111140 X6.60076 Y0.538845 Z4.14937

N111150 X6.59019 Y0.538845 Z4.18583

N111160 X6.56405 Y0.538845 Z4.27746

N111170 X6.55592 Y0.538845 Z4.30454

N111180 X6.51817 Y0.538845 Z4.42273

N111190 X6.508 Y0.538845 Z4.45168

N111200 X6.47038 Y0.538845 Z4.55366

N111210 X6.44618 Y0.538845 Z4.61472

N111220 X6.42102 Y0.538845 Z4.67448

N111230 X6.37751 Y0.538845 Z4.77346

N111240 X6.36402 Y0.538845 Z4.80254

N111250 X6.34374 Y0.538845 Z4.84488

N111260 X6.31243 Y0.538845 Z4.91023

N111270 X6.23526 Y0.538845 Z5.051

N111280 X6.21268 Y0.538845 Z5.08771

N111290 X6.17208 Y0.538845 Z5.1529

N111300 X6.13386 Y0.538845 Z5.21175

N111310 X6.1024 Y0.538845 Z5.25925

N111320 X6.0389 Y0.538845 Z5.35044

N111330 X6.02909 Y0.538845 Z5.36389

N111340 X5.96265 Y0.538845 Z5.45225

N111350 X5.89807 Y0.538845 Z5.5334

N111360 X5.87868 Y0.538845 Z5.55752

N111370 X5.86512 Y0.538845 Z5.57396

N111380 X5.77804 Y0.538845 Z5.67686

N111390 X5.76093 Y0.538845 Z5.69612

N111400 X5.69087 Y0.538845 Z5.77163

N111410 X5.66288 Y0.538845 Z5.80034

N111420 X5.60765 Y0.538845 Z5.85374

N111430 X5.56898 Y0.538845 Z5.88903

N111440 X5.51871 Y0.538845 Z5.9329

N111450 X5.46691 Y0.538845 Z5.9749

N111460 X5.41733 Y0.538845 Z6.01404

N111470 X5.3543 Y0.538845 Z6.05892

N111480 X5.29646 Y0.538845 Z6.10118

N111490 X5.19521 Y0.538845 Z6.16451

N111500 X5.16891 Y0.538845 Z6.18126

N111510 X5.07339 Y0.538845 Z6.23416

N111520 X5.01909 Y0.538845 Z6.26453

N111530 X5.0057 Y0.538845 Z6.27131

N111540 X4.92623 Y0.538845 Z6.31024

N111550 X4.8517 Y0.538845 Z6.34728

N111560 X4.83558 Y0.538845 Z6.35453

N111570 X4.71336 Y0.538845 Z6.40796

N111580 X4.67687 Y0.538845 Z6.4223

N111590 X4.57846 Y0.538845 Z6.45933

N111600 X4.52953 Y0.538845 Z6.4756

N111610 X4.39633 Y0.538845 Z6.51563

N111620 X4.27208 Y0.538845 Z6.54693

N111630 X4.22367 Y0.538845 Z6.55836

N111640 X4.10592 Y0.538845 Z6.58436

N111650 X4.00034 Y0.538845 Z6.60688

N111660 X3.90619 Y0.538845 Z6.62567

N111670 X3.7826 Y0.538845 Z6.64919

N111680 X3.66776 Y0.538845 Z6.66908

N111690 X3.58882 Y0.538845 Z6.68204

N111700 X3.41772 Y0.538845 Z6.70728

N111710 X3.3958 Y0.538845 Z6.70996

N111720 X3.27479 Y0.538845 Z6.72351

N111730 X3.23998 Y0.538845 Z6.7266

N111740 X3.14092 Y0.538845 Z6.73429

N111750 X3.07116 Y0.538845 Z6.73768

N111760 X3.00377 Y0.538845 Z6.74029

N111770 X2.91363 Y0.538845 Z6.74237

N111780 X2.88264 Y0.538845 Z6.74287

N111790 X2.84739 Y0.538845 Z6.74275

N111800 X2.76973 Y0.538845 Z6.74167

N111810 X2.68326 Y0.538845 Z6.73918

N111820 X2.66562 Y0.538845 Z6.73852

N111830 X2.65149 Y0.538845 Z6.7377

N111840 X2.55894 Y0.538845 Z6.73073

N111850 X2.45663 Y0.538845 Z6.71968

N111860 X2.43456 Y0.538845 Z6.71667

N111870 X2.32202 Y0.538845 Z6.69779

N111880 X2.19219 Y0.538845 Z6.6733

N111890 X2.17535 Y0.538845 Z6.66985

N111900 X2.16037 Y0.538845 Z6.66656

N111910 X2.02833 Y0.538845 Z6.63608

N111920 X1.99762 Y0.538845 Z6.62812

N111930 X1.88774 Y0.538845 Z6.59909

N111940 X1.86755 Y0.538845 Z6.59355

N111950 X1.76331 Y0.538845 Z6.56189

N111960 X1.69668 Y0.538845 Z6.54118

N111970 X1.64629 Y0.538845 Z6.52444

N111980 X1.57482 Y0.538845 Z6.49847

N111990 X1.5249 Y0.538845 Z6.47917

N112000 X1.46755 Y0.538845 Z6.45496

N112010 X1.36849 Y0.538845 Z6.41261

N112020 X1.33906 Y0.538845 Z6.39918

N112030 X1.22589 Y0.538845 Z6.34658

N112040 X1.19846 Y0.538845 Z6.33315

N112050 X1.09295 Y0.538845 Z6.28024

N112060 X1.05695 Y0.538845 Z6.26129

N112070 X0.954631 Y0.538845 Z6.20605

N112080 X0.928745 Y0.538845 Z6.19121

N112090 X0.860741 Y0.538845 Z6.15198

N112100 X0.814981 Y0.538845 Z6.12568

N112110 X0.796746 Y0.538845 Z6.11526

N112120 X0.714433 Y0.538845 Z6.06539

N112130 X0.668584 Y0.538845 Z6.03754

N112140 X0.605675 Y0.538845 Z5.99655

N112150 X0.537306 Y0.538845 Z5.95193

N112160 X0.491808 Y0.538845 Z5.91964

N112170 X0.382505 Y0.538845 Z5.84063

N112180 X0.373567 Y0.538845 Z5.83351

N112190 X0.283709 Y0.538845 Z5.76338

N112200 X0.207719 Y0.538845 Z5.70211

N112210 X0.194665 Y0.538845 Z5.69108

N112220 X0.184059 Y0.538845 Z5.68184

N112230 X0.112072 Y0.538845 Z5.6202

N112240 X0.026301 Y0.538845 Z5.54401

N112250 X-0.051842 Y0.538845 Z5.47052

N112260 X-0.101607 Y0.538845 Z5.42415

N112270 X-0.138479 Y0.538845 Z5.38759

N112280 X-0.216852 Y0.538845 Z5.3094

N112290 X-0.230644 Y0.538845 Z5.29473

N112300 X-0.314525 Y0.538845 Z5.20249

N112310 X-0.330953 Y0.538845 Z5.18391

N112320 X-0.417679 Y0.538845 Z5.08165

N112330 X-0.430707 Y0.538845 Z5.06616

N112340 X-0.458697 Y0.538845 Z5.03234

N112350 X-0.576582 Y0.538845 Z4.88627

N112360 X-0.60754 Y0.538845 Z4.8471

N112370 X-0.617636 Y0.538845 Z4.8355

N112380 X-0.700623 Y0.538845 Z4.73726

N112390 X-0.750122 Y0.538845 Z4.68292

N112400 X-0.767556 Y0.538845 Z4.66508

N112410 X-0.783776 Y0.538845 Z4.65021

N112420 X-0.795218 Y0.538845 Z4.64034

N112430 X-0.816685 Y0.538845 Z4.62239

N112440 X-0.832323 Y0.538845 Z4.61133

N112450 X-0.841994 Y0.538845 Z4.60737

N112460 X-0.86057 Y0.538845 Z4.6047

N112470 X-0.884989 Y0.538845 Z4.60517

N112480 X-0.897323 Y0.538845 Z4.60654

N112490 X-0.91432 Y0.538845 Z4.60724

N112500 X-0.928553 Y0.538845 Z4.60931

N112510 X-0.987249 Y0.538845 Z4.62025

N112520 X-1.00208 Y0.538845 Z4.62329

N112530 X-1.0407 Y0.538845 Z4.63282

N112540 X-1.0726 Y0.538845 Z4.6424
[truncated: 472,150 more chars]
