# Supplementary material for: B-spline curve fitting based on dynamic adjustment of knot vector using feature points
Source: PLoS One. 2025 Jun 27;20(6):e0325458. doi: 10.1371/journal.pone.0325458 (PMC12204621; doi:10.1371/journal.pone.0325458)
Supplement: Supporting information — (DOCX) [file pone.0325458.s003.docx]

The experiment is conducted under the Dental CAD/CAM prototype system developed by the author's team in the early stage, using VC++ and HOOPs as development tools. The B-spline tool path generation for CNC machining of the outer surface of human molar is used as an example. The triangular mesh model of teeth is reconstructed from 3D laser point cloud data, and figure 3 (a) shows the three-dimensional mesh model of molar with lighting effect in Dental CAD/CAM. The molars model is a triangular mesh model with 6064 vertices and 11887 triangular pieces, and its size of the bounding box is 15mm×15mm×6mm.The data points to be fitted in the planes are obtained by intersecting a series of section planes and triangular mesh model, as shown in figure 3 (b), the discrete data points in each plane are a set of test data. In this experiment, the spacing between the section planes is 0.1mm, with a total of 148 section planes, resulting in 148 sets of test data. Fig. 3(c) shows the linear tool paths using the section plane method in Dental CAD/CAM.Fig.3(d) shows the linear tool path with section plane of $XOZ$, with a total of 163 data points.

Traditional least squares fitting, DOM, and DAKM algorithms are used to fit B-spline curve. In traditional least squares fitting, knot vectors are calculated using KTP method. In DOM and DAKM algorithms, knot vectors are calculated using AVG method. Therefore, the traditional least squares fitting method is abbreviated as KTP algorithm.

The data points in the *XOZ* plane are fitted using KTP, DOM, and DAKM algorithm, respectively.Fig.4 shows a comparison of the knot value distributions of three algorithms. From the distribution of inner knot values, the KTP algorithm has a relatively uniform distribution and the largest number of nodes in knot vector, followed by the DOM algorithm, while the DAKM algorithm has a less uniform distribution and the smallest number of nodes. The more evenly and numerous the node values are distributed, the greater the number of control points are required. Table 1 shows the experimental result using different fitting algorithms, indicating that the B-spline curve fitted by the DAKM algorithm requires fewer control points, has a shorter execution time, and a smaller fitting error.

Fig 3. (a) and Fig 3. (c) have been marked（Reprinted from [31] under a CC BY license, with permission from [The Science Press], original copyright [2010]）
