# Supplementary material for: B-spline curve fitting based on dynamic adjustment of knot vector using feature points
Source: PLoS One. 2025 Jun 27;20(6):e0325458. doi: 10.1371/journal.pone.0325458 (PMC12204621; doi:10.1371/journal.pone.0325458)
Supplement: Supporting information — (DOCX) [file pone.0325458.s004.docx]

//Improved DOM:DAKM

void CDentalCADDoc::getBSplineByDAKM()

{

/*Basic steps of DAKM:

1. Initial parameterization of data points;

2. Discrete curvature calculation;

3. Selection of initial feature points, the number of initial feature points = the number of initial control vertices;

4. Parametric AVG method of feature points;

5. Fitting B-spline curve by least square method;

6. Calculate the maximum deviation of the fitting curve;

7. Calculate the nearest parameter point and its parameters;

8. Replace the original parameter value with the parameter value of the nearest parameter point;

9. Select new feature points in the curve segment where the maximum deviation is located (the principle of maximum included angle between adjacent straight line segments);

10. Insert the parameter value corresponding to the new feature point into the knot vector (that is, insert one knot), and then add 1 to the number of control vertices;

11. Turn to 5 until the maximum deviation is less than the fitting accuracy, and the algorithm ends.

*/

//Model vertex equidistant

float r=0.5;

nc_maching.r=r;

nc_maching.offsetMeshByVertex(mesh_io,offset_mesh_io);

//Fitting accuracy

float delt=0.05;

//Linked list of data points to be fitted

vector<Point3D> clPointList;

//Fitting curve knot vector linked list

vector<vector<float> > uListList;

//Fitting curve control polygon length linked list

vector<vector<float> > conPolyLengthListList;

//Calculate the fitted data points

int no=0;

clPointList.clear();

//Calculate the intersection of plane y=0 and mesh model

nc_maching.calToolPath(0.0, 1.0, 0.0,d,offset_mesh_io,toolPath);

for(tpIter=toolPath.begin();tpIter!=toolPath.end();++tpIter)

{ //Data preprocessing

if((*tpIter).getDistance()>delt)

{ //Obtaining data point linked list

Point3D cl_pt=(*tpIter)._pt1;

clPointList.push_back(cl_pt);

}//end if

}//end for tpIter

//1. //Parameterization of initial point of data (parameterization of cumulative chord length);

vector<float> ptUList=nc_maching.nurbs_clPtsParametrization(clPointList);

//2.Discrete curvature calculation;

int i,j;

//Arc radius corresponding to data point

vector<float> ptRList;

//Circular arc linked list obtained by fitting

vector<CArc> arcList;

//Fitting accuracy

float fit_delt=delt;

nc_maching.getCurvatureRadiusByLeastSquare(fit_delt,clPointList,ptRList,arcList);

float r_min=*min_element(ptRList.begin(),ptRList.end());

float r_max=*max_element(ptRList.begin(),ptRList.end());

//3.Select the initial feature points, the number = the number of initial control vertices;

vector<Point3D> seedPtList; //Seed store linked list

vector<float> seedPtUList; //Seed point corresponding parameter linked list

vector<int> seedPtNoInClPtsList;// Serial number linked list of data points corresponding to seed points.

seedPtUList.push_back(ptUList[0]);

seedPtNoInClPtsList.push_back(0);

//Obtaining initial feature points

nc_maching.getInitialFeaturePoints(clPointList,seedPtList,seedPtUList,seedPtNoInClPtsList);

int m=ptUList.size()-1;

seedPtUList.push_back(ptUList[m]);

seedPtNoInClPtsList.push_back(m);

///////////////////

int seed_num=0;

float e_max;

//Control vertex linked list

vector<Point3D> conPtList;

vector<float> conPolyLengthList;

//Knot vector

vector<float> uList;

//cl_pt_flag_list Mark clPointList status:

//1-: Feature points have been added, and cannot be added again.

//0-: Feature points have not been added, so you can add them.

vector<int> cl_pt_flag_list;

for(i=0;i<clPointList.size();i++)

cl_pt_flag_list.push_back(0);

///////////////////////////////////

//Iterations

int iter_num=0;

do{

//4.Parametric AVG method for feature points;

//The number of initial feature points = the number of initial control vertices, and the corresponding n value is: seed_num--;

seed_num=seedPtUList.size();

seed_num=seed_num-1;

//Knot vector

uList.clear();

nc_maching.nurbs_calKnotVectorByAVG(seed_num,seedPtUList,uList);

//nc_maching.nurbs_calKnotVectorByKTP(seed_num,seedPtUList,uList);

//5.Fitting b-spline curve by least square method;

conPtList.clear();

//seed_num-- //Control the number of vertices

nc_maching.nurbs_calConPtsByClPtsAndConPtsNum(clPointList,seed_num,ptUList,uList,conPtList);

//6.Calculate the maximum deviation of fitting curve;

//7.Calculate the nearest parameter point and its parameters;

vector<Point3D> nearPtOnCurveList;

vector<float> new_ptUList,disList;

nc_maching.nurbs_calNearestPts_disList_ptUList_OfClPts(delt,clPointList,ptUList,uList,conPtList,nearPtOnCurveList,new_ptUList,disList);

//8.Replace the original parameter value with the parameter value of the nearest parameter point;

//ptUList.clear();

//ptUList=new_ptUList;

// Determine the serial number of the data point corresponding to the maximum value in disList, that is, the corresponding serial number j0 in the parameterized list.

int d_num=disList.size();

int e_max_no=-1;

int j0;

e_max=-10.0;

for(j0=0;j0<d_num;j0++)

{ if(disList[j0]>e_max)

{ e_max=disList[j0];

e_max_no=j0;

}

}//end for

//Calculate piecewise connection points on curves

vector<Point3D> connectPtsOnCurveList;

//Add the starting point of the connection point

connectPtsOnCurveList.push_back(clPointList.front());

for(i=3;i<seed_num;i++)

{ Point3D temp_pt;

float u0=seedPtUList[i];

nc_maching.nurbs_calPointOnNurbsCurve(u0,uList,conPtList,temp_pt);

connectPtsOnCurveList.push_back(temp_pt);

}

//Add connection point end point

connectPtsOnCurveList.push_back(clPointList.back());

//The data point corresponding to the maximum value

Point3D pt_e_max=clPointList[e_max_no];

// Indicates that the point has been added.

cl_pt_flag_list[e_max_no]=1;

// Determine the curve segment where the point corresponding to the maximum value is located: x1<=pt_e_max.x<=x2.

float x1,x2;

int connect_pt_num=connectPtsOnCurveList.size();

for(i=0;i<connect_pt_num-1;i++)

{ float xx1,xx2;

xx1=connectPtsOnCurveList[i].x;

xx2=connectPtsOnCurveList[i+1].x;

if((pt_e_max.x>xx1) && (pt_e_max.x<xx2))

{ x1=xx1;

x2=xx2;

}

}

vector<Point3D> temp_pt_list;//Store points with x between [x1,x2]

vector<int> temp_pt_no_list;//The serial number in clPointList corresponding to temp_pt_list.

//One-to-one correspondence between data points and parameterization

for(i=0;i<clPointList.size();i++)

{ float x0=clPointList[i].x;

if(x0>=x1 && x0<=x2)

{ temp_pt_list.push_back(clPointList[i]);

temp_pt_no_list.push_back(i);

}

}

//9. Select new feature points in the curve segment where the maximum deviation exists (the principle of maximum included angle between adjacent straight lines);

//temp_pt_list find the point j1 with the largest included angle in.

int j1=0;

float pi=3.1415926;

//intersection angle

float sita_max=-1;

//In temp_pt_list，sequence number of initial point in clPointList

int init_pt_no=temp_pt_no_list.front();

for(i=1;i<temp_pt_list.size()-1;i++)

{ Point3D pt1,pt2,pt3;

pt1=temp_pt_list[i-1];

pt2=temp_pt_list[i];

pt3=temp_pt_list[i+1];

CSVec3 vec2_1,vec2_3;

vec2_1.x=pt1.x-pt2.x;

vec2_1.y=pt1.y-pt2.y;

vec2_1.z=pt1.z-pt2.z;

vec2_3.x=pt3.x-pt2.x;

vec2_3.y=pt3.y-pt2.y;

vec2_3.z=pt3.z-pt2.z;

vec2_1.normalize();

vec2_3.normalize();

float sita=pi-acos(vec2_1.dot(vec2_3));

//Add non-repeating points

int f=cl_pt_flag_list[i];

if((sita>=sita_max) && (f==0))

{ sita_max=sita;

j1=i;

}

}//end for

//10. Insert the parameter value corresponding to the new feature point into the knot vector (that is, insert one knot), and then add 1 to the number of control vertices;

j1+=init_pt_no;

seedPtUList.push_back(ptUList[j1]);

seedPtNoInClPtsList.push_back(j1);

//Replace the original parameter value with the parameter value of the nearest parameter point;

ptUList.clear();

ptUList=new_ptUList;

int a=seedPtUList.size();

int b=seedPtNoInClPtsList.size();

//Parameter value correction of feature points;

int seed_pt_num=seedPtUList.size();

seedPtUList.clear();

for(i=0;i<seed_pt_num;i++)

{

int jj=seedPtNoInClPtsList[i];

seedPtUList.push_back(ptUList[jj]);

}

sort(seedPtUList.begin(),seedPtUList.end());

iter_num++;//Count the number of iterations

connectPtsOnCurveList.clear();

temp_pt_list.clear();

temp_pt_no_list.clear();

}while(e_max>=delt);//11. Turn to 5 until the maximum deviation is less than the fitting accuracy, and the algorithm ends.

//At this point, a BSpline fitting curve is obtained.

}//end void
